# Supplementary material for: FNDC1 is a myokine that promotes myogenesis and muscle regeneration
Source: EMBO J. 2024 Nov 20;44(1):30–53. doi: 10.1038/s44318-024-00285-0 (PMC11695938; doi:10.1038/s44318-024-00285-0)
Supplement: Supplementary file 1 — Appendix [file 44318_2024_285_MOESM1_ESM.pdf]

FNDC1 is a myokine that promotes myogenesis and muscle regeneration

Rui Xin Zhang [1, #], Yuan Yuan Zhai [1, #], Rong Rong Ding [1, #], Jia He Huang [1], Xiao Chen Shi [1], Huan Liu [1], Xiao Peng Liu [1], Jian Feng Zhang [1], Jun Feng Lu [1], Zhe Zhang [1], Xiang Kai Leng [1], De Fu Li [1], Jun Ying Xiao [1], Bo Xia [1], and Jiang Wei Wu [1, *]

[1] Key Laboratory of Animal Genetics, Breeding and Reproduction of Shaanxi Province, College of Animal Science and Technology, Northwest A&F University, Yangling, Shaanxi 712100, China

[#] equally contributed to this work

* Corresponding authors: Jiang Wei Wu, E-mail: wujiangwei@nwafu.edu.cn

Table of contents page

-Appendix Figure S1 to S21 2-36
-Appendix Tables S1 to S3 37-38

Appendix Figure S1

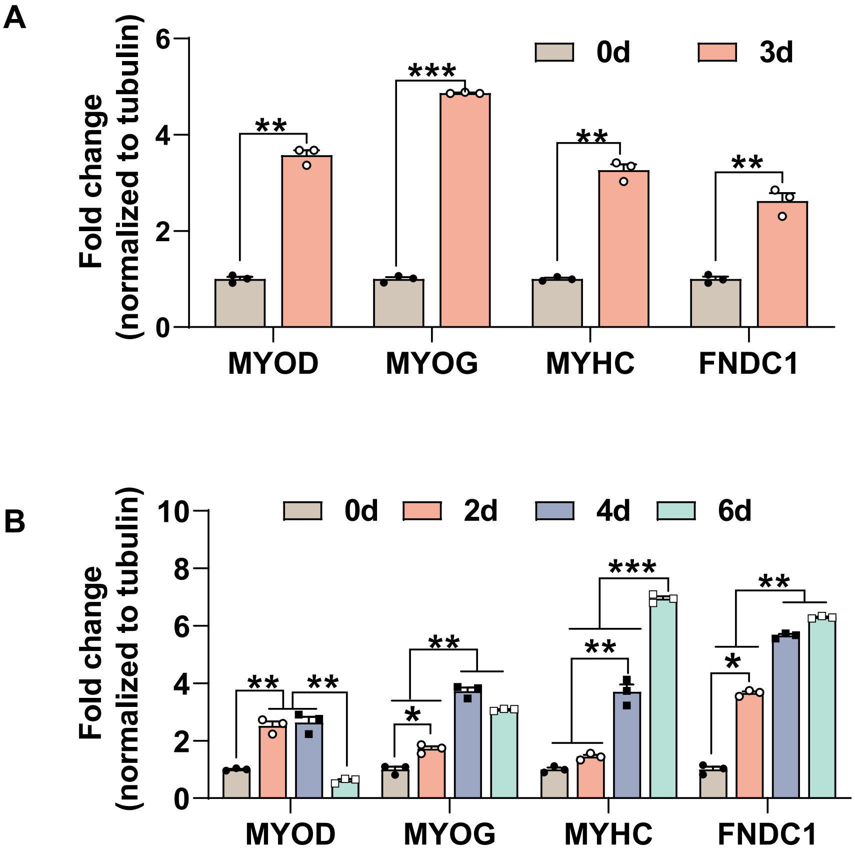

Appendix Figure S1. FNDC1 is closely associated with myogenesis.

(A-B) Quantification of MYOD, MYOG, MYHC, and FNDC1 protein levels in primary myoblasts (A) and C2C12 cells (B) during myogenic differentiation at the indicated time points (n = 3 independent experiments). Two-tailed t-test (A) or One-way ANOVA (B) was performed to compare all listed conditions unless otherwise noted, and data are represented as mean ± SEM. *p < 0.05, **p < 0.01, and ***p < 0.001. Source data are provided as a Source data file.

Appendix Figure S2

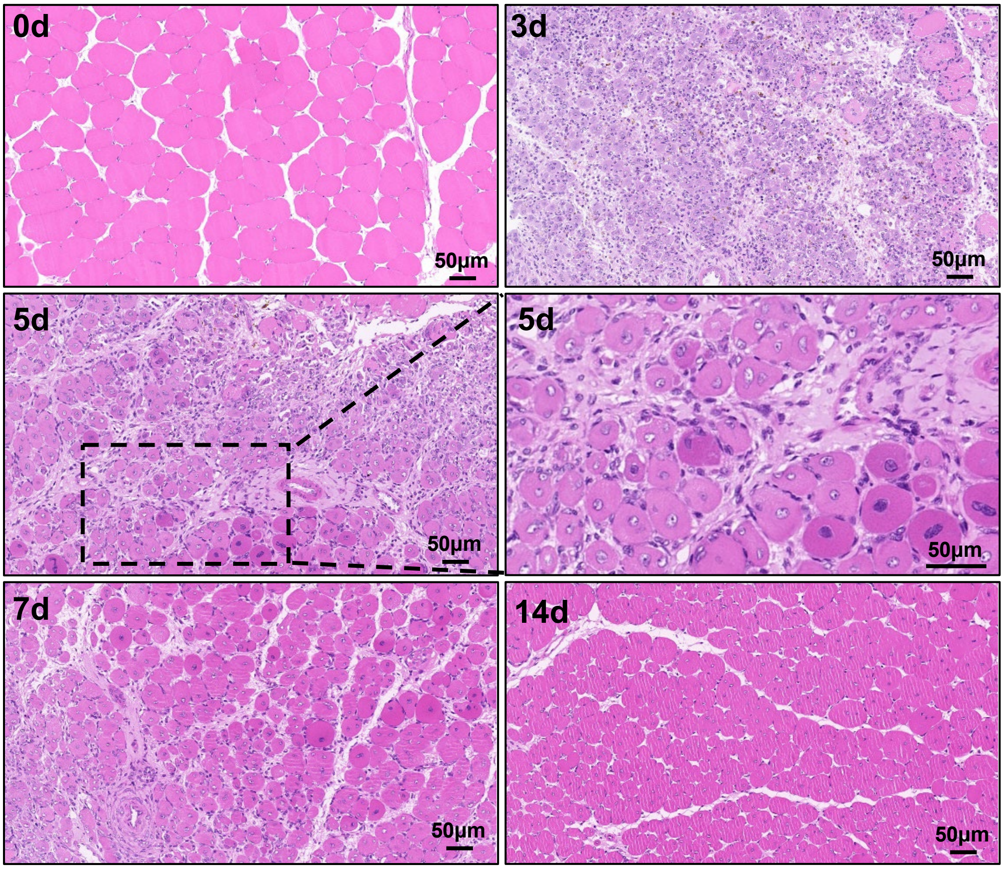

Appendix Figure S2. Skeletal muscle pathological phenotype of CTX-injured mice.

Representative H&E staining of TA muscle sections in CTX-injured mice at the indicated time points. Scale bar = 50μm.

Appendix Figure S3

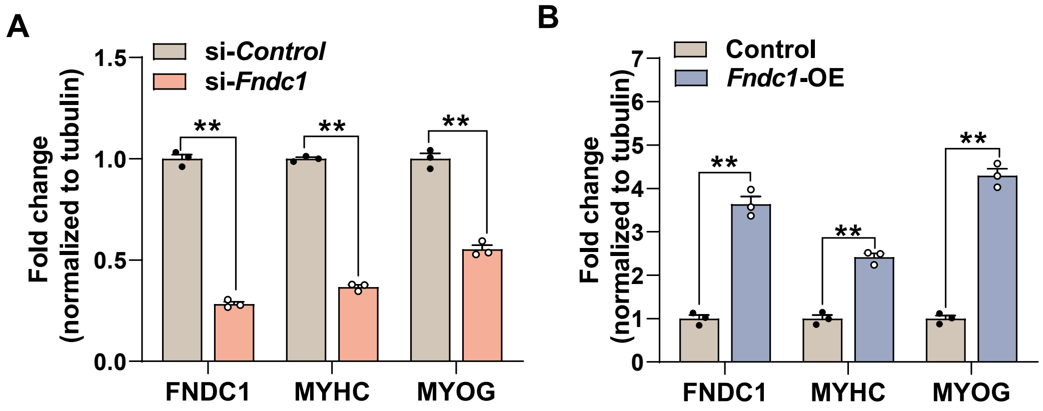

Appendix Figure S3. The efficiency of FNDC1 interference and overexpression in C2C12 cells

(A) *Fndc1* interference efficiency. Quantification of FNDC1, MYHC and MYOG immunoblot (n = 3 independent experiments). **(B)** *Fndc1* overexpression efficiency. Quantification of FNDC1, MYHC and MYOG immunoblot (n = 3 independent experiments). Two-tailed t-test was performed to compare all listed conditions unless otherwise noted, and data are represented as mean ± SEM. $^{**}p < 0.01$. Source data are provided as a Source data file.

Appendix Figure S4

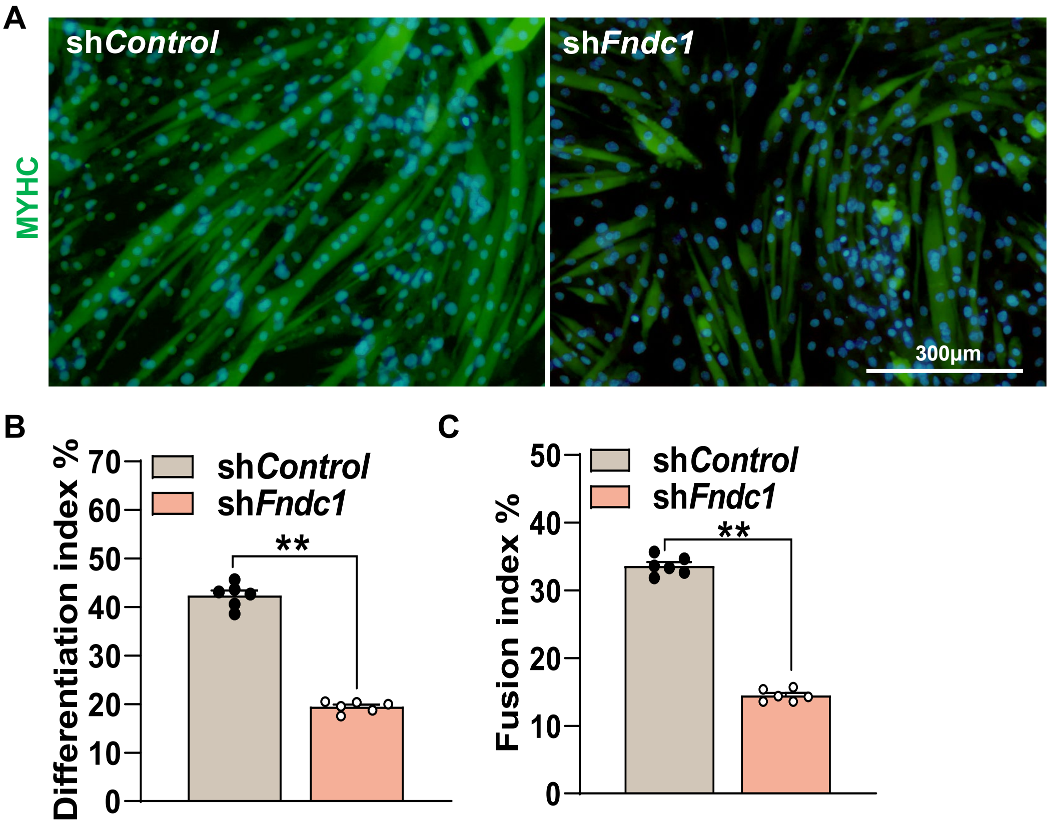

Appendix Figure S4. Fndc1 knockdown interferes with myoblast differentiation

(A) Representative immunofluorescence staining of MYHC (in green) in shControl or sh*Fndc1* primary myoblasts at day 3 post-differentiation. Scale bars = 300µm. **(B)** Quantification of differentiation index. **(C)** Quantification of fusion index (n = 6 independent experiments). Two-tailed t-test was performed to compare all listed conditions unless otherwise noted, and data are represented as mean ± SEM. **p < 0.01. Source data are provided as a Source data file.

Appendix Figure S5

SignalP forecast results:

XP_006523415.1_fibronectin_type_III_domain-containing_protein_1_isoform_X4__Mus
Prediction: Signal Peptide (Sec/SPI)
Cleavage site between pos. 29 and 30. Probability 0.987102

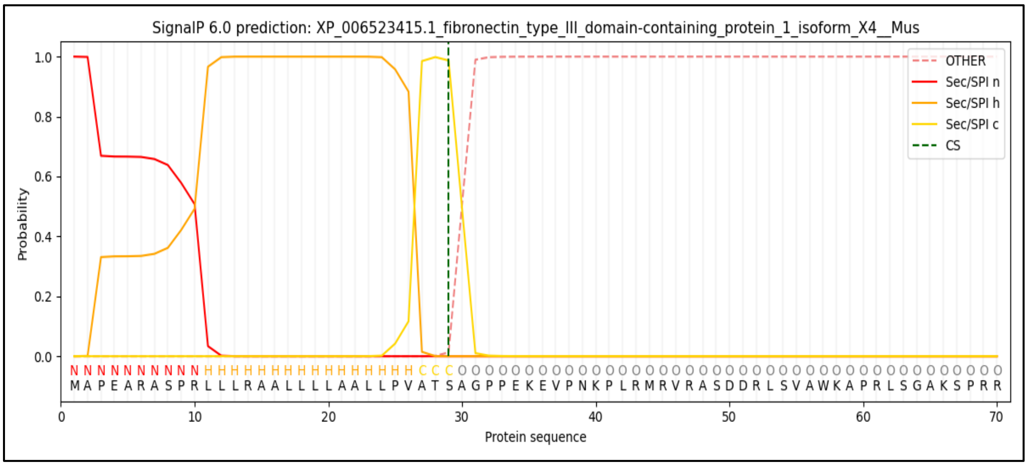

DeepTMHMM forecast results:

```
##gff-version 3
# Unnamed Length: 1716
# Unnamed Number of predicted TMRs: 0
Unnamed signal   1    29
Unnamed outside 30   1716
```

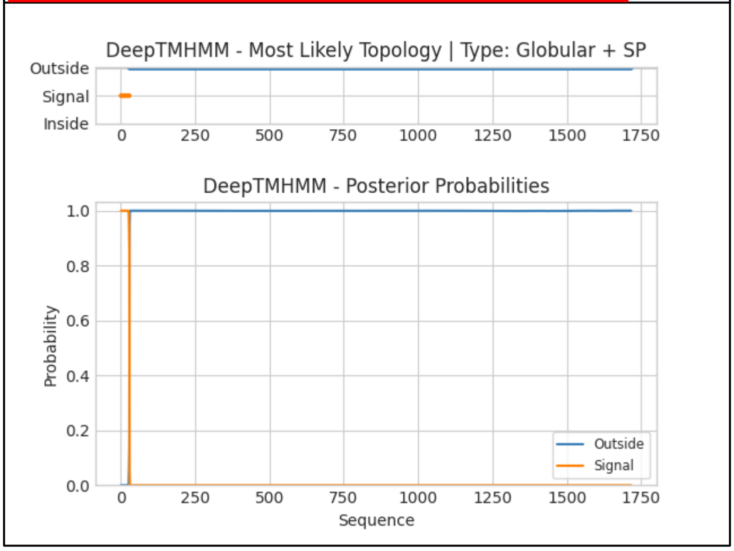

Appendix Figure S5. FNDC1 structure prediction

Prediction of FNDC1 signal peptide and transmembrane structure by SignalP (https://services.healthtech.dtu.dk/service.php?SignalP-6.0) and DeepTMHMM I (https://dtu.biolib.com/DeepTMHMM/).

Appendix Figure S6

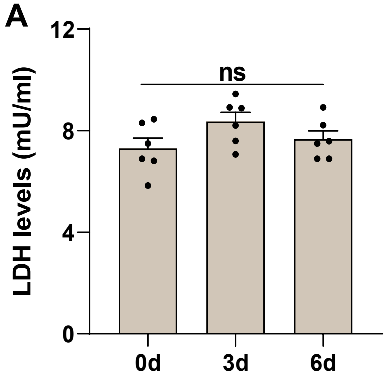

Appendix Figure S6. The LDH levels during differentiation of C2C12 cells.

(A) Medium LDH levels in cultured C2C12 cells at days 0, 3 or 6 post-differentiation (n = 6 independent experiments). Two-tailed t-test was performed to compare all listed conditions, and data are represented as mean ± SEM.

Appendix Figure S7

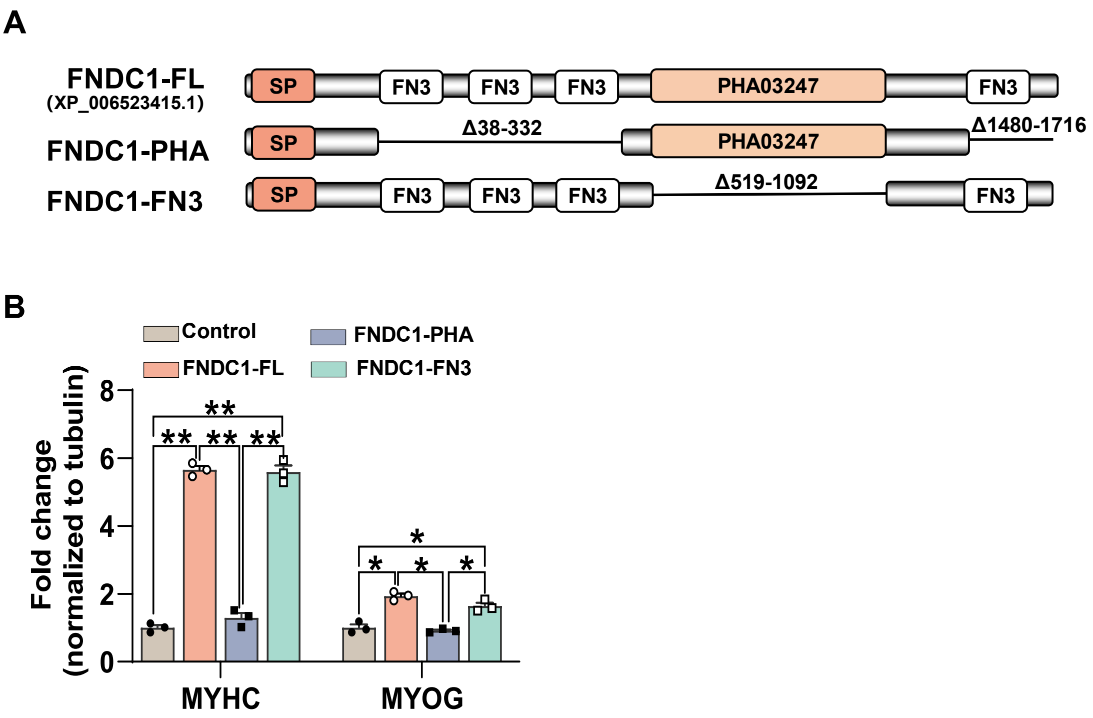

Appendix Figure S7. FN3 domain is essential for the myogenic function of FNDC1

(A) Schematic diagram of FNDC1 domains. Deletion mutants used in this study are shown. SP, signal peptide. **(B)** Quantification of MYHC and MYOG in cells transfected with Control (empty vector), FNDC1-FL, FNDC1-FN3 or FNDC1-PHA (n = 3 independent experiments). Cells were collected at day 4 post-differentiation. One-way ANOVA was performed to compare all listed conditions unless otherwise noted, and data are represented as mean ± SEM. *$p < 0.05$ and **$p < 0.01$. Source data are provided as a Source data file.

Appendix Figure S8

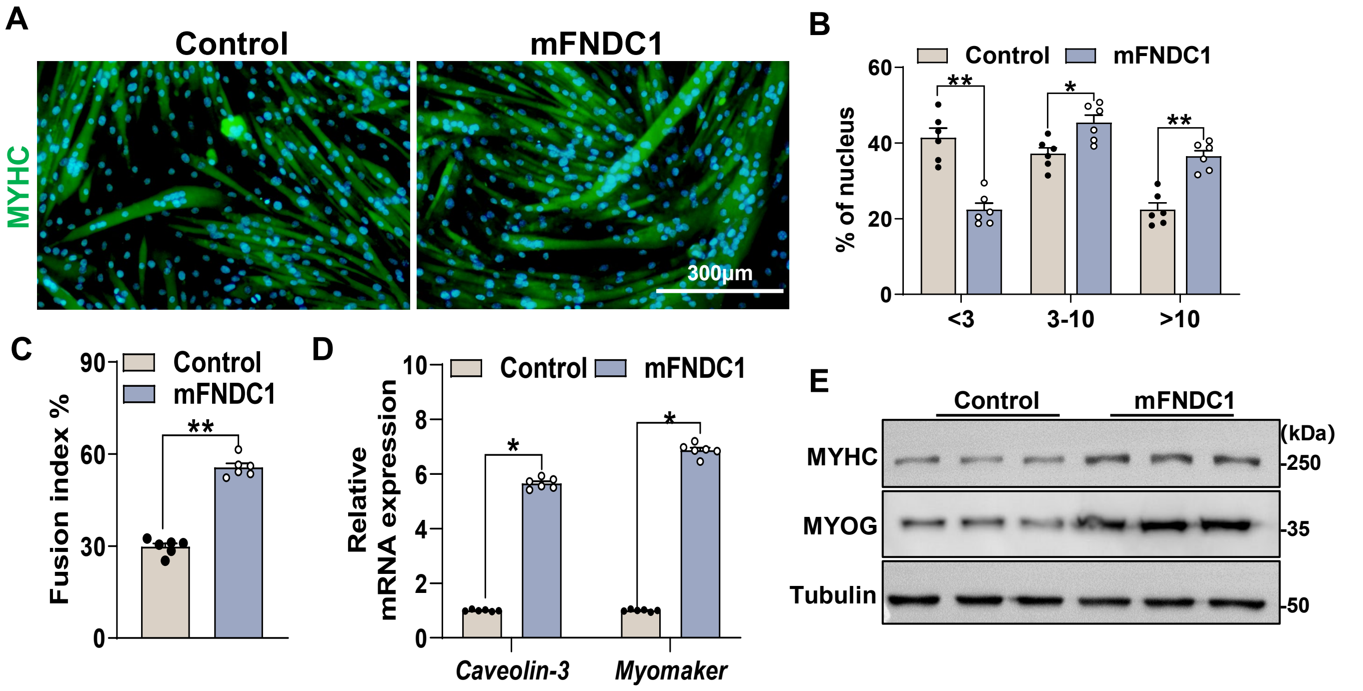

Appendix Figure S8. Identification of the purified recombinant FNDC1 protein

(A) Representative immunofluorescence staining of MYHC in Control (dialysis buffer, DB) or mFNDC1 (truncated recombinant FNDC1 protein, 0.1μg/ml)-treated C2C12 cells at day 4 post-differentiation. Staining for MYHC (in green) marks differentiated cells. Scale bar = 300μm. **(B-C)** Quantification of the nucleus distribution per myotube and fusion index (a MYHC^{+} cell with at least three nucleus) (n = 6 independent experiments). **(D)** mRNA expression of *Caveolin-3* and *Myomaker* in Control or mFNDC1-treated C2C12 cells at day 4 post-differentiation (n = 6 independent experiments). **(E)** Representative immunoblot analysis of FNDC1 and MYHC in Control or mFNDC1-treated C2C12 cells (n = 3 independent experiments). Two-tailed t-test was performed to compare all listed conditions unless otherwise noted, and data are represented as mean ± SEM. *$p < 0.05$ and **$p < 0.01$. Source data are provided as a Source data file.

Appendix Figure S9

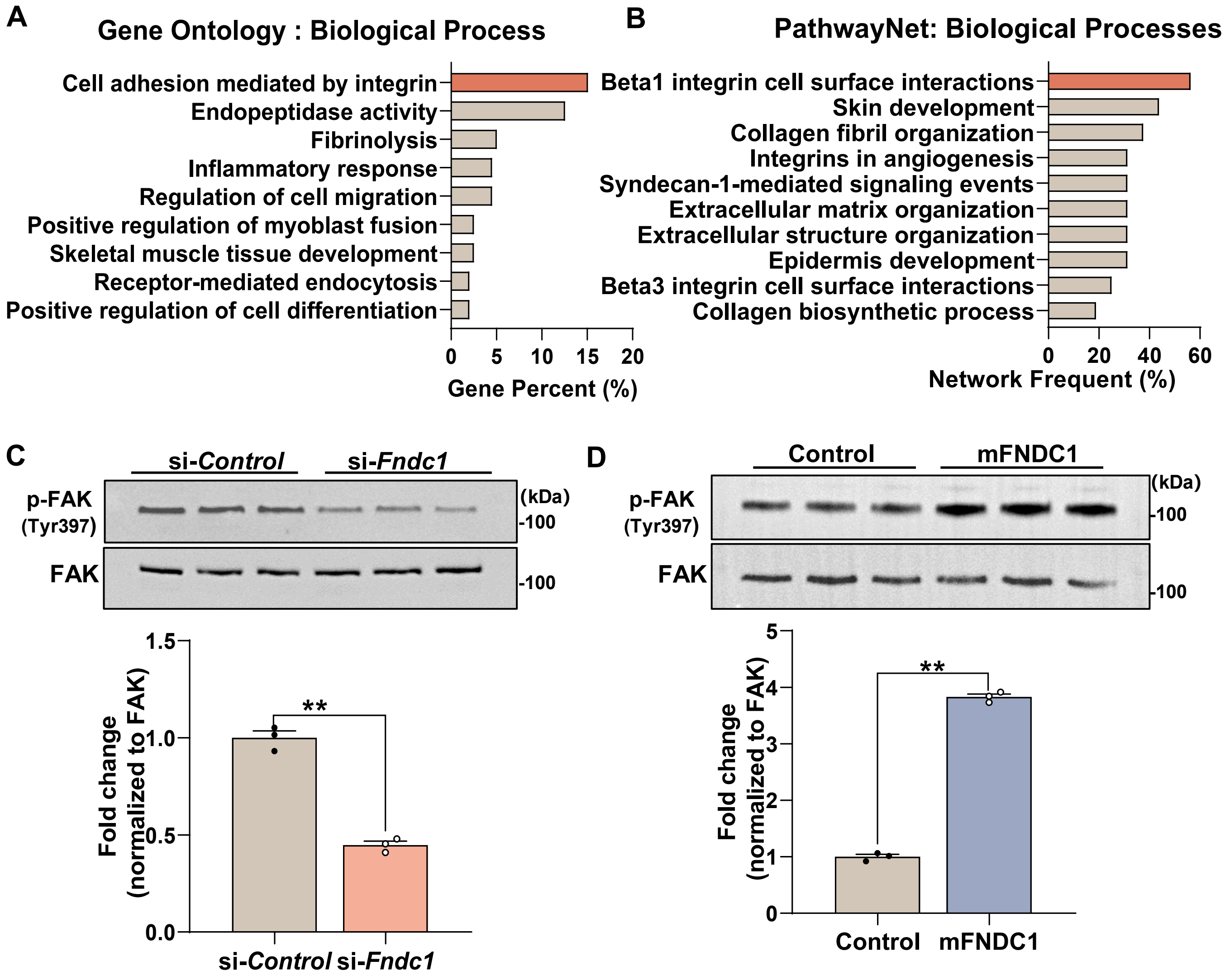

Appendix Figure S9. Integrin β1 is the receptor for FNDC1

(A) GO term analysis of DEGs ($p < 0.05$) in *Fndc1* knockdown and control C2C12 cells after 4 days of differentiation. **(B)** Enrichment analysis of potential co-complex partners of FNDC1 by PathwayNet. **(C-D)** Representative immunoblotting and quantification of focal adhesion kinase (FAK) and phosphorylated FAK in si-control, si-*Fndc1*, or mFNDC1-treated C2C12 cells (n = 3 independent experiments). Cells were collected at day 4 post-differentiation. Two-tailed t-test was performed to compare all listed conditions unless otherwise noted, and data are represented as mean ± SEM. **p < 0.01. Source data are provided as a Source data file.

Appendix Figure 10

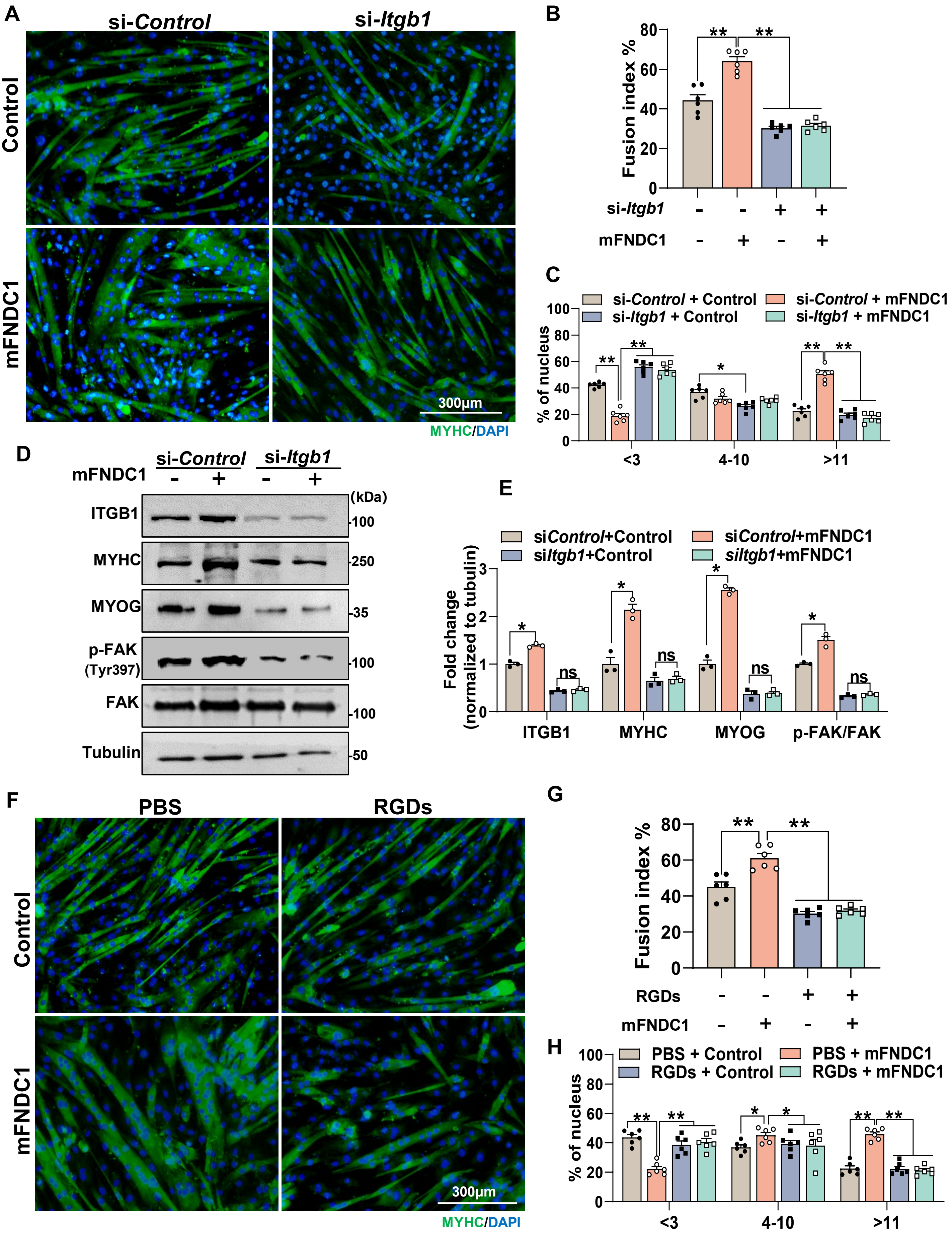

Appendix Figure S10. FNDC1 targeting integrin β1 promotes myogenic differentiation

(A) Representative immunofluorescence staining of MYHC (in green) in si-Control and si-*Itgb1* C2C12 cells treated with control (dialysis buffer) or mFNDC1 at day 4 post differentiation. Nuclei are counterstained with DAPI (in blue). C2C12 cells were transfected with si-Control or si-*Itgb1* for 12 hours before the initiation of differentiation. Scale bars = 300μm. **(B-C)** Quantification of the fusion index (a $MYHC^+$ cell with at least three nucleus) and nucleus distribution per myotube (n = 6 independent experiments). **(D-E)** Representative immunoblotting (D) and quantification (E) of indicated proteins in si-Control or si-*Itgb1* C2C12 cells treated with control or mFNDC1 at day 4 post differentiation (n = 3 independent experiments). **(F)** Representative immunofluorescence staining of MYHC (in green) in C2C12 cells from control, mFNDC1-treated, RGDs-treated, and mFNDC1+RGDs groups at day 4 post differentiation. Scale bars = 300μm. **(G-H)** Quantification of fusion index (a $MYHC^+$ cell with at least three nucleus) and nucleus distribution per myotube (n = 6 independent experiments). One-way ANOVA was performed to compare all listed conditions unless otherwise noted, and data are represented as mean ± SEM. *p < 0.05 and **p < 0.01. Source data are provided as a Source data file.

Appendix Figure S11

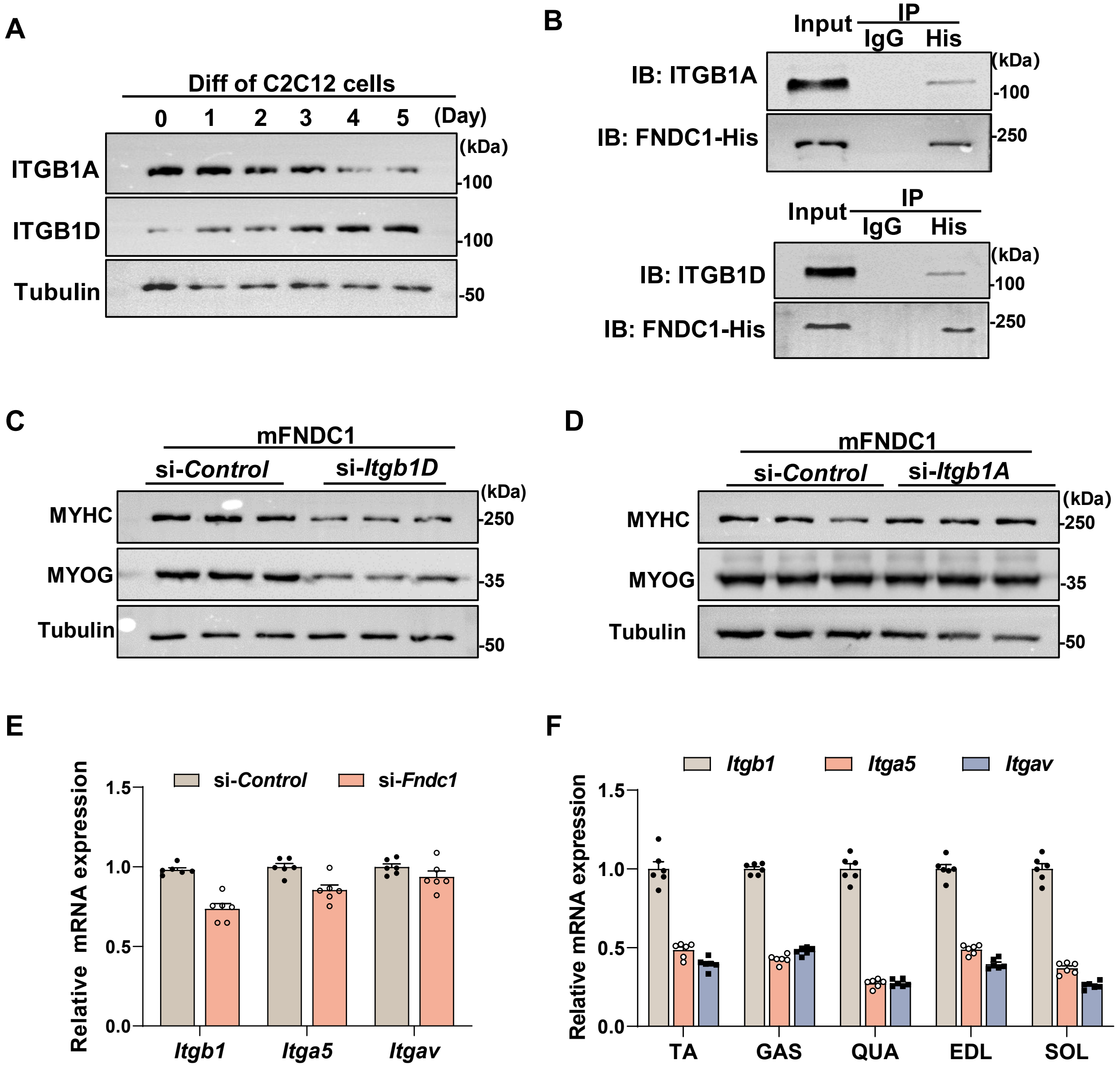

Appendix Figure S11. Integrin α5β1D is the receptor for FNDC1

(A) Representative immunoblotting of integrin β1A (ITGB1A) and integrin β1D (ITGB1D) during differentiation of C2C12 cells (n = 3 independent experiments). **(B)** Immunoprecipitation of FNDC1 and ITGB1A or ITGB1D in C2C12 cells at day 3 post differentiation. **(C-D)** Representative immunoblotting analysis of MYHC and MYOG in *Itgb1D* or *Itgb1A* knockdown C2C12 cells treated with mFNDC1 at day 3 post differentiation (n = 3 independent experiments). **(E)** mRNA expression of *Itgb1, Itga5,* and *Itgav* in si-control and si-*Fndc1* C2C12 cells (n = 6). **(F)** mRNA expression of *Itgb1, Itga5*, and *Itgav* in different depots of skeletal muscles from mice (n = 6 independent experiments). Two-tailed t-test (E and F) were performed to compare all listed conditions unless otherwise noted, and data are represented as mean ± SEM. Source data are provided as a Source data file.

Appendix Figure S12

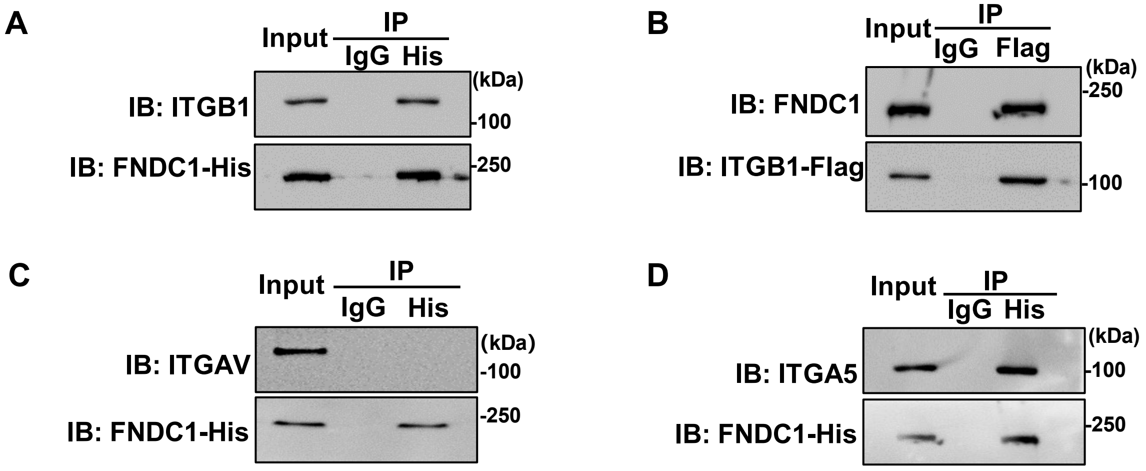

Appendix Figure S12. FNDC1 binds to ITGB1 and ITGA5 in primary myoblasts. (A-B) Co-IP of FNDC1 and ITGB1 in primary myoblasts. **(C-D)** Immunoprecipitation of FNDC1 and ITGAV or ITGA5 in primary myoblasts. Source data are provided as a Source data file.

Appendix Figure S13

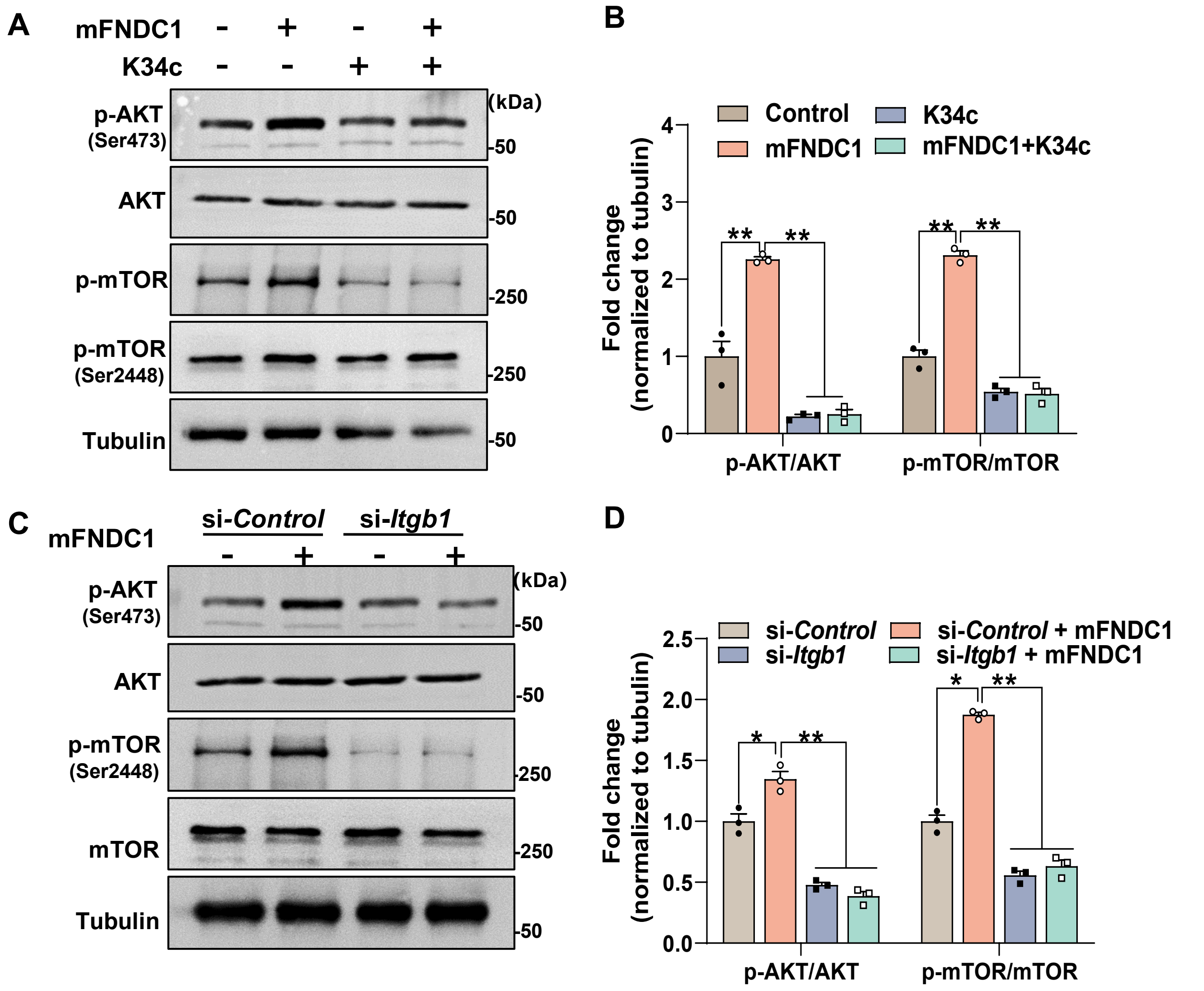

Appendix Figure S13. FNDC1 improves myogenesis through activating the AKT/mTOR pathway

(A-B) Representative immunoblotting (A) and quantification (B) of indicated proteins in control (PBS) or K34c treated C2C12 cells receiving Control or mFNDC1 (n = 3 independent experiments). **(C-D)** Representative immunoblotting (C) and quantification (D) of total and phosphorylated AKT and mTOR in si-Control or si-*Itgb1* C2C12 cells treated with Control or mFNDC1 at day 4 post differentiation (n = 3 independent experiments). One-way ANOVA was performed to compare all listed conditions unless otherwise noted, and data are represented as mean ± SEM. $^{*}p < 0.05$, $^{**}p < 0.01$, and $^{***}p < 0.001$. Source data are provided as a Source data file.

Appendix Figure S14

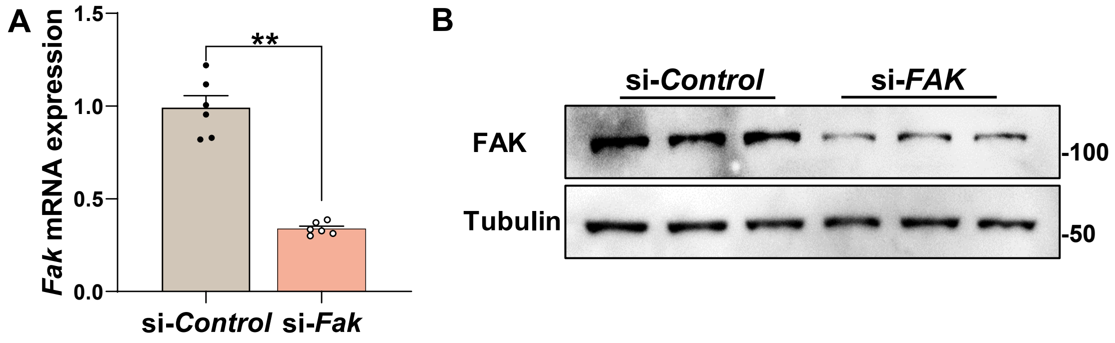

Appendix Figure S14. Efficiency of FAK knockdown in C2C12 cells

(A) mRNA expression of *Fak* in si-control and si-*Fak* C2C12 cells (n = 6 independent experiments). **(B)** Representative immunoblotting of FAK in si-control and si-*Fak* C2C12 cells (n = 3 independent experiments). Two-tailed t-test was performed to compare all listed conditions unless otherwise noted, and data are represented as mean ± SEM. $^{**}p$ < 0.01. Source data are provided as a Source data file.

Appendix Figure S15

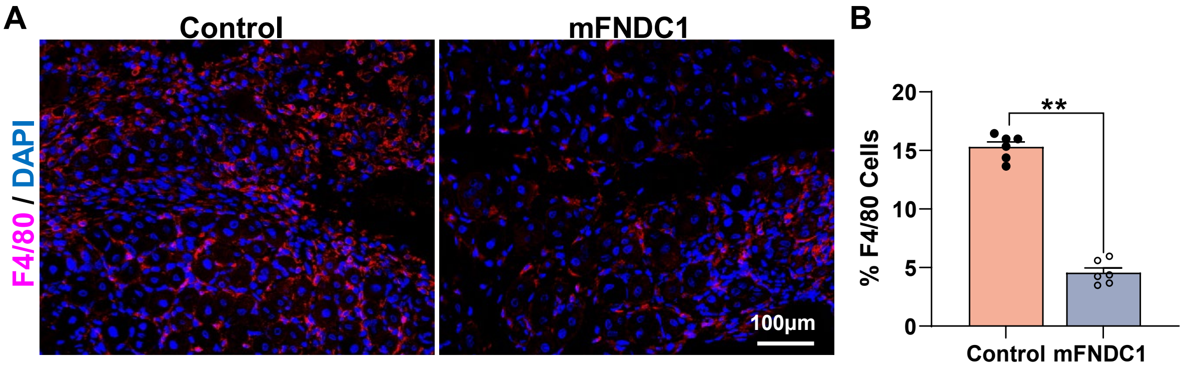

Appendix Figure S15. FNDC1 inhibits the inflammatory cell infiltration in skeletal muscle of CTX-injured mice

(A) Representative images of immunofluorescence staining and quantification **(B)** for F4/80-labeled macrophages (in red) from CTX-injured mice treated with mFNDC1 or control (n = 6 mice). Scale bars = 100μm. Two-tailed t-test was performed to compare all listed conditions unless otherwise noted, and data are represented as mean ± SEM. $^{**}p <$ 0.01. Source data are provided as a Source data file.

Appendix Figure S16

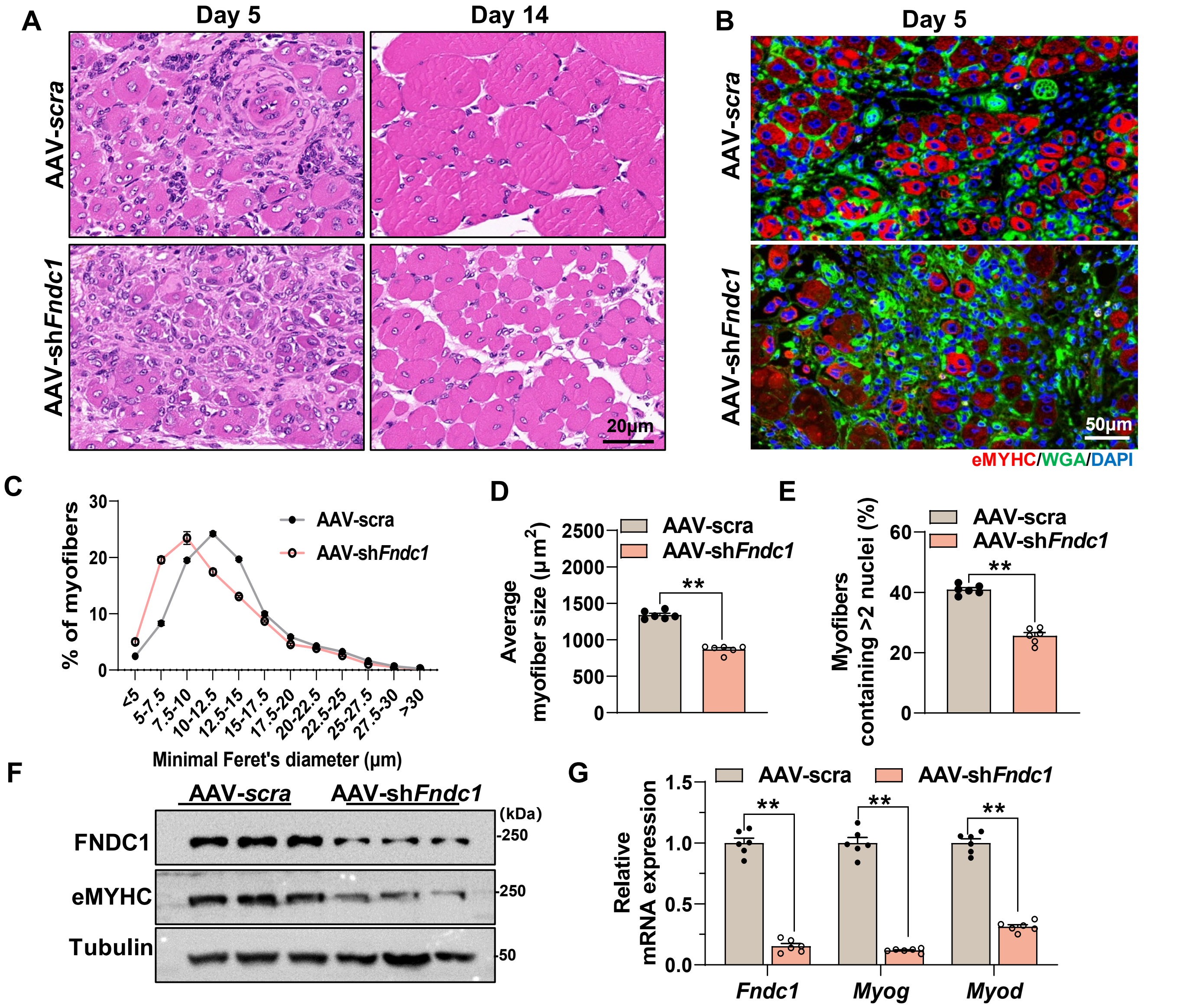

Appendix Figure S16. *Fndc1* knockdown delayed skeletal muscle regeneration

(A) Representative H&E staining of TA muscle sections at days 5 and 14 post-injury in AAV-*Scra* or AAV-sh*Fndc1* mice (n = 6 mice). Scale bars = 20μm. **(B)** Representative immunofluorescence staining of eMYHC$^+$ fibers in AAV-*Scra* or AAV-sh*Fndc1* TA muscle at day 5 post-injury (n = 6 mice). Scale bars = 50 μm. **(C-D)** Distribution of eMYHC+ minimal Feret's diameter and average CSA in AAV-*Scra* or AAV-sh*Fndc1* TA muscle at day 5 post-injury (n = 6 mice). **(E)** Percentage of newly formed myofibers containing two or more central nuclei in muscle fibers at day 5 post-injury (n = 6 mice). **(F)** Representative immunoblotting analysis of FNDC1 and eMYHC in TA muscle with AAV-*Scra* or AAV-sh*Fndc1* at day 5 post-injury (n = 3 independent experiments). **(G)** mRNA expression of *Fndc1*, *Myog,* and *Myod* at day 5 post-injury (n = 6 independent experiments). Two-tailed t-test was performed to compare all listed conditions unless otherwise noted, and data are represented as mean ± SEM. $^{**}p < 0.01$. Source data are provided as a Source data file.

Appendix Figure S17

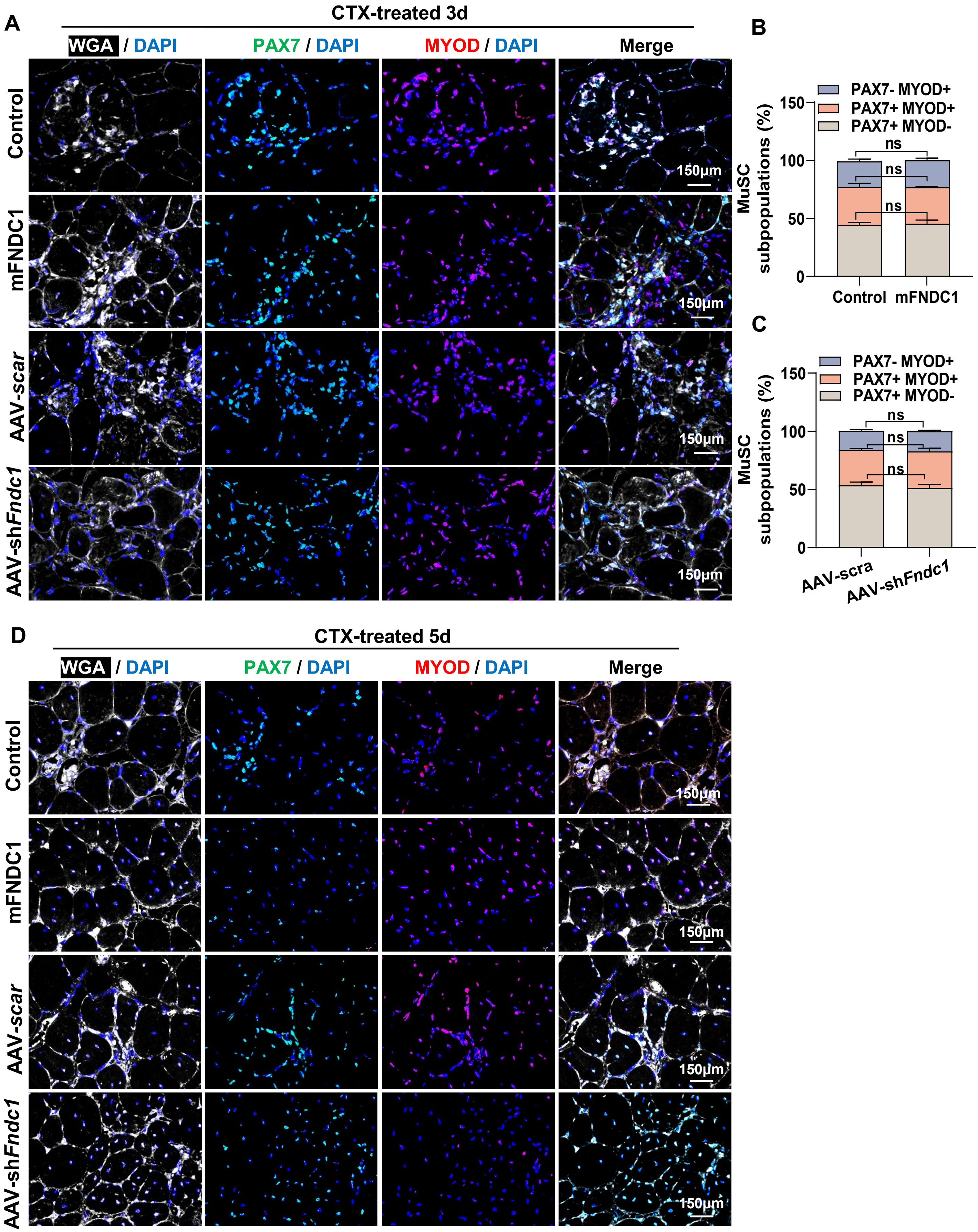

Appendix Figure S17. FNDC1 promotes satellite cell differentiation during skeletal muscle regeneration

(A) Immunofluorescence staining of TA muscle for WGA (in white), PAX7 (in green) and MYOD

(in red) in CTX-injured mice receiving mFNDC1 or AAV-sh*Fndc1* at day 3 post-injury (n = 6 mice). Nuclei were counterstained with DAPI (in blue). Scale bar = 150μm. **(B-C)** Quantification of different states of satellite cell (MuSC) in TA muscle from mice in Figure (A) (n = 6 mice). **(D)** Immunofluorescence staining of TA for WGA (in white), PAX7 (in green) and MYOD (in red) in CTX-injured mice receiving mFNDC1 or AAV-sh*Fndc1* at day 5 post-injury (n = 6 mice). Scale bar = 150μm. Two-tailed t-test was performed to compare all listed conditions unless otherwise noted, and data are represented as mean ± SEM. Source data are provided as a Source data file.

Appendix Figure S18

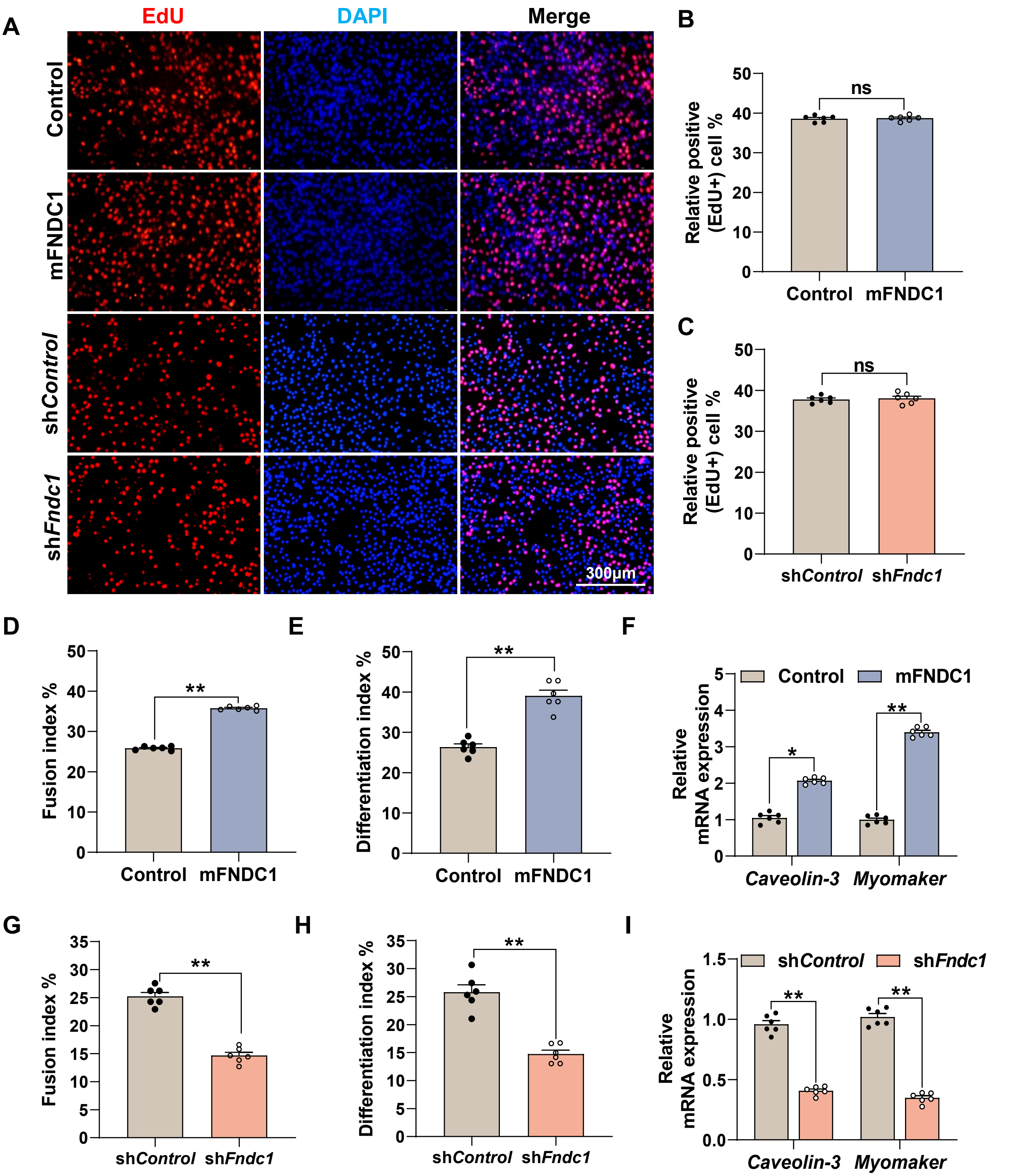

Appendix Figure S18. FNDC1 promotes satellite cell-derived primary myoblasts differentiation during skeletal muscle regeneration *in vitro*

(A-C) Representative photographs of satellite cell-derived primary myoblasts treated with control, mFNDC1, shControl, or sh*Fndc1* for the detection of 5-ethynyl-2-deoxyuridine (EdU) staining and the quantification of EdU-positive cells (n = 6 independent experiments). Satellite cell were isolated by FACS from WT mice and were treated with recombinant proteins (control or mFNDC1) or lentivirus (shControl or sh*Fndc1*) for 24 h, followed by EdU treatment for 4h to detect cell proliferation. Scale bars = 300 μm. **(D-E)** Quantification of fusion index and differentiation index in satellite cell-derived primary myoblasts treated with Control or mFNDC1 at day 3 post-differentiation. **(F)** mRNA expression of *Caveolin-3* and *Myomaker* in primary myoblasts

treated with Control or mFNDC1 at day 3 post-differentiation (n = 6 independent experiments). **(G-H)** Quantification of fusion index and differentiation index in primary myoblasts treated with shControl or sh*Fndc1* at day 3 post-differentiation. **(I)** mRNA expression of *Caveolin-3* and *Myomaker* in primary myoblasts treated with shControl or sh*Fndc1* at day 3 post-differentiation (n = 6 independent experiments). Two-tailed t-test was performed to compare all listed conditions unless otherwise noted, and data are represented as mean ± SEM. $^{*}p < 0.05$ and $^{**}p < 0.01$. Source data are provided as a Source data file.

Appendix Figure S19

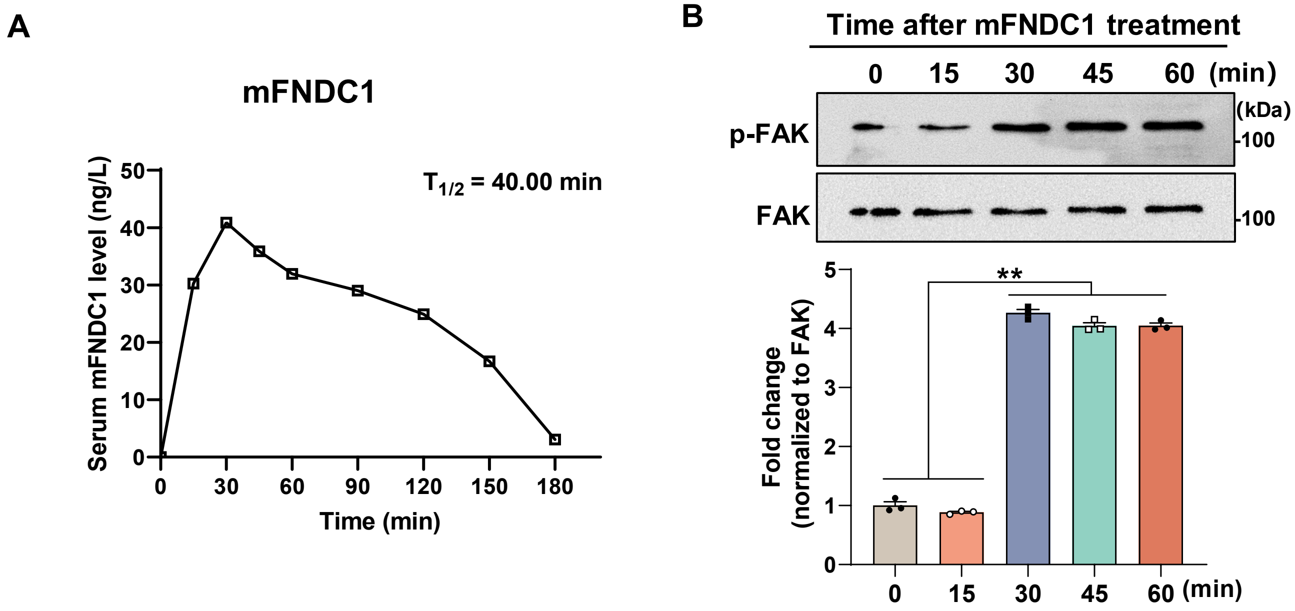

Appendix Figure S19. Pharmacokinetics and pharmacodynamics of full length and truncated FNDC1 in mice

(A) Serum concentration-time profiles in C57BL/6J mice following single *i.p.* injection of 2.5 mg/kg body weight of mFNDC1 (n = 12 mice). **(B)** Representative Western blots and quantification of p-FAK in TA muscle from 8-week C57BL/6J mice after *i.p.* injection of 2.5 mg/kg recombinant mFNDC1 (n = 3 independent experiments). One-way ANOVA was performed to compare all listed conditions unless otherwise noted, and data are represented as mean ± SEM. $^{**}p < 0.01$. Source data are provided as a Source data file.

Appendix Figure S20

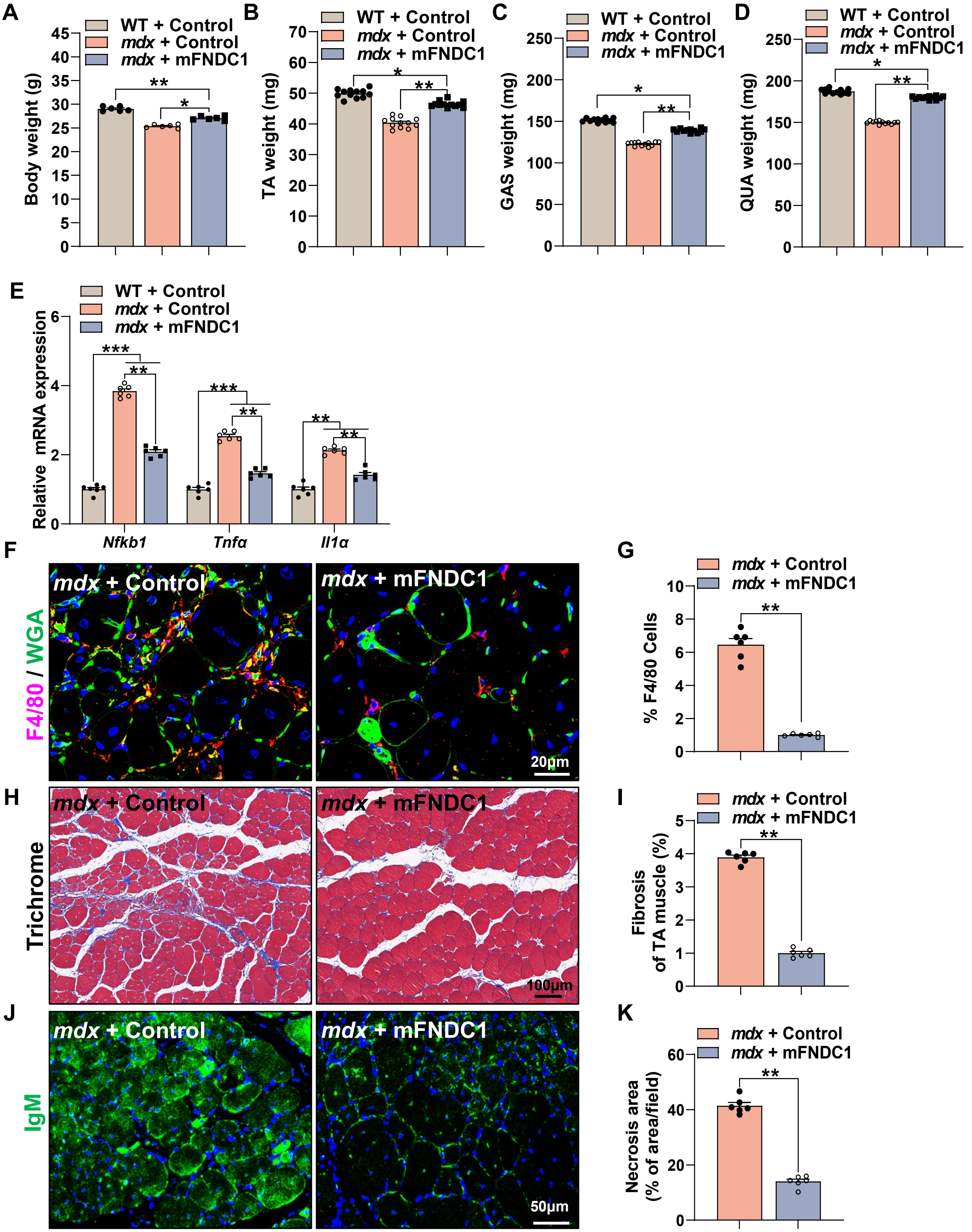

Appendix Figure S20. Effects of FNDC1 on muscle weight and pathology indices in *mdx* mice

(A) Body weight of WT and *mdx* mice treated with or without mFNDC1 for 4 weeks (n = 12 mice). **(B-D)** Weights of TA, GAS and QUA muscles from WT and *mdx* mice treated with or without mFNDC1 (n = 12 mice). **(E)** mRNA expression of inflammatory marker genes *Nfkb1*,

Tnfα, and *Il1α* (n = 6 mice). **(F-G)** Representative images of immunofluorescence staining for F4/80-labeled macrophages (in red) (F) and quantification (G) from WT and *mdx* mice treated with mFNDC1 or control for 4 weeks (n = 6 mice). Scale bars = 20μm. **(H-I)** Representative Masson's Trichrome staining (H) and fibrosis quantification (I) of TA muscle from *mdx* mice treated with control or mFNDC1 for 4 weeks (n = 6 mice). Scale bars = 100μm. **(J-K)** Representative immunofluorescence staining for IgM (in green) of fibers (J) and quantification of the area of muscle fiber necrosis (K) in TA from *mdx* mice treated with control or mFNDC1 for 4 weeks (n = 6 mice). Scale bars = 50μm. One-way ANOVA (A, B, C, D, and E) or two-tailed t-test (G, I, and K) was performed to compare all listed conditions unless otherwise noted, and data are represented as mean ± SEM. $^{**}p < 0.01$, and $^{***}p < 0.001$. Source data are provided as a Source data file.

Appendix Figure S21

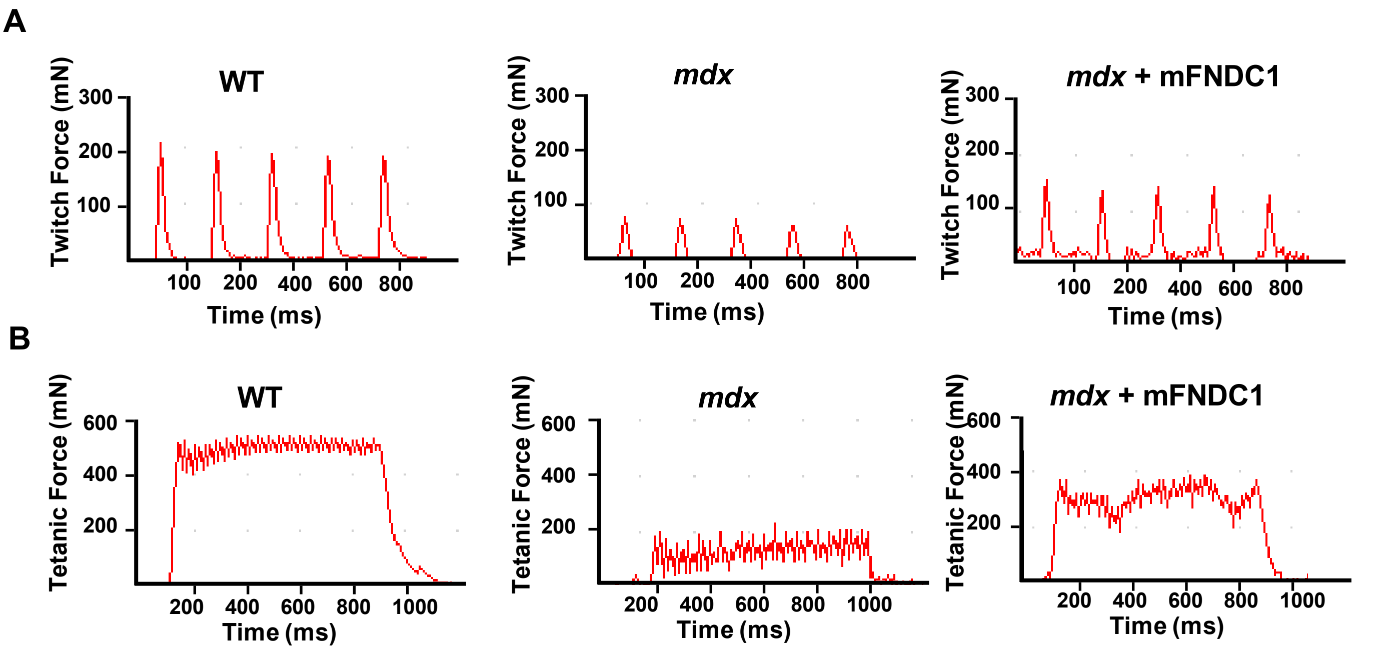

Appendix Figure S21. Effect of FNDC1 on twitch force and tetanic force of TA muscle in *mdx* mice

(A-B) Representative traces of twitch force and tetanic force of TA muscle (n = 12 mice).

Appendix Table S1. LC-MS analysis of recombinant FNDC1 protein

Accession	Sum PEP Score	Peptides	# PSMs	# Unique Peptides	calc. pI	Sequest HT	Gene name
E9Q043	44.345	20	133	20	9.42	475.47	Fndc1
P07724	4.903	2	10	2	6.07	29.86	ALB
P04104	3.597	2	2	1	8.15	8.81	KRT1
A8DUK4	3.356	2	2	2	7.69	7.18	HBB-BS
P02535	2.916	2	2	2	5.11	7.05	KRT10
Q6NXH9	2.853	2	2	1	8.09	7.55	KRT73
P01787	1.697	1	1	1	6.62	3.74	Ig heavy chain V
Q61781	1.693	1	1	1	5.17	3.41	KRT14
P01942	1.59	2	2	2	8.22	5.7	HBA
Q3TTY5	1.433	1	1	1	8.06	3.34	KRT2
Q922U2	1.356	1	1	1	7.75	3.04	KRT5
Q9WTP6	1.153	1	1	1	7.39	2.6	AK2
Q8VED5	1.064	1	1	1	7.69	3.37	KRT79
E9Q0F0	0.965	1	1	1	7.97	3.61	KRT78
Q9Z0J7	0.934	1	1	1	9.11	2.99	GDF15
Q61171	0.885	1	1	1	5.41	2.4	PRDX2
G3UZF7	0.828	1	1	1	8.44	0	D7ERTD443E
P27661	0.787	1	1	1	10.74	2.35	H2AFX
Q6IFX2	0.777	1	1	1	5.16	2.72	KRT42
Q9Z2K1	0.672	1	1	1	5.2	2.5	KRT16

Appendix Table S2. Pharmacokinetic parameters of intraperitoneal injection of mFNDC1 in mice

	Dose (mg/kg)	Route	C_{max} (mg/kg)	T_{max} (min)	AUC_{all} (min*mg/kg)	AUC_{inf} (min*mg/kg)
**mFNDC1**	2.5	*i.p.*	40.86±1.56	30	2862.25±8.36	2945.23±4.59

Appendix Table S3. Mass spectrometry results

Accession	Coverage [%]	# Peptides	# PSMs	# Unique Peptides	Sequest HT	Gene name
Q4ZHG4.4	8	6	6	6	35.25	FNDC1
P09055.1	6	4	4	4	24.36	ITGB1
P09813.2	3	3	3	2	14.36	APOA1
Q920R6.1	7	3	3	2	10.35	ATP6V0C
P01027.3	5	3	3	1	10.6	C3
P23927.2	5	4	3	1	8.32	CRYAB
Q9D952.3	5	3	3	2	5.36	EVPL
E9PV24.1	4	2	2	1	3.23	FGA
Q8K0E8.1	4	3	3	1	4.36	FGB
Q8VCM7.1	3	2	1	1	5.32	FGG
P68871.2	2	2	1	1	3.26	HBB
Q61646.1	2	1	1	1	2.31	HP
P01868.1	4	2	1	1	2.35	IGHG1
P01837.2	2	1	1	1	2.36	IGKC
P63328.1	3	1	1	1	4.32	IVL
Q60590.1	4	1	1	1	6.32	LGALS7B
Q8K4L4.3	3	2	1	1	3.25	ORM1
Q9R269.1	8	2	1	1	5.36	POF1B
P26595.1	3	1	1	1	2.83	AAT
P70124.1	2	1	1	1	3.68	SERPINB5
Q921I1.1	3	1	1	1	15.32	TRF
Q9ESL8.2	2	1	1	1	14.3	FGF16
Q66K08.1	5	2	1	1	10.5	CILP1
Q8BV57.1	6	3	1	1	8.36	SSC5D
P37889.2	4	2	1	1	7.25	FBLN2
Q61554.2	6	3	1	1	3.25	FBLN1
Q9QZJ6.1	2	1	1	1	3.66	MFAP5
O08999.2	4	2	1	1	1.3	LTBP2
Q9QZZ6.1	2	1	1	1	5.36	DPT
P61622.1	3	2	1	1	8.691	ITGBA11
Q3TYX2.1	2	1	1	1	7.25	LRRN4CL
Q9R045.2	2	1	1	1	4.23	ANGPTL2
Q6PE55.1	2	1	1	1	2.01	PDGFRL
Q9ET66.2	2	1	1	1	1.25	PI16
Q99MQ4.1	2	1	1	1	1.58	ASPN
Q8CD91.1	2	1	1	1	2.98	SMOC2
