# Supplementary figures and images for: FNDC1 is a myokine that promotes myogenesis and muscle regeneration (part 1 of 2)
Source: EMBO J. 2024 Nov 20;44(1):30–53. doi: 10.1038/s44318-024-00285-0 (PMC11695938; doi:10.1038/s44318-024-00285-0)

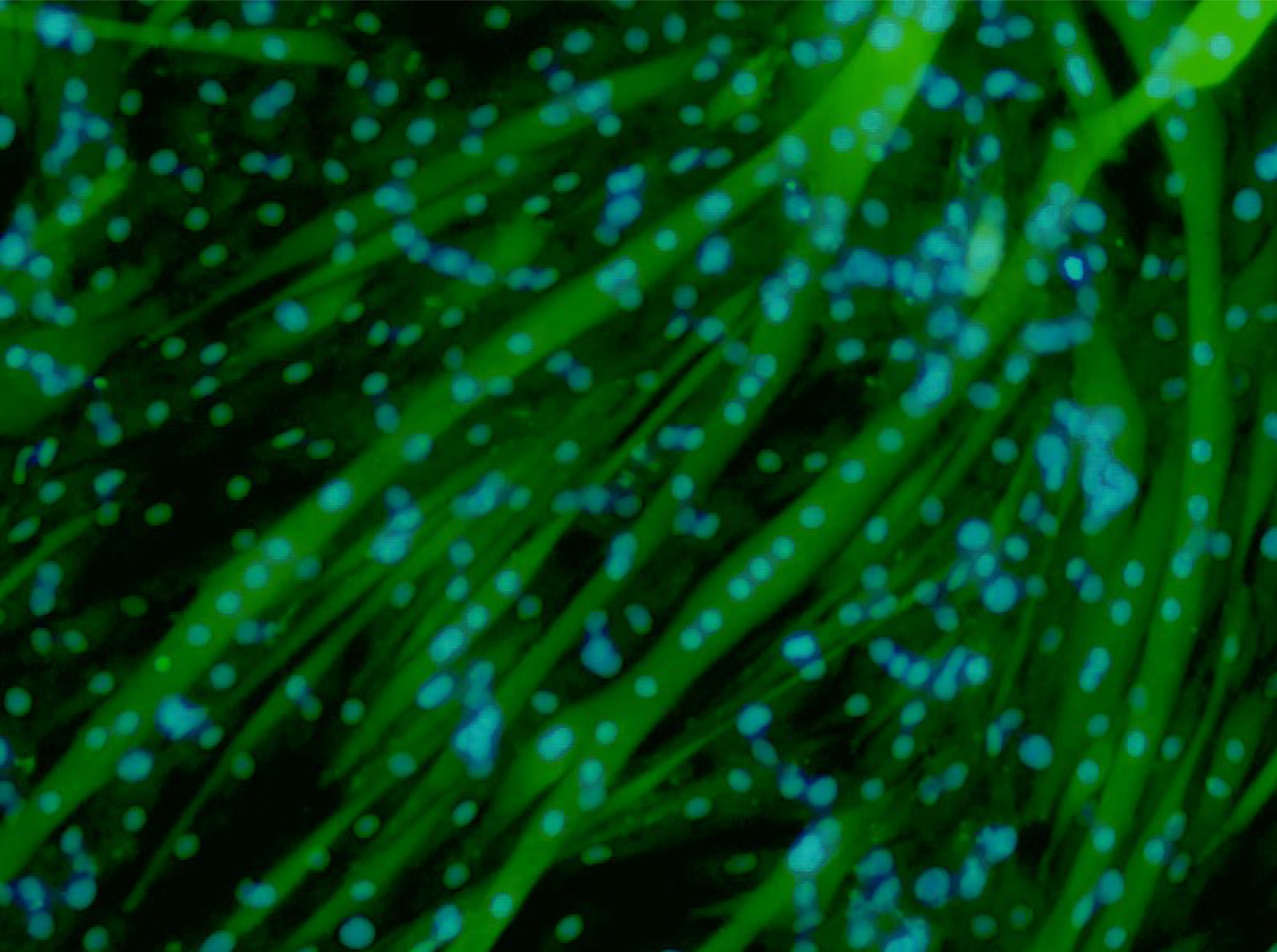

Supplement: Supplementary file 3 — Appendix Figure Source Data [file 44318_2024_285_MOESM3_ESM.zip › Appendix Figure S4/SF 4A/SF4-A-shControl.tif]

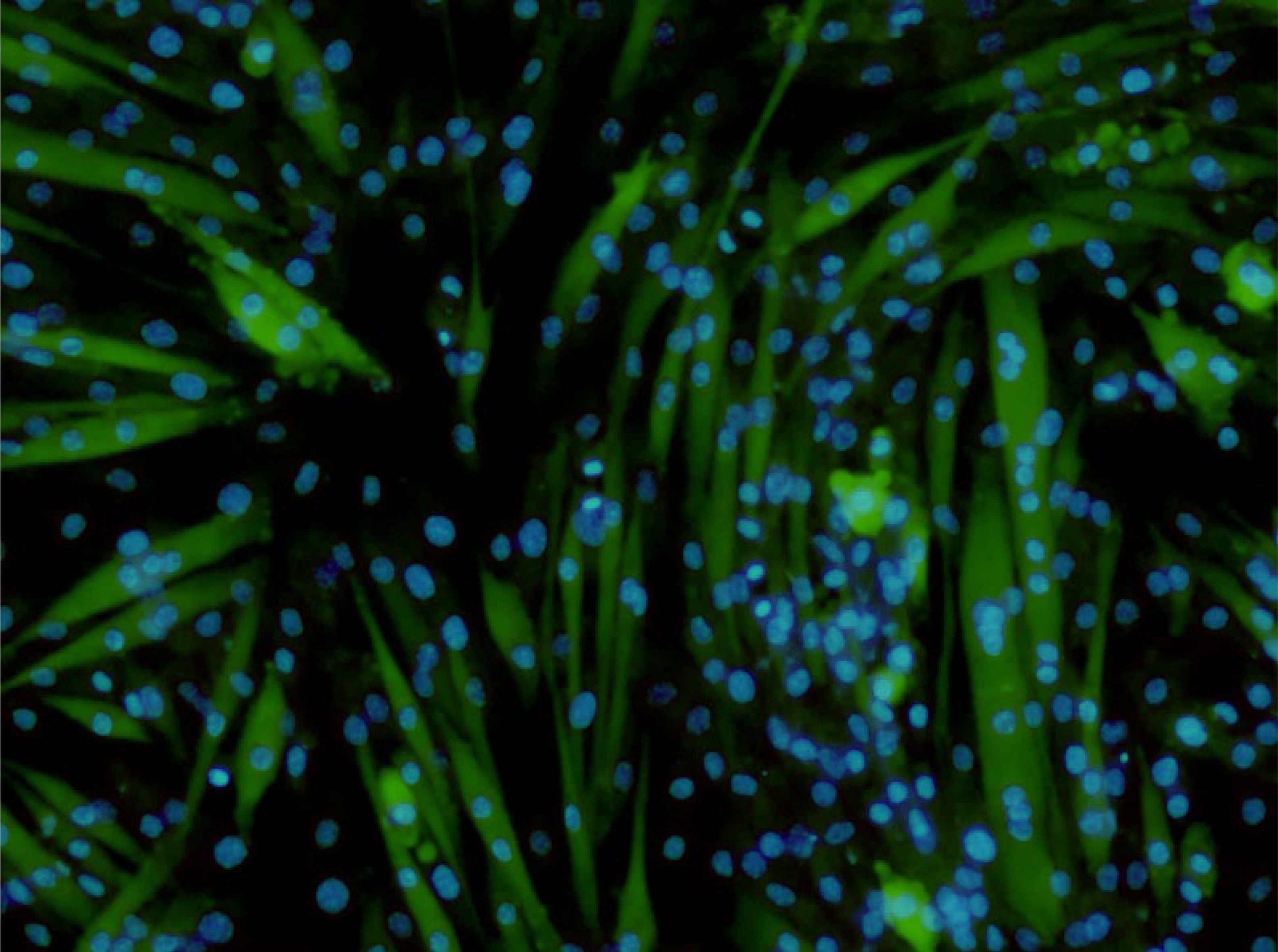

Supplement: Supplementary file 3 — Appendix Figure Source Data [file 44318_2024_285_MOESM3_ESM.zip › Appendix Figure S4/SF 4A/SF4-A-shFndc1.tif]

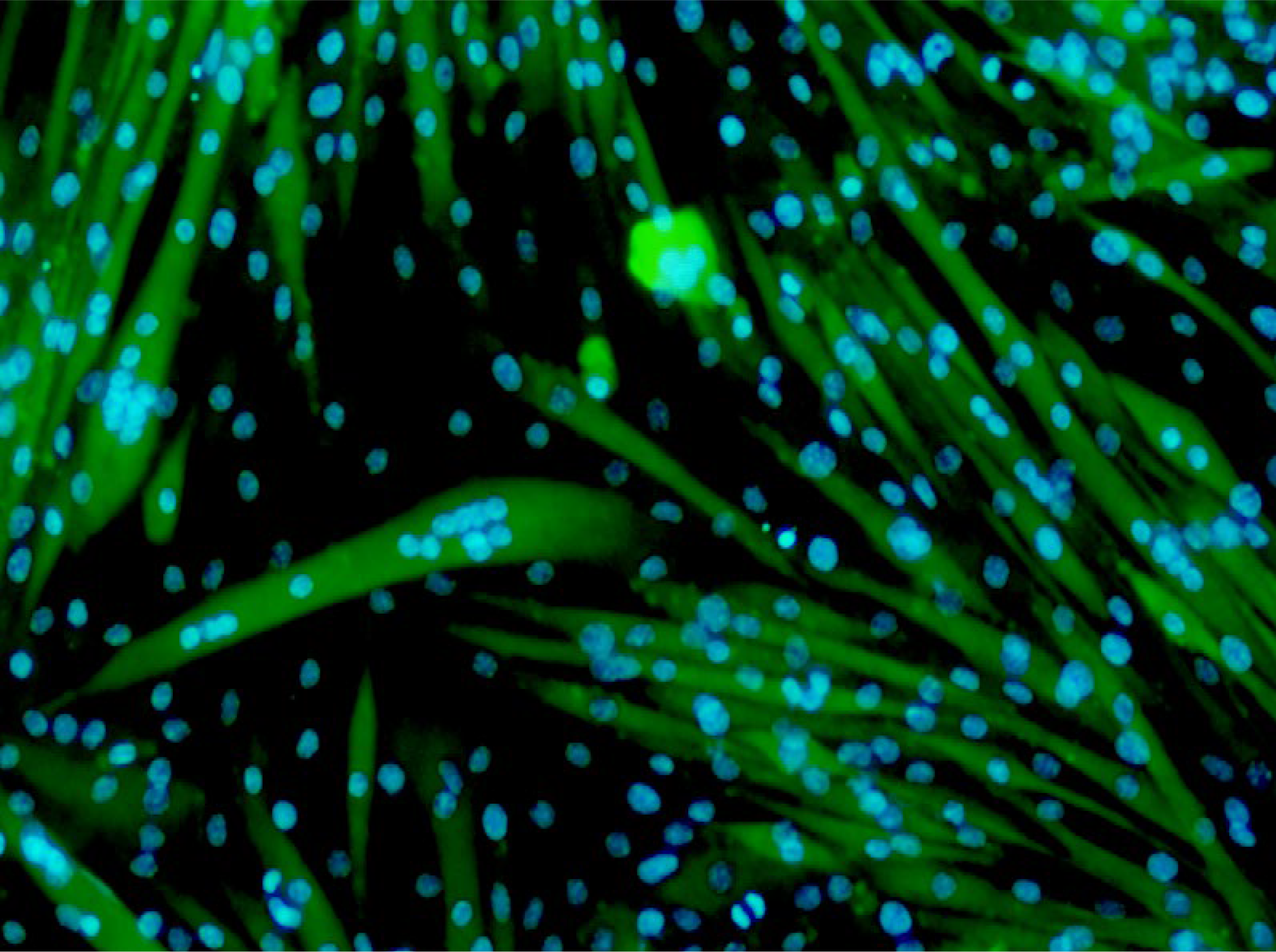

Supplement: Supplementary file 3 — Appendix Figure Source Data [file 44318_2024_285_MOESM3_ESM.zip › Appendix Figure S8/SF 8A/SF8-A-Control.tif]

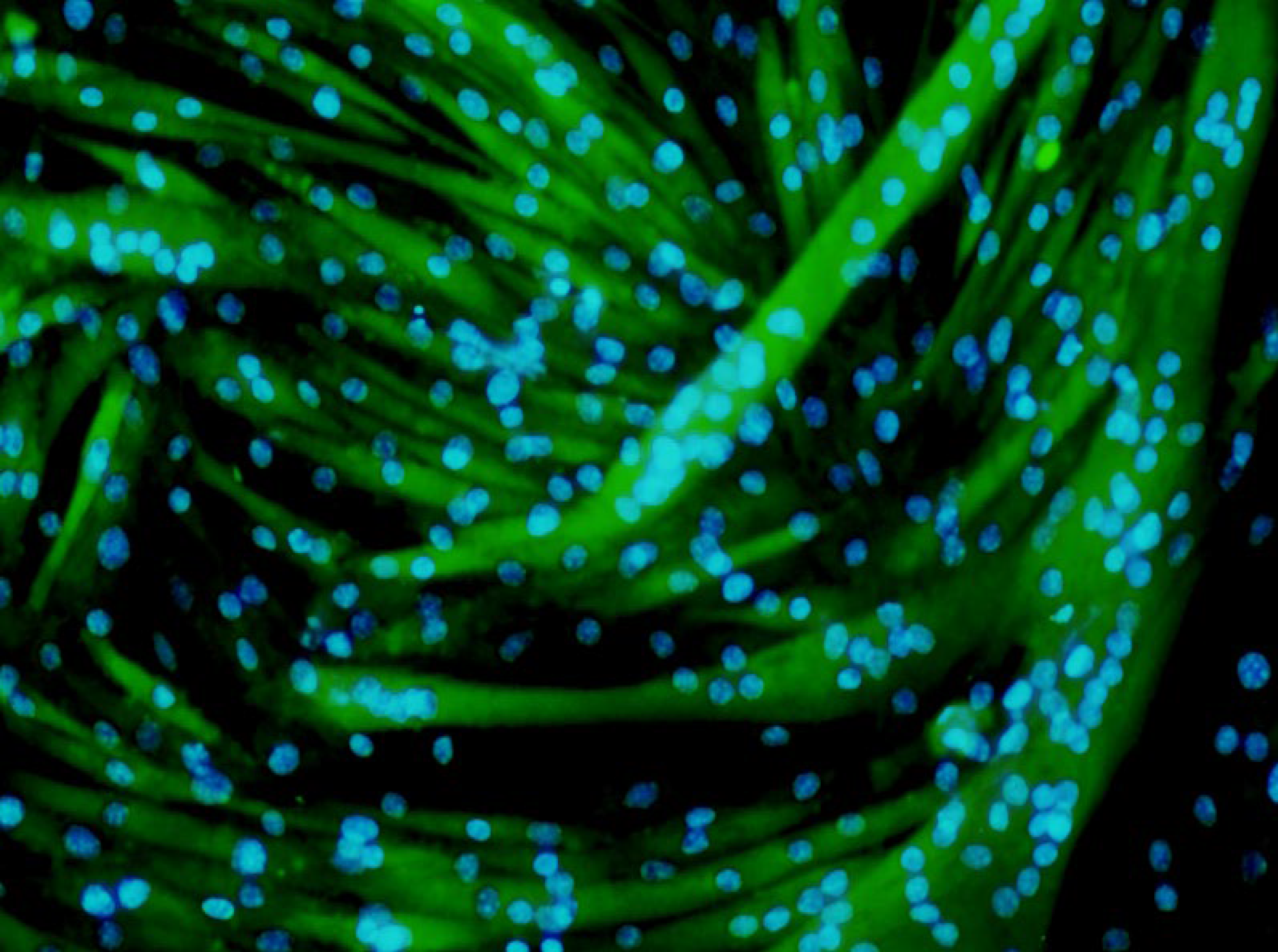

Supplement: Supplementary file 3 — Appendix Figure Source Data [file 44318_2024_285_MOESM3_ESM.zip › Appendix Figure S8/SF 8A/SF8-A-mFNDC1.tif]

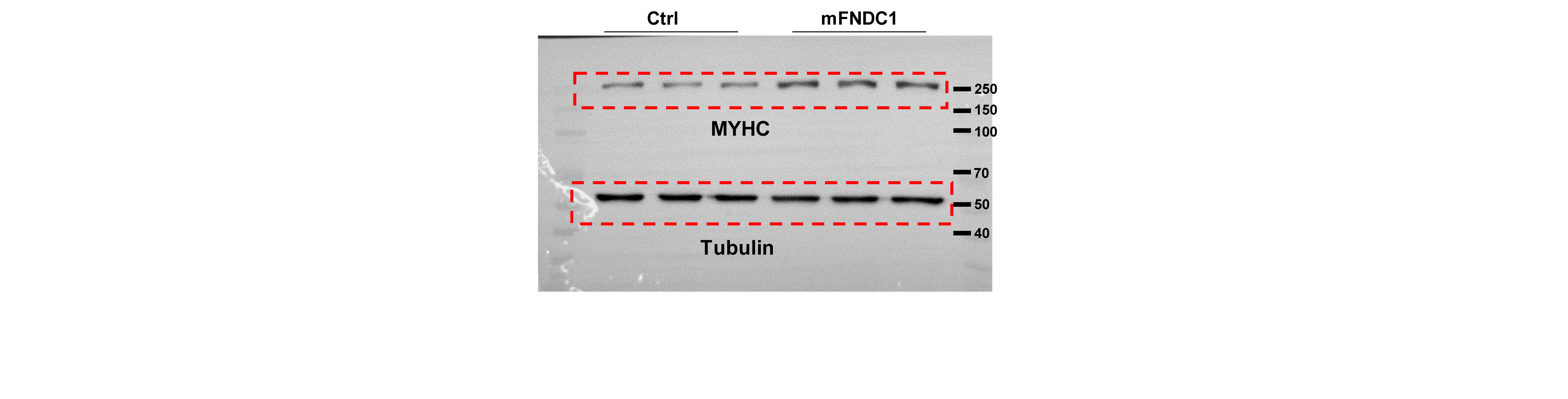

Supplement: Supplementary file 3 — Appendix Figure Source Data [file 44318_2024_285_MOESM3_ESM.zip › Appendix Figure S8/SF 8E/SF-8-E-MYHC-TUBULIN.tif]

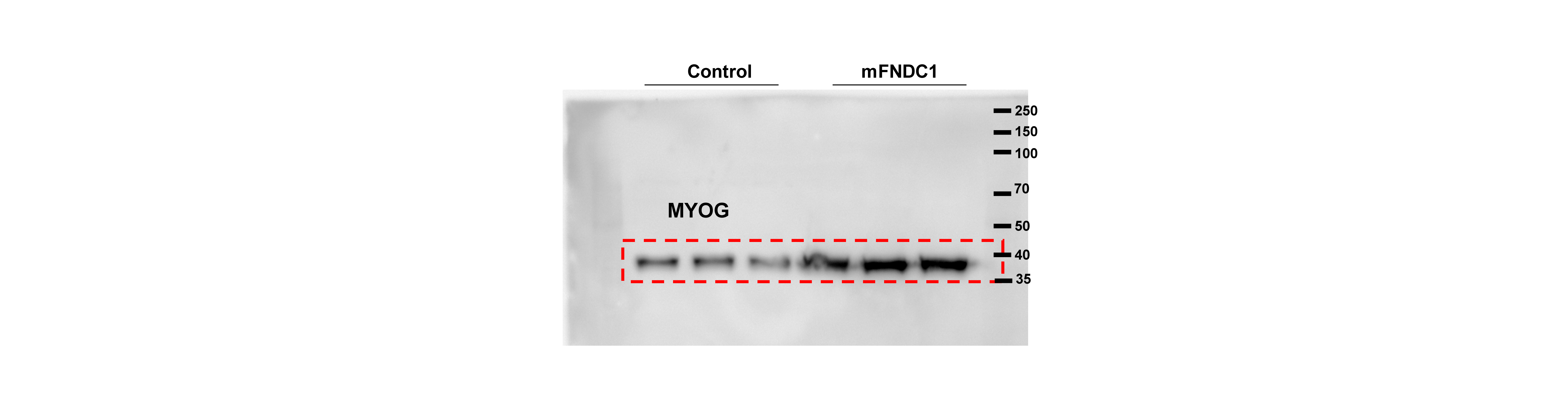

Supplement: Supplementary file 3 — Appendix Figure Source Data [file 44318_2024_285_MOESM3_ESM.zip › Appendix Figure S8/SF 8E/SF-8-E-MYOG.tif]

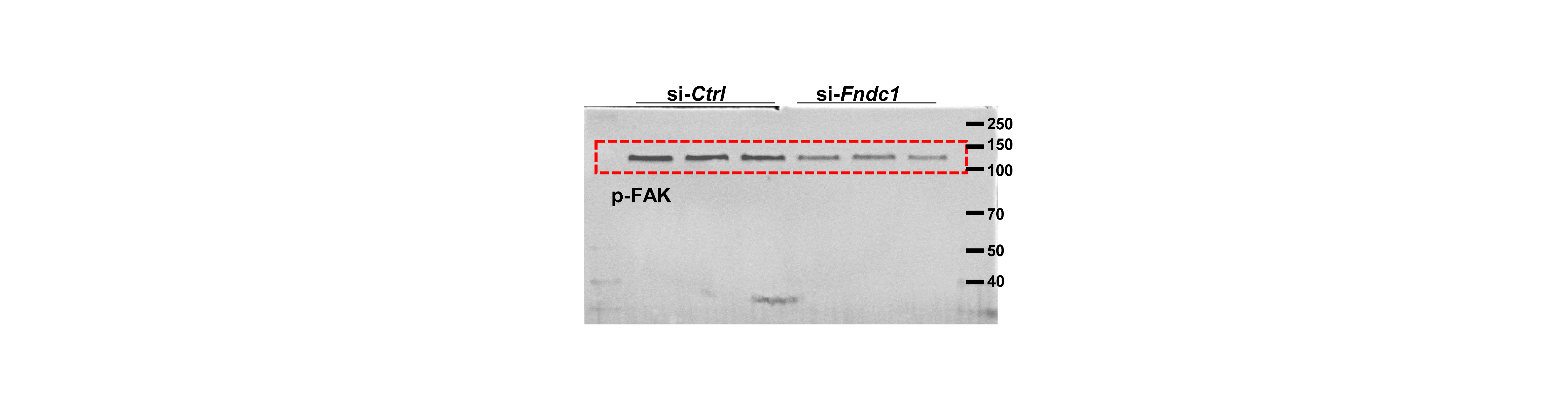

Supplement: Supplementary file 3 — Appendix Figure Source Data [file 44318_2024_285_MOESM3_ESM.zip › Appendix Figure S9/SF 9C/SF-9-C-pFAK.tif]

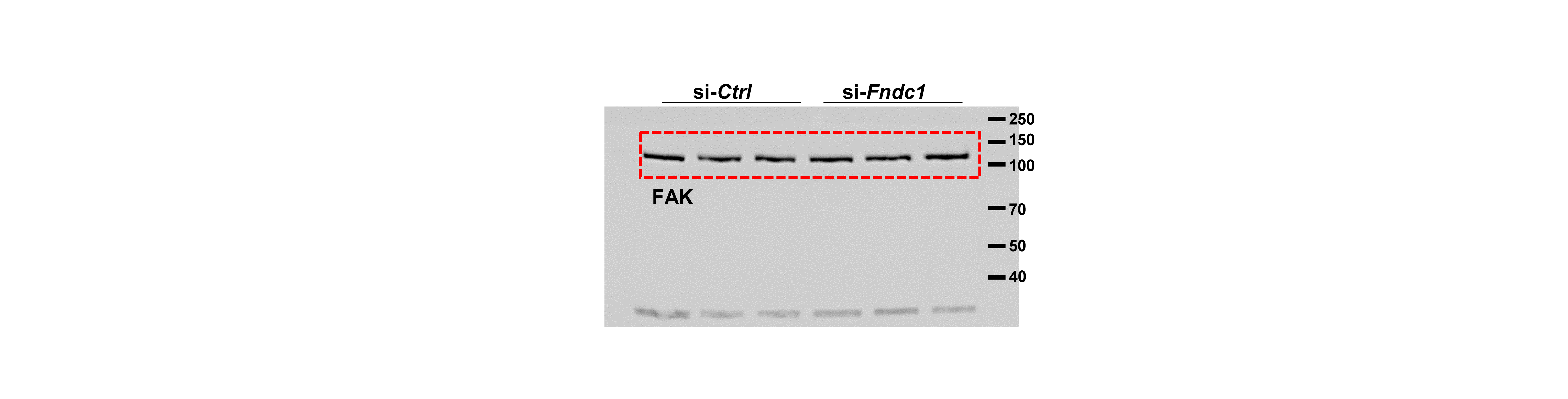

Supplement: Supplementary file 3 — Appendix Figure Source Data [file 44318_2024_285_MOESM3_ESM.zip › Appendix Figure S9/SF 9C/SF-9C-FAK.tif]

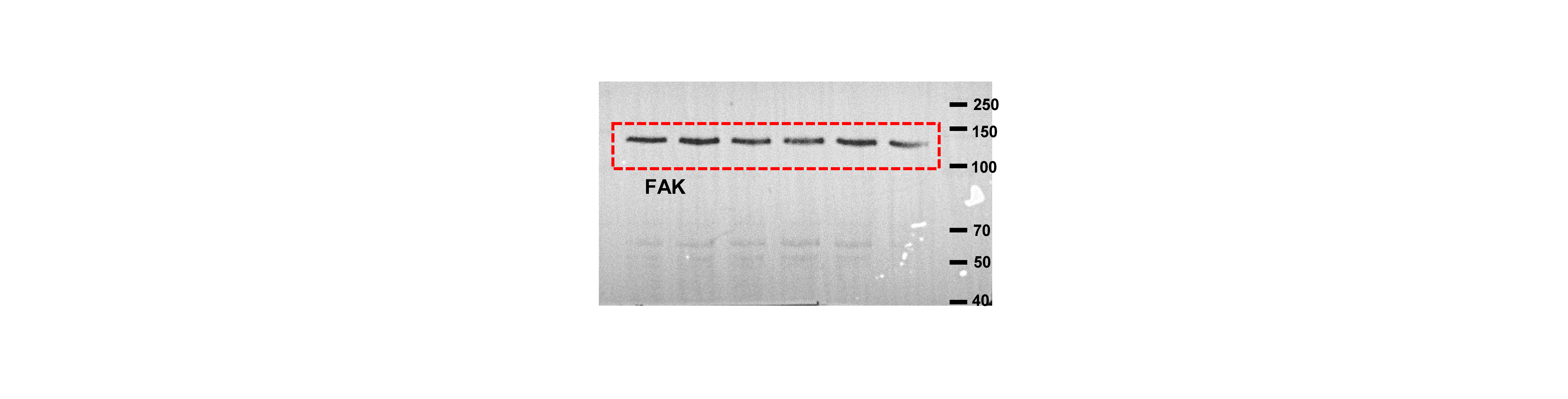

Supplement: Supplementary file 3 — Appendix Figure Source Data [file 44318_2024_285_MOESM3_ESM.zip › Appendix Figure S9/SF 9D/SF-9-D-FAK.tif]

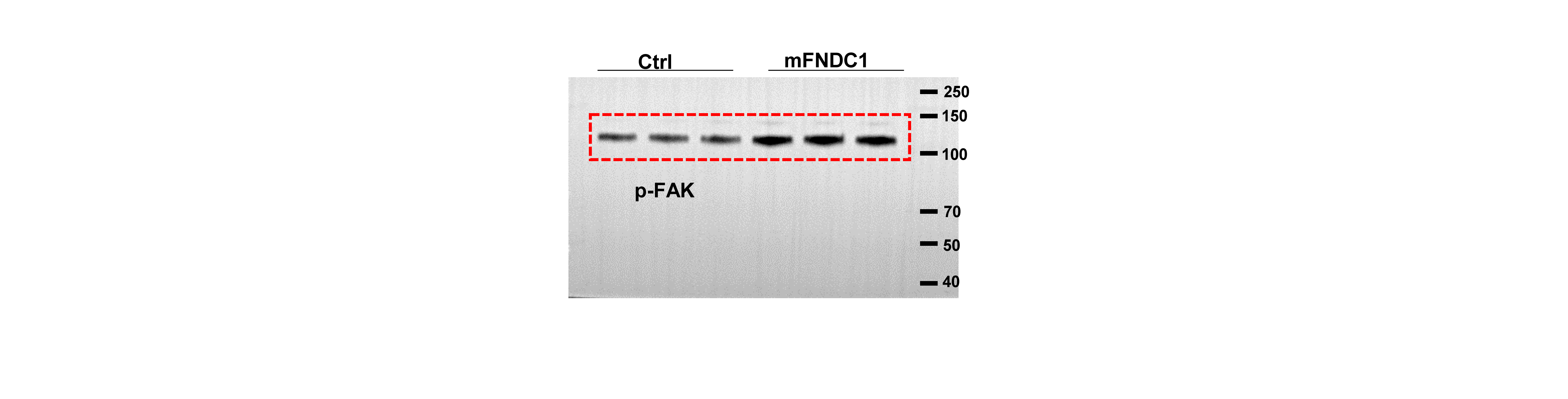

Supplement: Supplementary file 3 — Appendix Figure Source Data [file 44318_2024_285_MOESM3_ESM.zip › Appendix Figure S9/SF 9D/SF-9-D-p-FAK.tif]

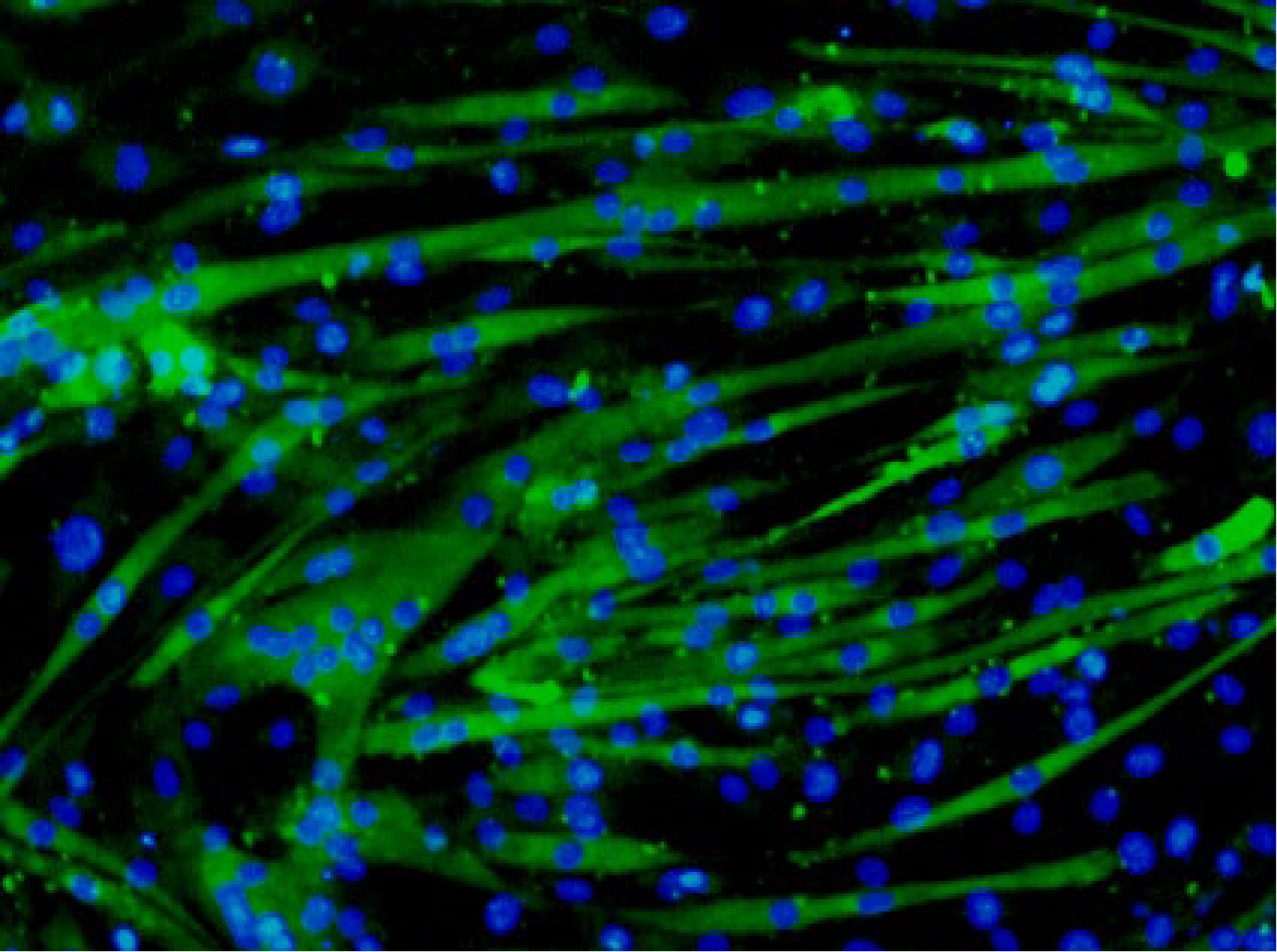

Supplement: Supplementary file 3 — Appendix Figure Source Data [file 44318_2024_285_MOESM3_ESM.zip › Appendix Figure S10/SF 10A/SF10-A-Control-siControl.tif]

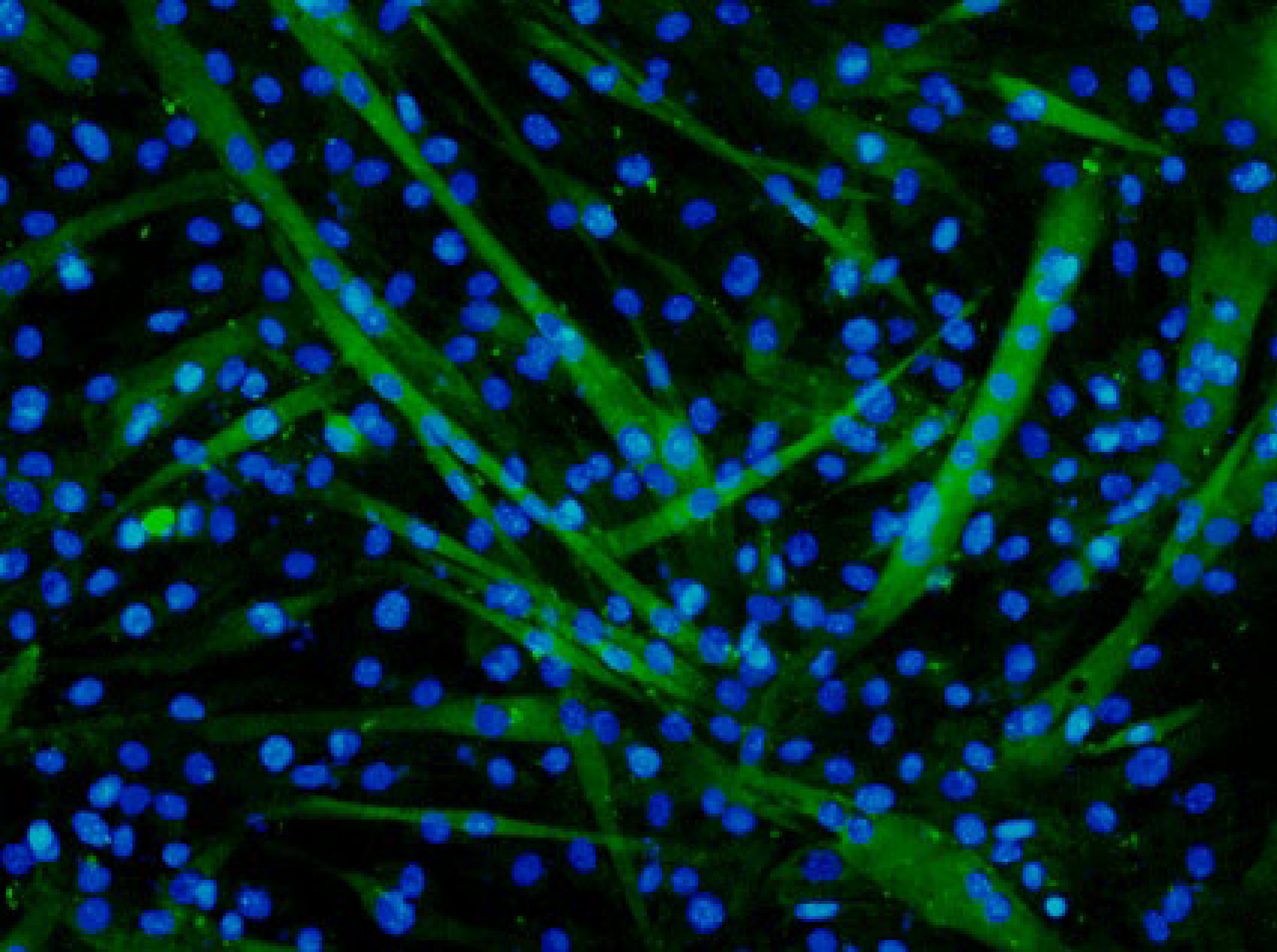

Supplement: Supplementary file 3 — Appendix Figure Source Data [file 44318_2024_285_MOESM3_ESM.zip › Appendix Figure S10/SF 10A/SF10-A-Control-siItgb1.tif]

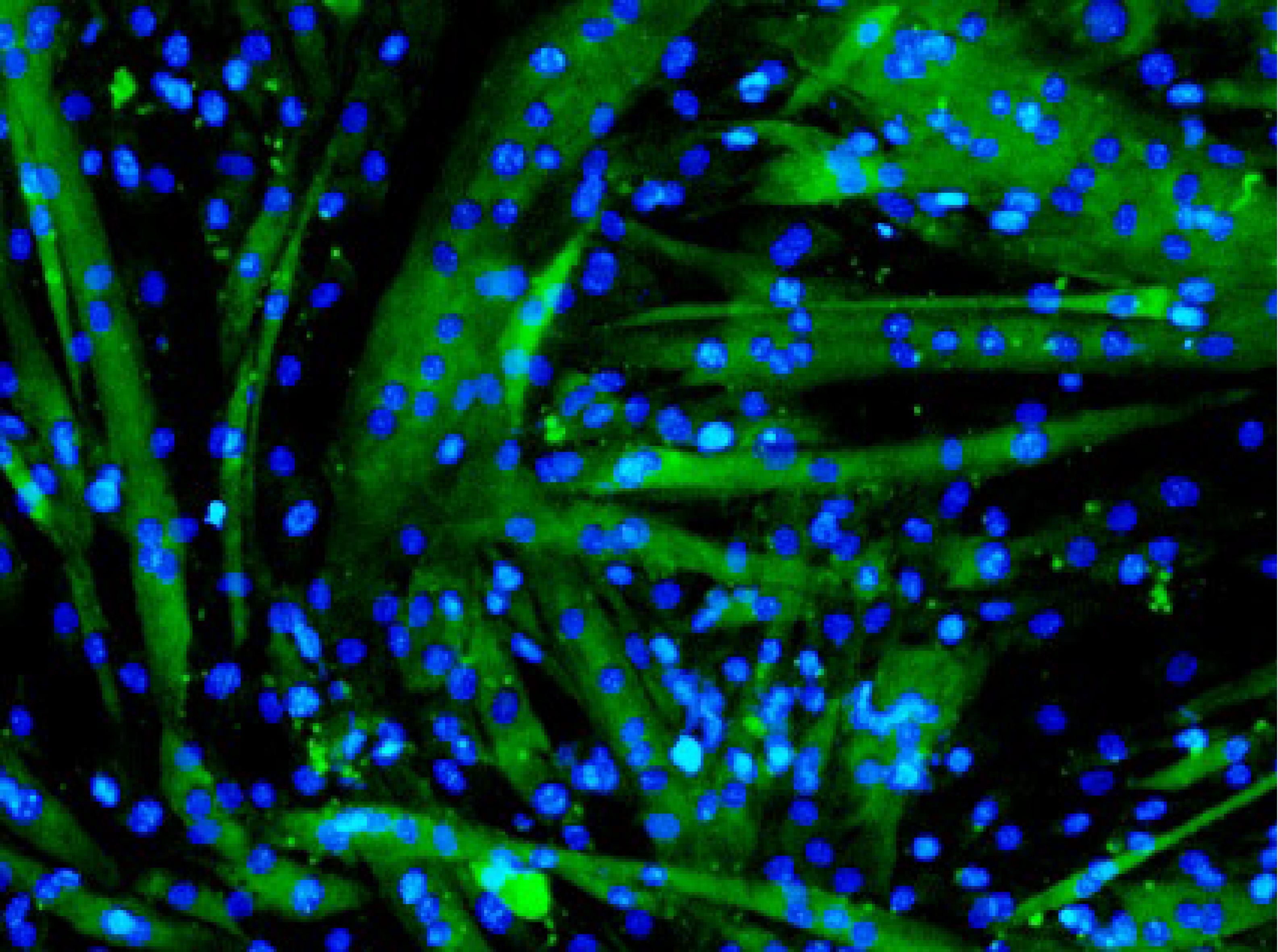

Supplement: Supplementary file 3 — Appendix Figure Source Data [file 44318_2024_285_MOESM3_ESM.zip › Appendix Figure S10/SF 10A/SF10-A-mFNDC1-sicontrol.tif]

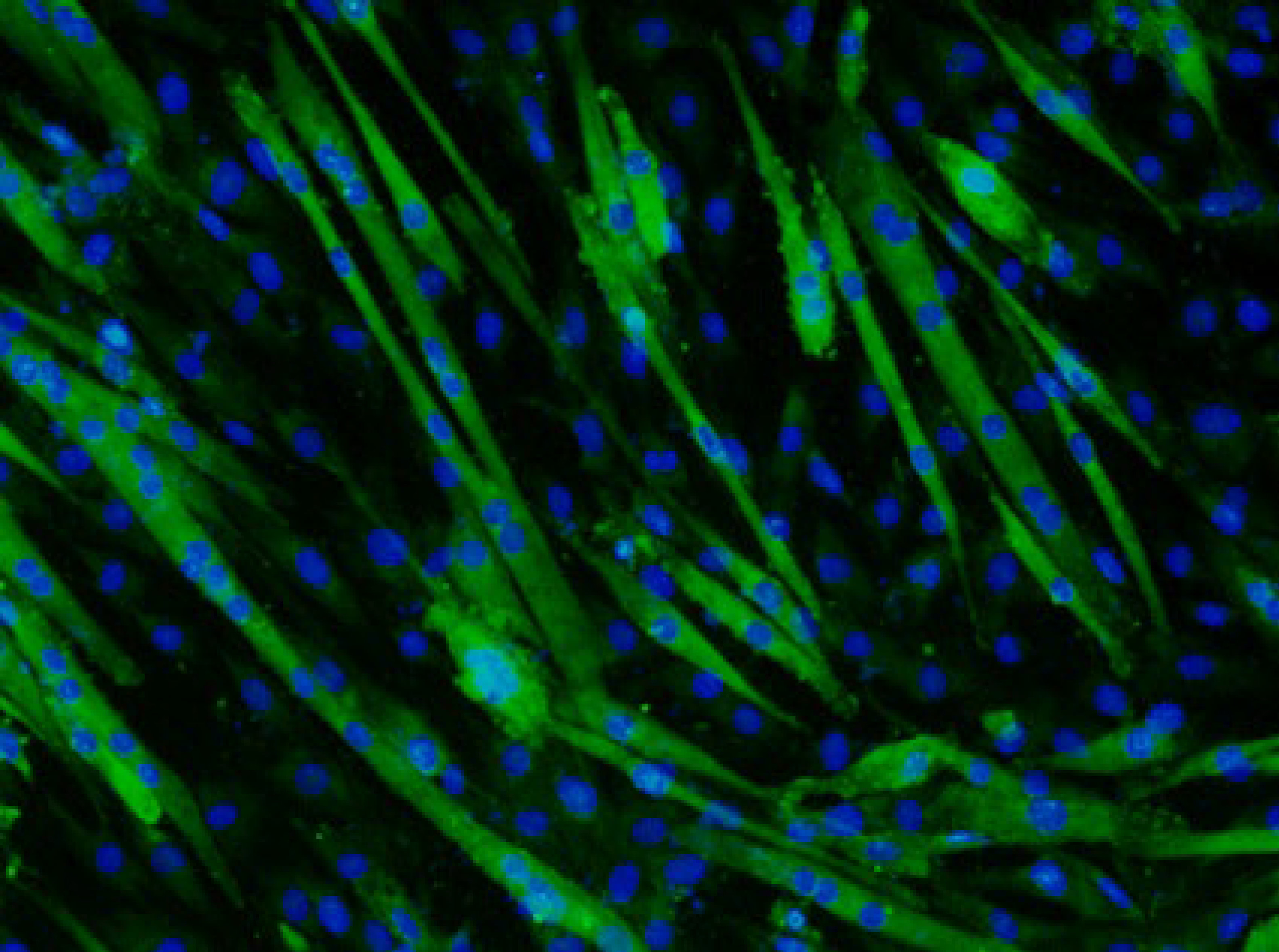

Supplement: Supplementary file 3 — Appendix Figure Source Data [file 44318_2024_285_MOESM3_ESM.zip › Appendix Figure S10/SF 10A/SF10-A-mFNDC1-siItgb1.tif]

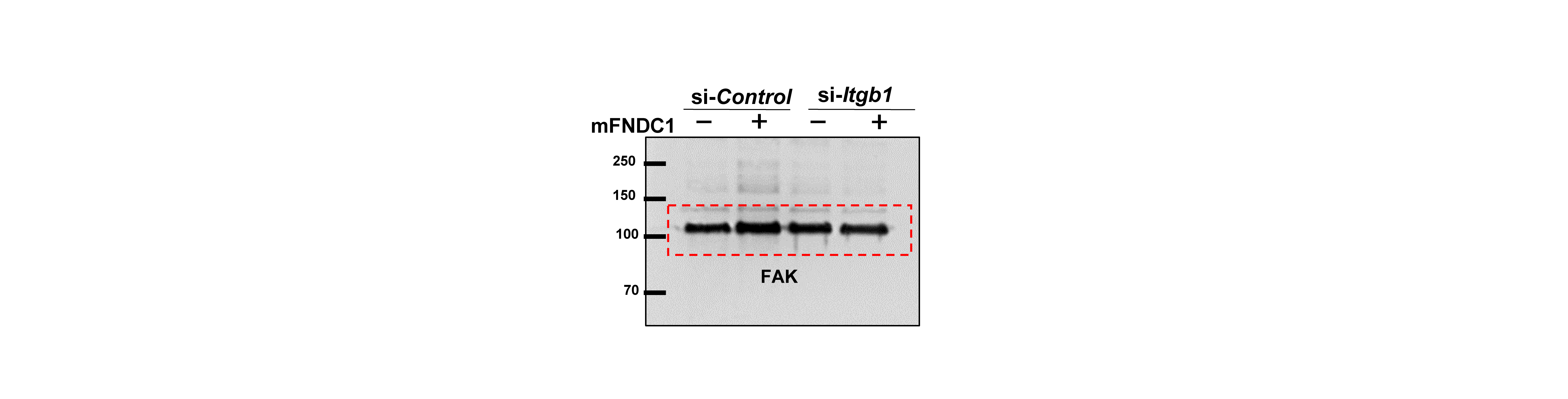

Supplement: Supplementary file 3 — Appendix Figure Source Data [file 44318_2024_285_MOESM3_ESM.zip › Appendix Figure S10/SF 10D/SF-10-D-FAK.tif]

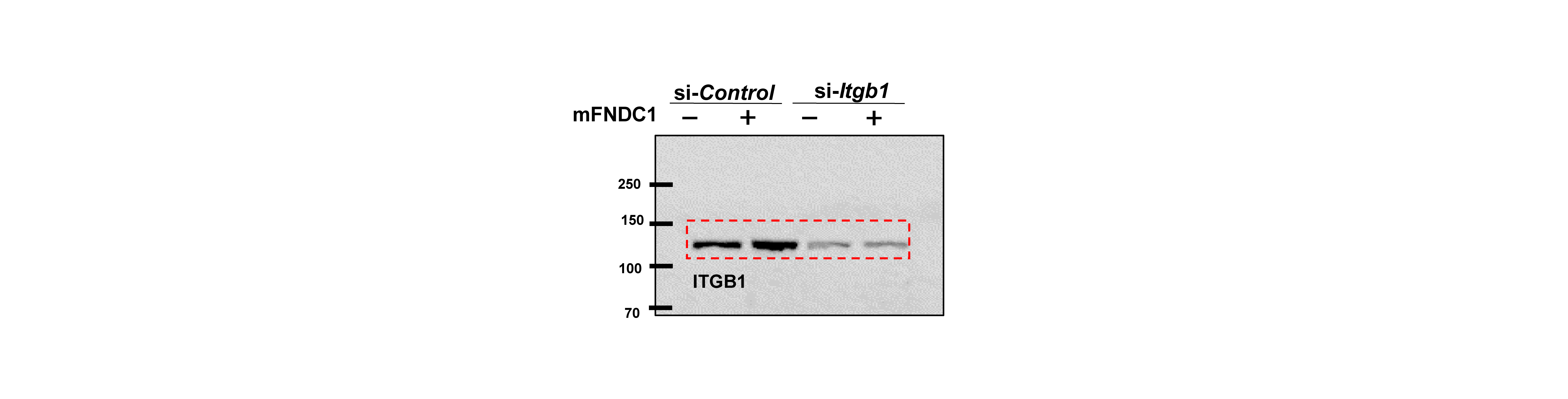

Supplement: Supplementary file 3 — Appendix Figure Source Data [file 44318_2024_285_MOESM3_ESM.zip › Appendix Figure S10/SF 10D/SF-10-D-ITGB1.tif]

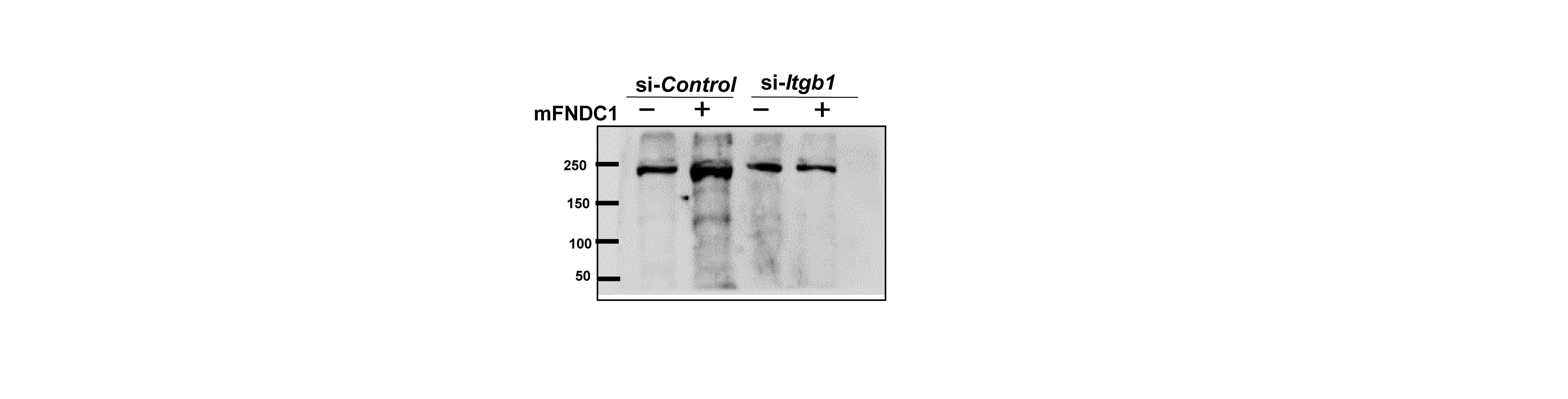

Supplement: Supplementary file 3 — Appendix Figure Source Data [file 44318_2024_285_MOESM3_ESM.zip › Appendix Figure S10/SF 10D/SF-10-D-MYHC.tif]

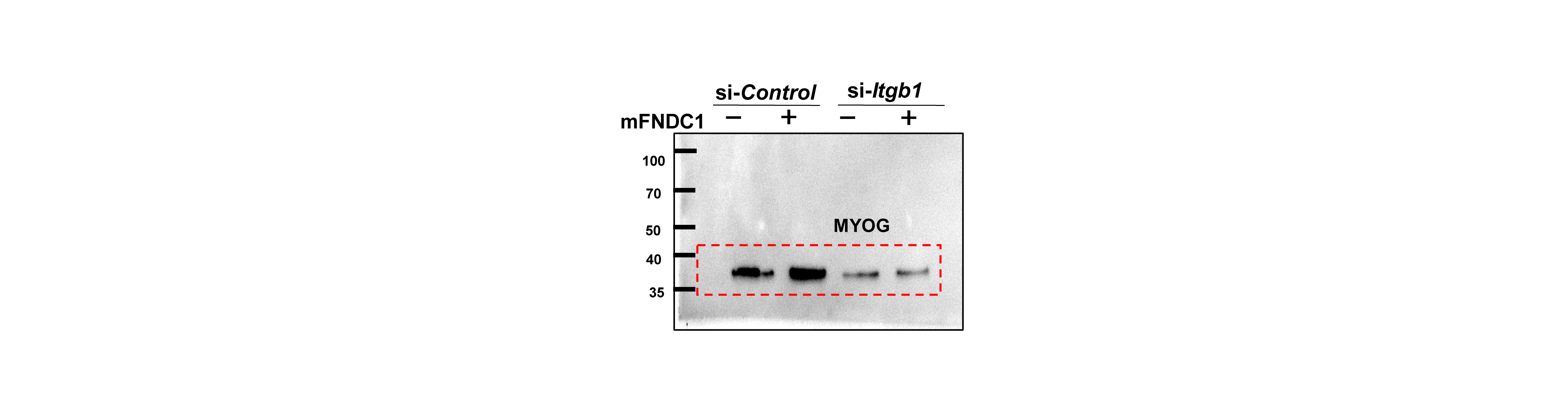

Supplement: Supplementary file 3 — Appendix Figure Source Data [file 44318_2024_285_MOESM3_ESM.zip › Appendix Figure S10/SF 10D/SF-10-D-MYOG.tif]

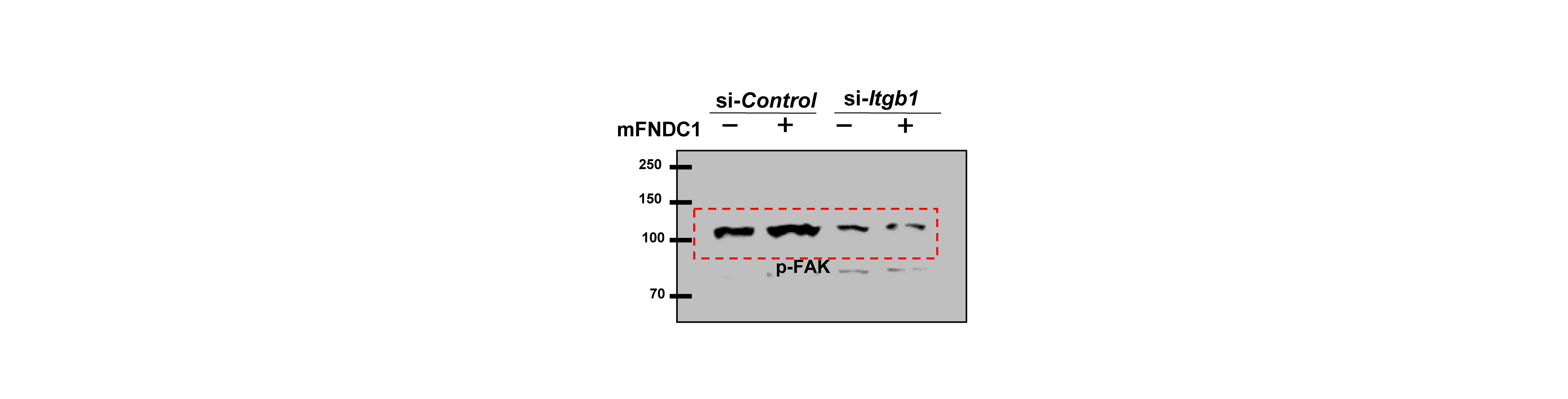

Supplement: Supplementary file 3 — Appendix Figure Source Data [file 44318_2024_285_MOESM3_ESM.zip › Appendix Figure S10/SF 10D/SF-10-D-p-FAK.tif]

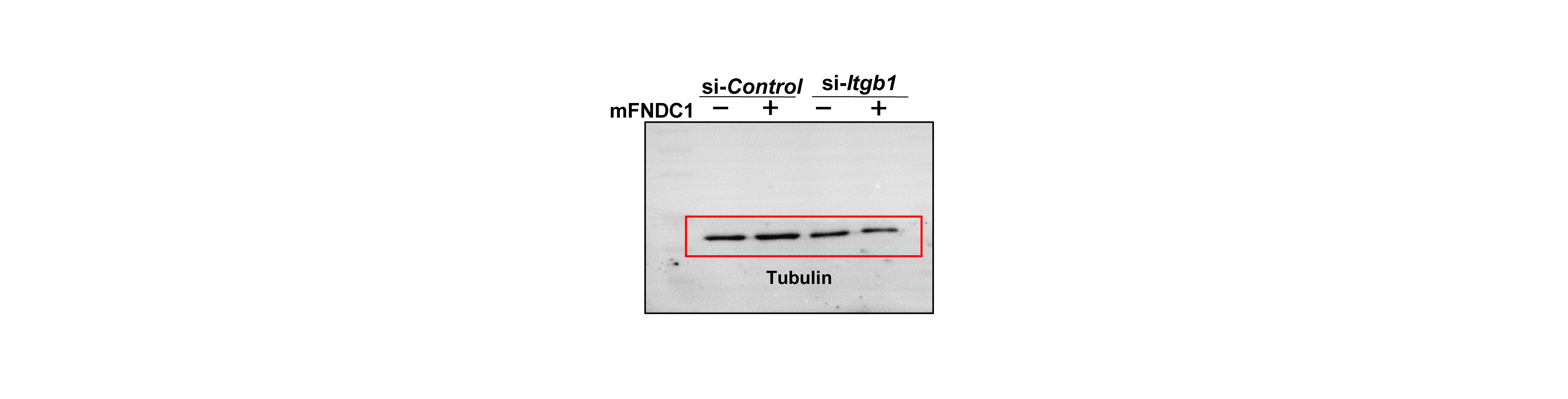

Supplement: Supplementary file 3 — Appendix Figure Source Data [file 44318_2024_285_MOESM3_ESM.zip › Appendix Figure S10/SF 10D/SF-10-D-TUBULIN.tif]

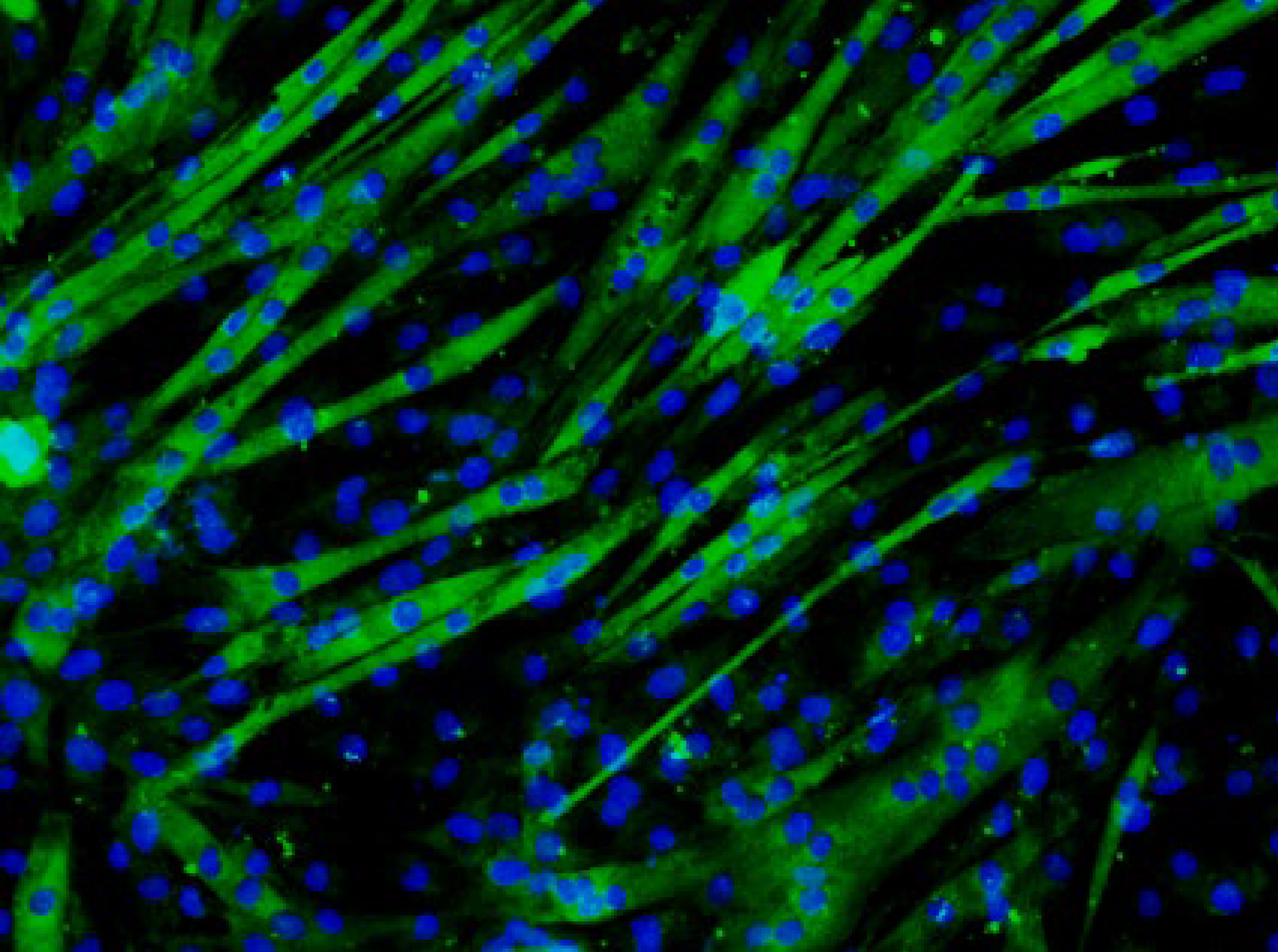

Supplement: Supplementary file 3 — Appendix Figure Source Data [file 44318_2024_285_MOESM3_ESM.zip › Appendix Figure S10/SF 10F/SF10-F-Control-PBS.tif]

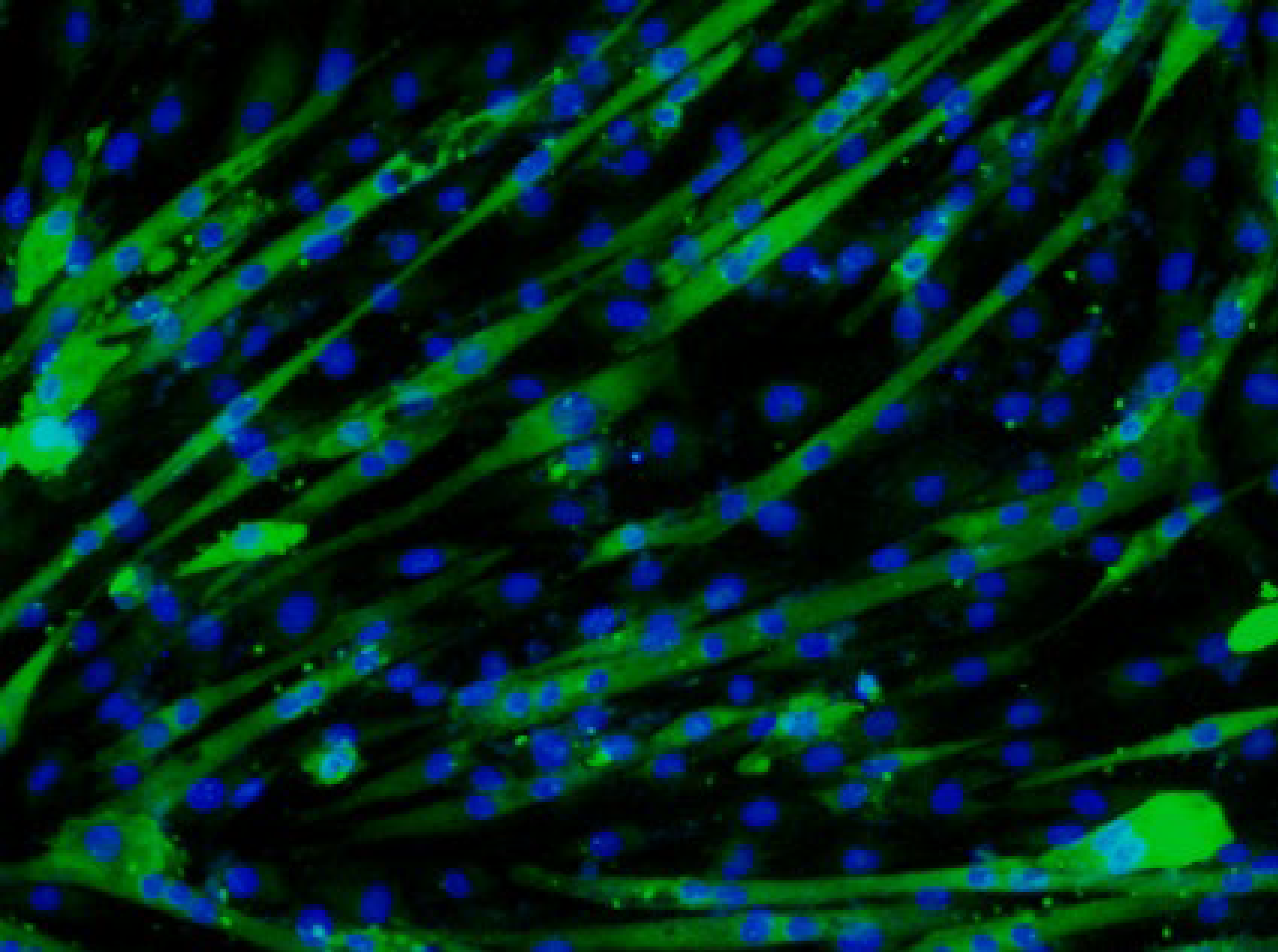

Supplement: Supplementary file 3 — Appendix Figure Source Data [file 44318_2024_285_MOESM3_ESM.zip › Appendix Figure S10/SF 10F/SF10-F-Control-RGDs.tif]

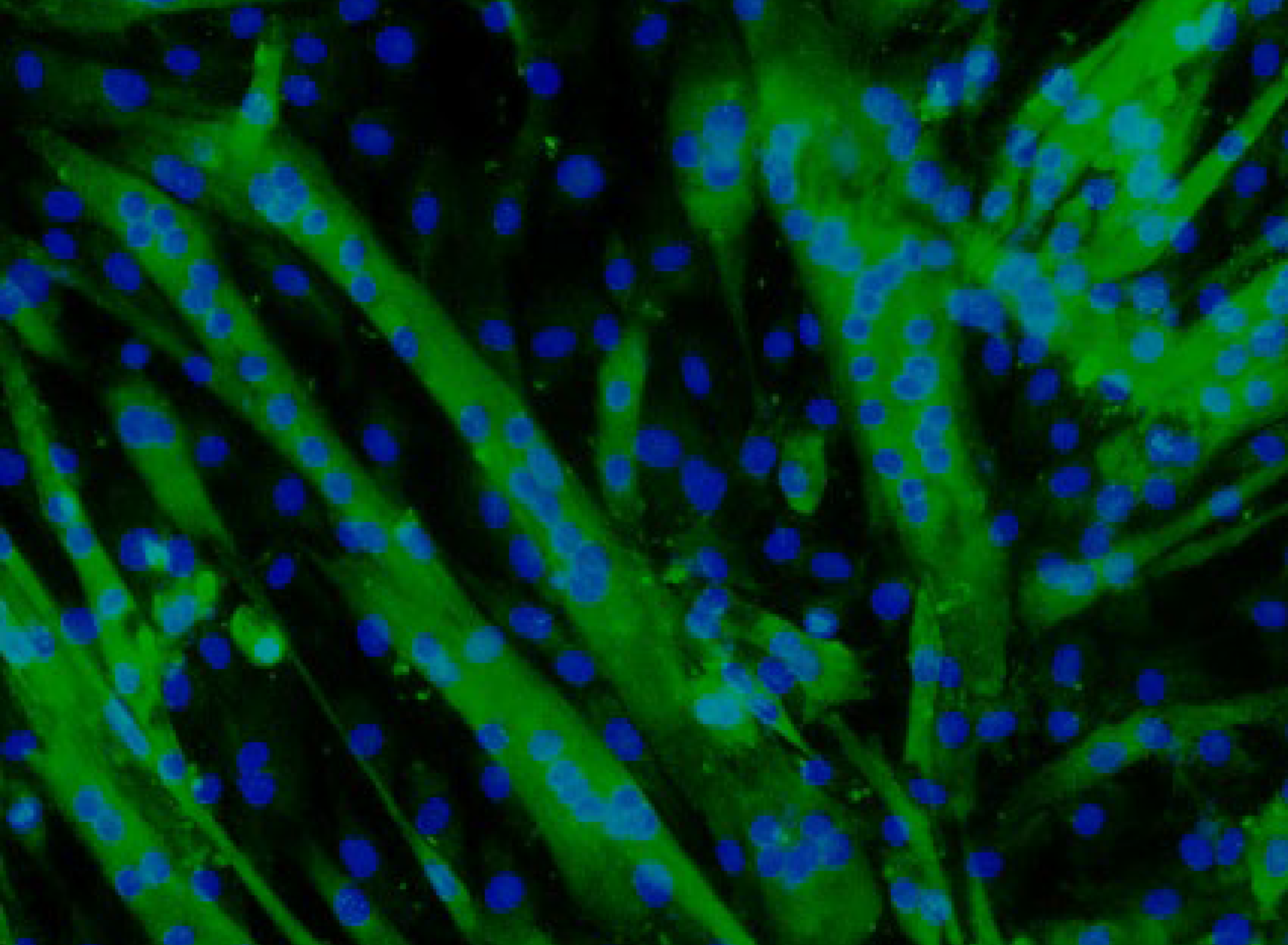

Supplement: Supplementary file 3 — Appendix Figure Source Data [file 44318_2024_285_MOESM3_ESM.zip › Appendix Figure S10/SF 10F/SF10-F-mFNDC1-PBS.tif]

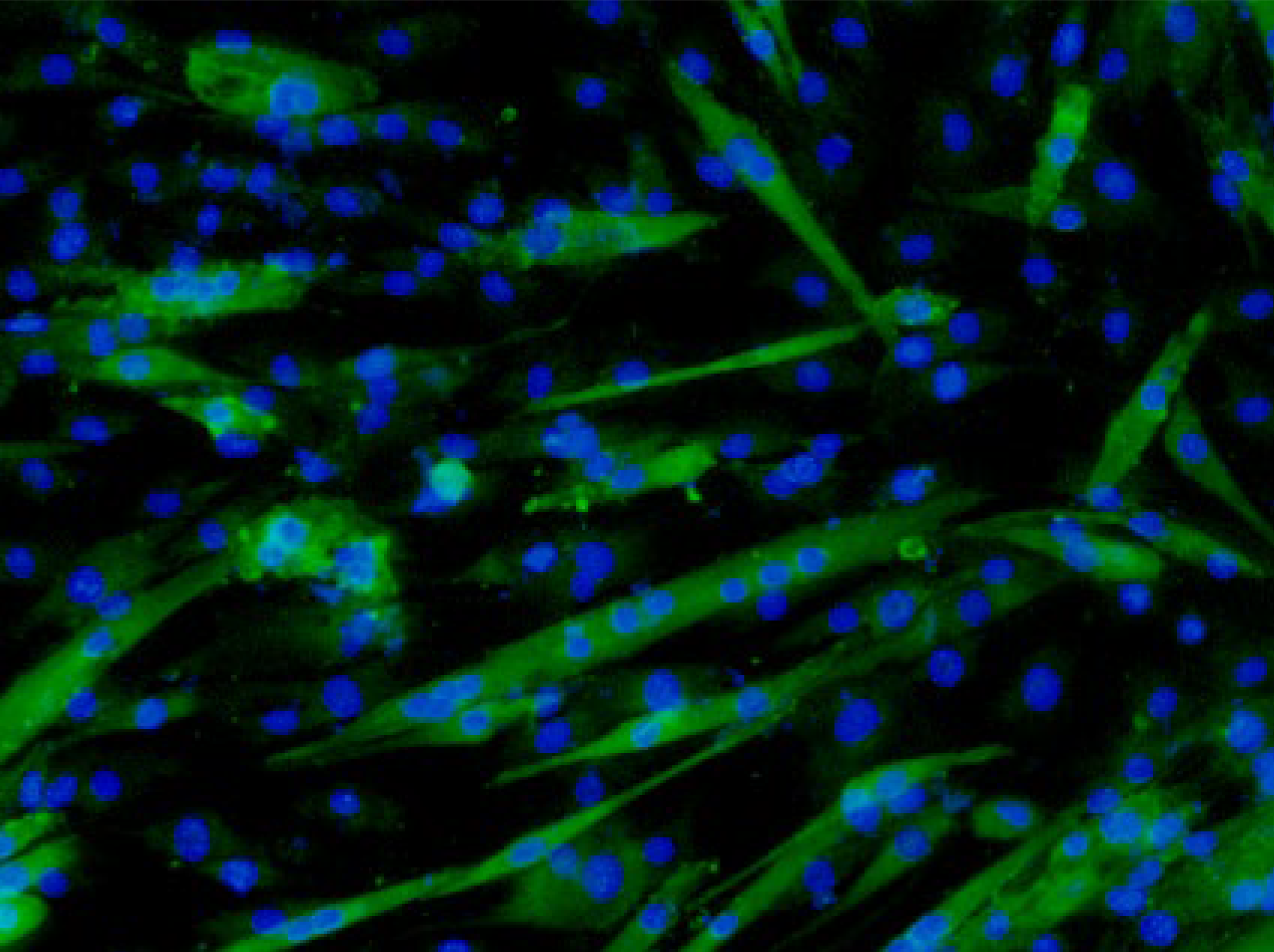

Supplement: Supplementary file 3 — Appendix Figure Source Data [file 44318_2024_285_MOESM3_ESM.zip › Appendix Figure S10/SF 10F/SF10-F-mFNDC1-RGDs.tif]

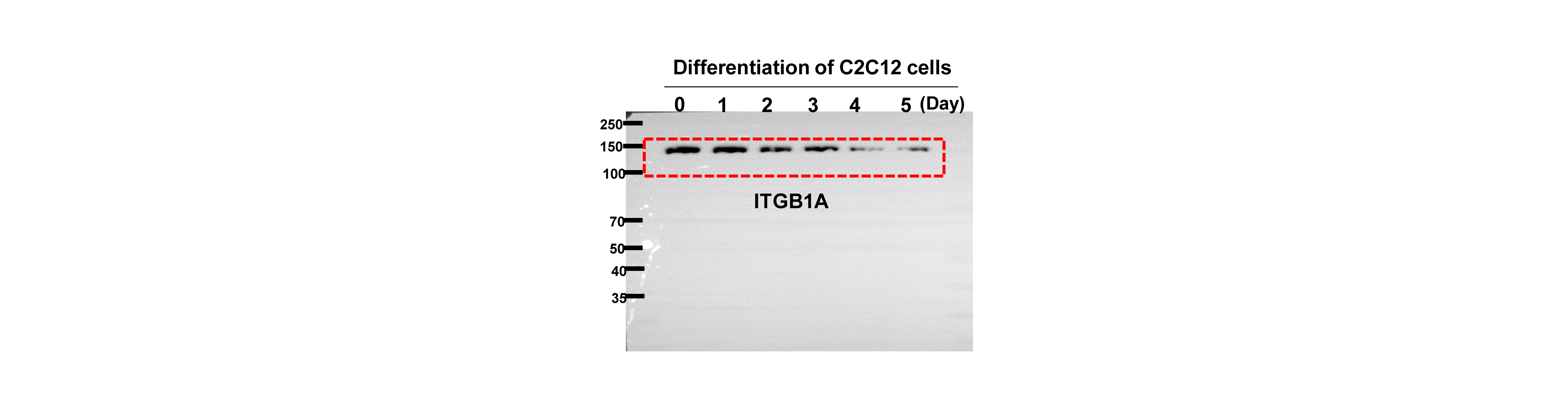

Supplement: Supplementary file 3 — Appendix Figure Source Data [file 44318_2024_285_MOESM3_ESM.zip › Appendix Figure S11/SF 11A/SF-11-A-ITGB1A.tif]

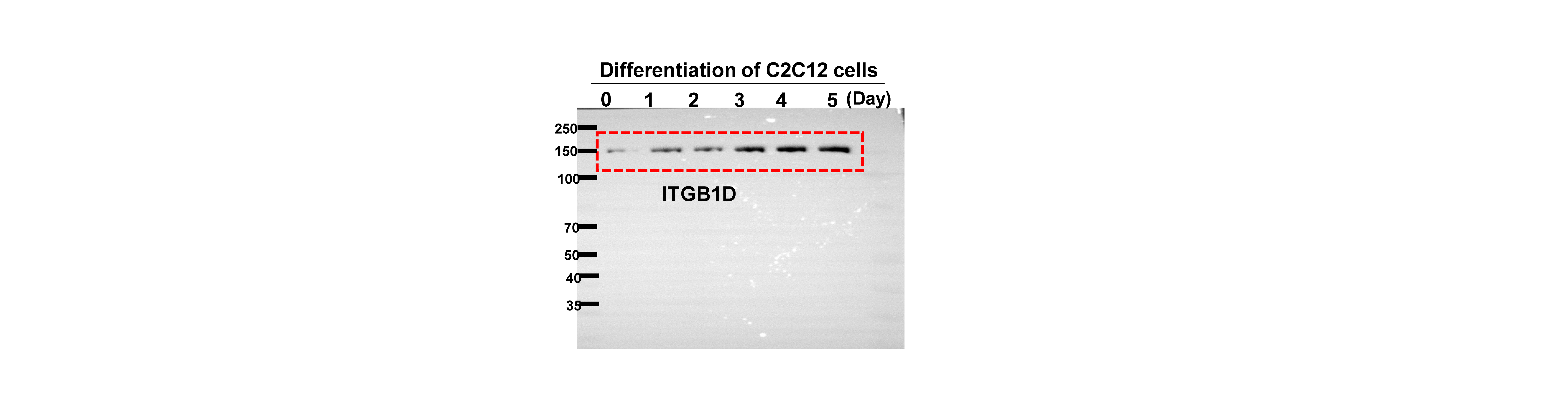

Supplement: Supplementary file 3 — Appendix Figure Source Data [file 44318_2024_285_MOESM3_ESM.zip › Appendix Figure S11/SF 11A/SF-11-A-ITGB1D.tif]

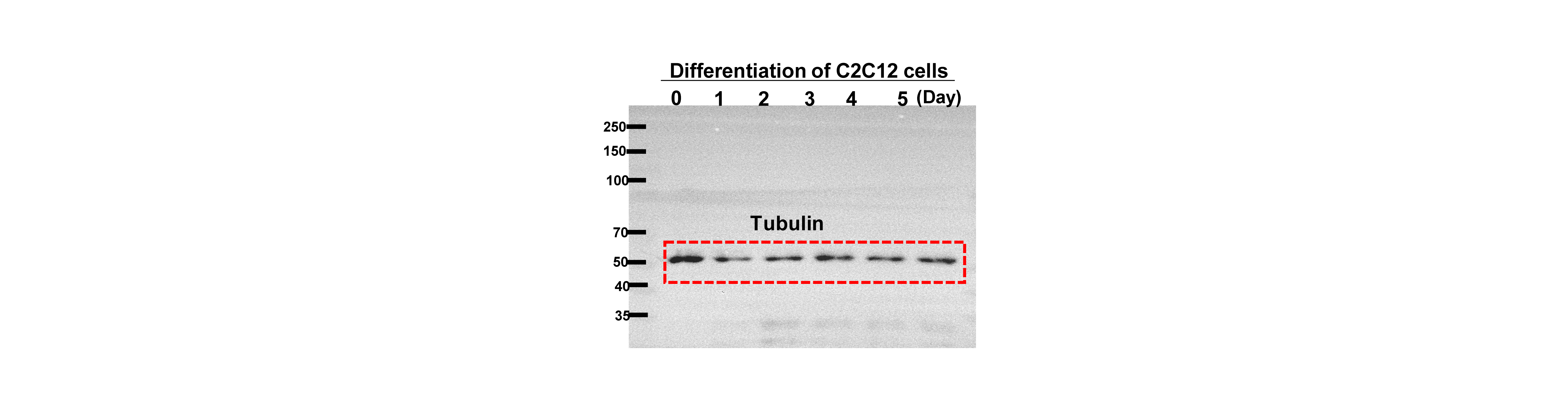

Supplement: Supplementary file 3 — Appendix Figure Source Data [file 44318_2024_285_MOESM3_ESM.zip › Appendix Figure S11/SF 11A/SF-11-A-TUBULIN.tif]

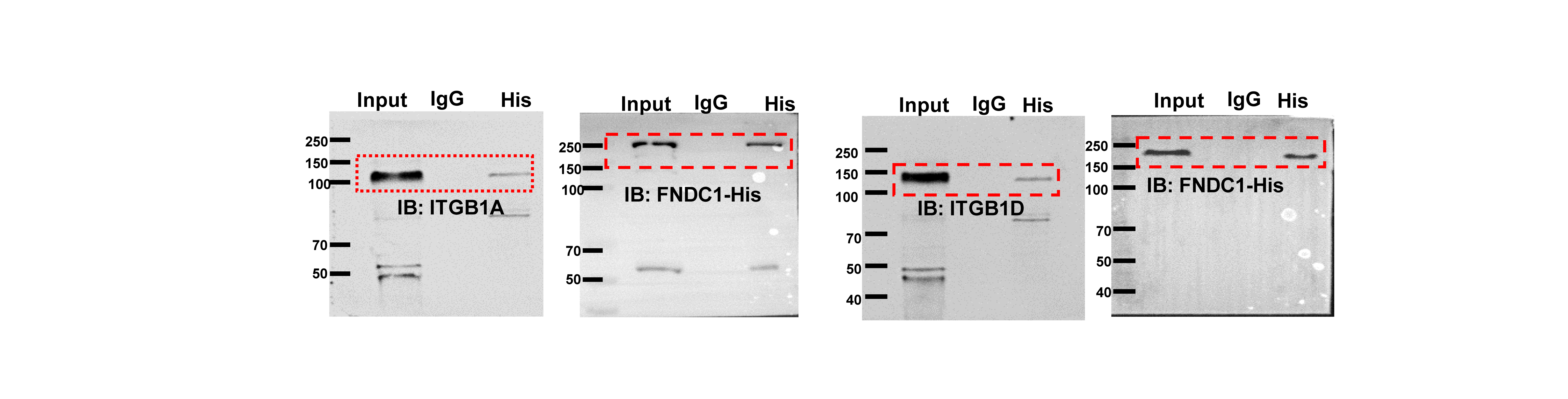

Supplement: Supplementary file 3 — Appendix Figure Source Data [file 44318_2024_285_MOESM3_ESM.zip › Appendix Figure S11/SF 11B/SF-11-B.tif]

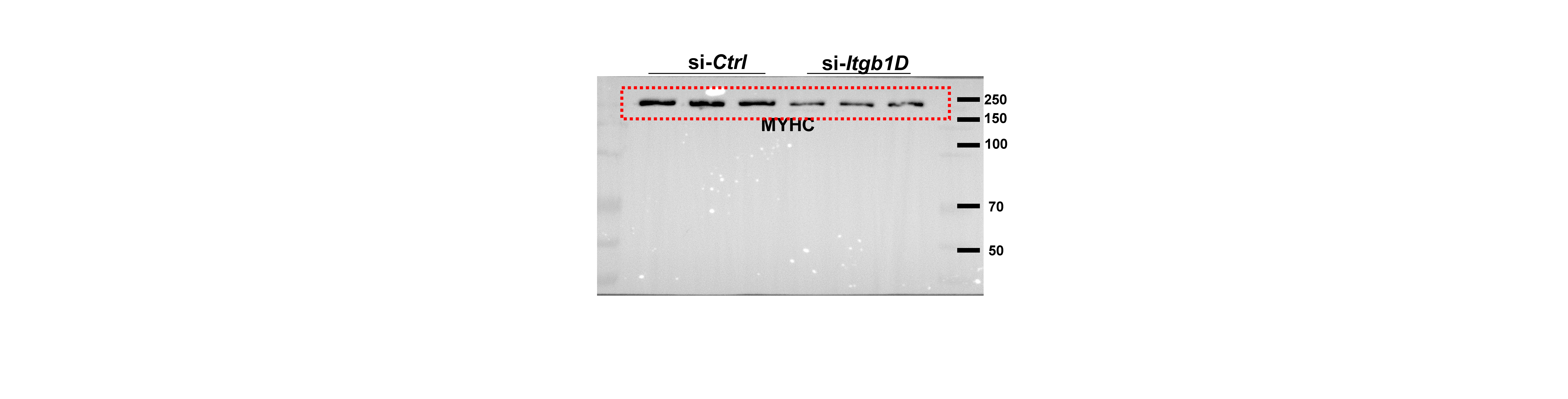

Supplement: Supplementary file 3 — Appendix Figure Source Data [file 44318_2024_285_MOESM3_ESM.zip › Appendix Figure S11/SF 11C/SF-11-C-MYHC.tif]

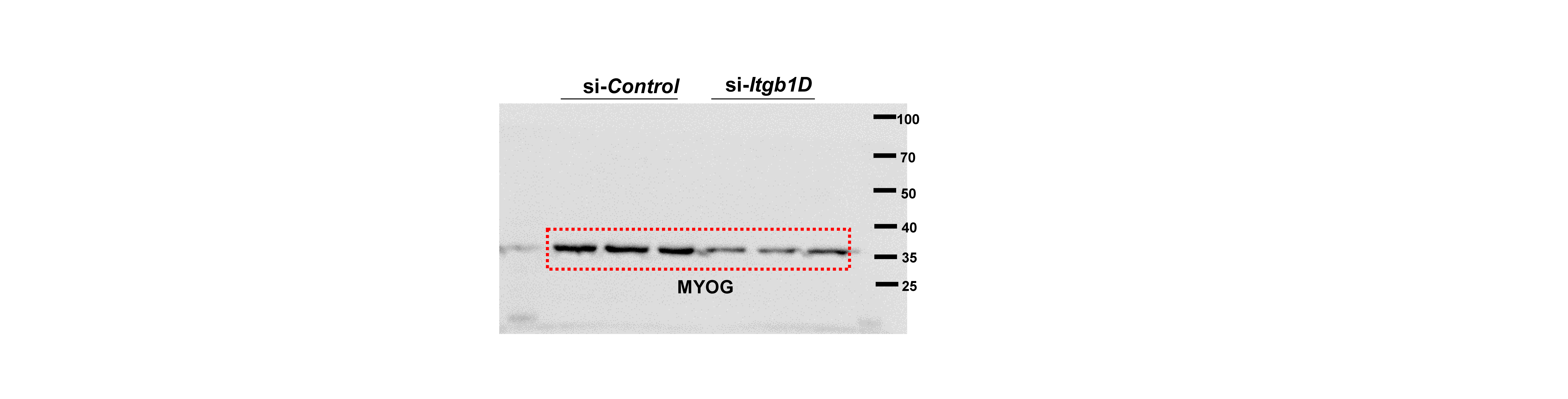

Supplement: Supplementary file 3 — Appendix Figure Source Data [file 44318_2024_285_MOESM3_ESM.zip › Appendix Figure S11/SF 11C/SF-11-C-MYOG.tif]

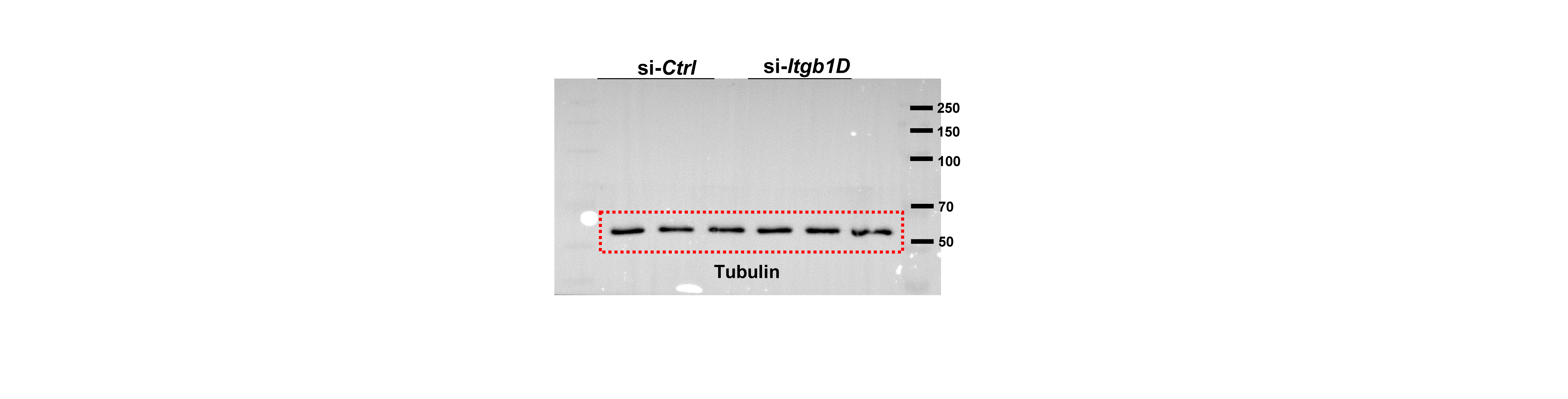

Supplement: Supplementary file 3 — Appendix Figure Source Data [file 44318_2024_285_MOESM3_ESM.zip › Appendix Figure S11/SF 11C/SF-11-C-YUBULIN.tif]

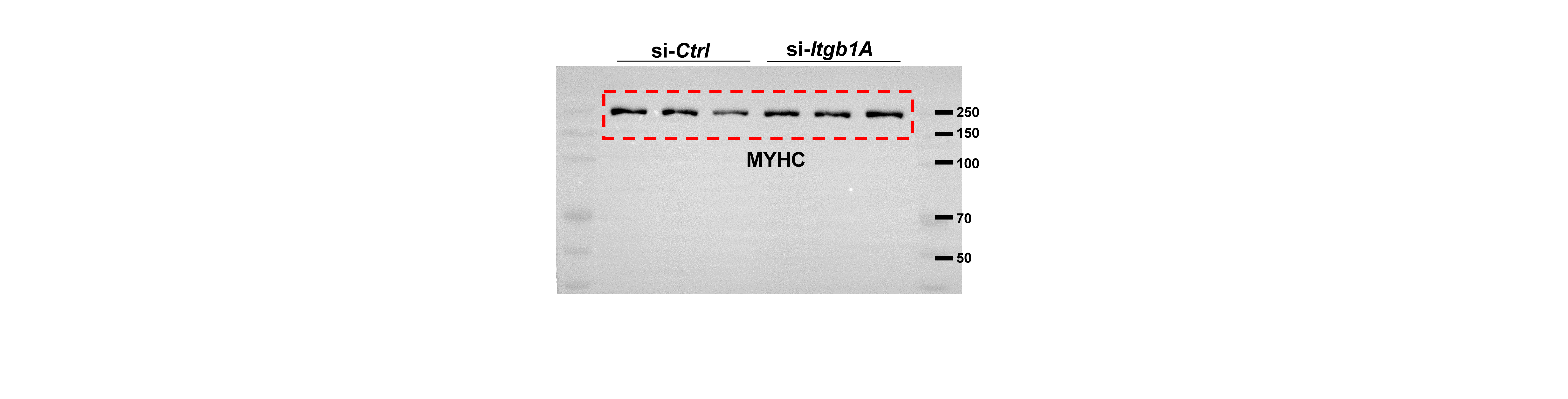

Supplement: Supplementary file 3 — Appendix Figure Source Data [file 44318_2024_285_MOESM3_ESM.zip › Appendix Figure S11/SF 11D/SF-11-D-MYHC.tif]

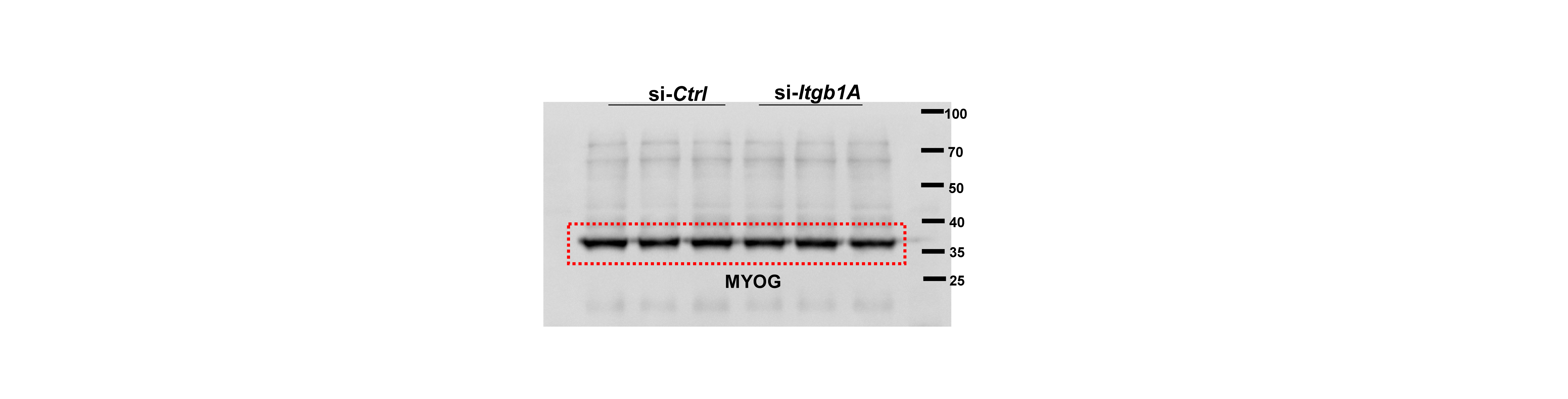

Supplement: Supplementary file 3 — Appendix Figure Source Data [file 44318_2024_285_MOESM3_ESM.zip › Appendix Figure S11/SF 11D/SF-11-D-MYOG.tif]

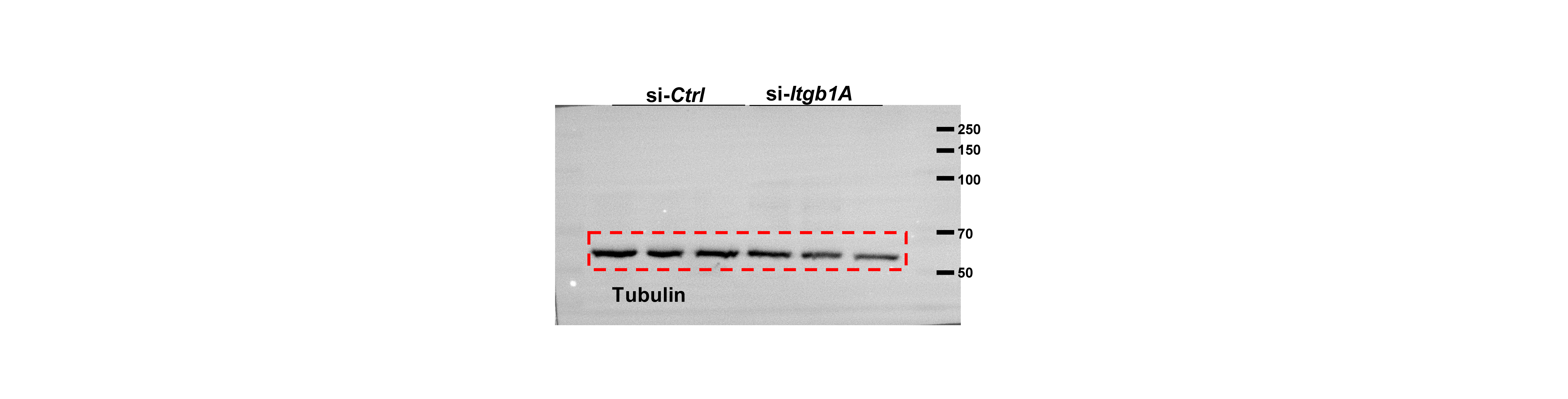

Supplement: Supplementary file 3 — Appendix Figure Source Data [file 44318_2024_285_MOESM3_ESM.zip › Appendix Figure S11/SF 11D/SF-11-DTUBULIN.tif]

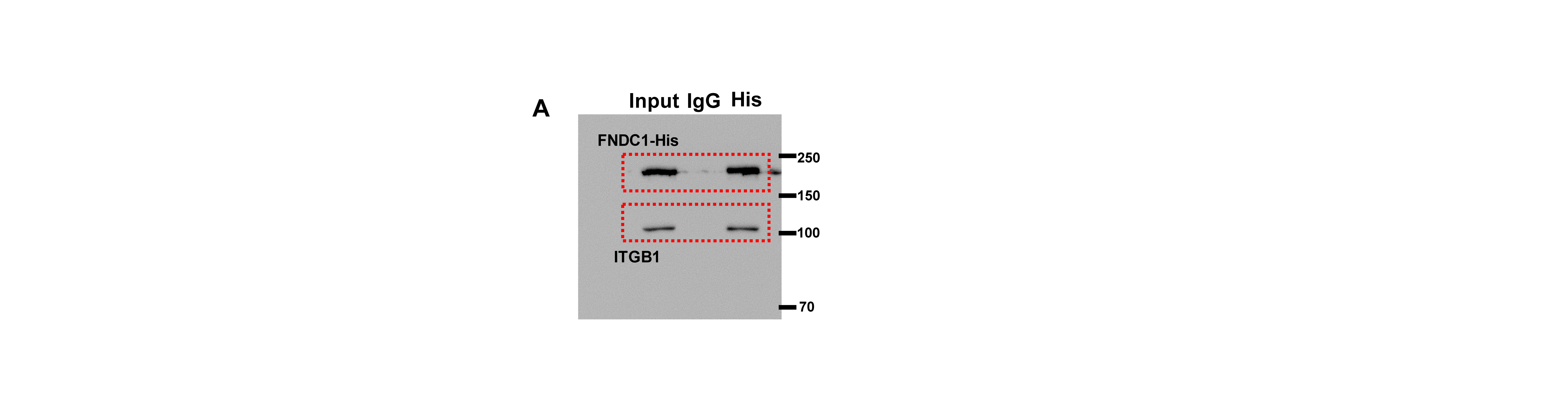

Supplement: Supplementary file 3 — Appendix Figure Source Data [file 44318_2024_285_MOESM3_ESM.zip › Appendix Figure S12/SF 12A/SF-12-A.tif]

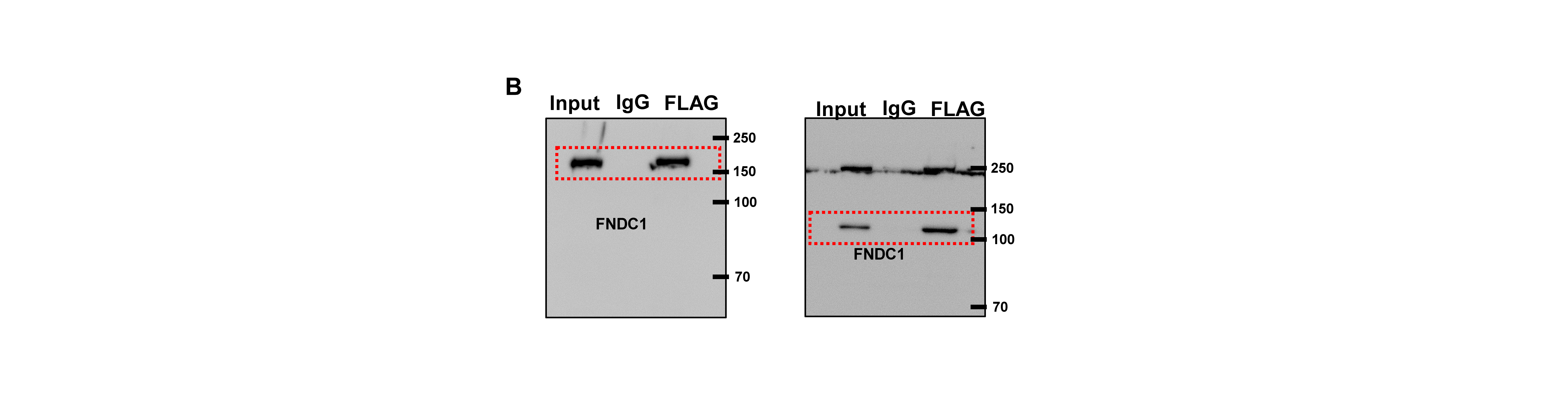

Supplement: Supplementary file 3 — Appendix Figure Source Data [file 44318_2024_285_MOESM3_ESM.zip › Appendix Figure S12/SF 12B/SF-12-B.tif]

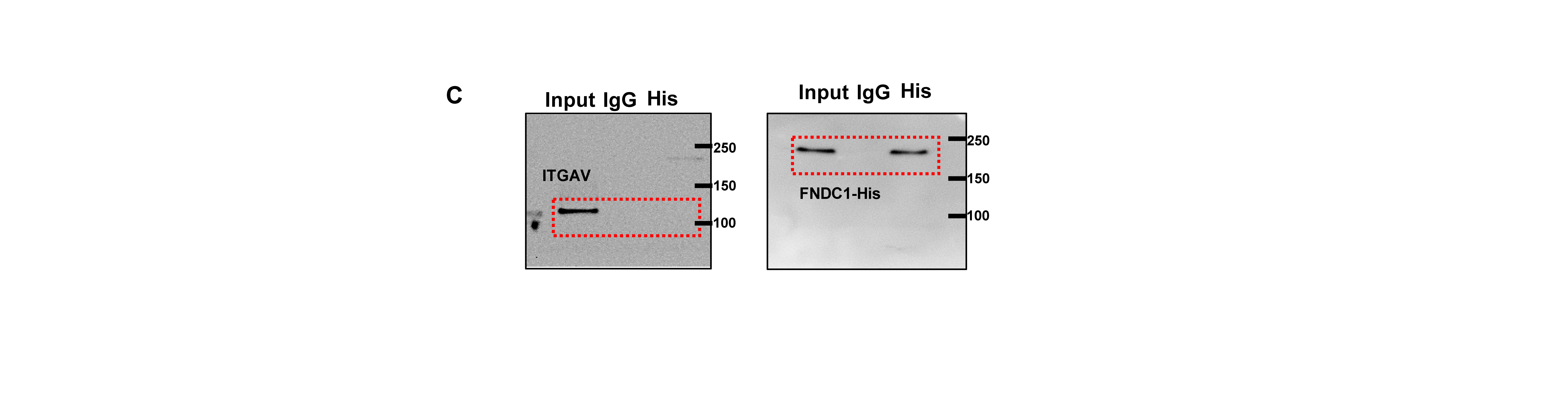

Supplement: Supplementary file 3 — Appendix Figure Source Data [file 44318_2024_285_MOESM3_ESM.zip › Appendix Figure S12/SF 12C/SF-12-C.tif]

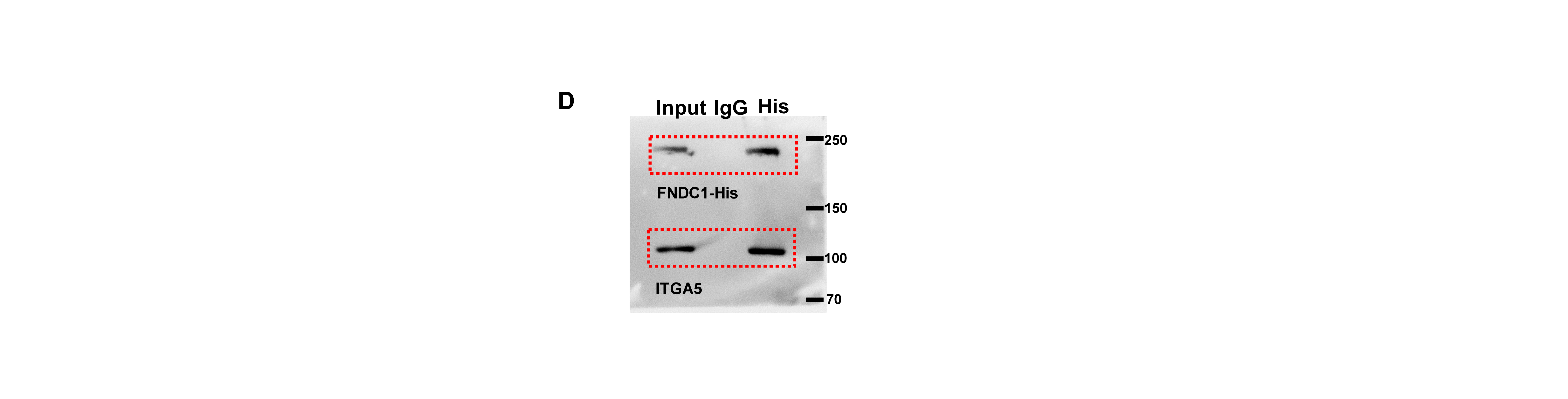

Supplement: Supplementary file 3 — Appendix Figure Source Data [file 44318_2024_285_MOESM3_ESM.zip › Appendix Figure S12/SF 12D/SF-12-D.tif]

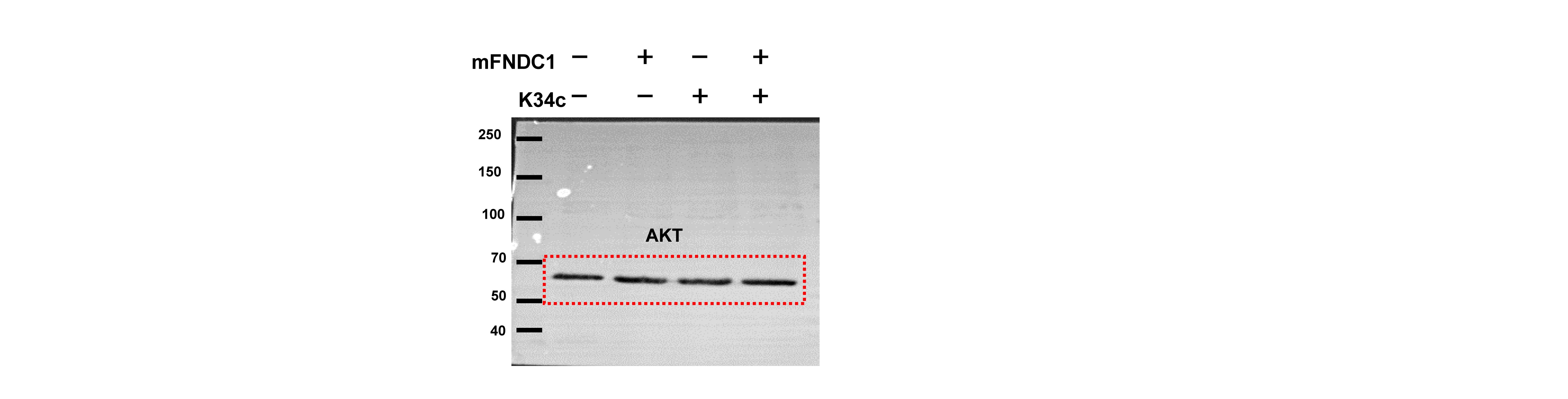

Supplement: Supplementary file 3 — Appendix Figure Source Data [file 44318_2024_285_MOESM3_ESM.zip › Appendix Figure S13/SF 13A/SF-13-A-AKT.tif]

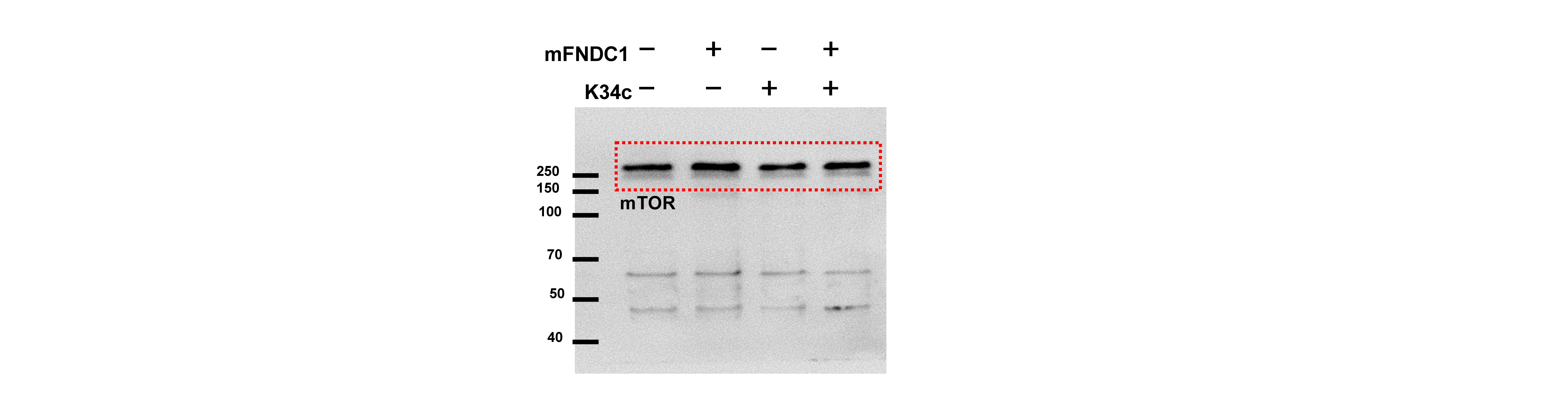

Supplement: Supplementary file 3 — Appendix Figure Source Data [file 44318_2024_285_MOESM3_ESM.zip › Appendix Figure S13/SF 13A/SF-13-A-mTOR.tif]

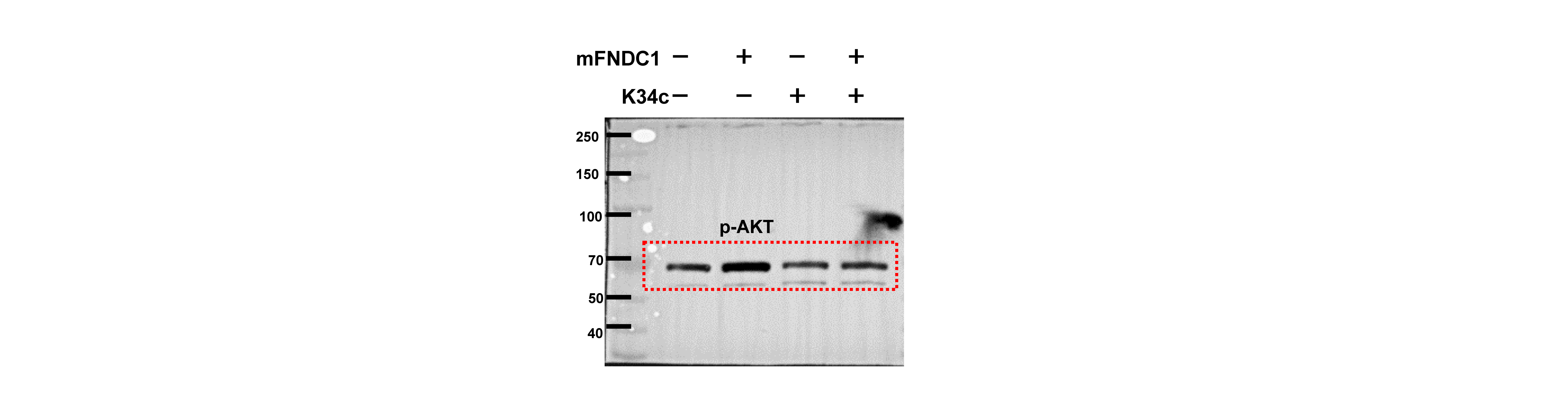

Supplement: Supplementary file 3 — Appendix Figure Source Data [file 44318_2024_285_MOESM3_ESM.zip › Appendix Figure S13/SF 13A/SF-13-A-p-AKT.tif]

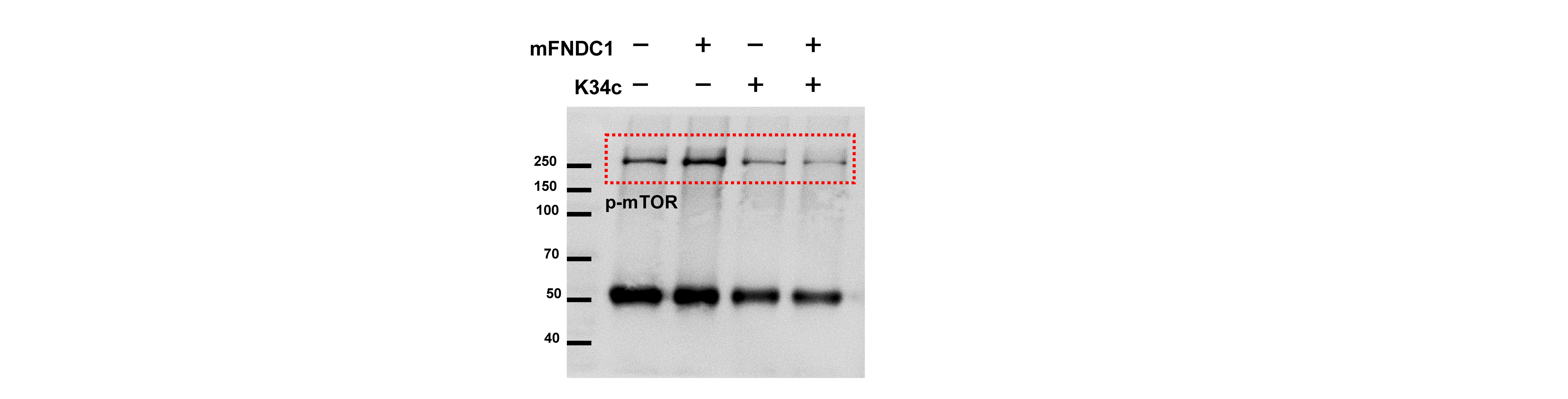

Supplement: Supplementary file 3 — Appendix Figure Source Data [file 44318_2024_285_MOESM3_ESM.zip › Appendix Figure S13/SF 13A/SF-13-A-p-mTOR.tif]

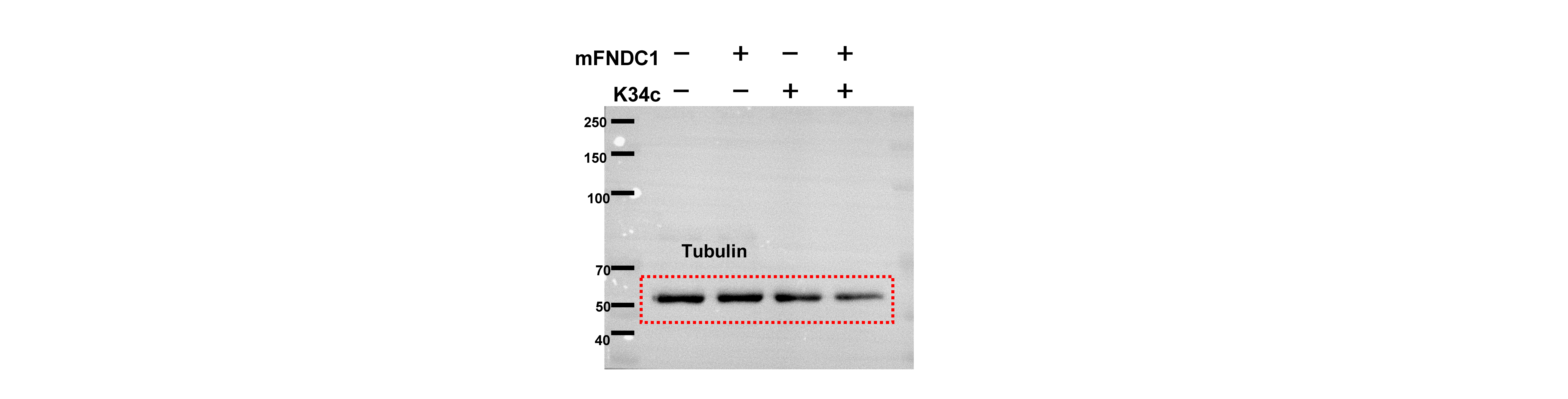

Supplement: Supplementary file 3 — Appendix Figure Source Data [file 44318_2024_285_MOESM3_ESM.zip › Appendix Figure S13/SF 13A/SF-13-A-TUBULIN.tif]

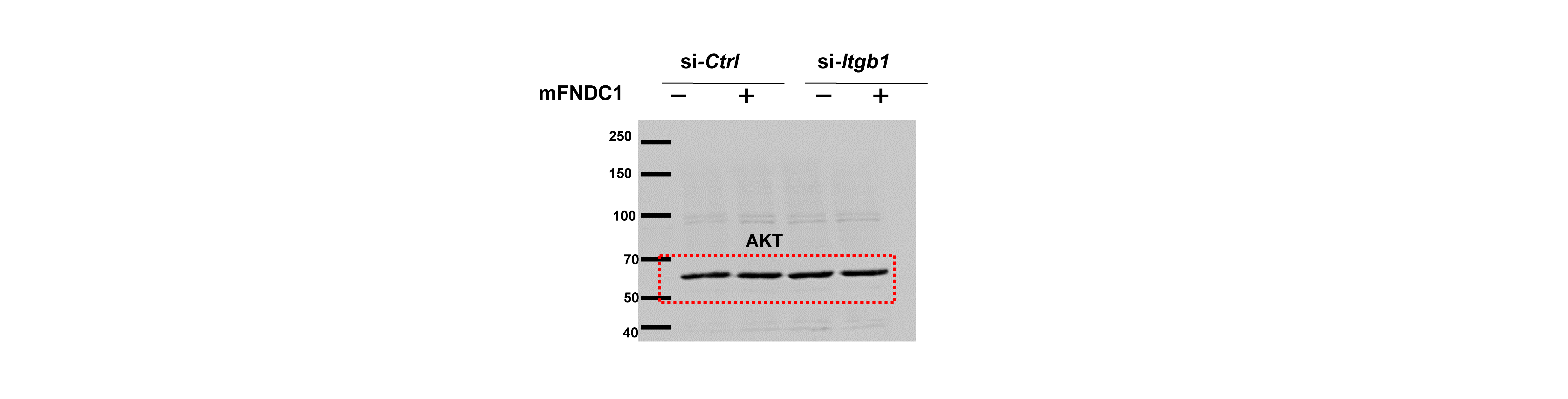

Supplement: Supplementary file 3 — Appendix Figure Source Data [file 44318_2024_285_MOESM3_ESM.zip › Appendix Figure S13/SF 13C/SF-13-C-AKT.tif]

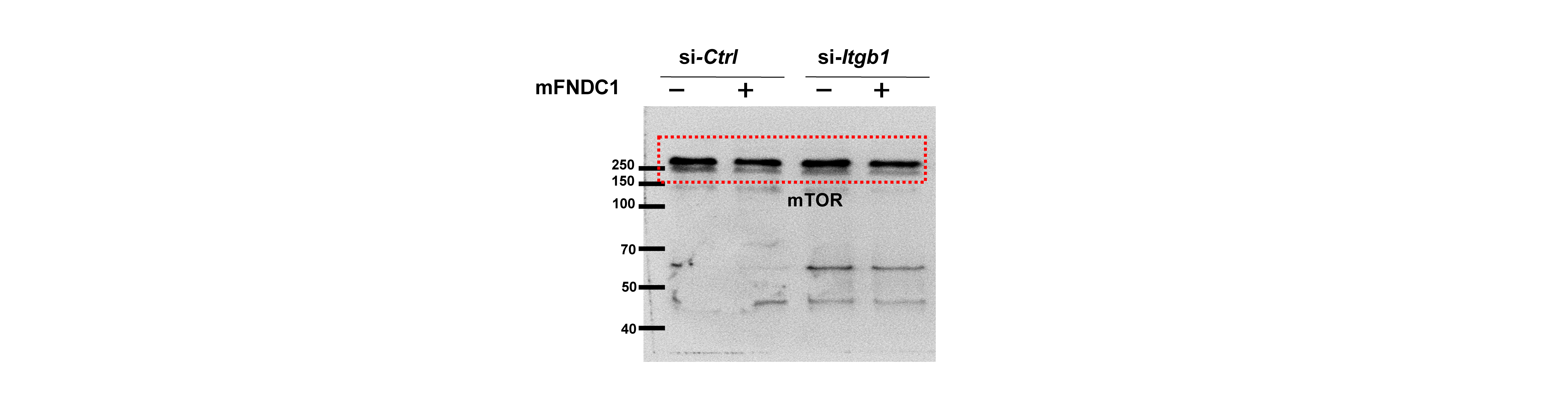

Supplement: Supplementary file 3 — Appendix Figure Source Data [file 44318_2024_285_MOESM3_ESM.zip › Appendix Figure S13/SF 13C/SF-13-C-mTOR.tif]

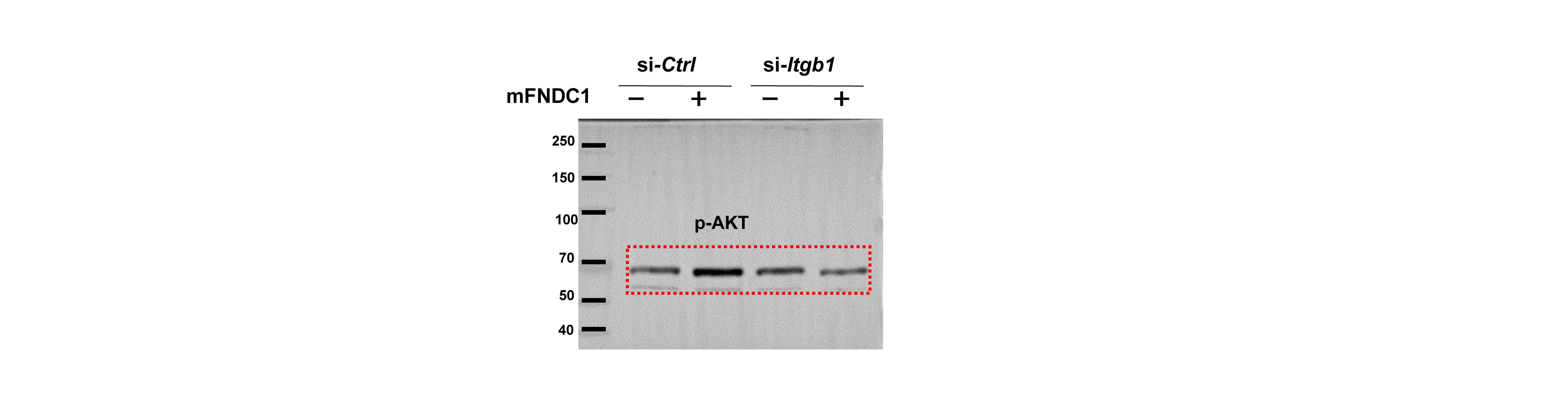

Supplement: Supplementary file 3 — Appendix Figure Source Data [file 44318_2024_285_MOESM3_ESM.zip › Appendix Figure S13/SF 13C/SF-13-C-p-AKT.tif]

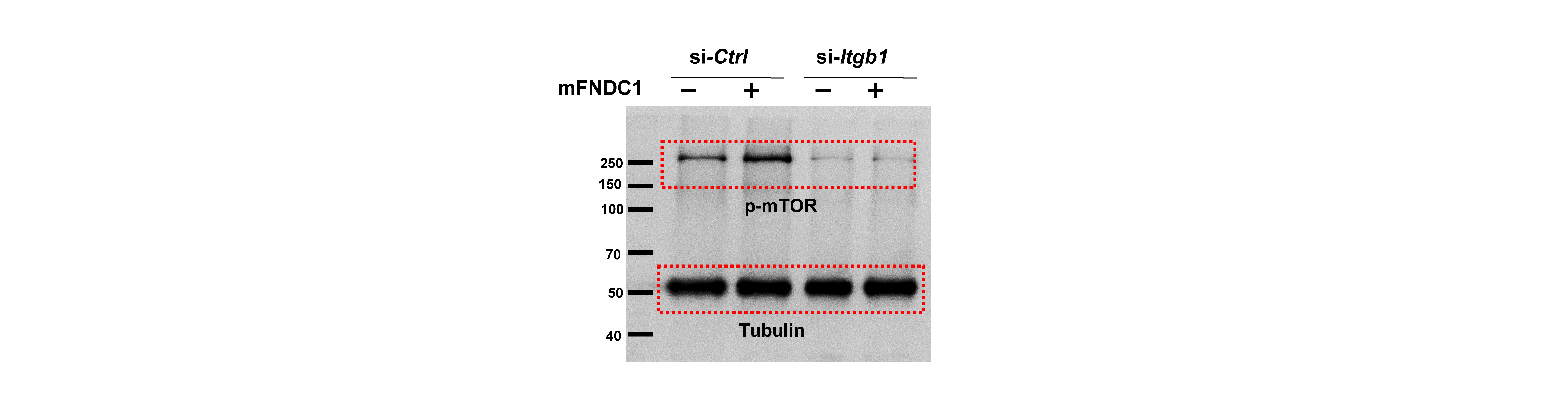

Supplement: Supplementary file 3 — Appendix Figure Source Data [file 44318_2024_285_MOESM3_ESM.zip › Appendix Figure S13/SF 13C/SF-13-C-p-mTOR+TUBULIN.tif]

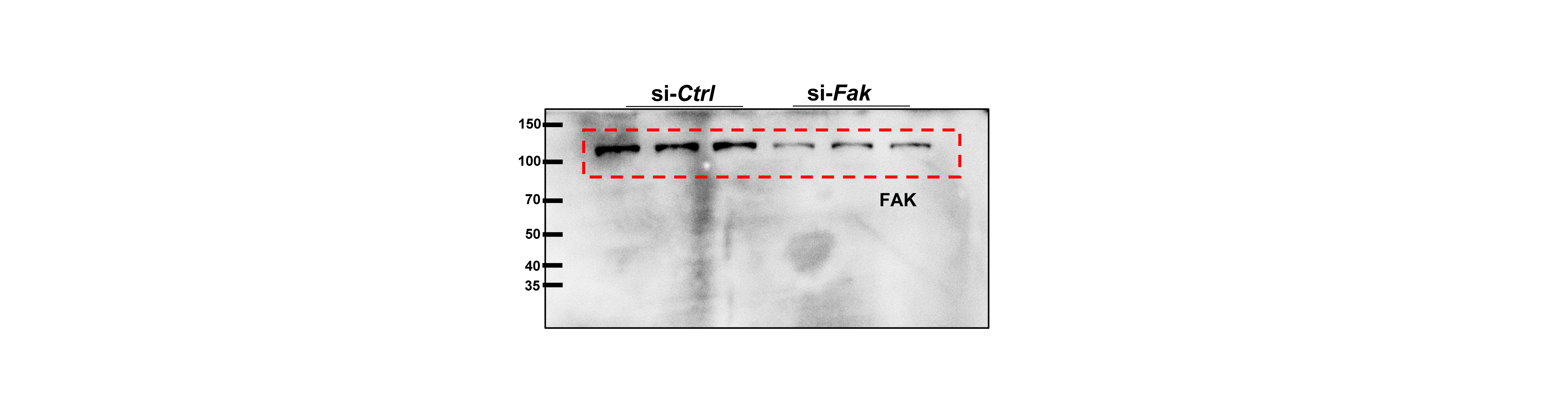

Supplement: Supplementary file 3 — Appendix Figure Source Data [file 44318_2024_285_MOESM3_ESM.zip › Appendix Figure S14/SF 14B/SF-14-B-FAK.tif]

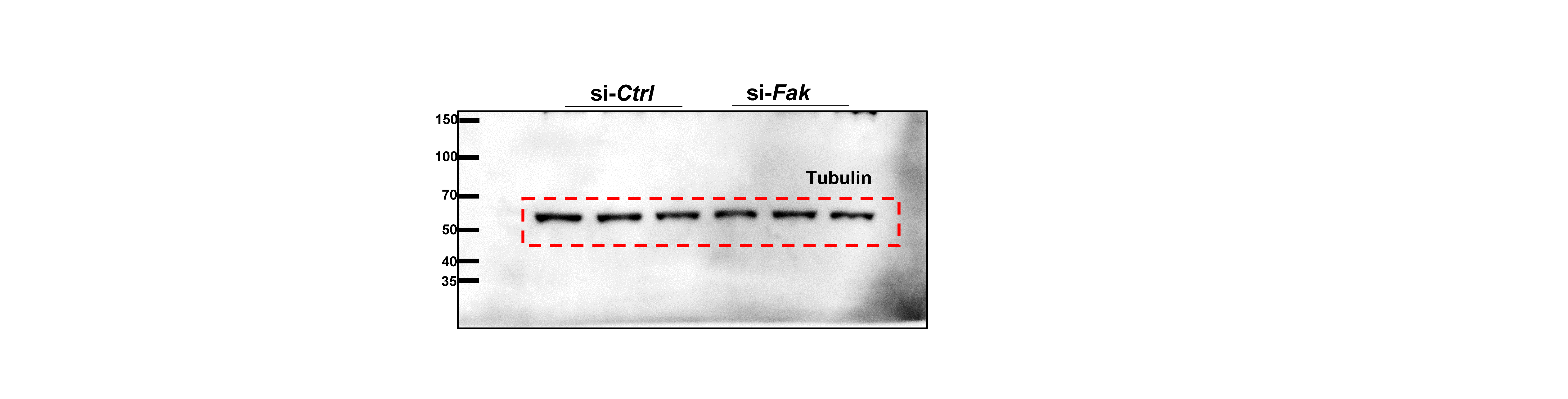

Supplement: Supplementary file 3 — Appendix Figure Source Data [file 44318_2024_285_MOESM3_ESM.zip › Appendix Figure S14/SF 14B/SF-14-B-TUBULIN.tif]

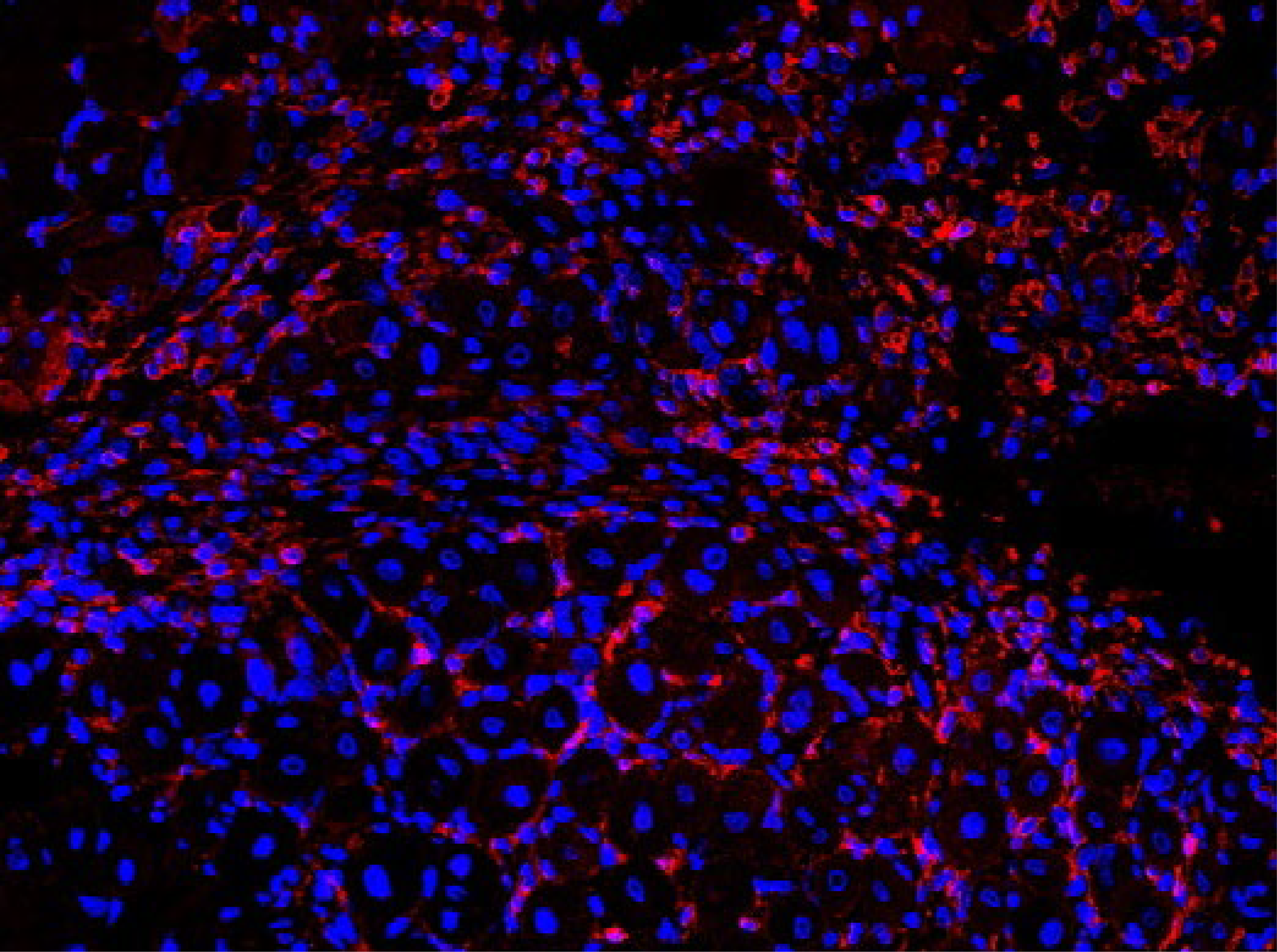

Supplement: Supplementary file 3 — Appendix Figure Source Data [file 44318_2024_285_MOESM3_ESM.zip › Appendix Figure S15/SF 15A/SF15-A-Control.tif]

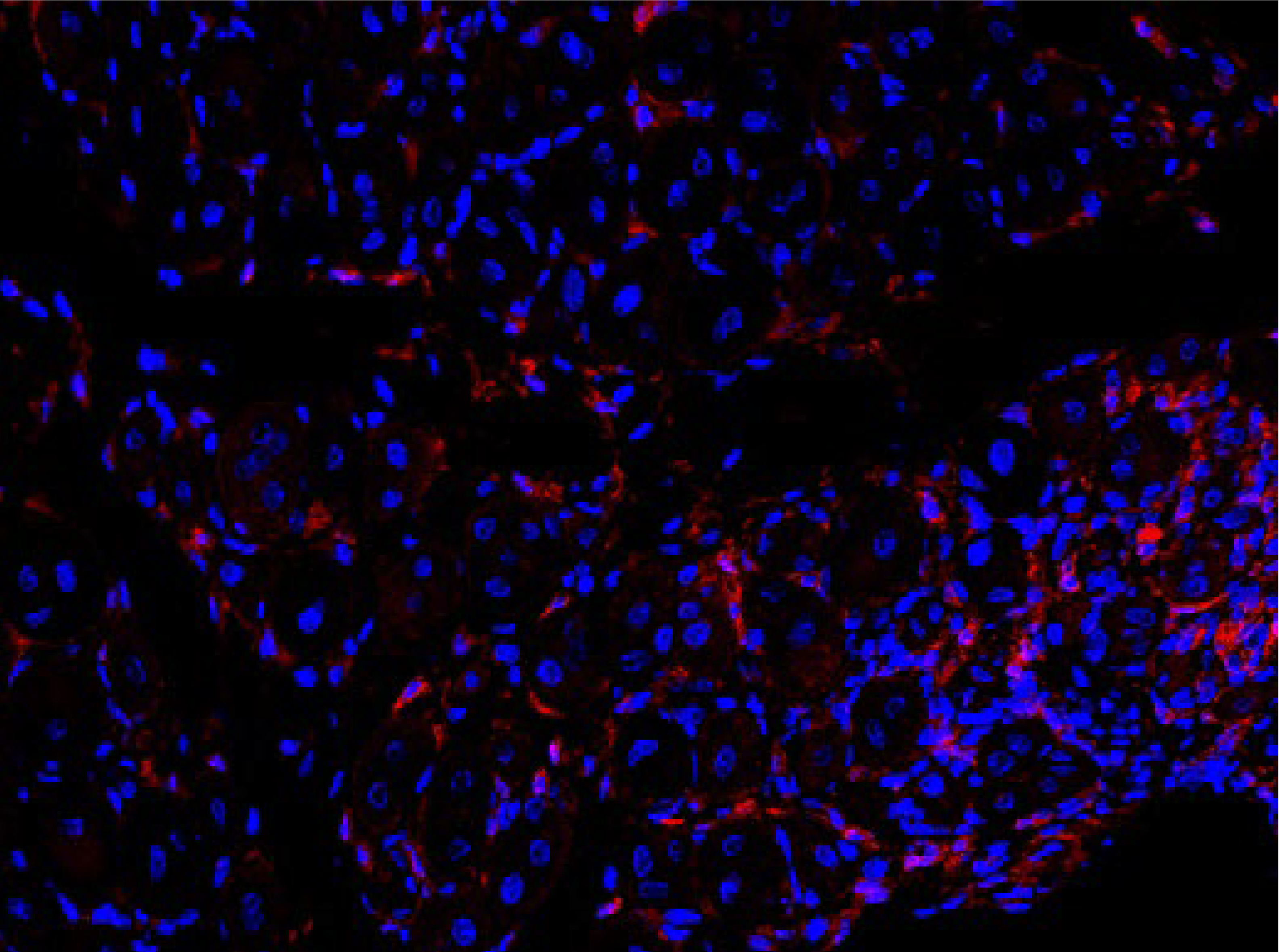

Supplement: Supplementary file 3 — Appendix Figure Source Data [file 44318_2024_285_MOESM3_ESM.zip › Appendix Figure S15/SF 15A/SF15-A-mFNDC1.tif]

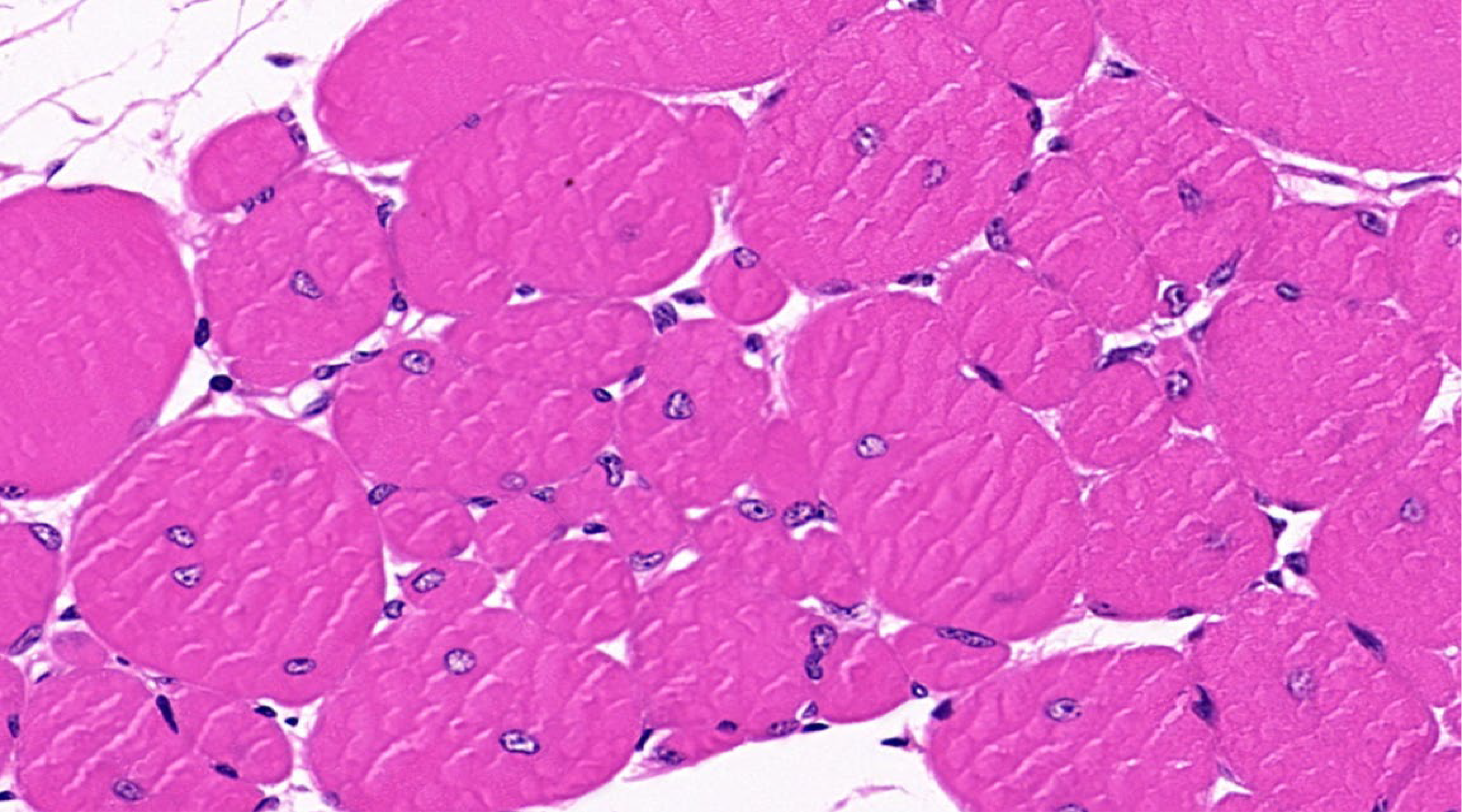

Supplement: Supplementary file 3 — Appendix Figure Source Data [file 44318_2024_285_MOESM3_ESM.zip › Appendix Figure S16/SF 16A/SF16-A-AAV-scar-Day14.tif]

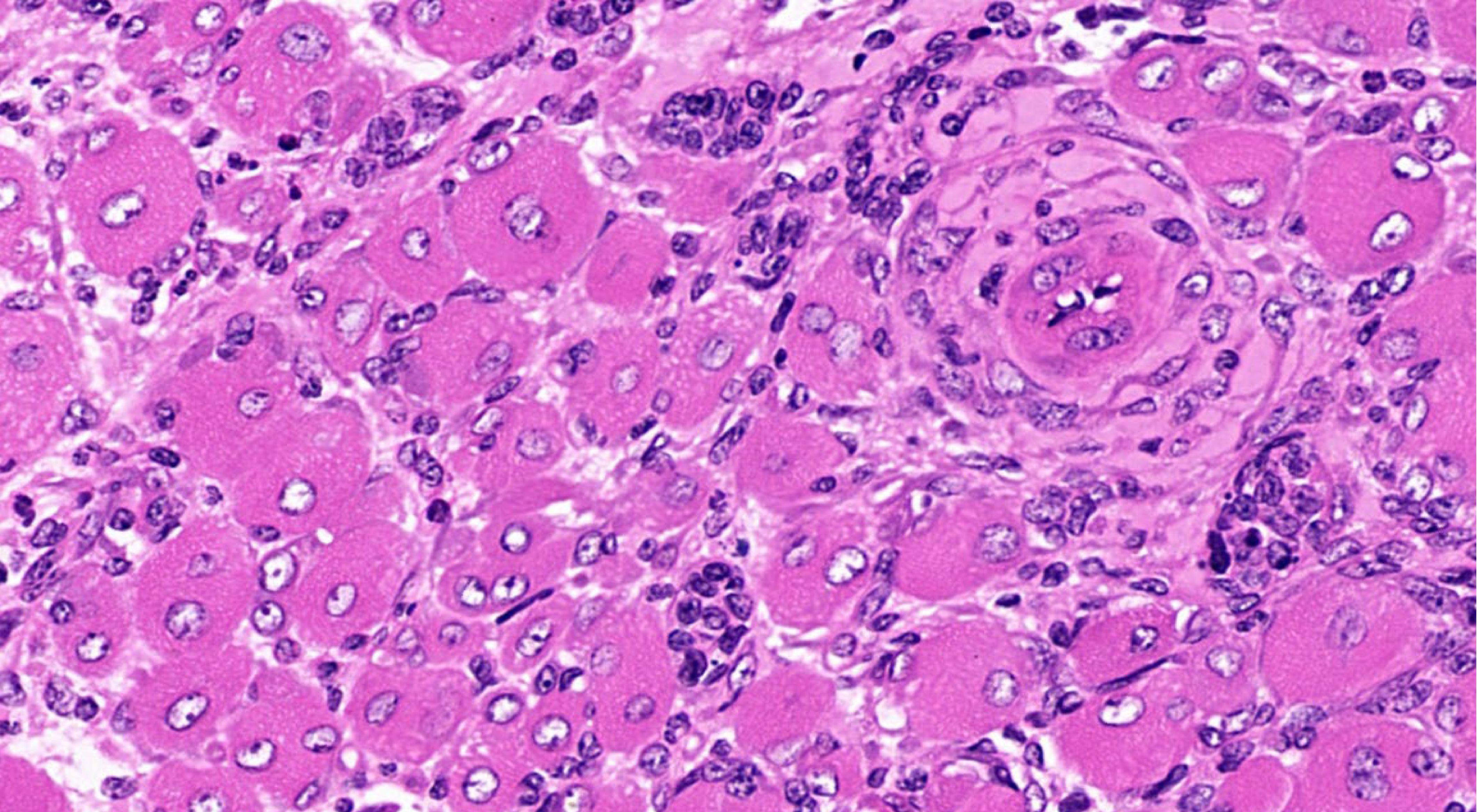

Supplement: Supplementary file 3 — Appendix Figure Source Data [file 44318_2024_285_MOESM3_ESM.zip › Appendix Figure S16/SF 16A/SF16-A-AAV-scar-Day5.tif]

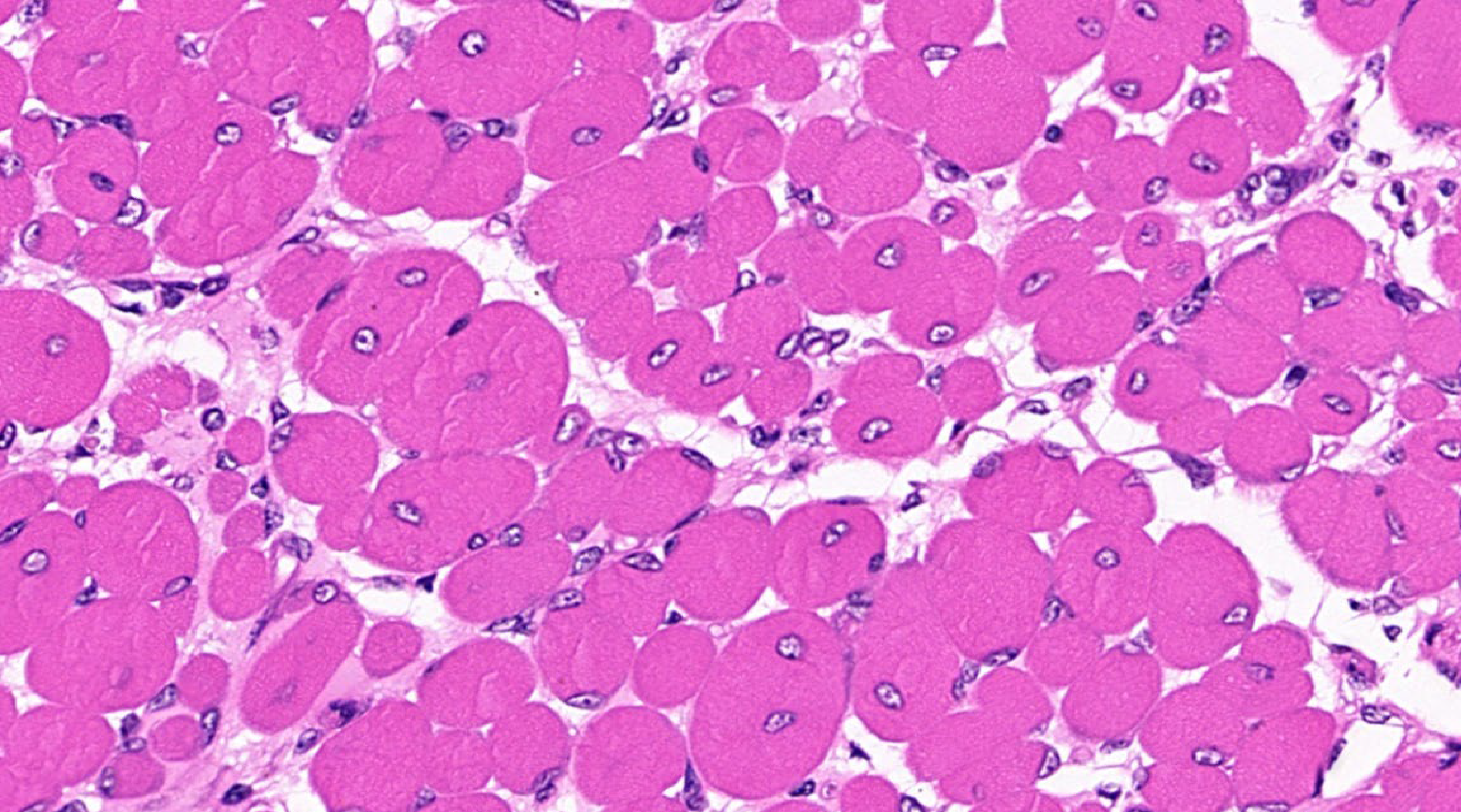

Supplement: Supplementary file 3 — Appendix Figure Source Data [file 44318_2024_285_MOESM3_ESM.zip › Appendix Figure S16/SF 16A/SF16-A-shFndc1-Day14.tif]

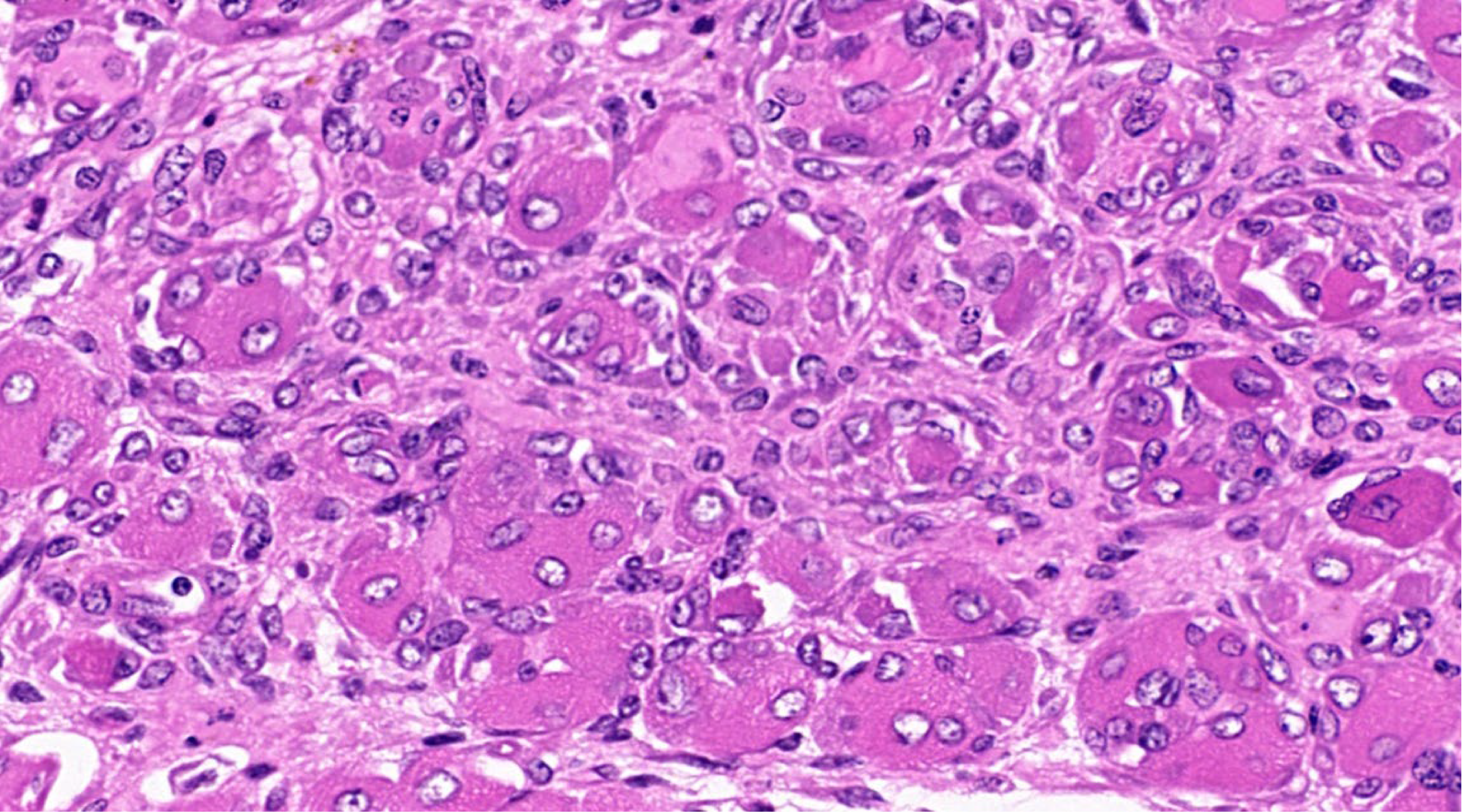

Supplement: Supplementary file 3 — Appendix Figure Source Data [file 44318_2024_285_MOESM3_ESM.zip › Appendix Figure S16/SF 16A/SF16-A-shFndc1-Day5.tif]

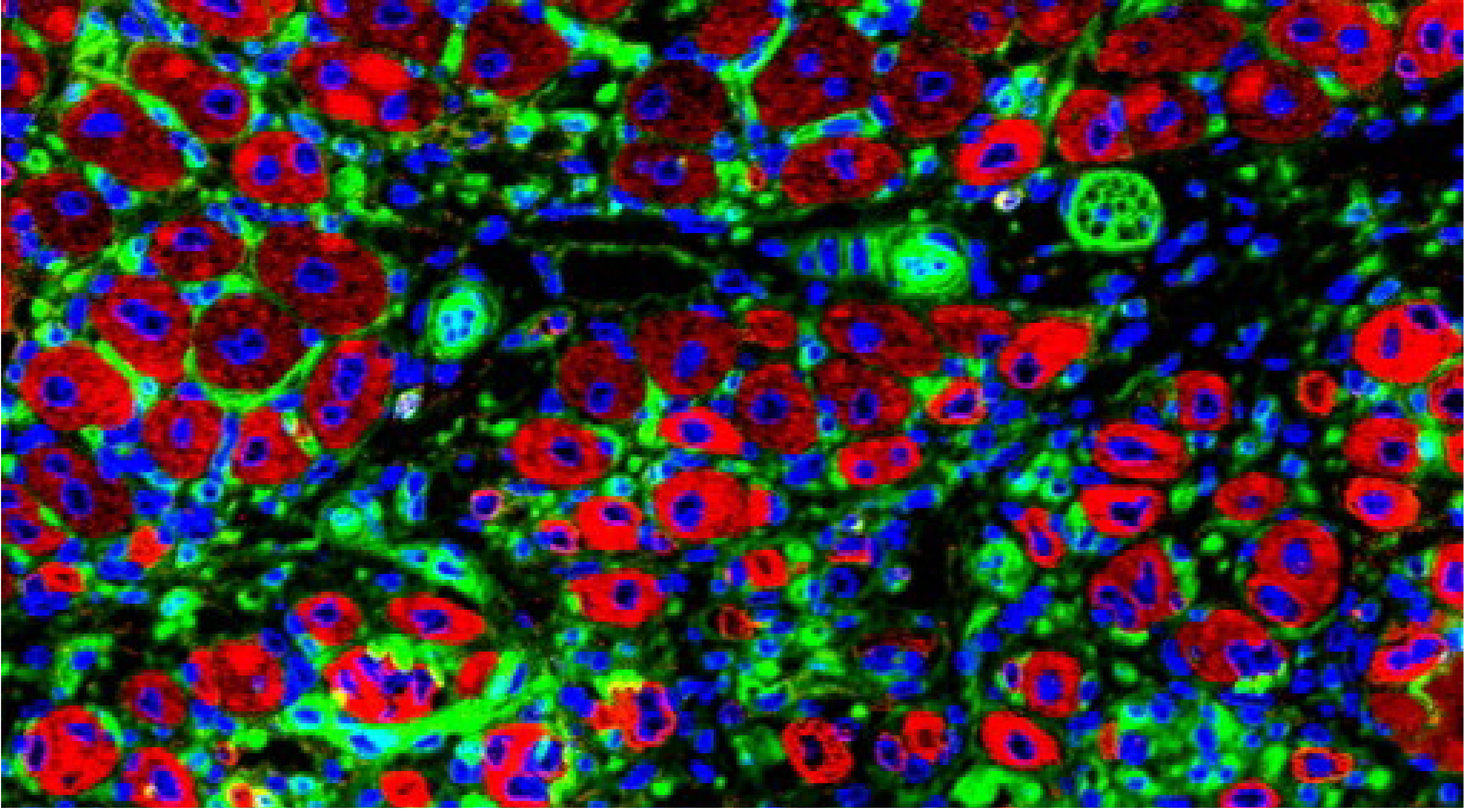

Supplement: Supplementary file 3 — Appendix Figure Source Data [file 44318_2024_285_MOESM3_ESM.zip › Appendix Figure S16/SF 16B/SF16-B-AAV-scar.tif]

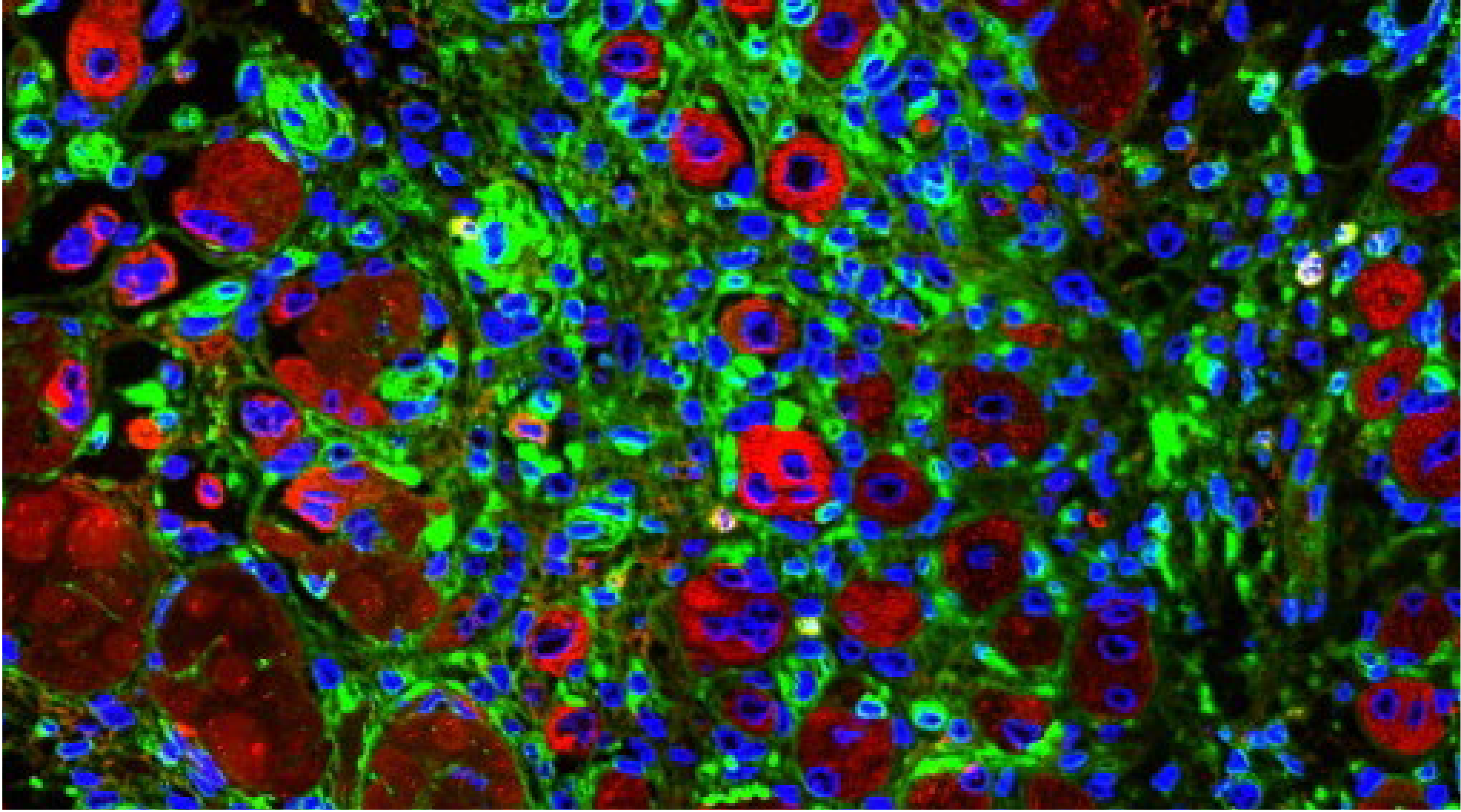

Supplement: Supplementary file 3 — Appendix Figure Source Data [file 44318_2024_285_MOESM3_ESM.zip › Appendix Figure S16/SF 16B/SF16-B-shFndc1.tif]

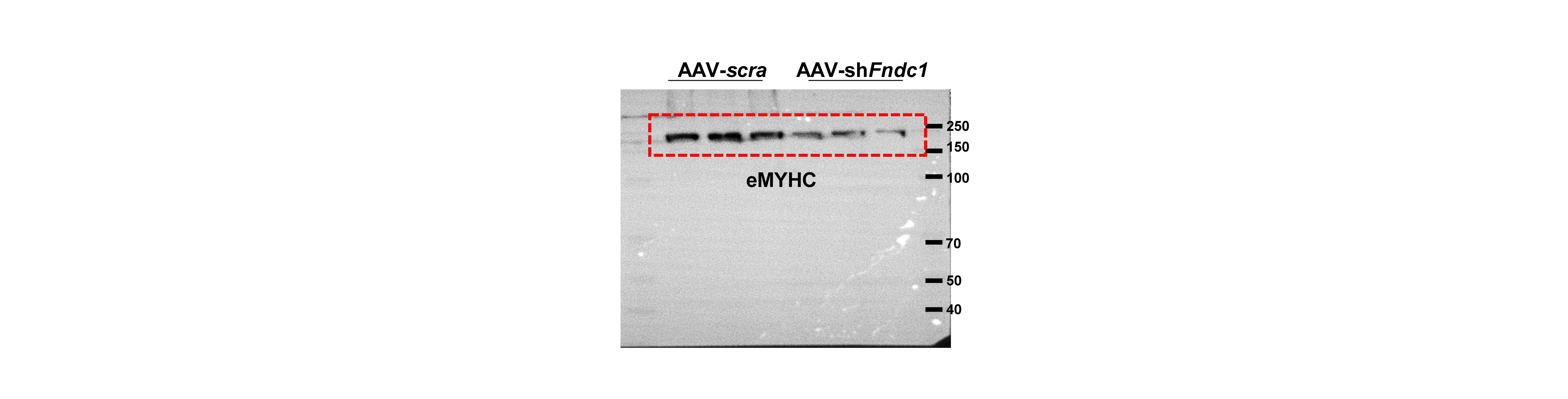

Supplement: Supplementary file 3 — Appendix Figure Source Data [file 44318_2024_285_MOESM3_ESM.zip › Appendix Figure S16/SF 16F/SF-16-F-eMYHC.tif]

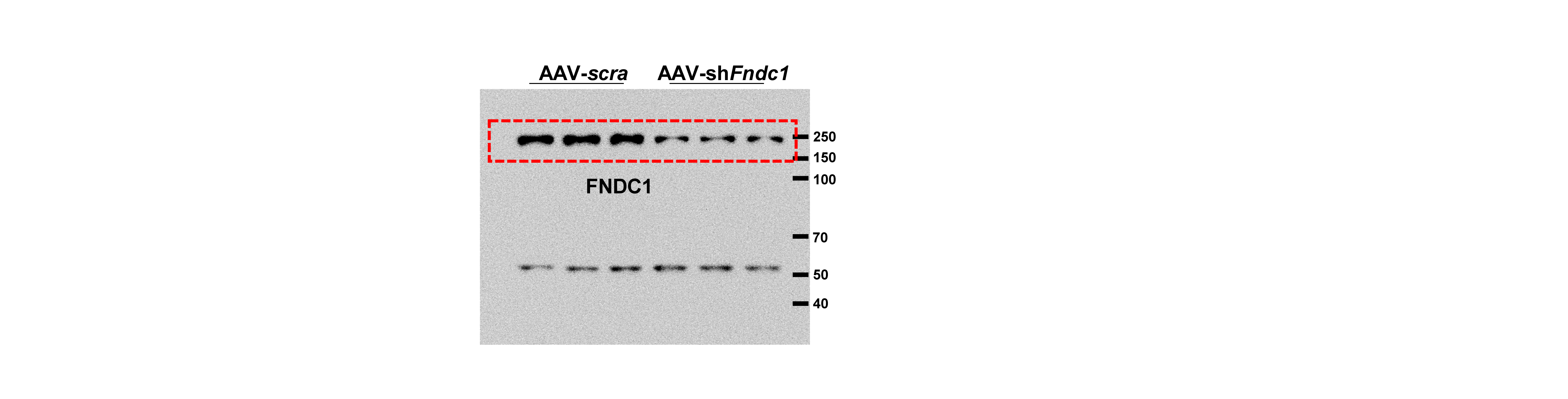

Supplement: Supplementary file 3 — Appendix Figure Source Data [file 44318_2024_285_MOESM3_ESM.zip › Appendix Figure S16/SF 16F/SF-16-F-FNDC1.tif]

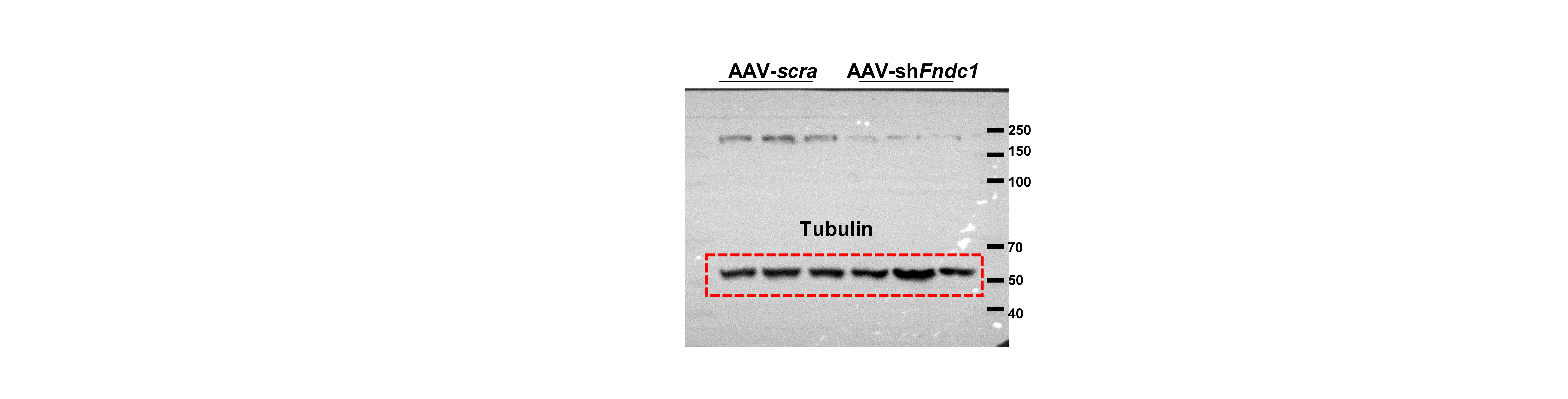

Supplement: Supplementary file 3 — Appendix Figure Source Data [file 44318_2024_285_MOESM3_ESM.zip › Appendix Figure S16/SF 16F/SF-16-F-TUBULIN.tif]

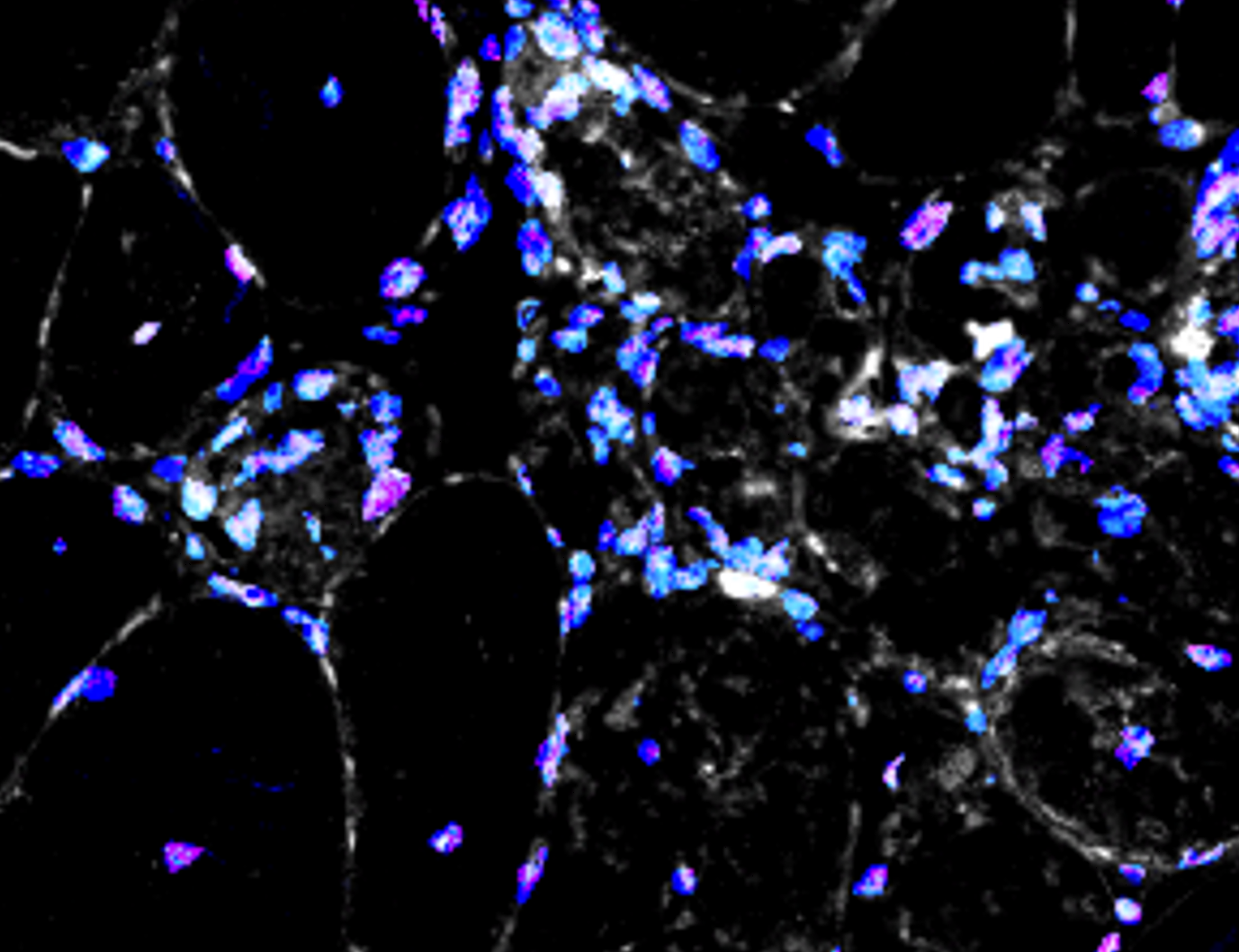

Supplement: Supplementary file 3 — Appendix Figure Source Data [file 44318_2024_285_MOESM3_ESM.zip › Appendix Figure S17/SF 17A/SF-17-A-AAV-scar-Day3-merger.tif]

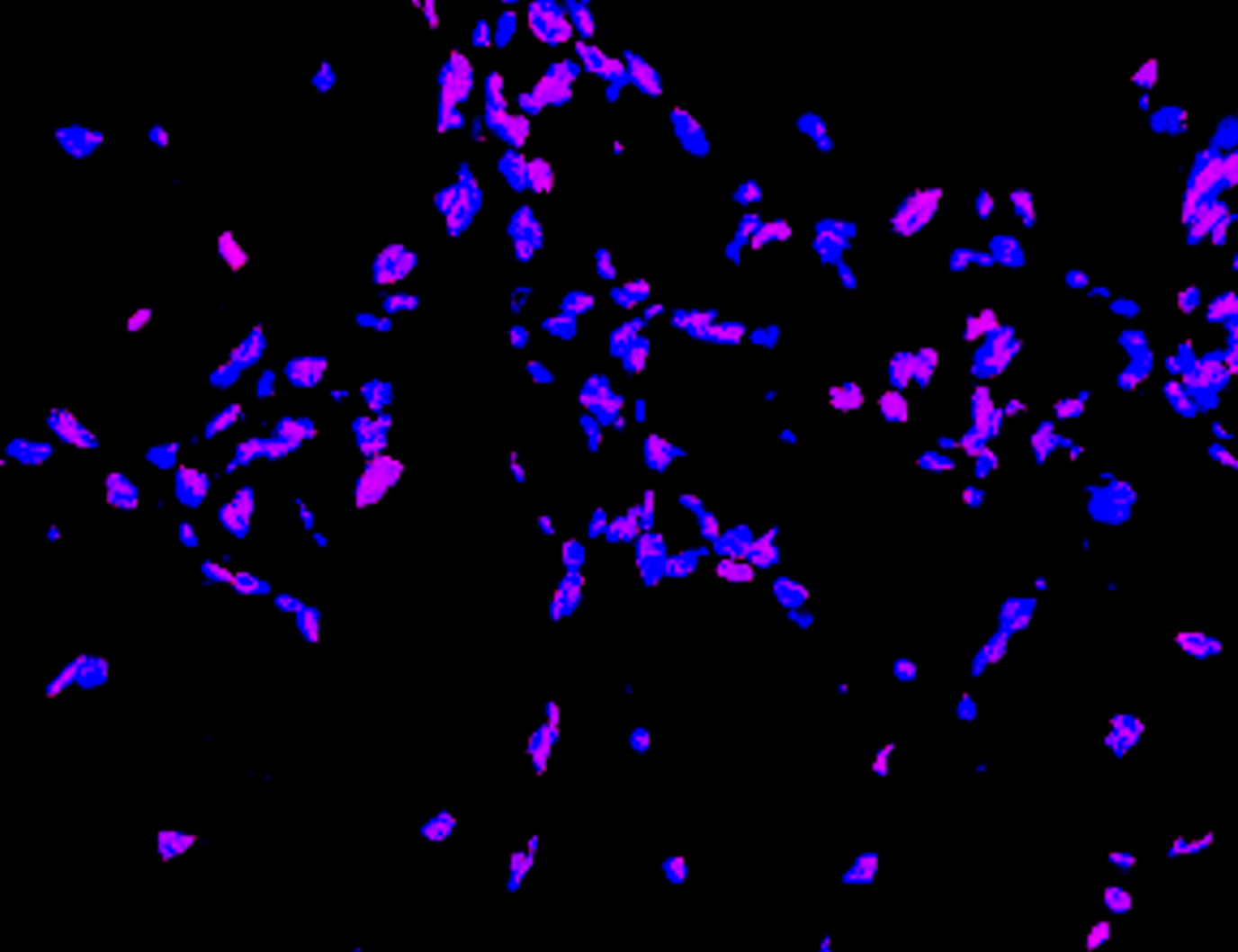

Supplement: Supplementary file 3 — Appendix Figure Source Data [file 44318_2024_285_MOESM3_ESM.zip › Appendix Figure S17/SF 17A/SF-17-A-AAV-scar-Day3-MYOD.tif]

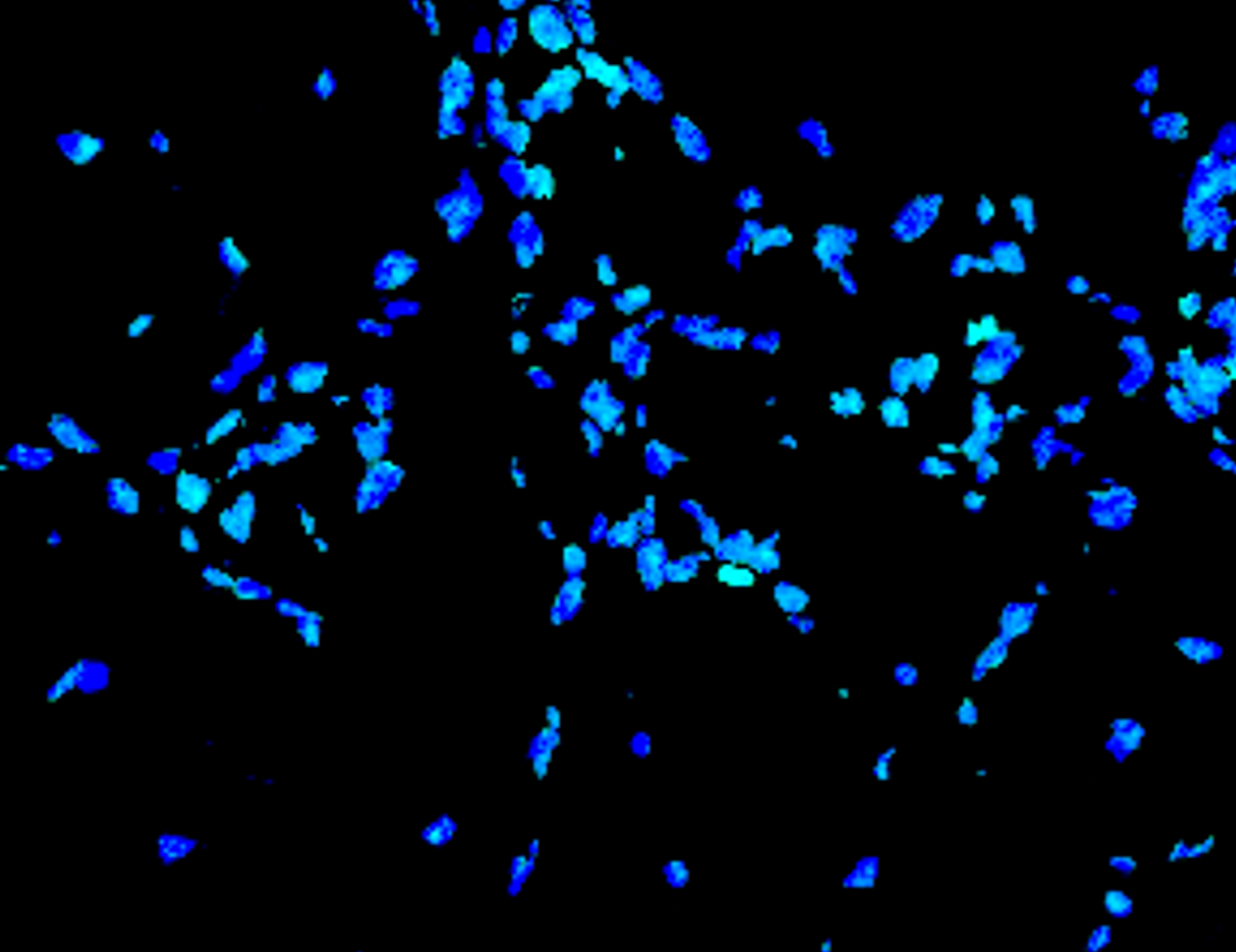

Supplement: Supplementary file 3 — Appendix Figure Source Data [file 44318_2024_285_MOESM3_ESM.zip › Appendix Figure S17/SF 17A/SF-17-A-AAV-scar-Day3-PAX7.tif]

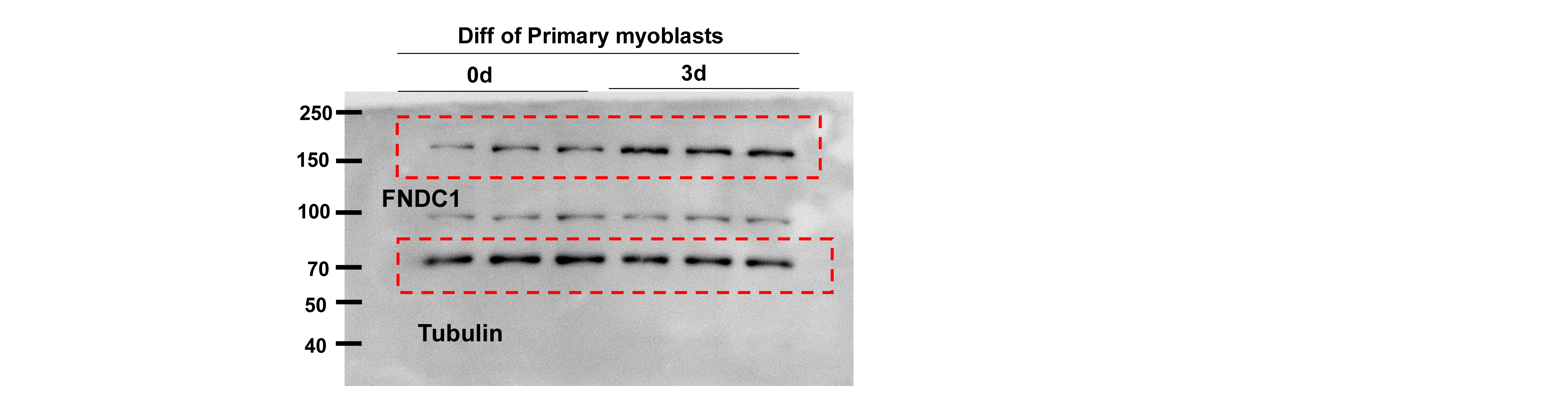

Supplement: Supplementary file 4 — Source data Fig. 1 [file 44318_2024_285_MOESM4_ESM.zip › Fig 1/Fig 1D/1D-FNDC1+TUBULIN.tif]

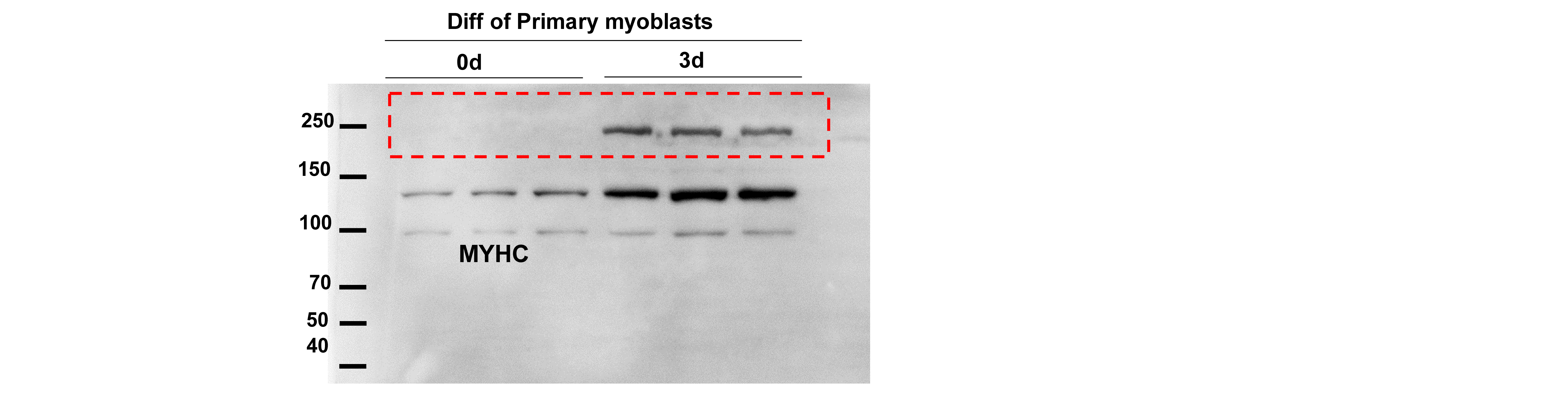

Supplement: Supplementary file 4 — Source data Fig. 1 [file 44318_2024_285_MOESM4_ESM.zip › Fig 1/Fig 1D/1D-MYHC.tif]

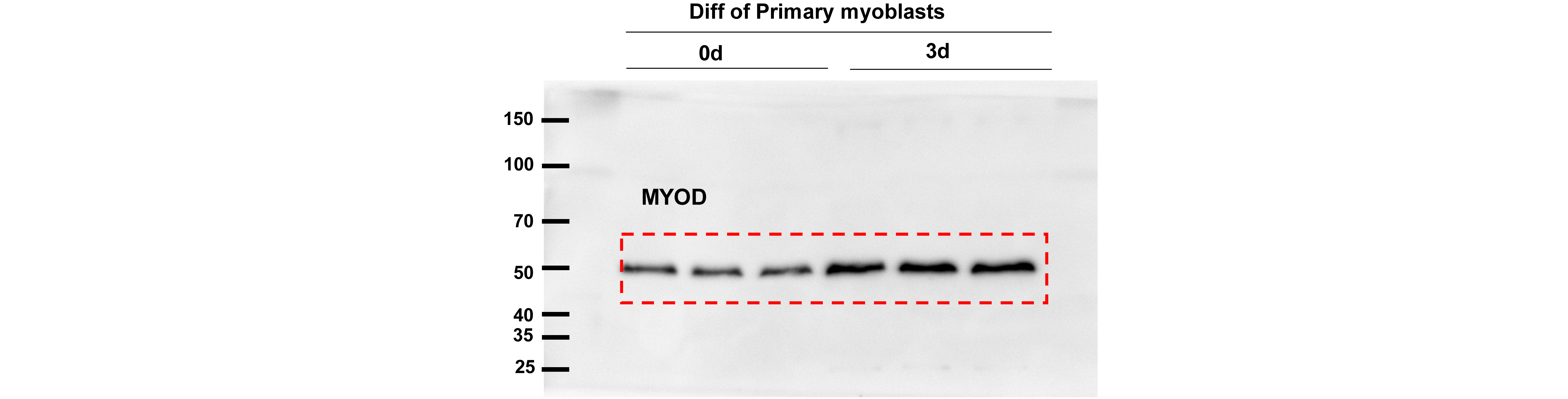

Supplement: Supplementary file 4 — Source data Fig. 1 [file 44318_2024_285_MOESM4_ESM.zip › Fig 1/Fig 1D/1D-MYOD.tif]

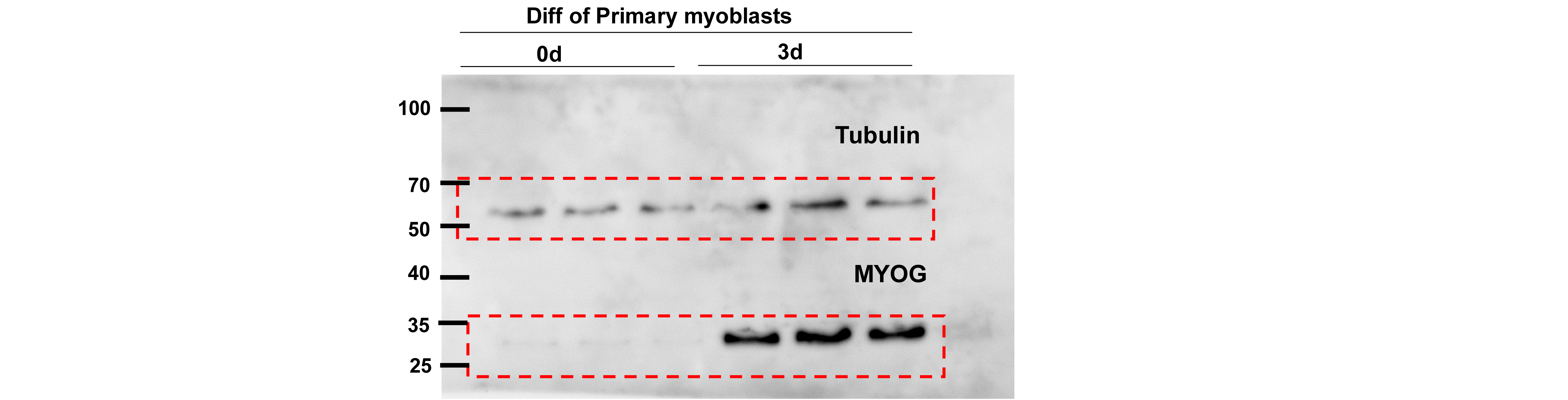

Supplement: Supplementary file 4 — Source data Fig. 1 [file 44318_2024_285_MOESM4_ESM.zip › Fig 1/Fig 1D/1D-MYOG.tif]

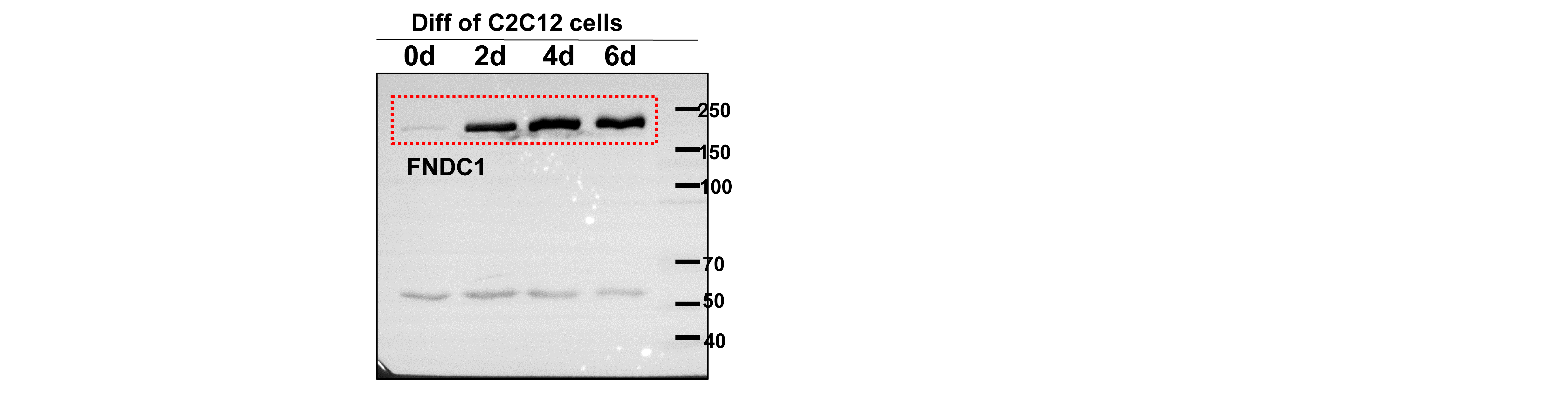

Supplement: Supplementary file 4 — Source data Fig. 1 [file 44318_2024_285_MOESM4_ESM.zip › Fig 1/Fig 1E/1-E-FNDC1.tif]

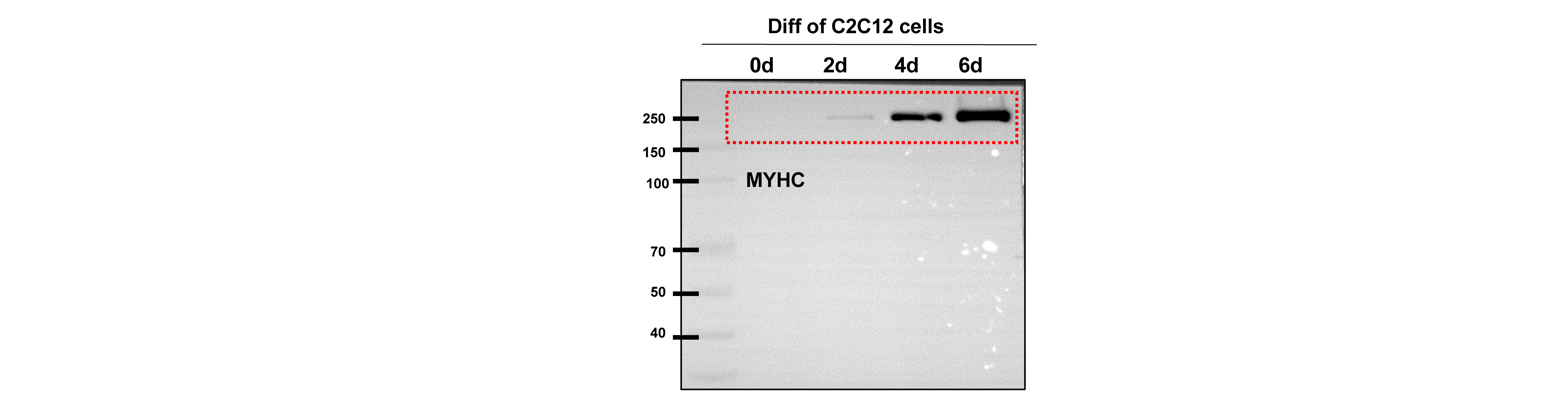

Supplement: Supplementary file 4 — Source data Fig. 1 [file 44318_2024_285_MOESM4_ESM.zip › Fig 1/Fig 1E/1-E-MYHC.tif]

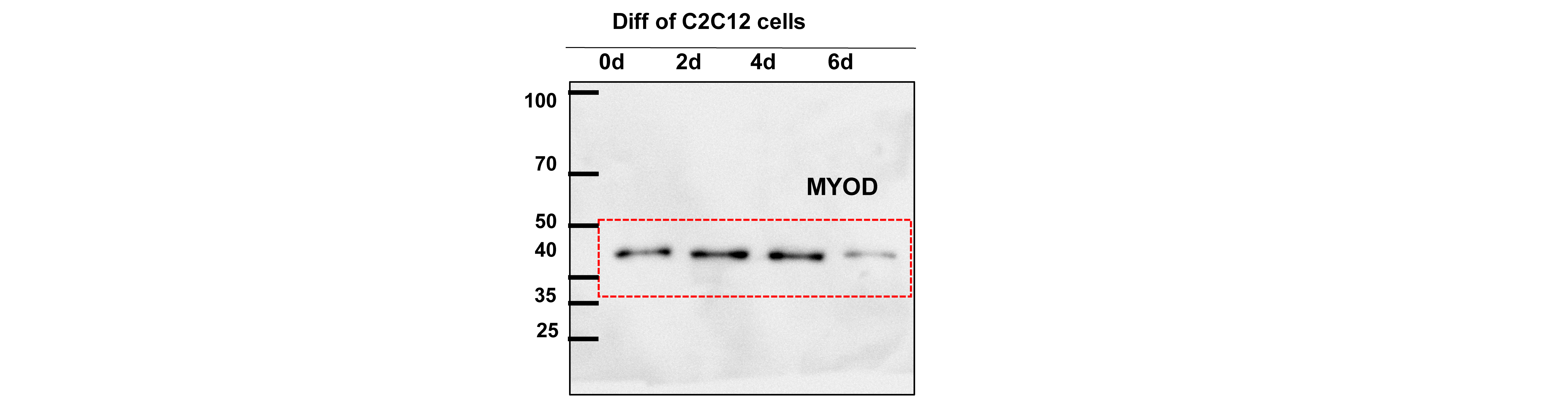

Supplement: Supplementary file 4 — Source data Fig. 1 [file 44318_2024_285_MOESM4_ESM.zip › Fig 1/Fig 1E/1-E-MYOD.tif]

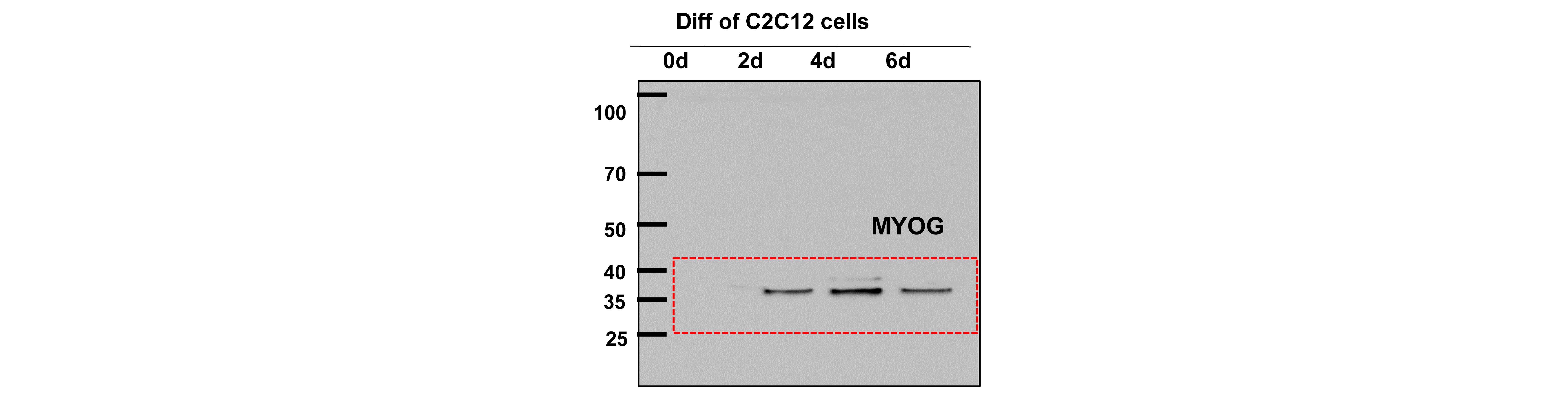

Supplement: Supplementary file 4 — Source data Fig. 1 [file 44318_2024_285_MOESM4_ESM.zip › Fig 1/Fig 1E/1-E-MYOG.tif]

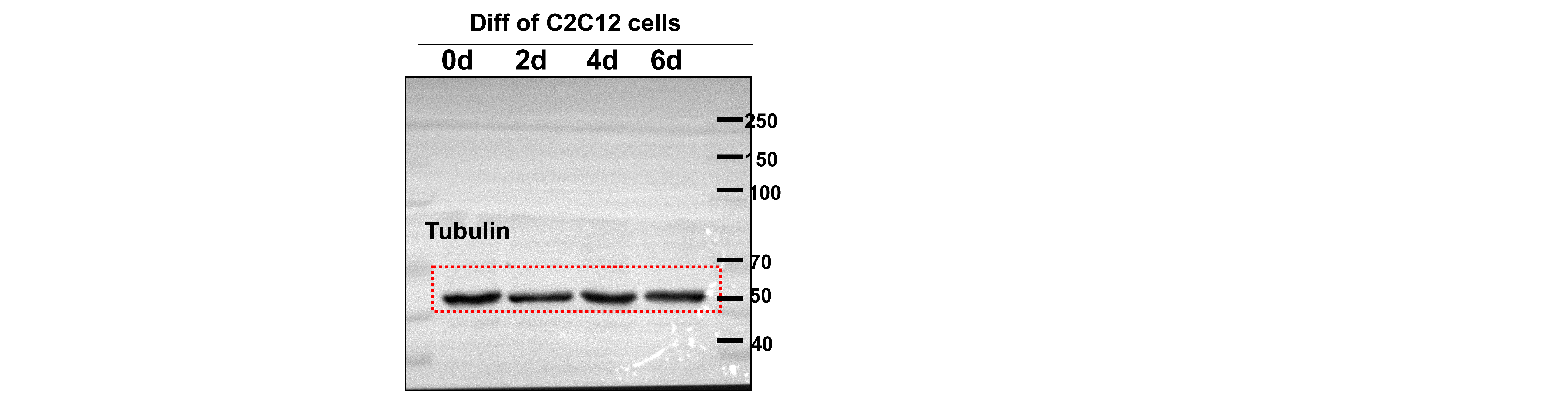

Supplement: Supplementary file 4 — Source data Fig. 1 [file 44318_2024_285_MOESM4_ESM.zip › Fig 1/Fig 1E/1-E-TUBULIN.tif]

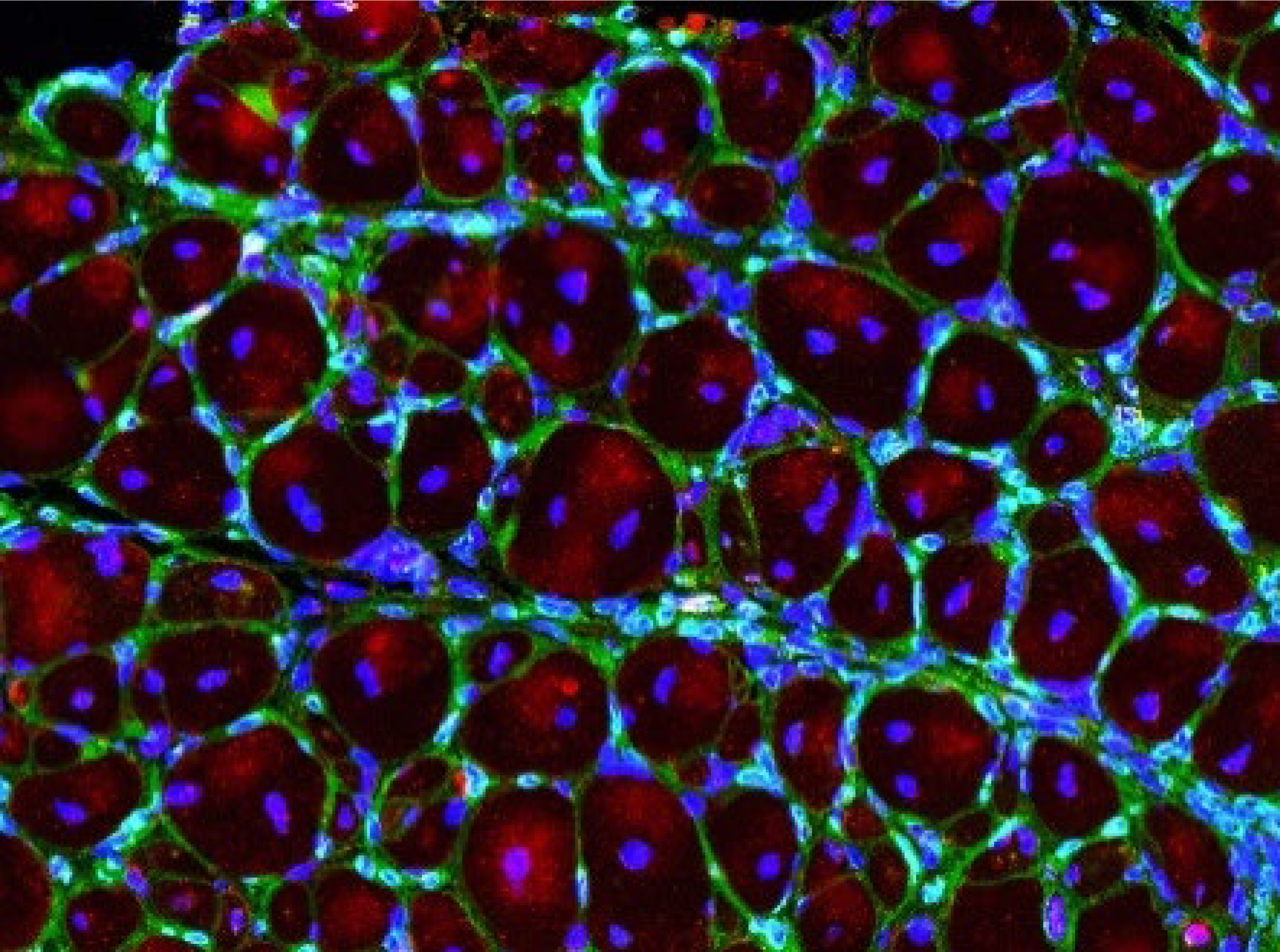

Supplement: Supplementary file 4 — Source data Fig. 1 [file 44318_2024_285_MOESM4_ESM.zip › Fig 1/Fig 1G/1-G-CTX-treated 5d.tif]

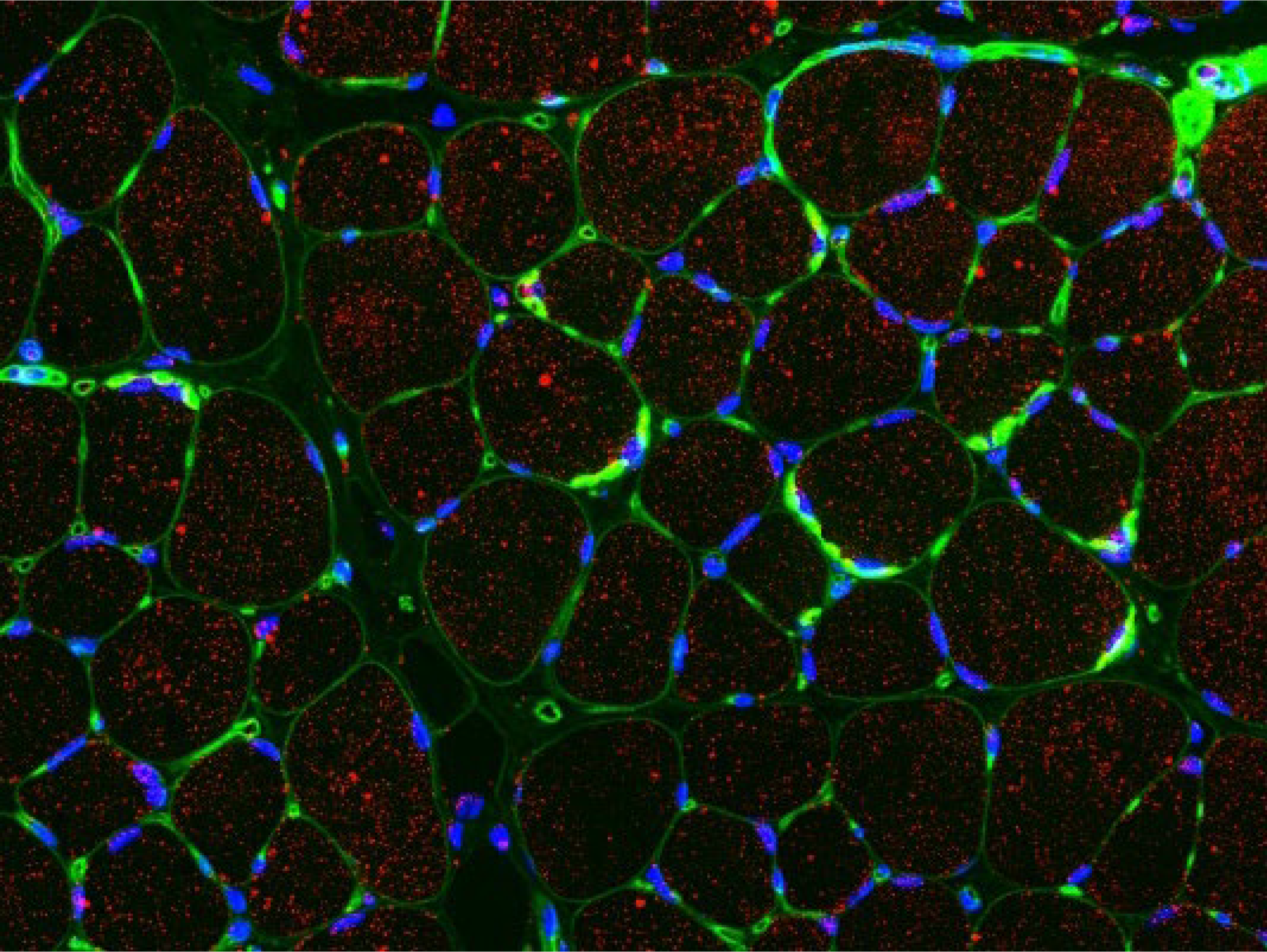

Supplement: Supplementary file 4 — Source data Fig. 1 [file 44318_2024_285_MOESM4_ESM.zip › Fig 1/Fig 1G/1-G-Untreated.tif]

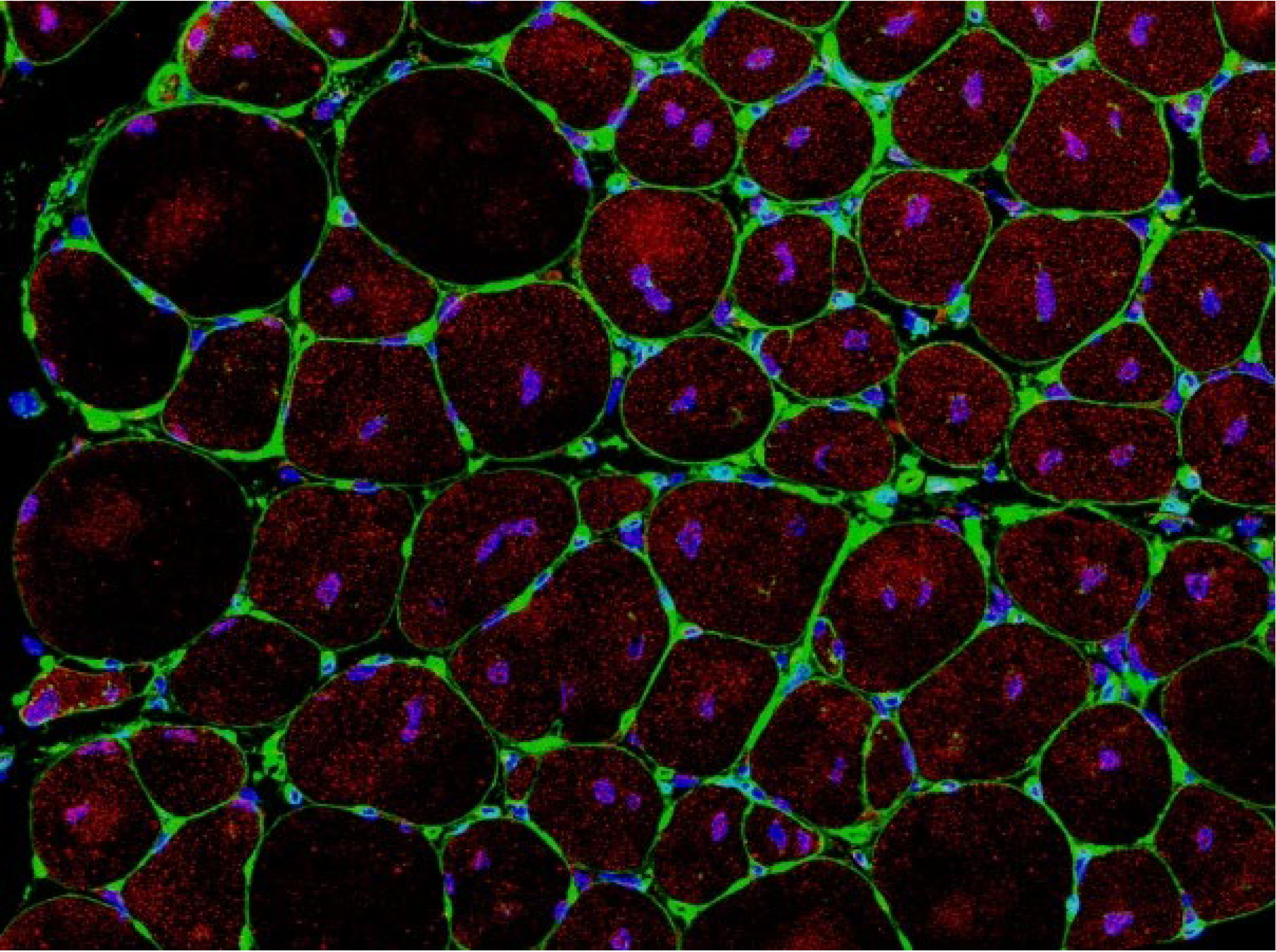

Supplement: Supplementary file 4 — Source data Fig. 1 [file 44318_2024_285_MOESM4_ESM.zip › Fig 1/Fig 1I/1-I-MDX .tif]

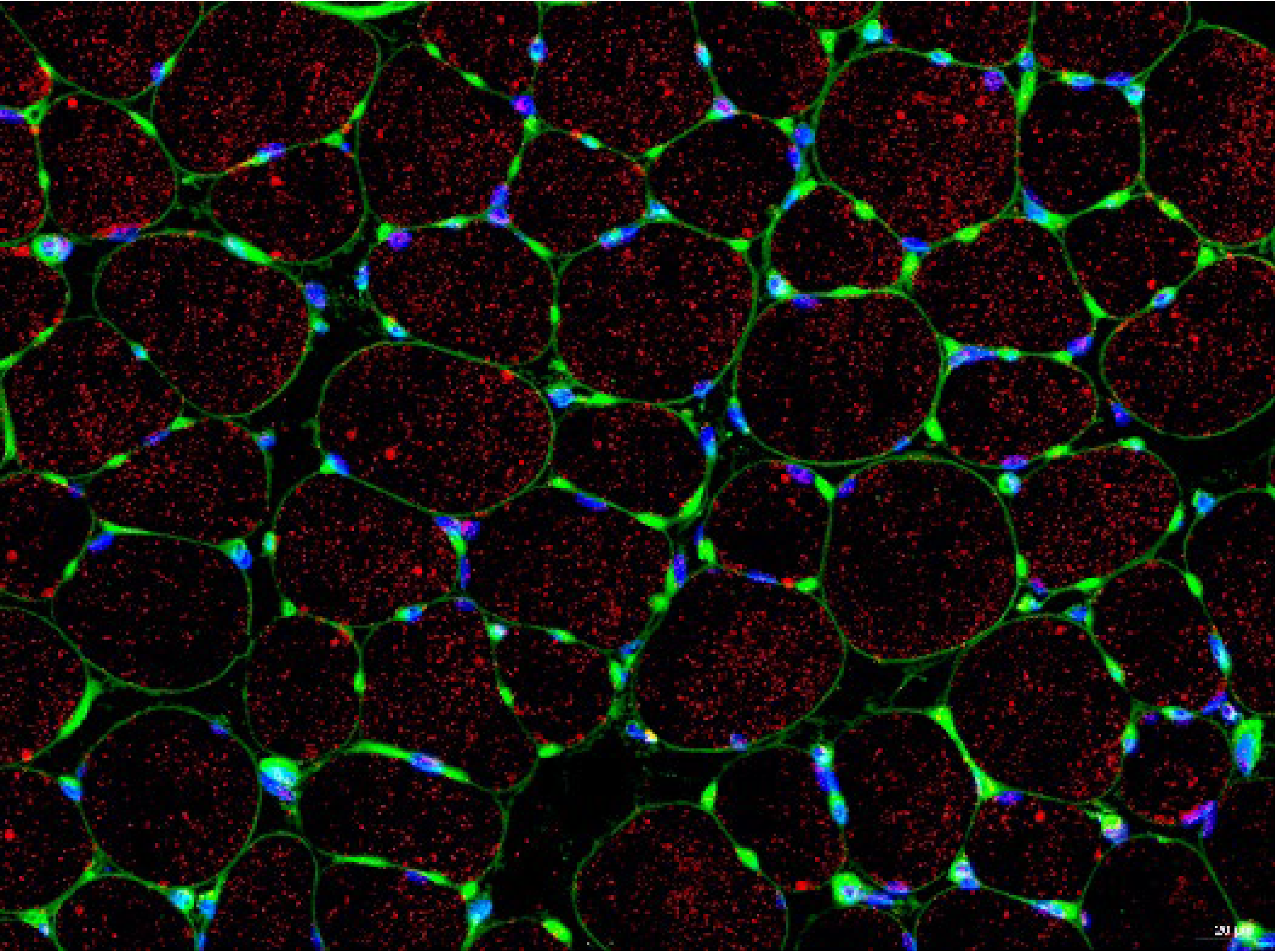

Supplement: Supplementary file 4 — Source data Fig. 1 [file 44318_2024_285_MOESM4_ESM.zip › Fig 1/Fig 1I/1-I-WT .tif]

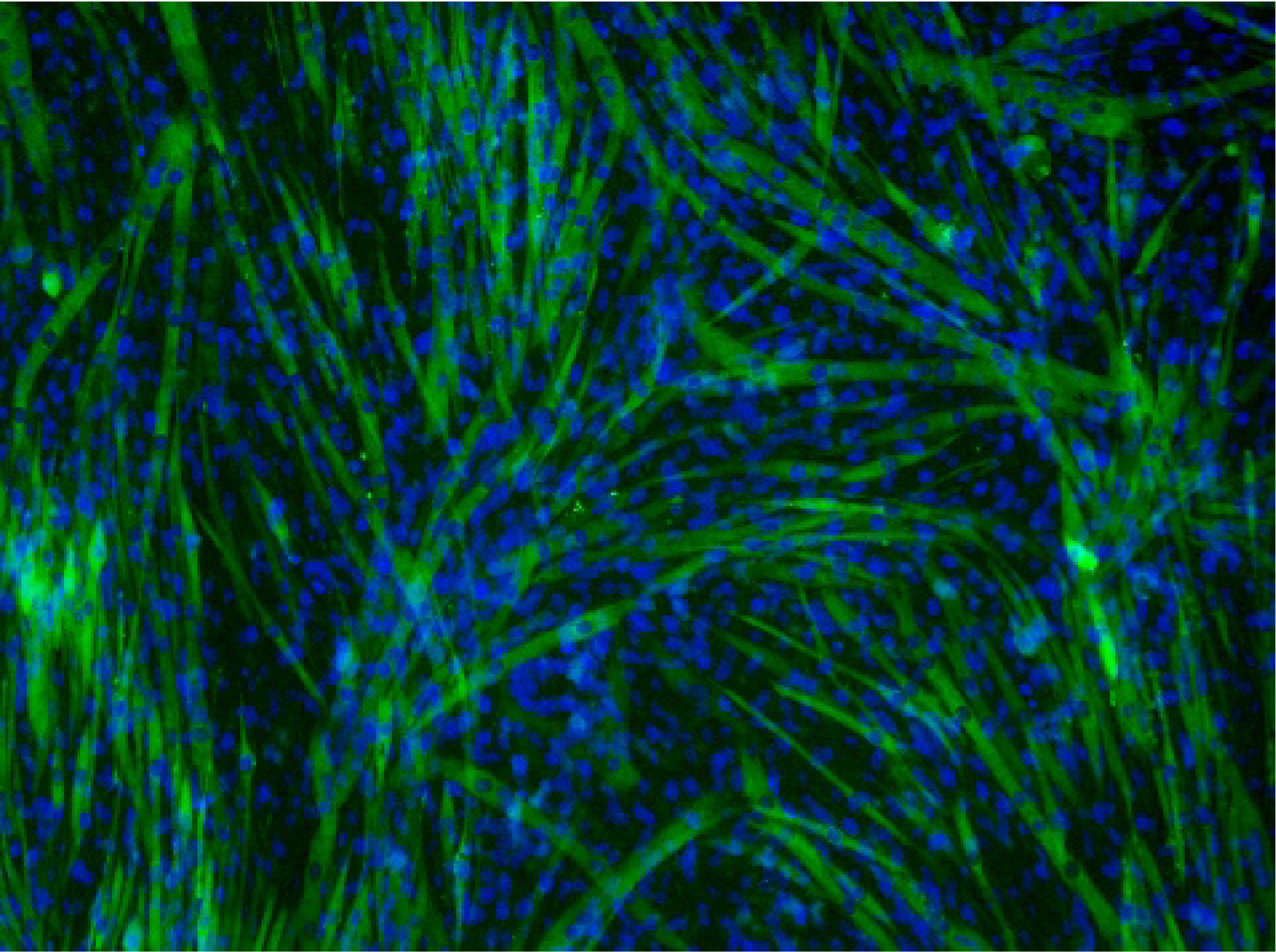

Supplement: Supplementary file 5 — Source data Fig. 2 [file 44318_2024_285_MOESM5_ESM.zip › Fig 2/Fig 2A/2-A-siControl.tif]

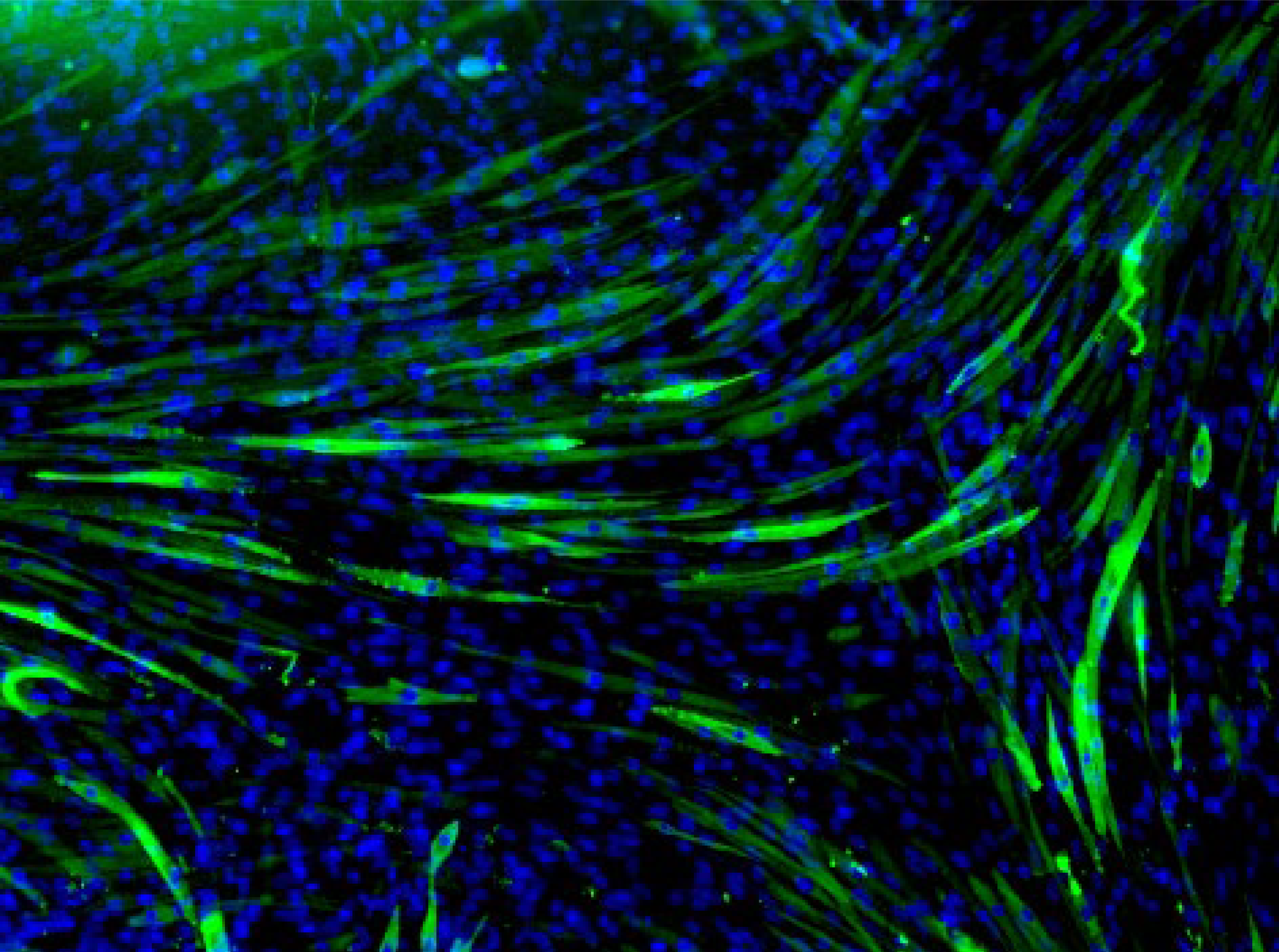

Supplement: Supplementary file 5 — Source data Fig. 2 [file 44318_2024_285_MOESM5_ESM.zip › Fig 2/Fig 2A/2-A-siFndc1.tif]

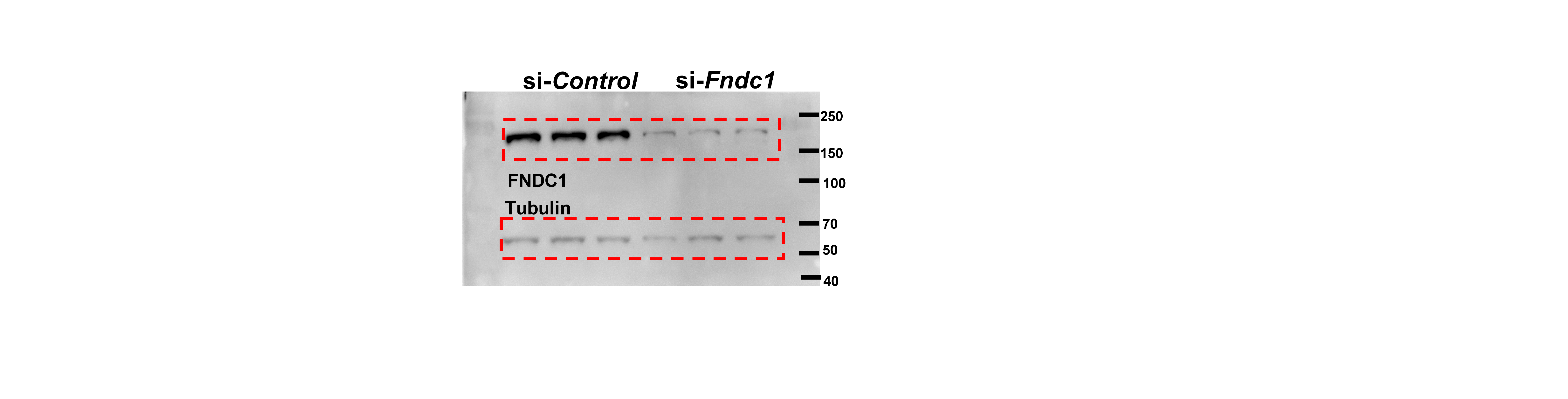

Supplement: Supplementary file 5 — Source data Fig. 2 [file 44318_2024_285_MOESM5_ESM.zip › Fig 2/Fig 2F/2-F-FNDC1 (2).tif]

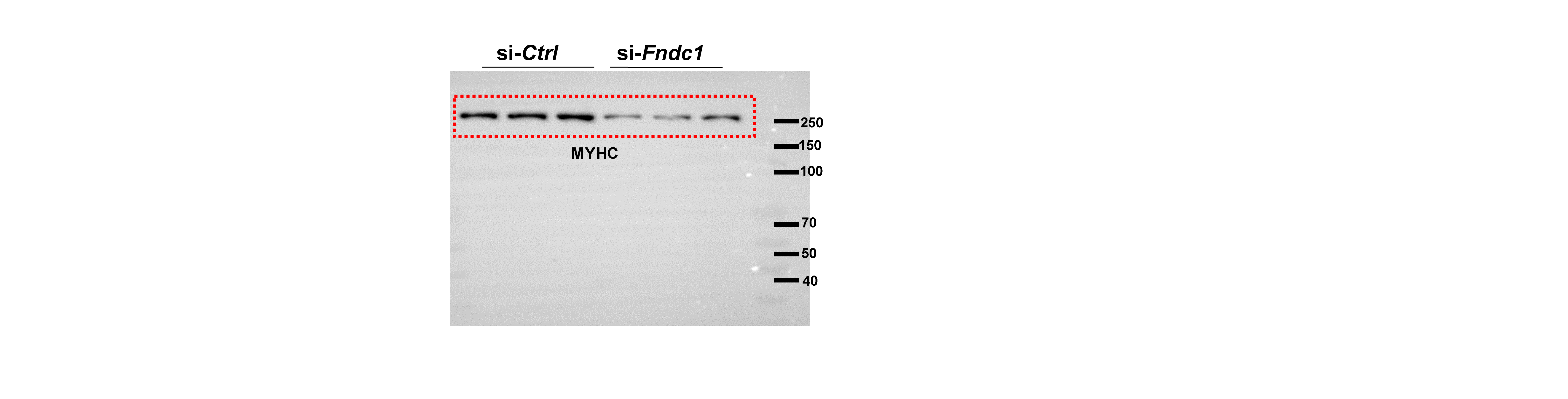

Supplement: Supplementary file 5 — Source data Fig. 2 [file 44318_2024_285_MOESM5_ESM.zip › Fig 2/Fig 2F/2-F-MYHC.tif]

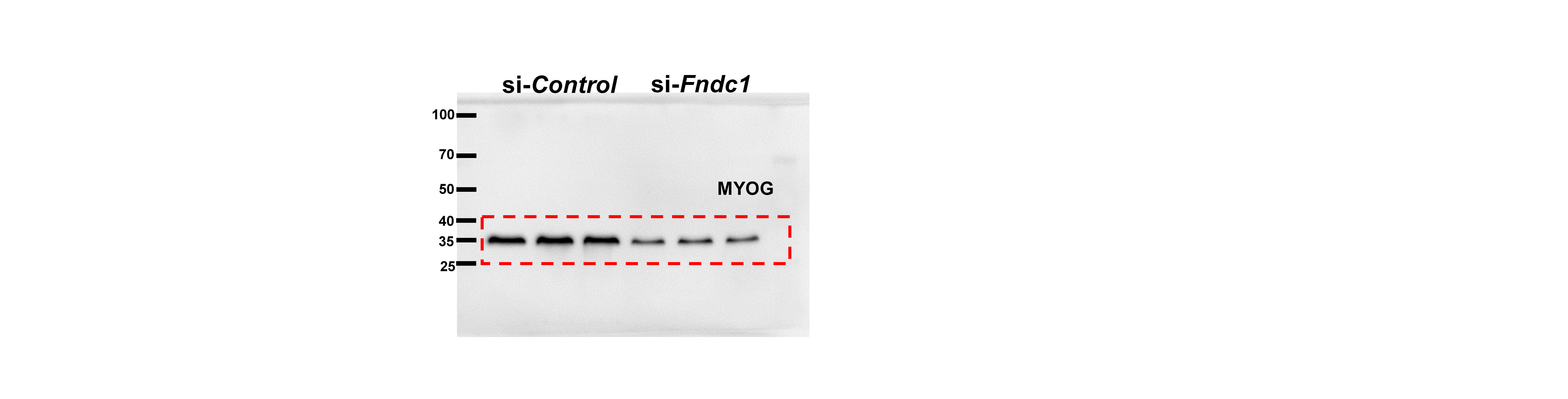

Supplement: Supplementary file 5 — Source data Fig. 2 [file 44318_2024_285_MOESM5_ESM.zip › Fig 2/Fig 2F/2-F-MYOG.tif]

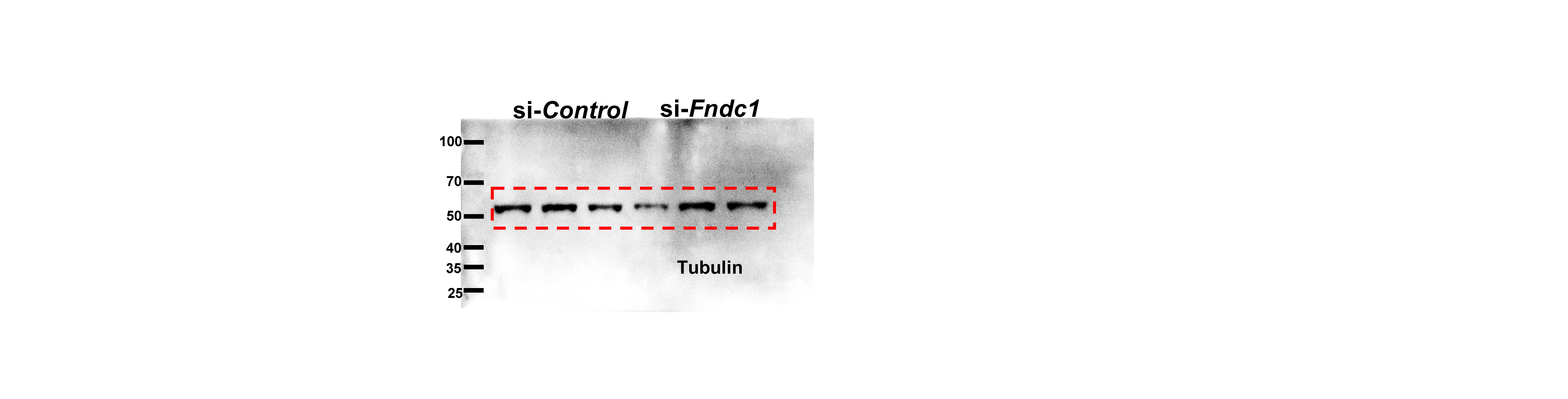

Supplement: Supplementary file 5 — Source data Fig. 2 [file 44318_2024_285_MOESM5_ESM.zip › Fig 2/Fig 2F/2-F-TUBULIN (2).tif]

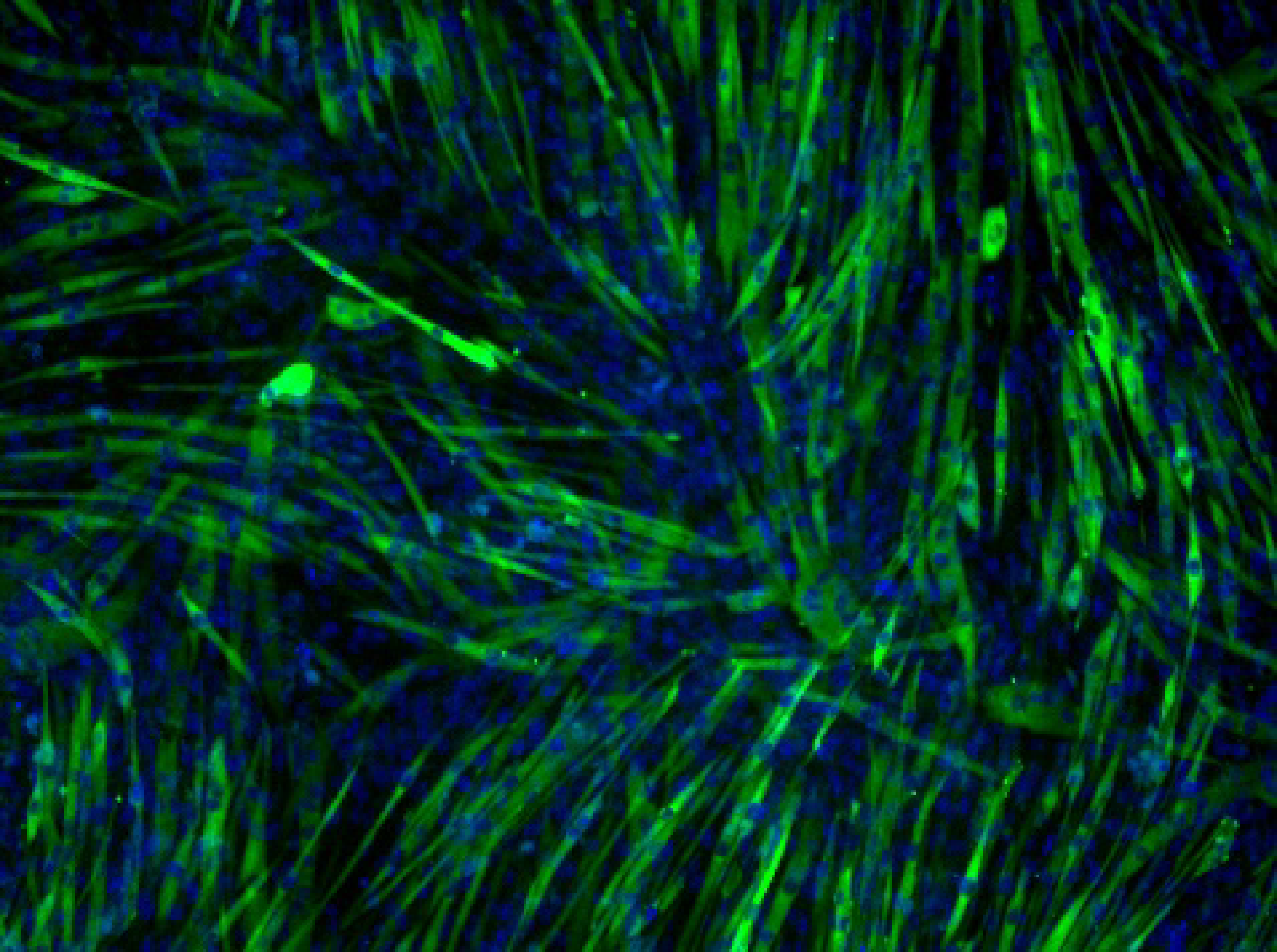

Supplement: Supplementary file 5 — Source data Fig. 2 [file 44318_2024_285_MOESM5_ESM.zip › Fig 2/Fig 2G/2-G-Control .tif]

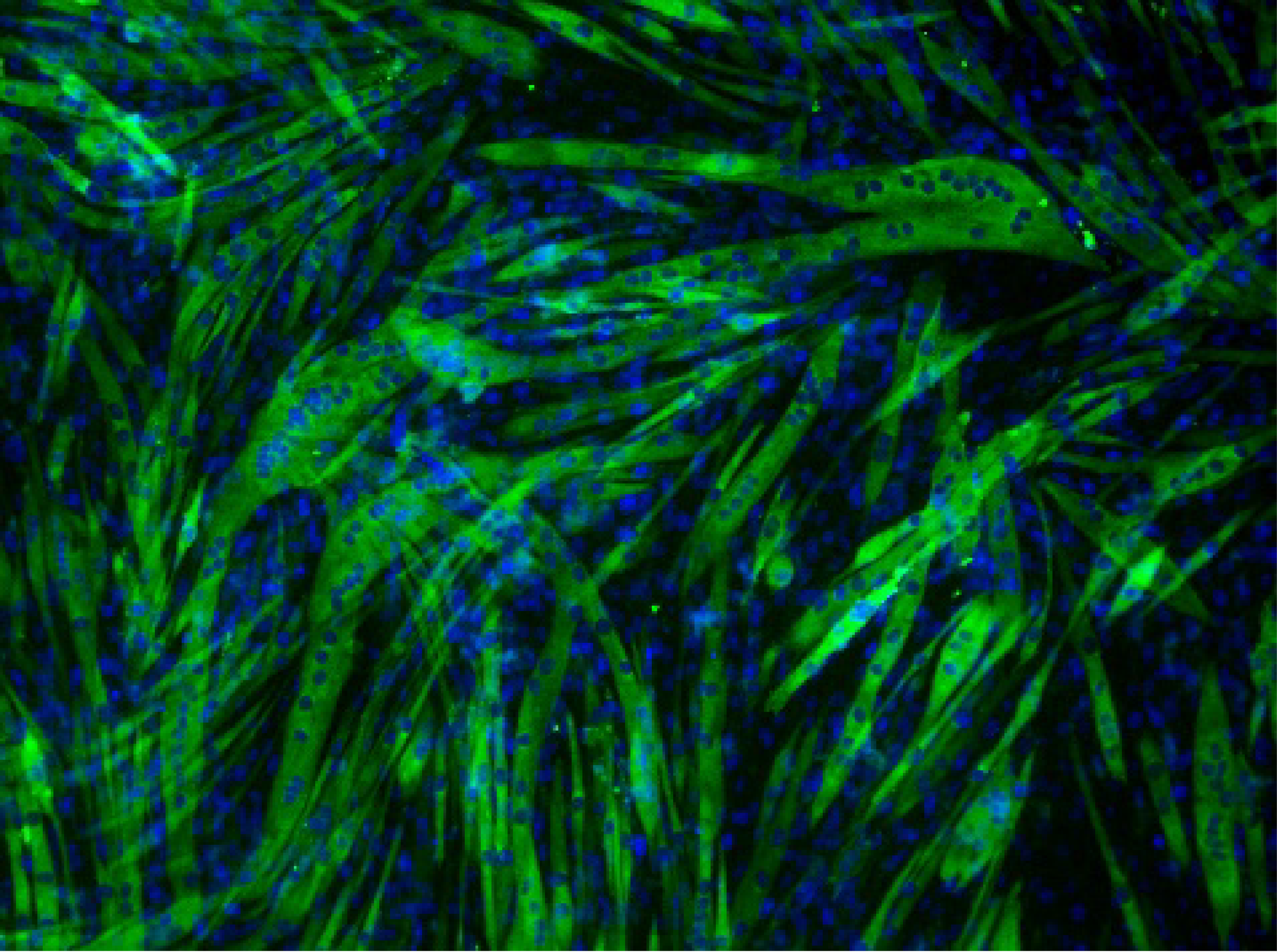

Supplement: Supplementary file 5 — Source data Fig. 2 [file 44318_2024_285_MOESM5_ESM.zip › Fig 2/Fig 2G/2-G-Fndc1-OE .tif]

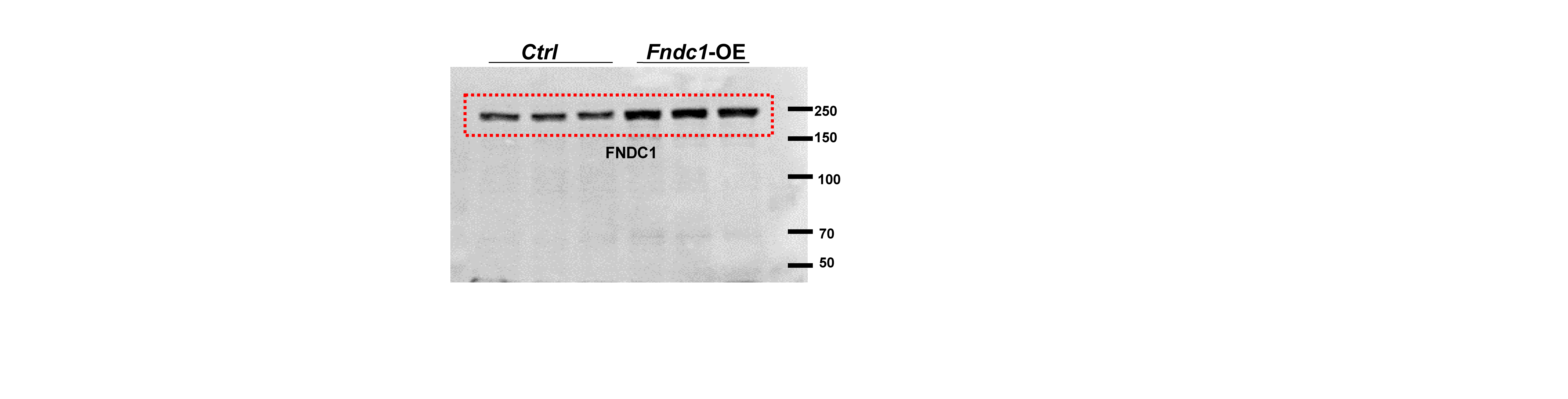

Supplement: Supplementary file 5 — Source data Fig. 2 [file 44318_2024_285_MOESM5_ESM.zip › Fig 2/Fig 2L/2-L-FNDC1.tif]

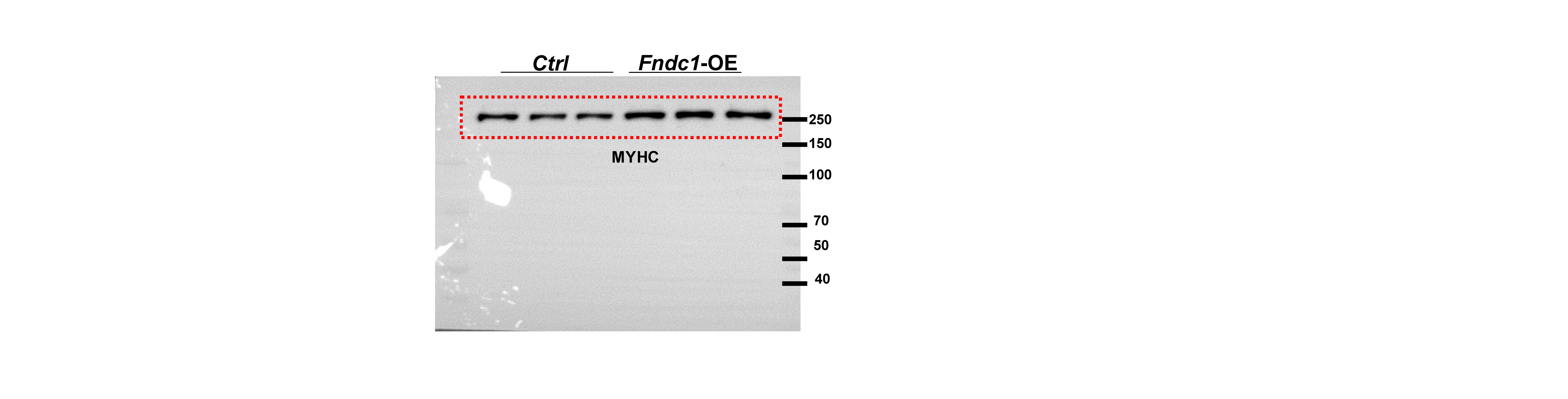

Supplement: Supplementary file 5 — Source data Fig. 2 [file 44318_2024_285_MOESM5_ESM.zip › Fig 2/Fig 2L/2-L-MYHC.tif]

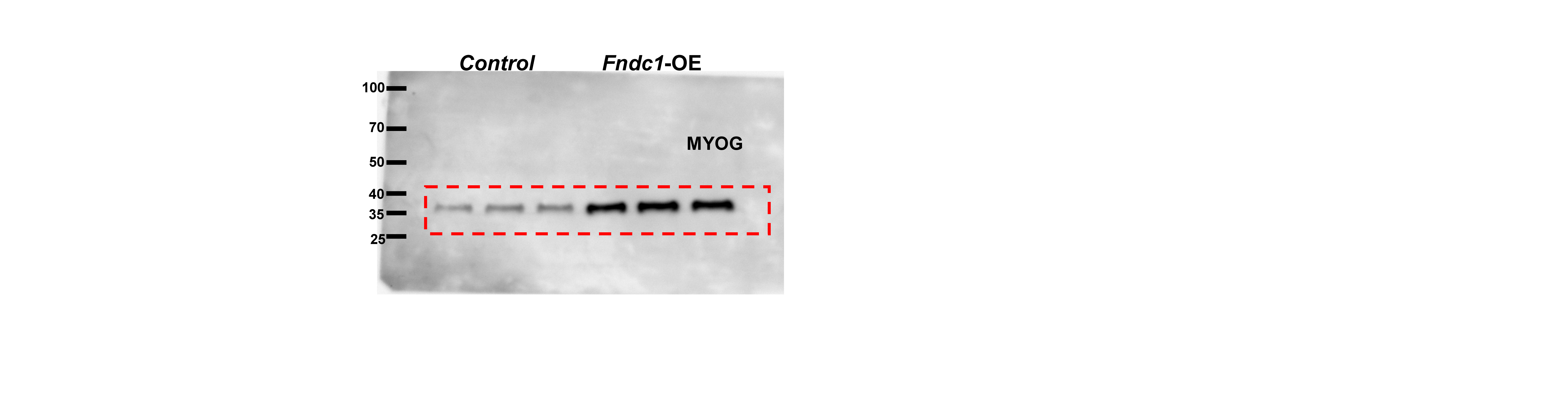

Supplement: Supplementary file 5 — Source data Fig. 2 [file 44318_2024_285_MOESM5_ESM.zip › Fig 2/Fig 2L/2-L-MYOG.tif]

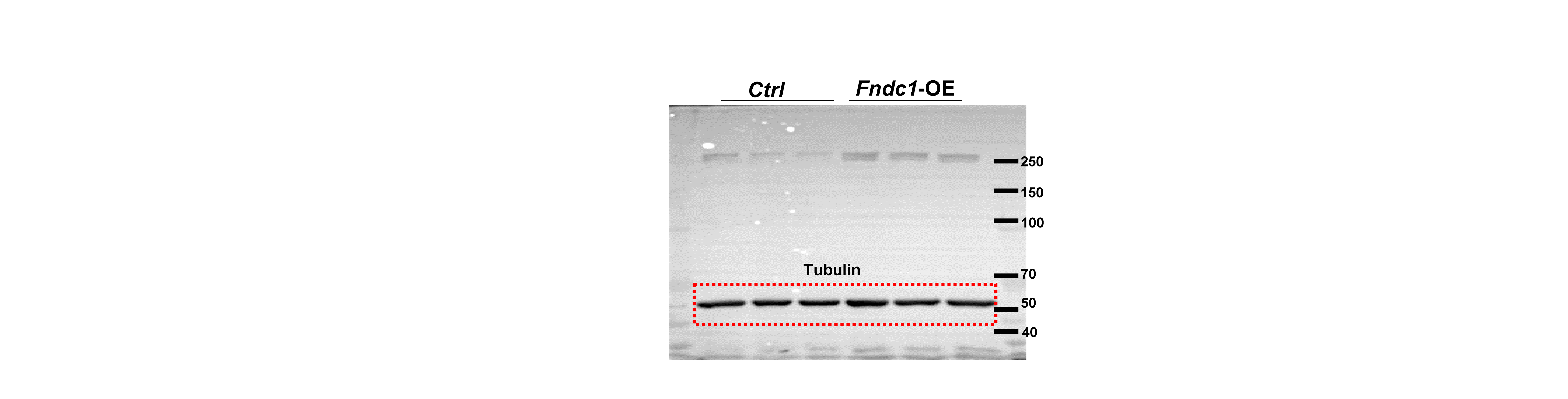

Supplement: Supplementary file 5 — Source data Fig. 2 [file 44318_2024_285_MOESM5_ESM.zip › Fig 2/Fig 2L/2-L-TUBULIN.tif]

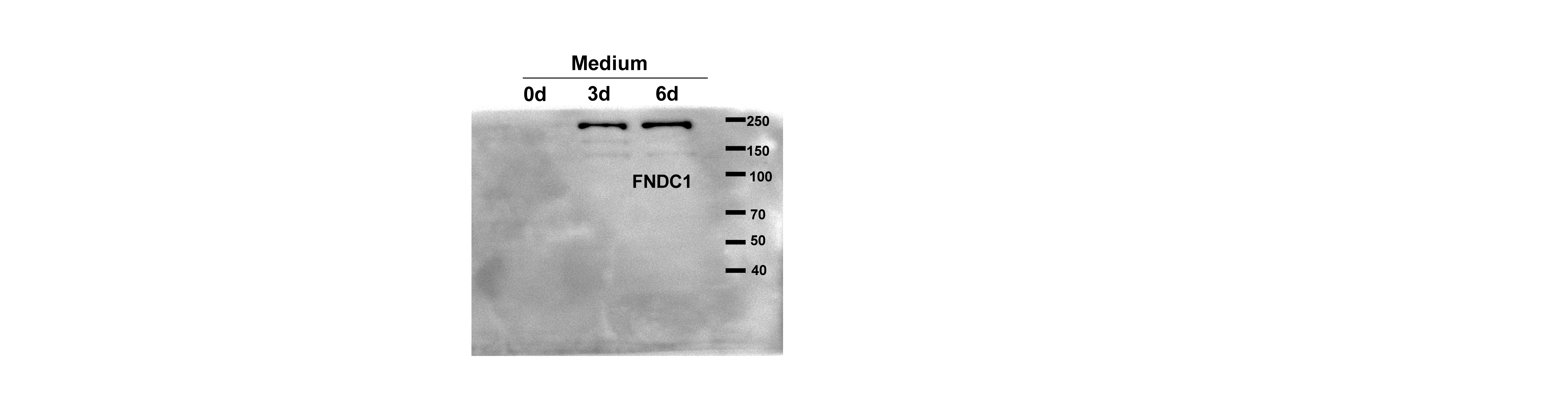

Supplement: Supplementary file 6 — Source data Fig. 3 [file 44318_2024_285_MOESM6_ESM.zip › Fig 3/Fig 3A/3-A-FNDC1.tif]

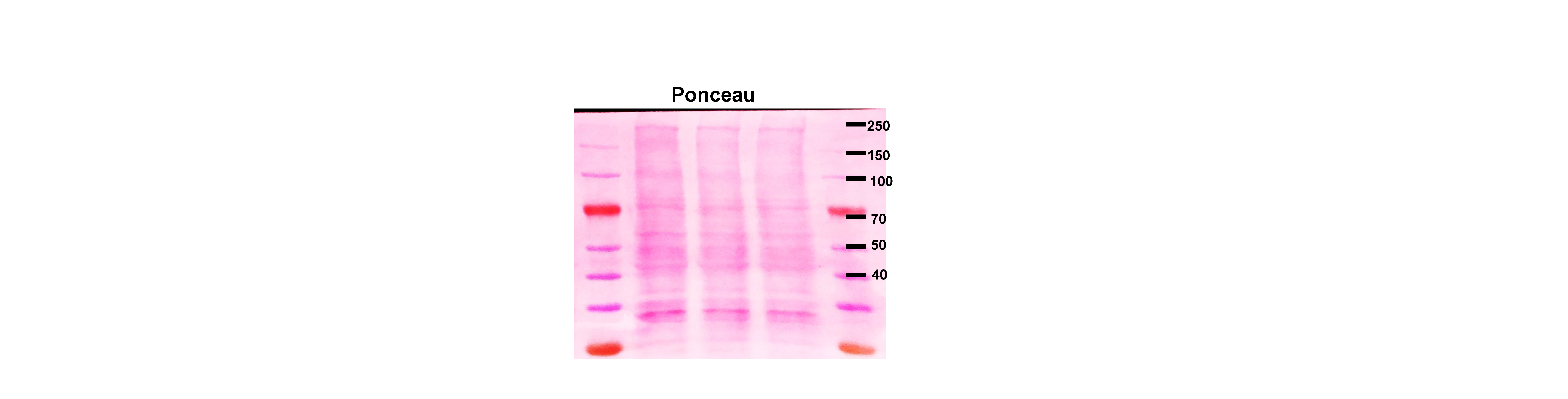

Supplement: Supplementary file 6 — Source data Fig. 3 [file 44318_2024_285_MOESM6_ESM.zip › Fig 3/Fig 3A/3-A-PONCEAU.tif]

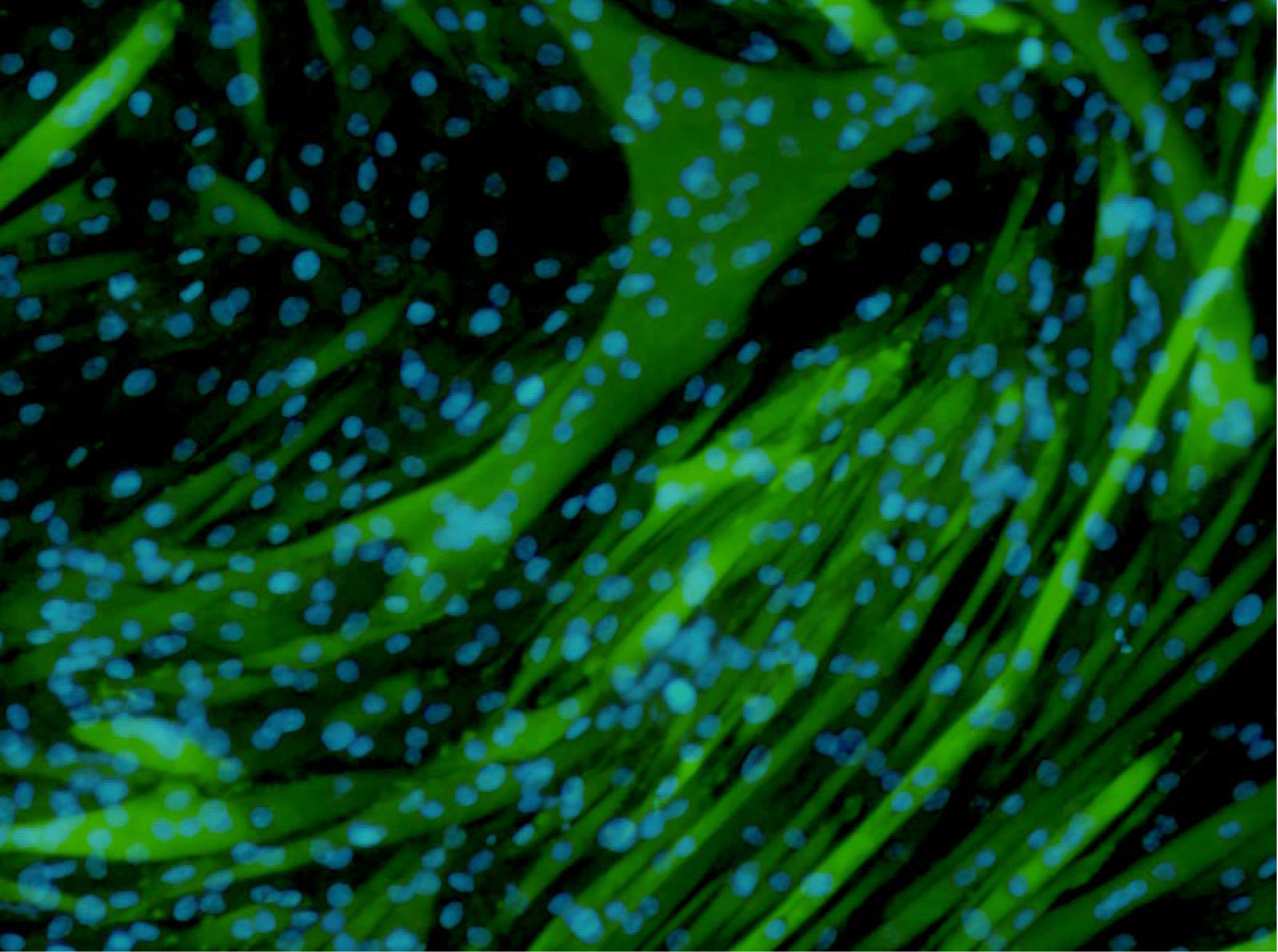

Supplement: Supplementary file 6 — Source data Fig. 3 [file 44318_2024_285_MOESM6_ESM.zip › Fig 3/Fig 3E/3-E-FNDC1-FN3.tif]

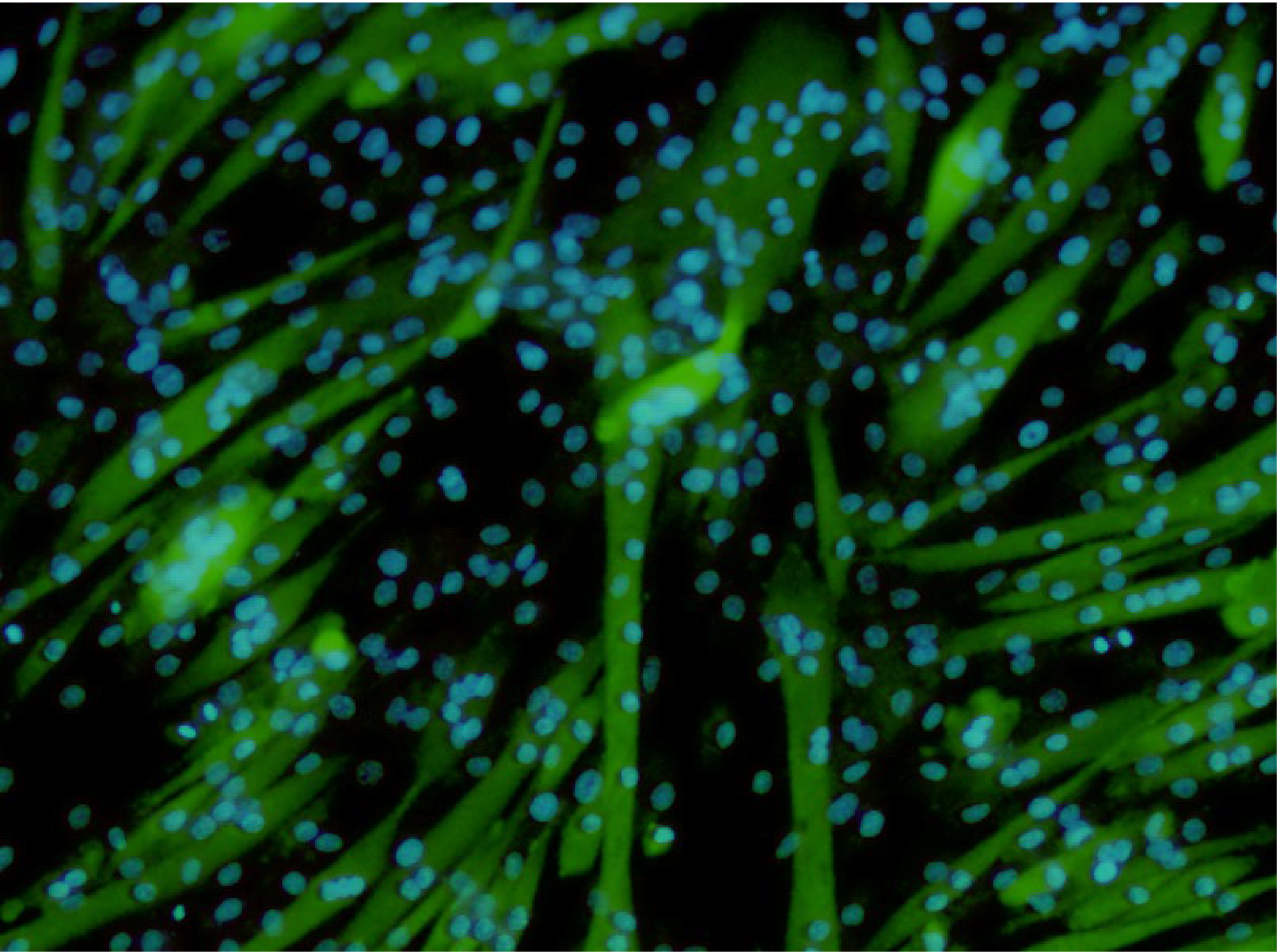

Supplement: Supplementary file 6 — Source data Fig. 3 [file 44318_2024_285_MOESM6_ESM.zip › Fig 3/Fig 3E/3E-Control.tif]

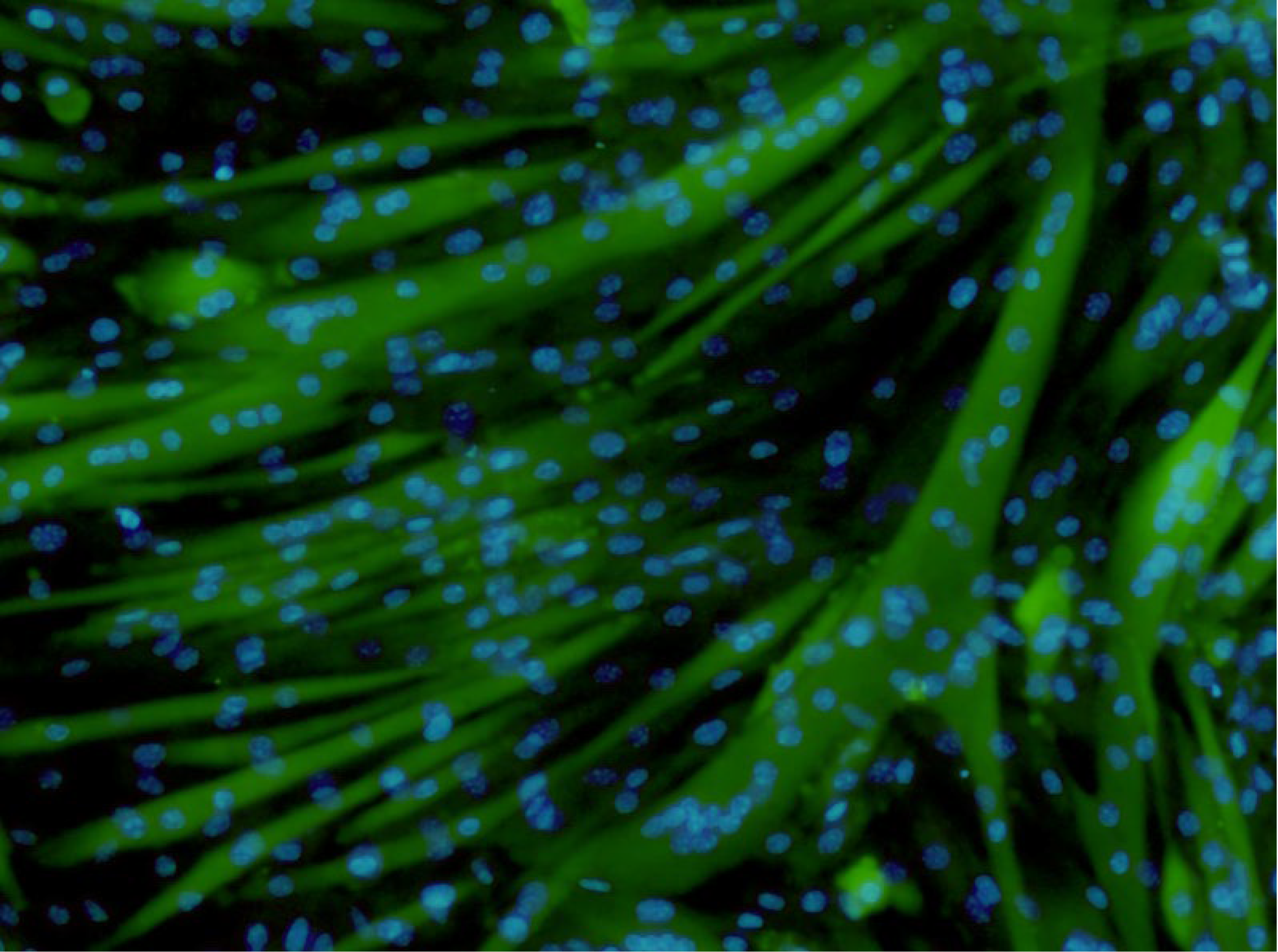

Supplement: Supplementary file 6 — Source data Fig. 3 [file 44318_2024_285_MOESM6_ESM.zip › Fig 3/Fig 3E/3E-FNDC1-FL .tif]

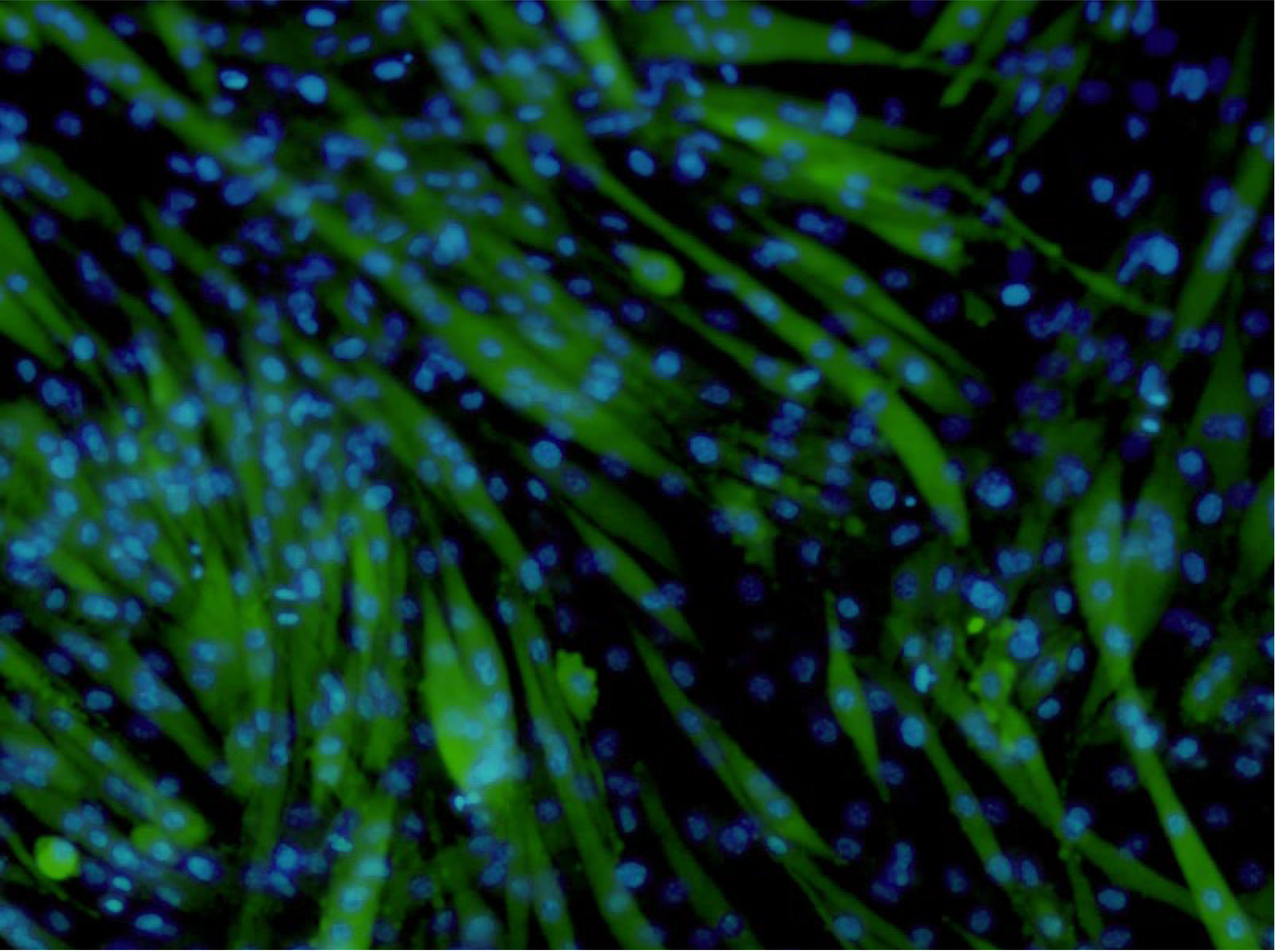

Supplement: Supplementary file 6 — Source data Fig. 3 [file 44318_2024_285_MOESM6_ESM.zip › Fig 3/Fig 3E/3E-FNDC1-PHA .tif]

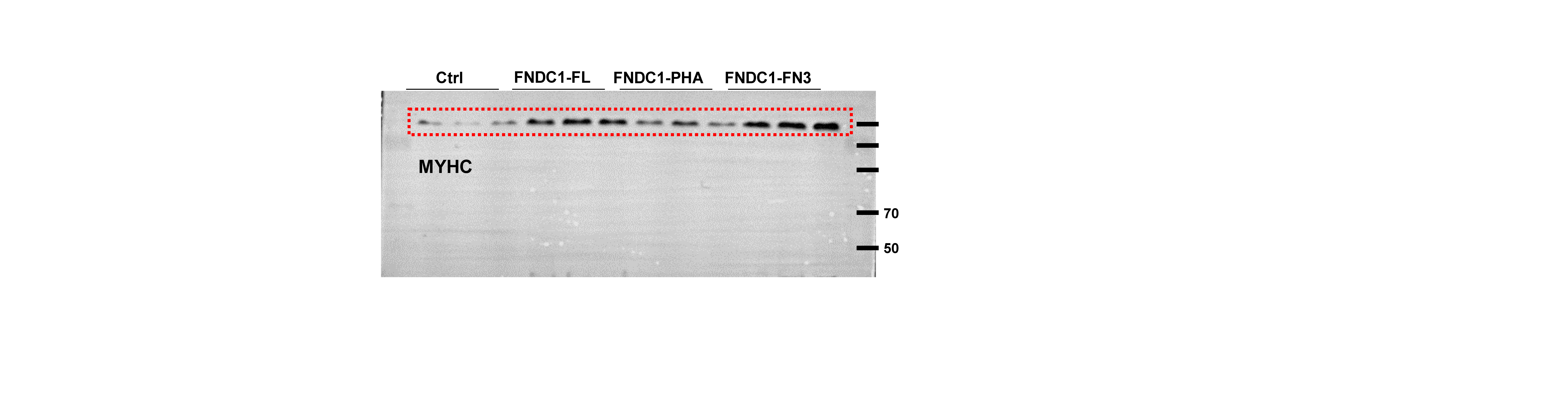

Supplement: Supplementary file 6 — Source data Fig. 3 [file 44318_2024_285_MOESM6_ESM.zip › Fig 3/Fig 3H/3-H-MYHC.tif]

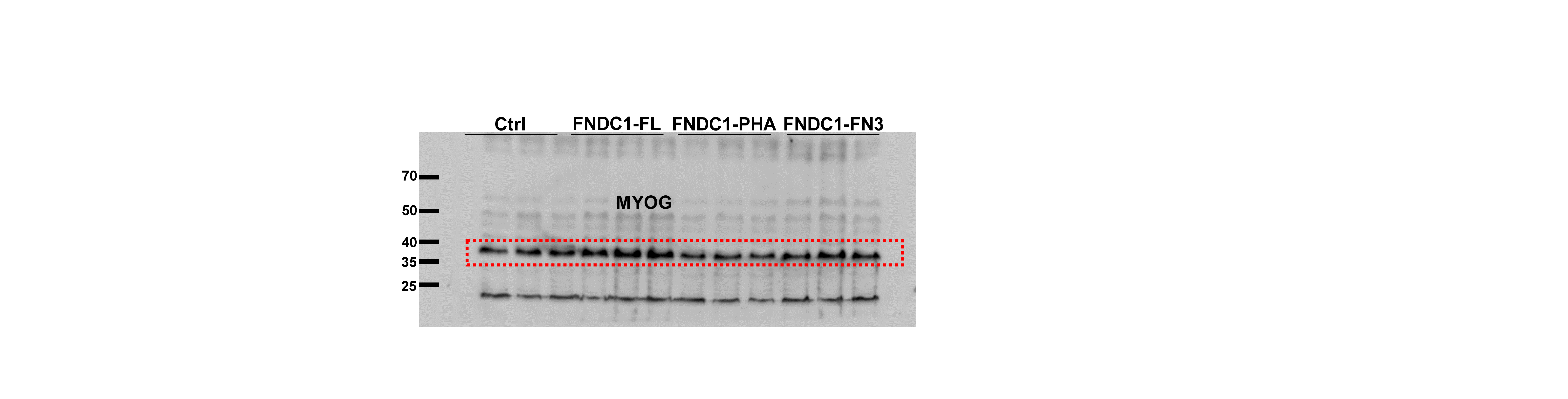

Supplement: Supplementary file 6 — Source data Fig. 3 [file 44318_2024_285_MOESM6_ESM.zip › Fig 3/Fig 3H/3-H-MYOG.tif]

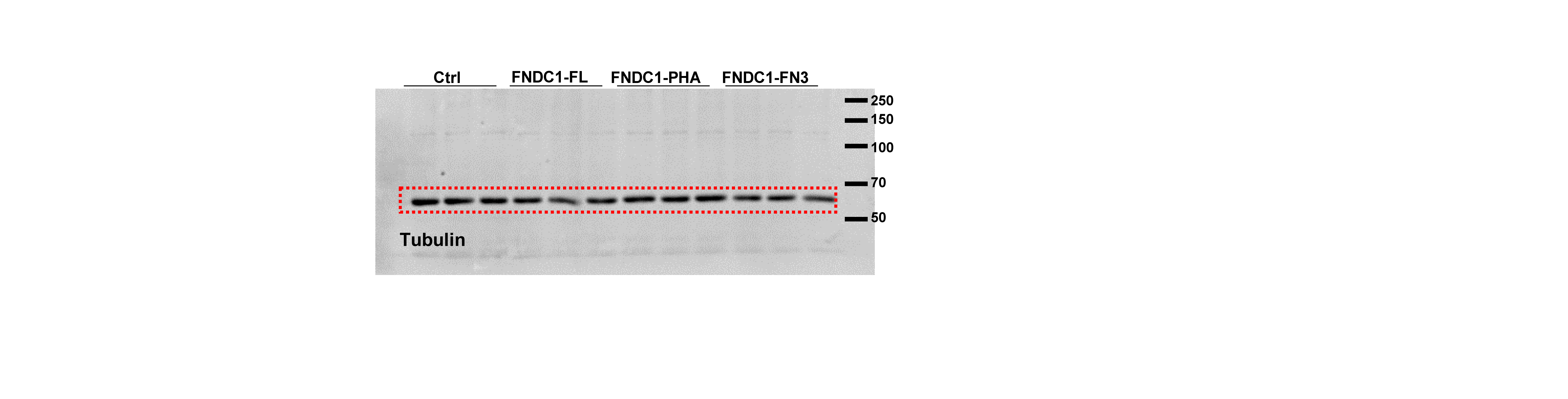

Supplement: Supplementary file 6 — Source data Fig. 3 [file 44318_2024_285_MOESM6_ESM.zip › Fig 3/Fig 3H/3-H-TUBULIN.tif]

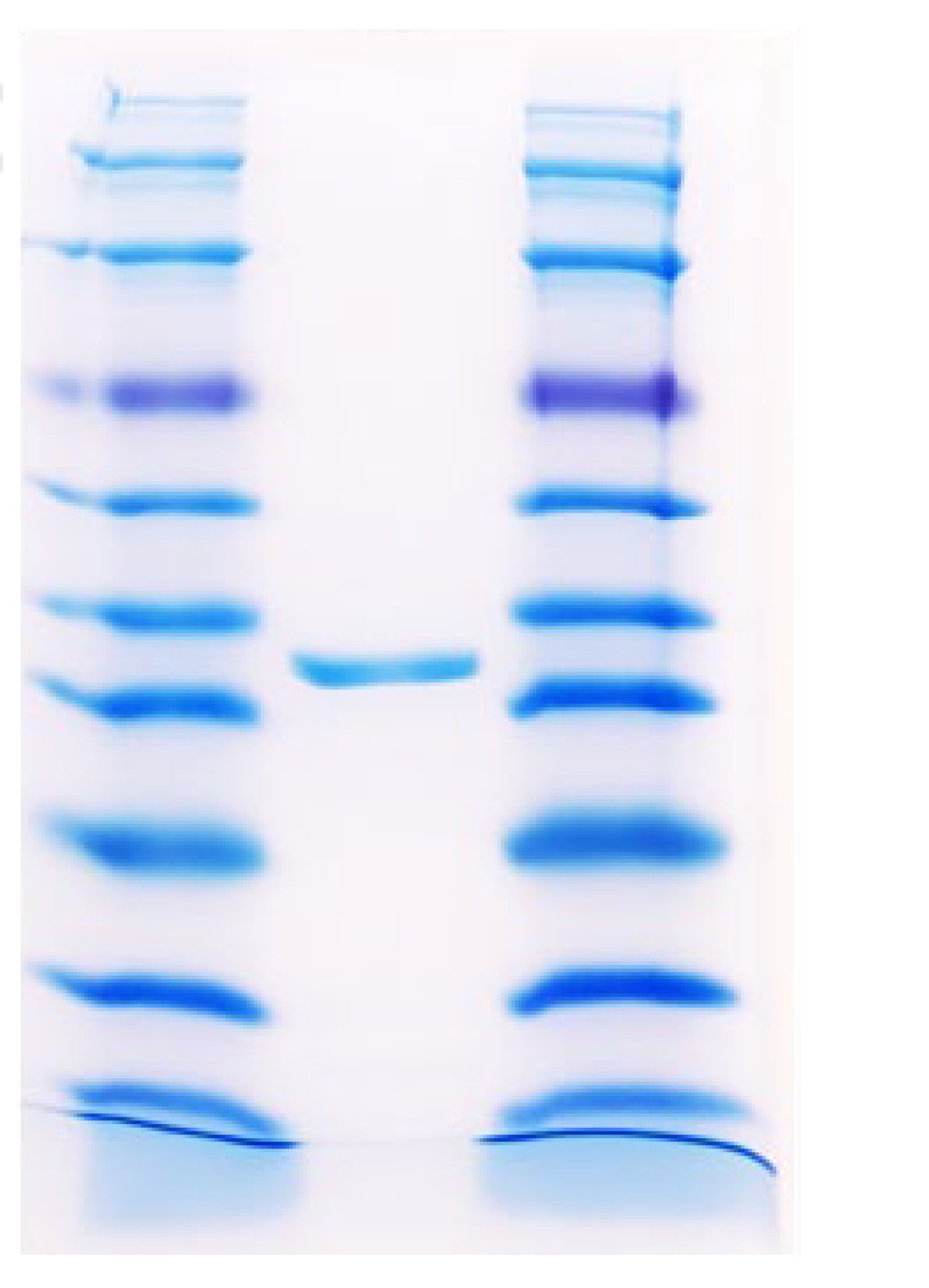

Supplement: Supplementary file 6 — Source data Fig. 3 [file 44318_2024_285_MOESM6_ESM.zip › Fig 3/Fig 3J/1.tif]

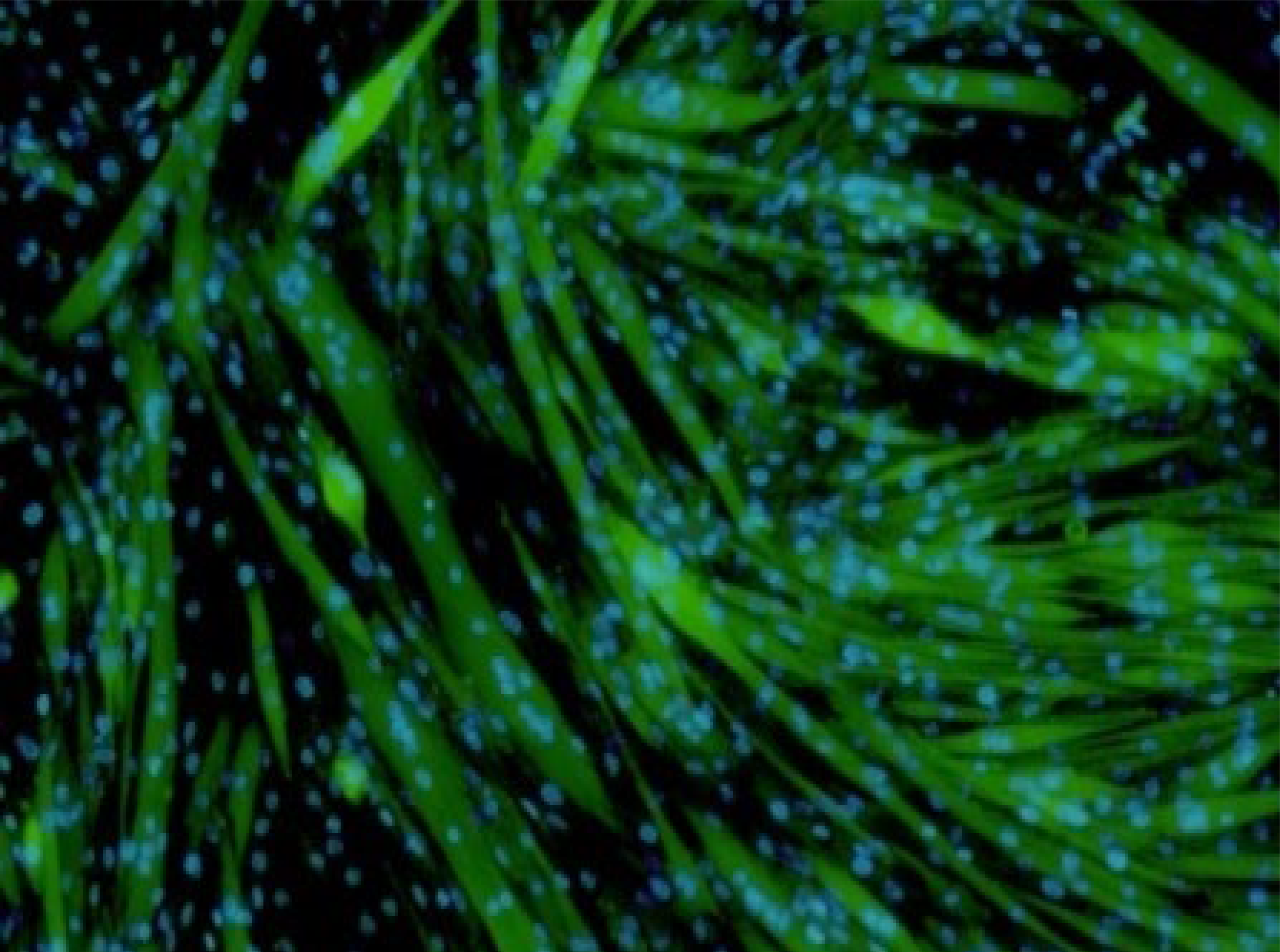

Supplement: Supplementary file 6 — Source data Fig. 3 [file 44318_2024_285_MOESM6_ESM.zip › Fig 3/Fig 3M/3M-0.01.tif]

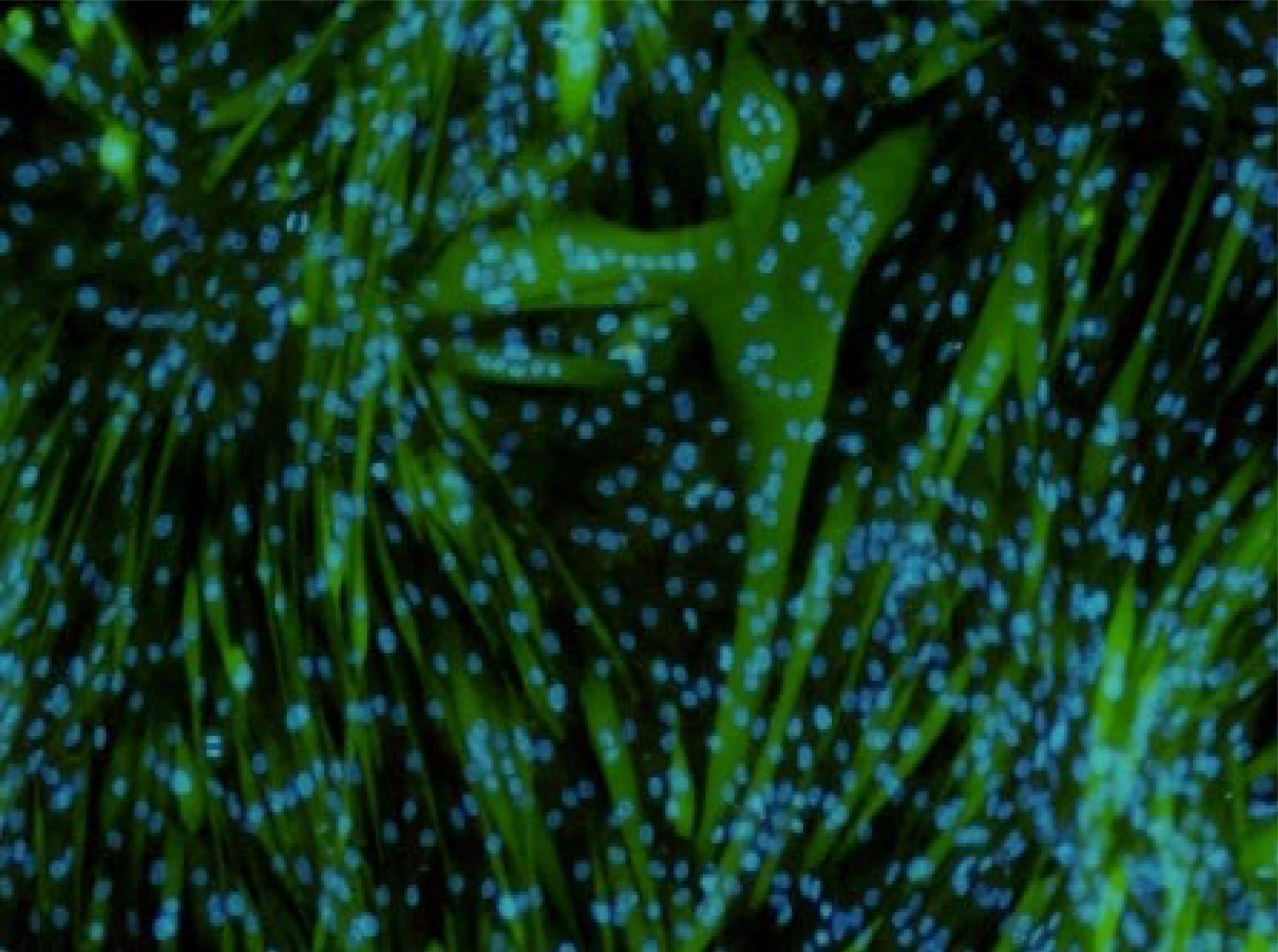

Supplement: Supplementary file 6 — Source data Fig. 3 [file 44318_2024_285_MOESM6_ESM.zip › Fig 3/Fig 3M/3M-0.1.tif]
