# Supplementary figures and images for: FNDC1 is a myokine that promotes myogenesis and muscle regeneration (part 2 of 2)
Source: EMBO J. 2024 Nov 20;44(1):30–53. doi: 10.1038/s44318-024-00285-0 (PMC11695938; doi:10.1038/s44318-024-00285-0)

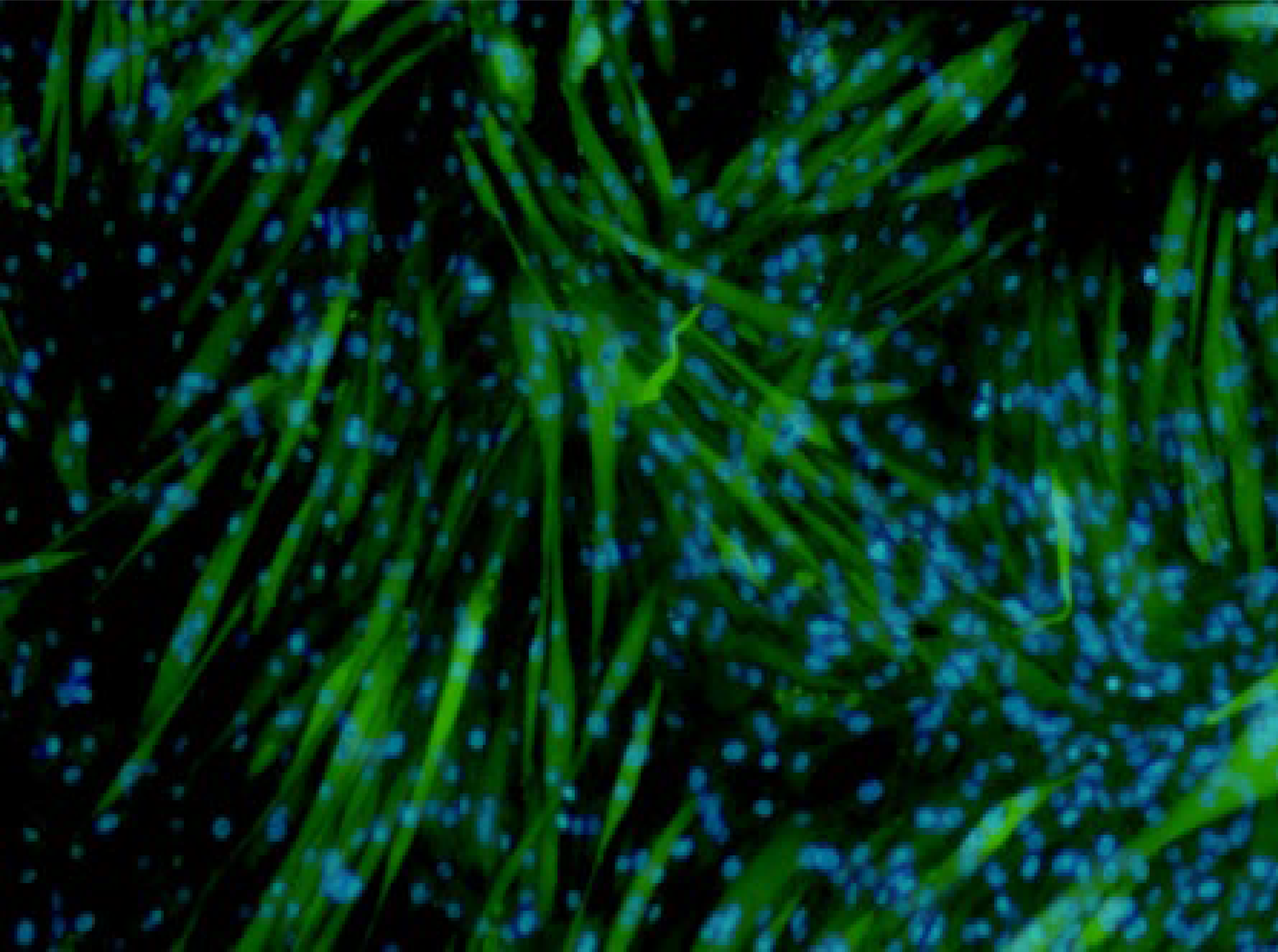

Supplement: Supplementary file 6 — Source data Fig. 3 [file 44318_2024_285_MOESM6_ESM.zip › Fig 3/Fig 3M/3M-0.tif]

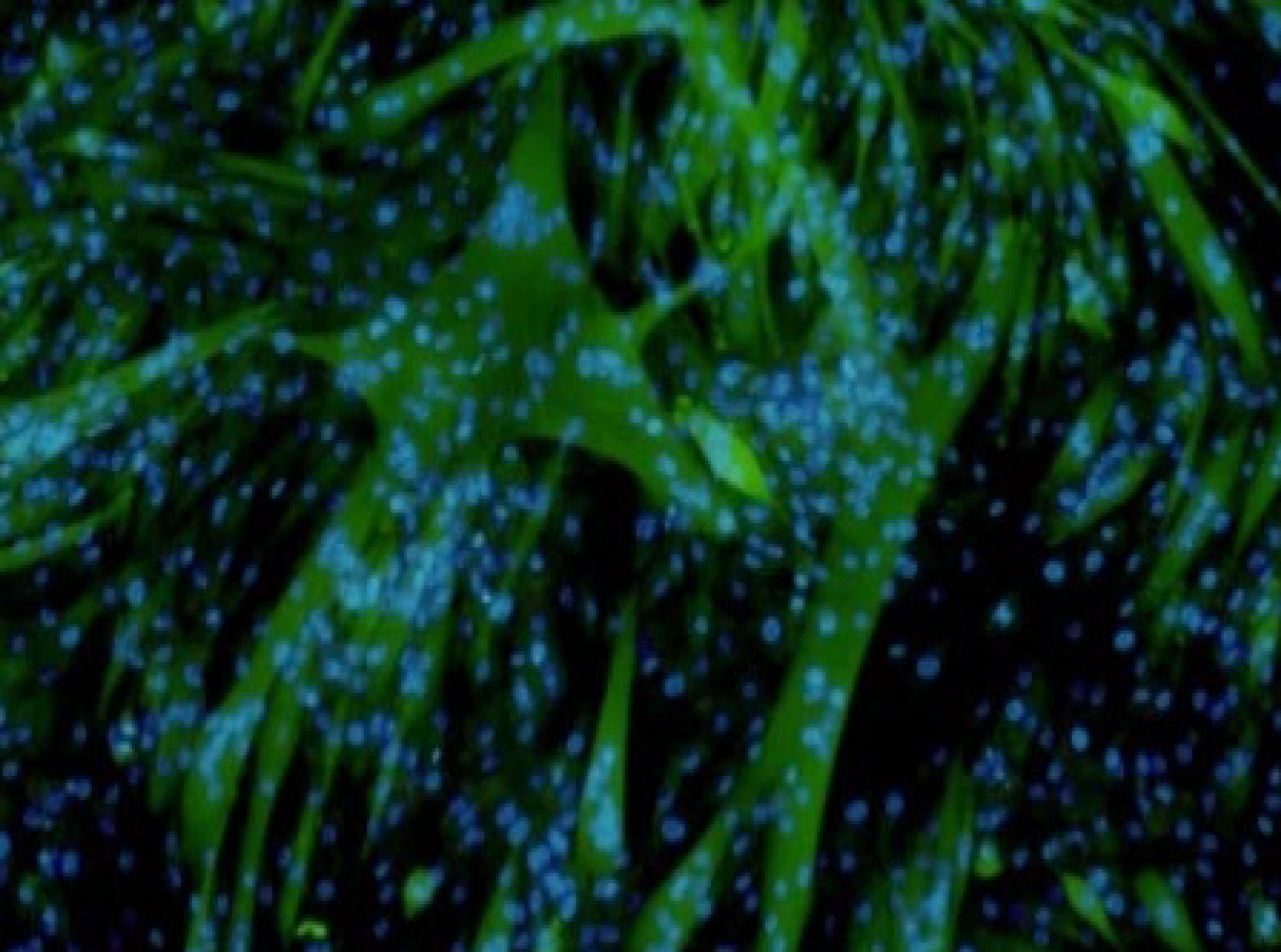

Supplement: Supplementary file 6 — Source data Fig. 3 [file 44318_2024_285_MOESM6_ESM.zip › Fig 3/Fig 3M/3M-1.tif]

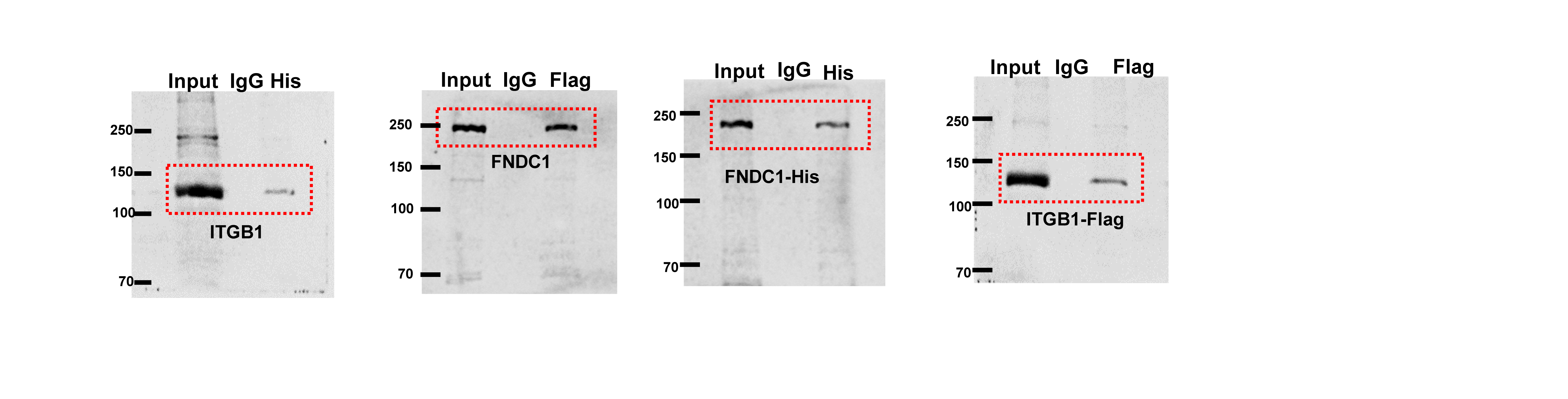

Supplement: Supplementary file 7 — Source data Fig. 4 [file 44318_2024_285_MOESM7_ESM.zip › Fig 4/Fig 4B/4-B.tif]

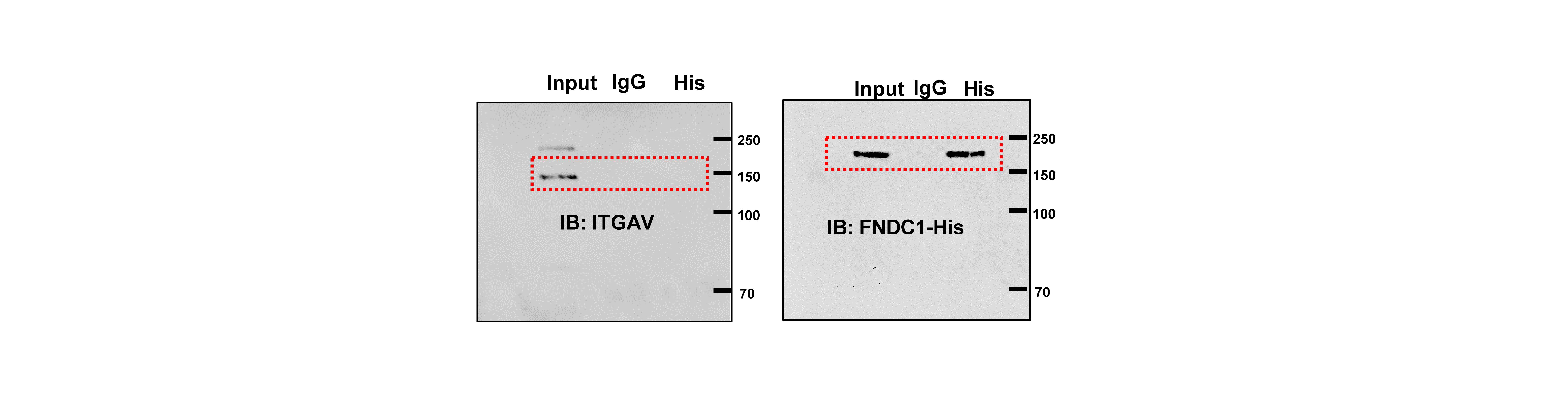

Supplement: Supplementary file 7 — Source data Fig. 4 [file 44318_2024_285_MOESM7_ESM.zip › Fig 4/Fig 4C/4-C.tif]

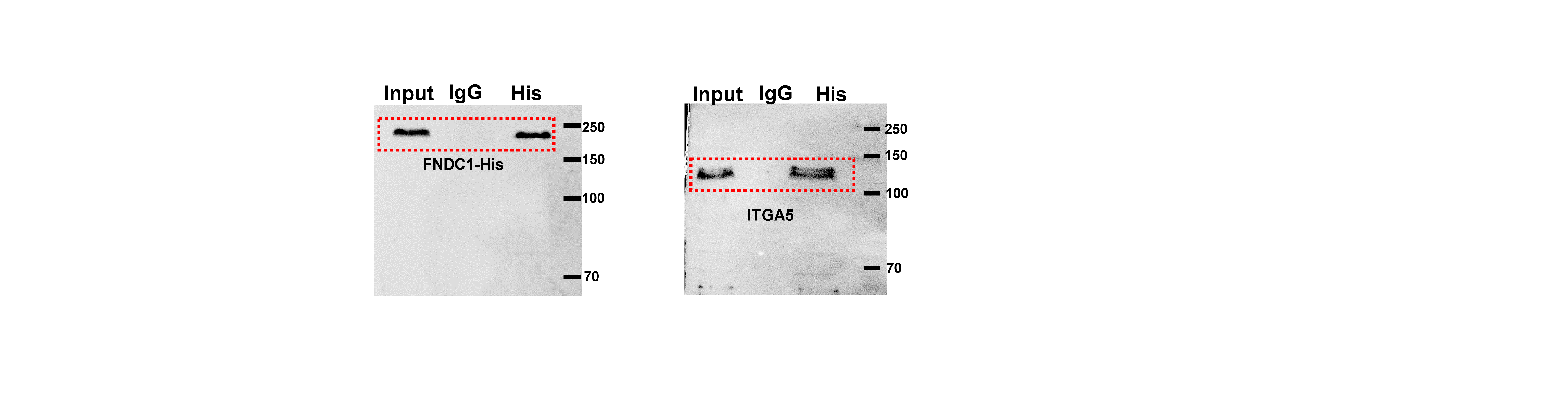

Supplement: Supplementary file 7 — Source data Fig. 4 [file 44318_2024_285_MOESM7_ESM.zip › Fig 4/Fig 4D/4-D.tif]

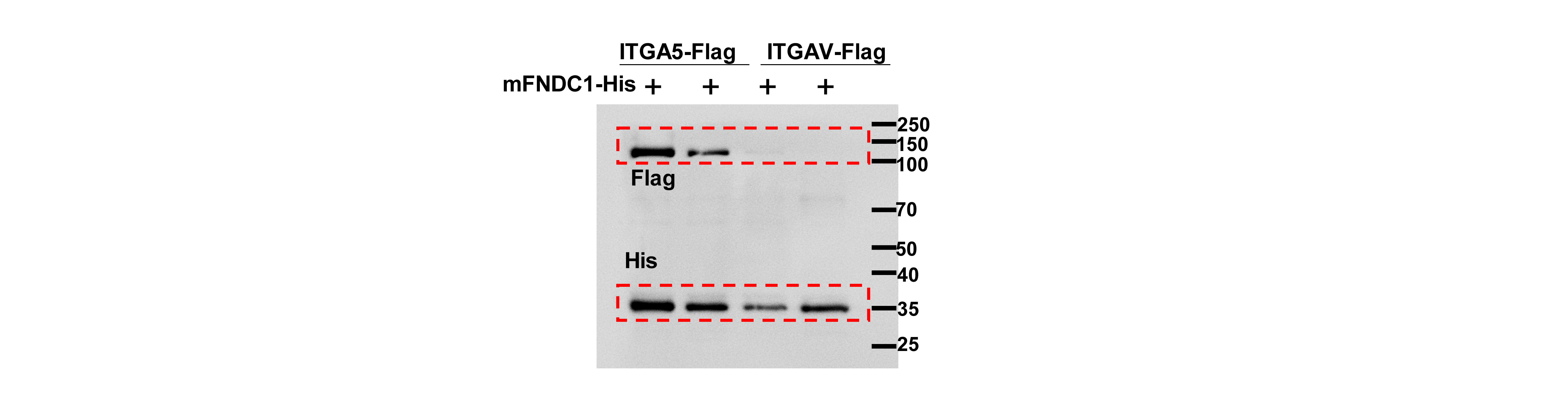

Supplement: Supplementary file 7 — Source data Fig. 4 [file 44318_2024_285_MOESM7_ESM.zip › Fig 4/Fig 4E/4-E.tif]

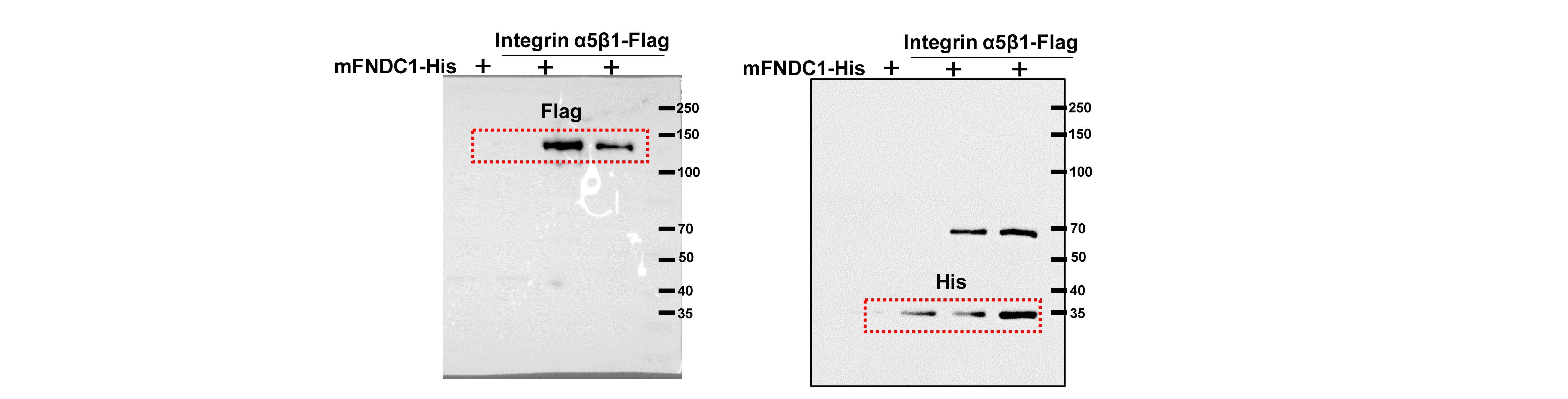

Supplement: Supplementary file 7 — Source data Fig. 4 [file 44318_2024_285_MOESM7_ESM.zip › Fig 4/Fig 4F/4-F.tif]

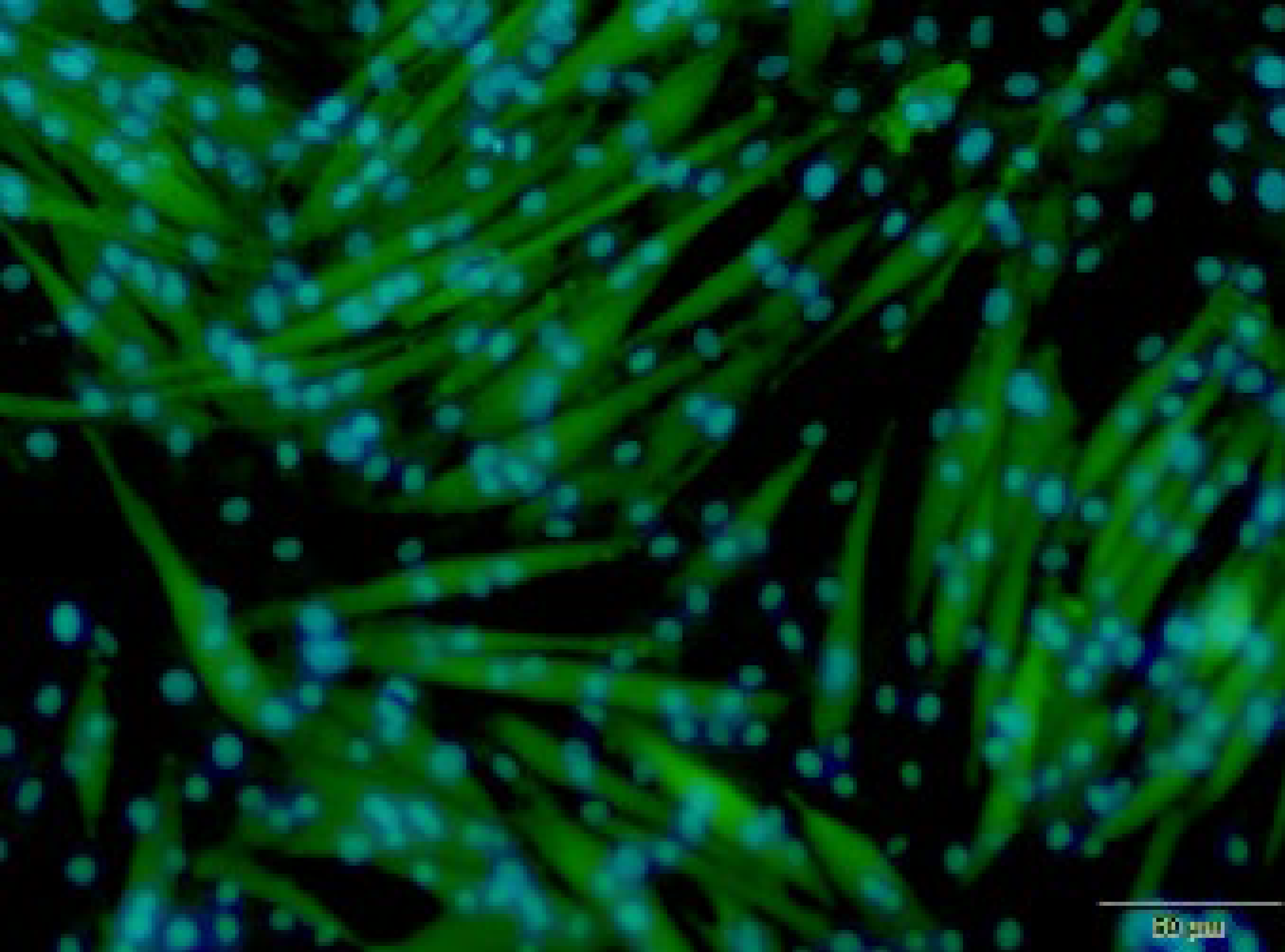

Supplement: Supplementary file 7 — Source data Fig. 4 [file 44318_2024_285_MOESM7_ESM.zip › Fig 4/Fig 4H/4H-Control+DMSO.tif]

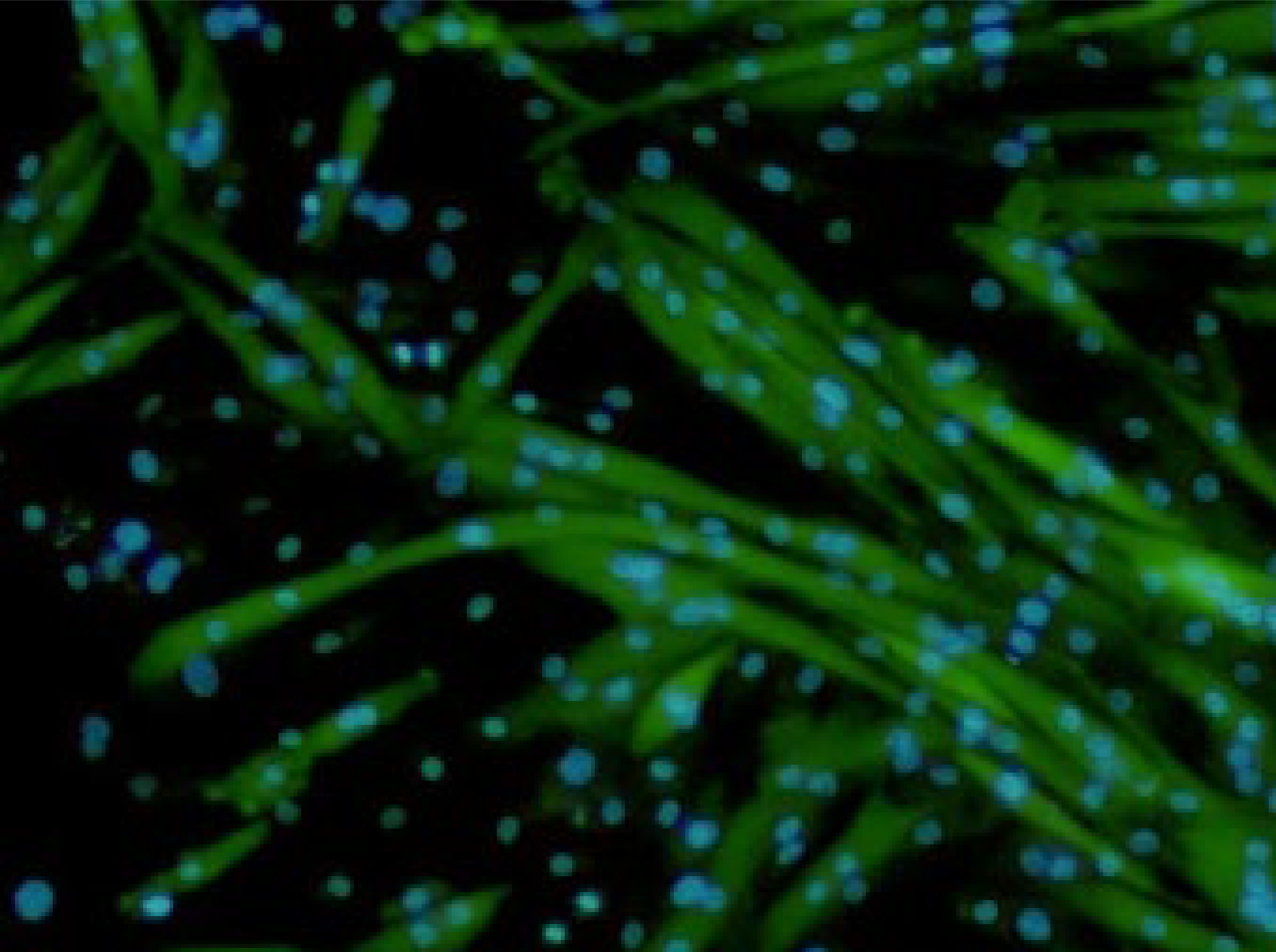

Supplement: Supplementary file 7 — Source data Fig. 4 [file 44318_2024_285_MOESM7_ESM.zip › Fig 4/Fig 4H/4H-Control+k43c.tif]

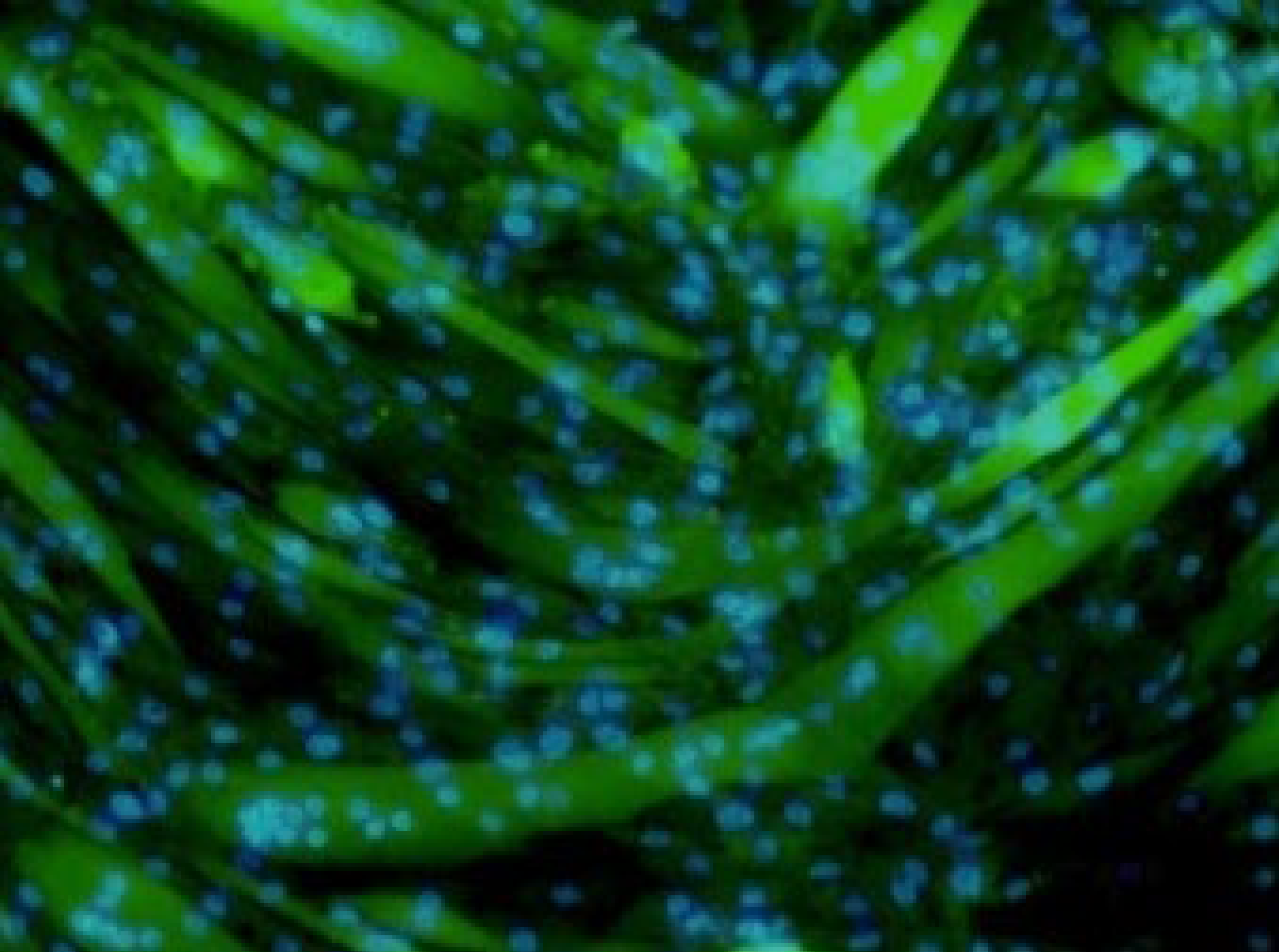

Supplement: Supplementary file 7 — Source data Fig. 4 [file 44318_2024_285_MOESM7_ESM.zip › Fig 4/Fig 4H/4H-mFNDC1+DMSO.tif]

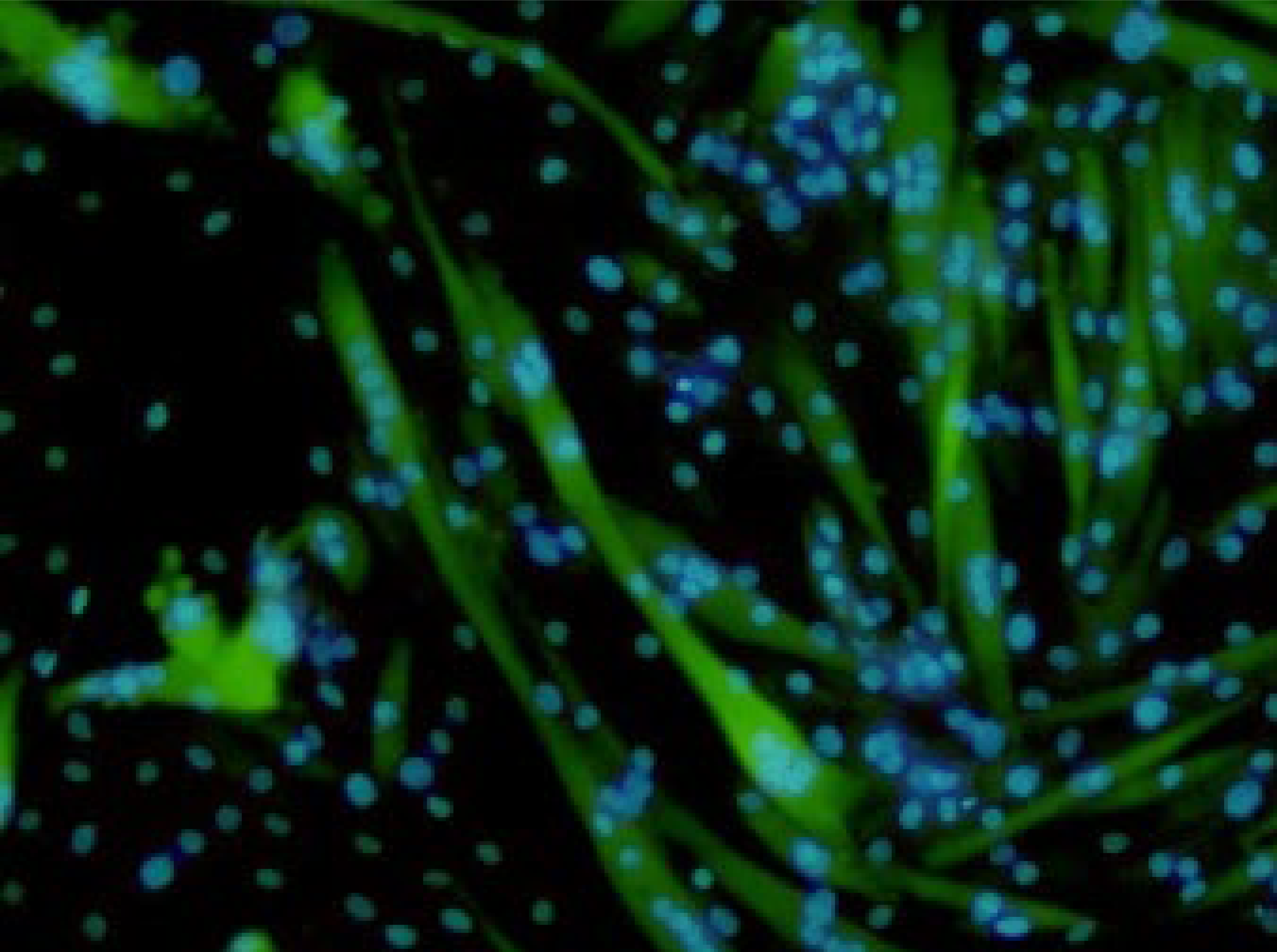

Supplement: Supplementary file 7 — Source data Fig. 4 [file 44318_2024_285_MOESM7_ESM.zip › Fig 4/Fig 4H/4H-mFNDC1+K43C.tif]

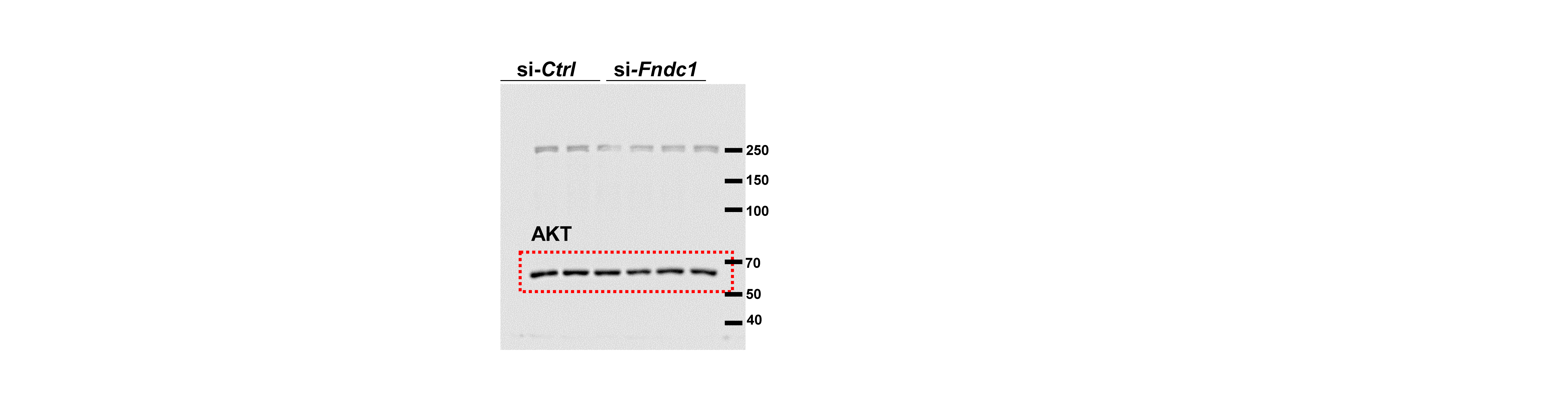

Supplement: Supplementary file 8 — Source data Fig. 5 [file 44318_2024_285_MOESM8_ESM.zip › Fig 5/Fig 5C/5-C-AKT.tif]

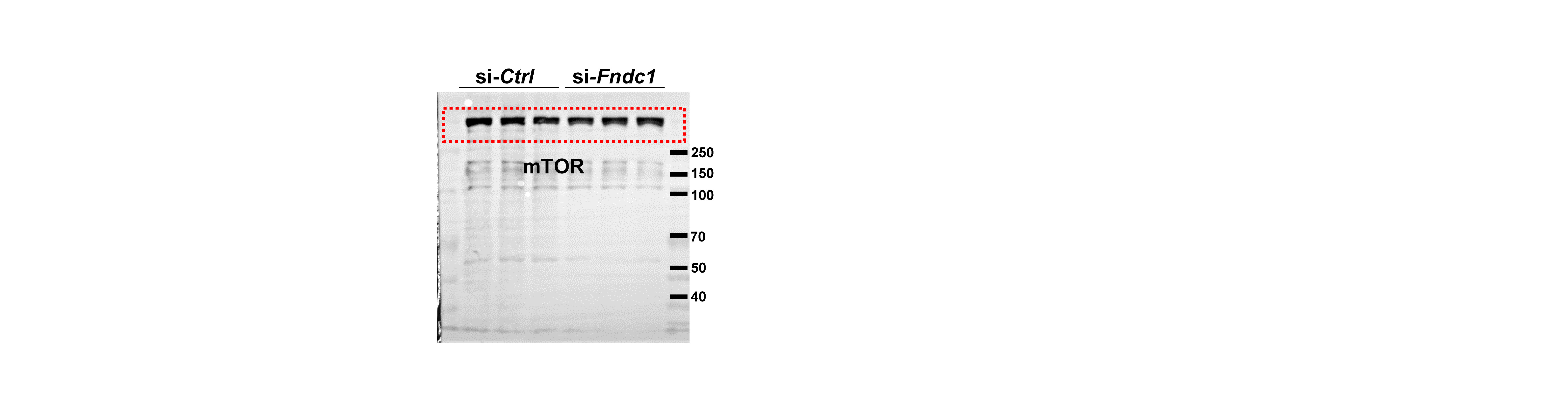

Supplement: Supplementary file 8 — Source data Fig. 5 [file 44318_2024_285_MOESM8_ESM.zip › Fig 5/Fig 5C/5-C-mTOR.tif]

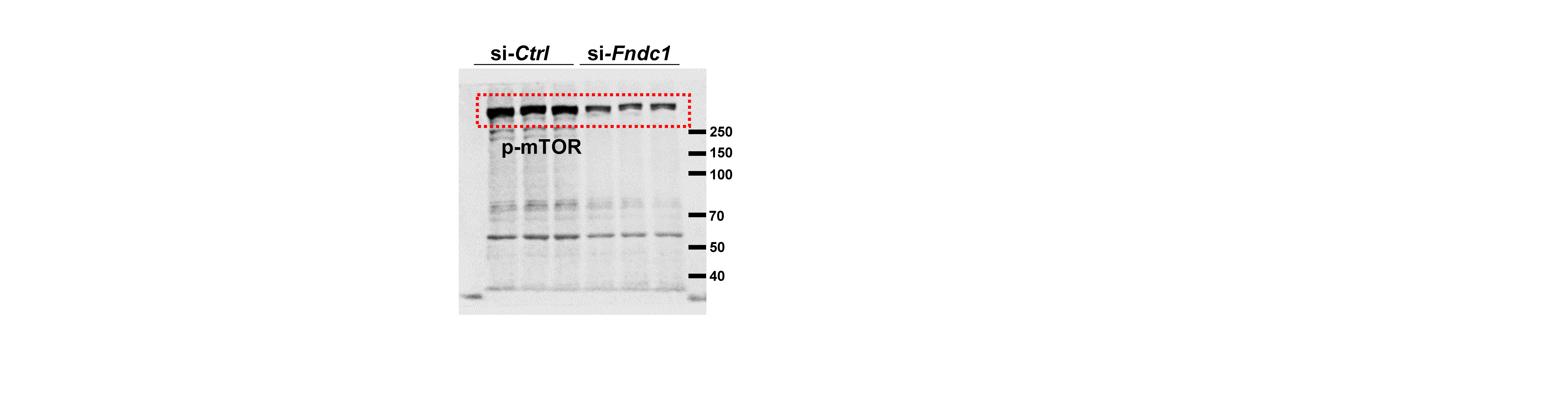

Supplement: Supplementary file 8 — Source data Fig. 5 [file 44318_2024_285_MOESM8_ESM.zip › Fig 5/Fig 5C/5-C-p-mTOR.tif]

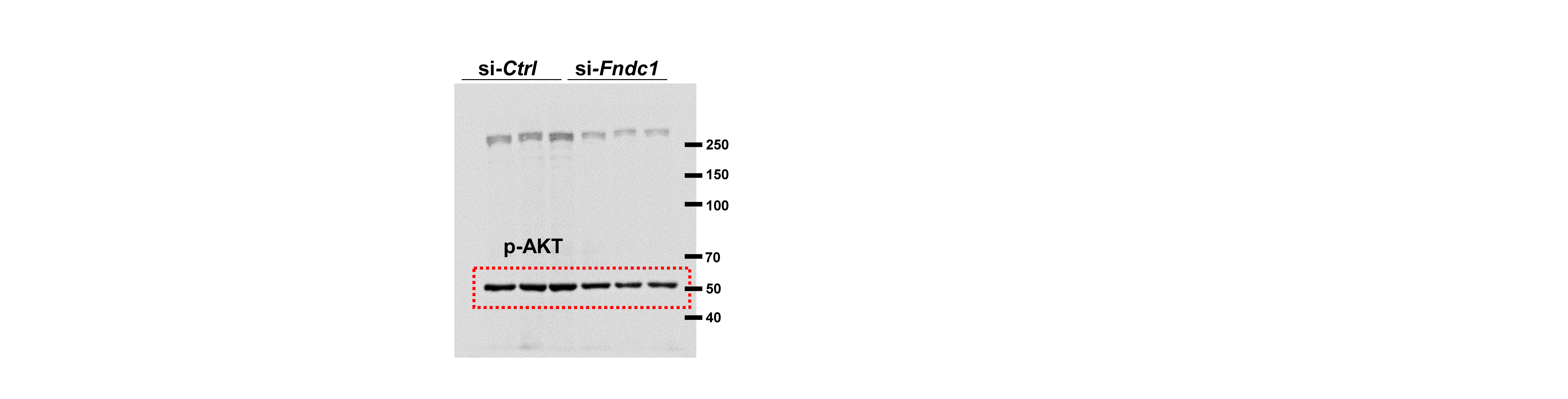

Supplement: Supplementary file 8 — Source data Fig. 5 [file 44318_2024_285_MOESM8_ESM.zip › Fig 5/Fig 5C/5-C-pAKT.tif]

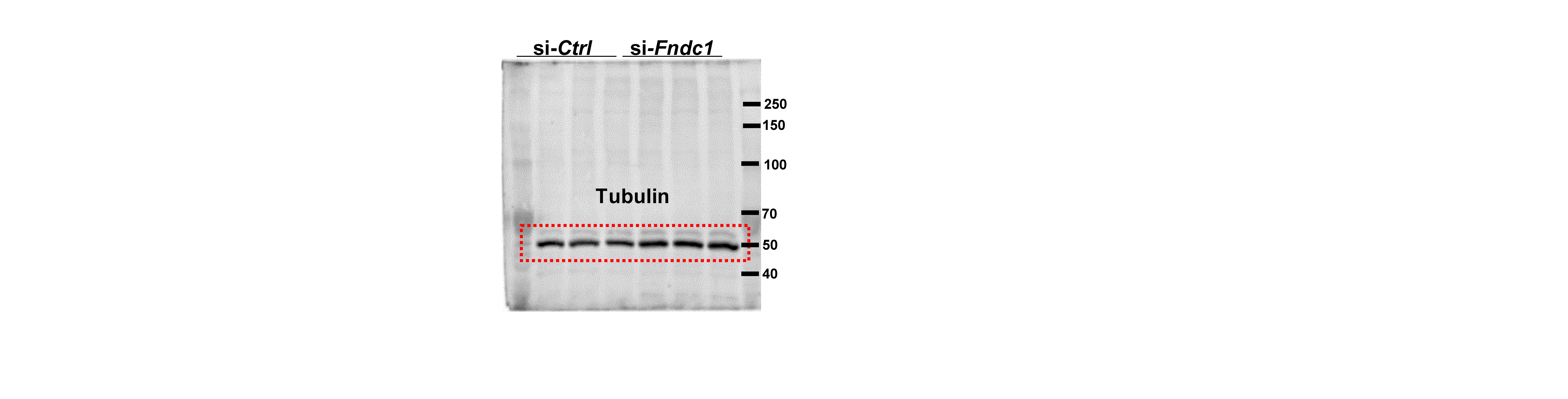

Supplement: Supplementary file 8 — Source data Fig. 5 [file 44318_2024_285_MOESM8_ESM.zip › Fig 5/Fig 5C/5-C-TUBULIN.tif]

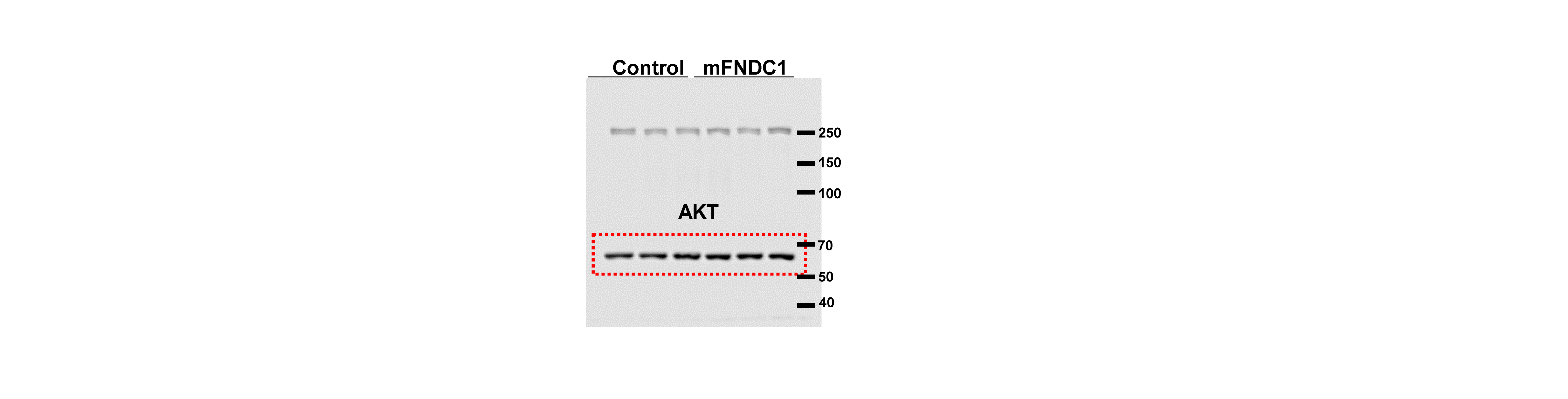

Supplement: Supplementary file 8 — Source data Fig. 5 [file 44318_2024_285_MOESM8_ESM.zip › Fig 5/Fig 5D/5-D-AKT.tif]

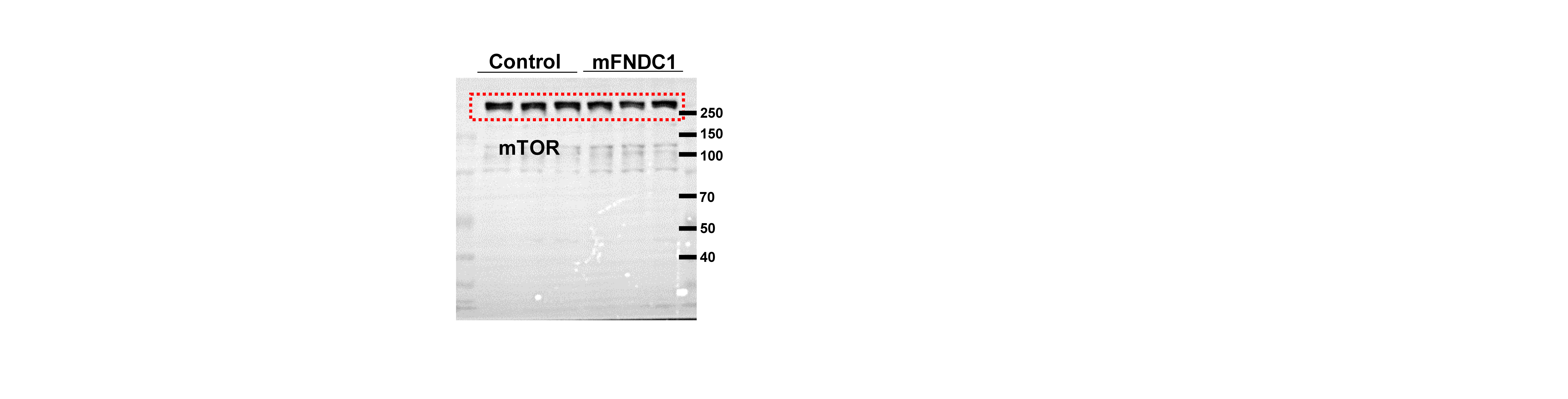

Supplement: Supplementary file 8 — Source data Fig. 5 [file 44318_2024_285_MOESM8_ESM.zip › Fig 5/Fig 5D/5-D-mTOR.tif]

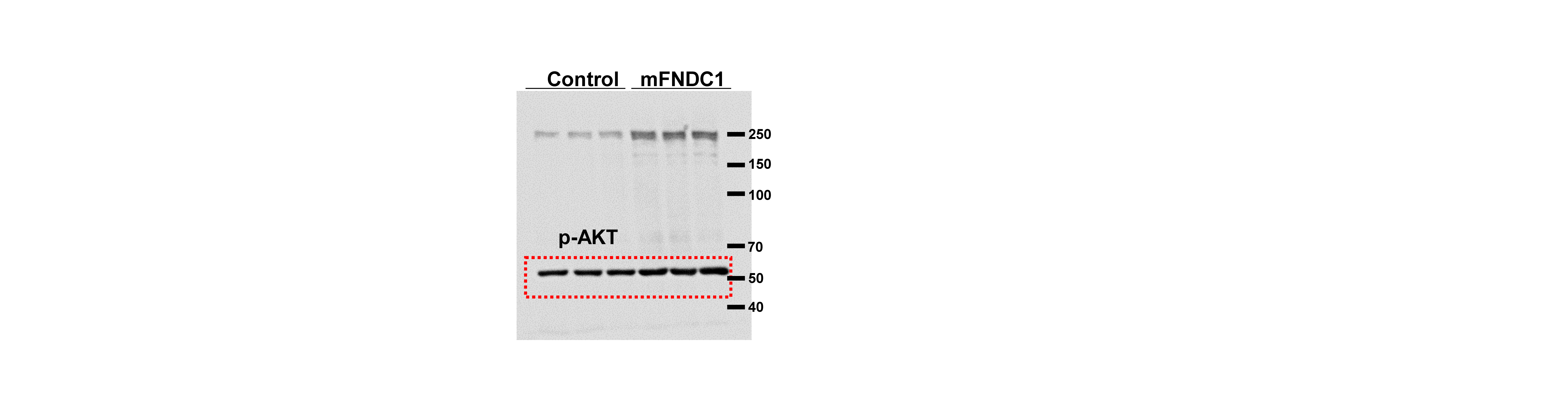

Supplement: Supplementary file 8 — Source data Fig. 5 [file 44318_2024_285_MOESM8_ESM.zip › Fig 5/Fig 5D/5-D-p-AKT.tif]

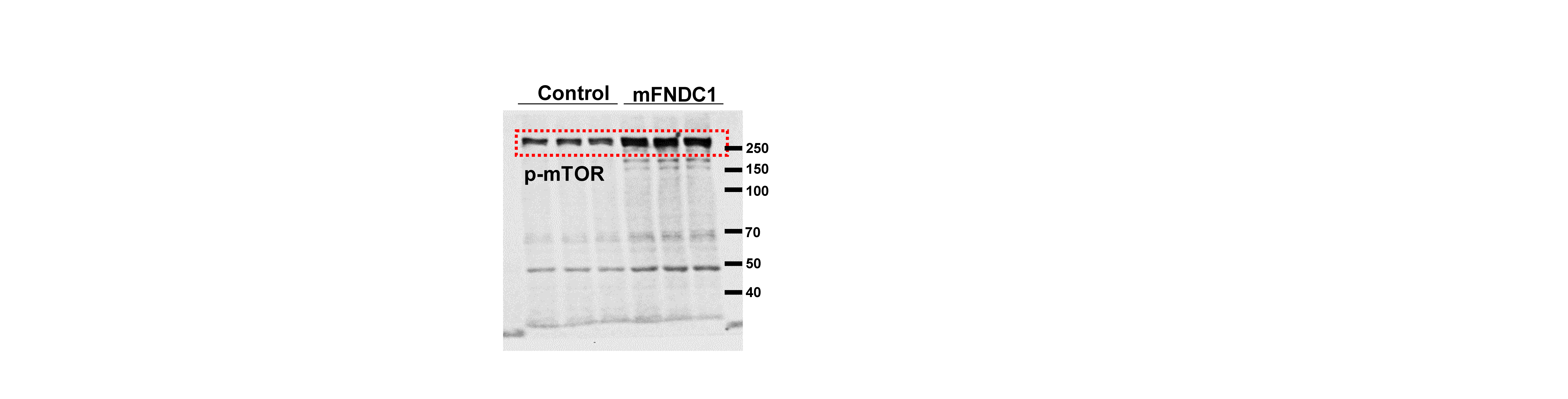

Supplement: Supplementary file 8 — Source data Fig. 5 [file 44318_2024_285_MOESM8_ESM.zip › Fig 5/Fig 5D/5-D-p-mTOR.tif]

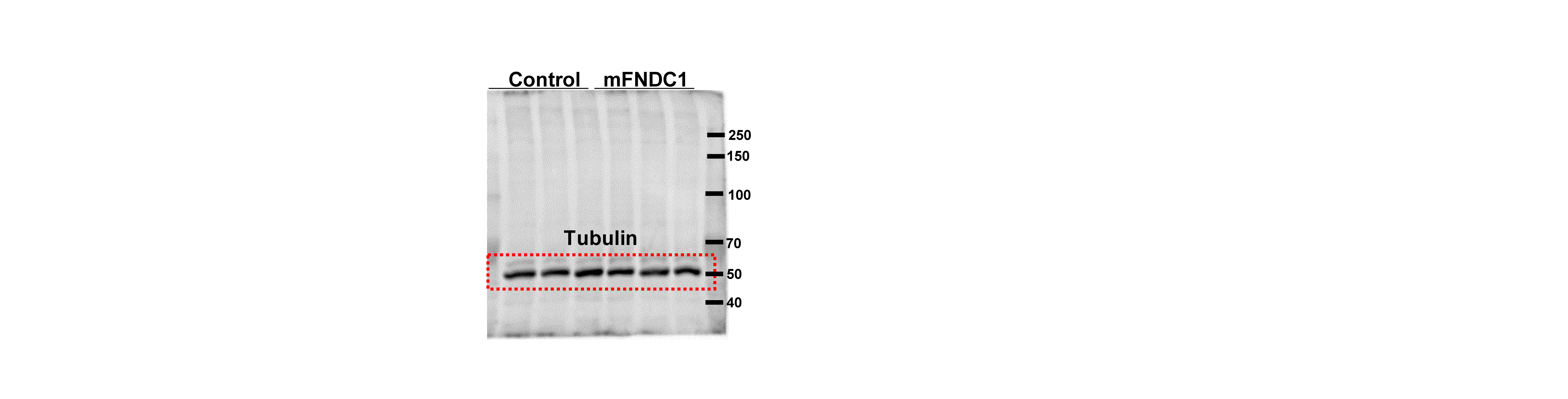

Supplement: Supplementary file 8 — Source data Fig. 5 [file 44318_2024_285_MOESM8_ESM.zip › Fig 5/Fig 5D/5-D-TUBULIN.tif]

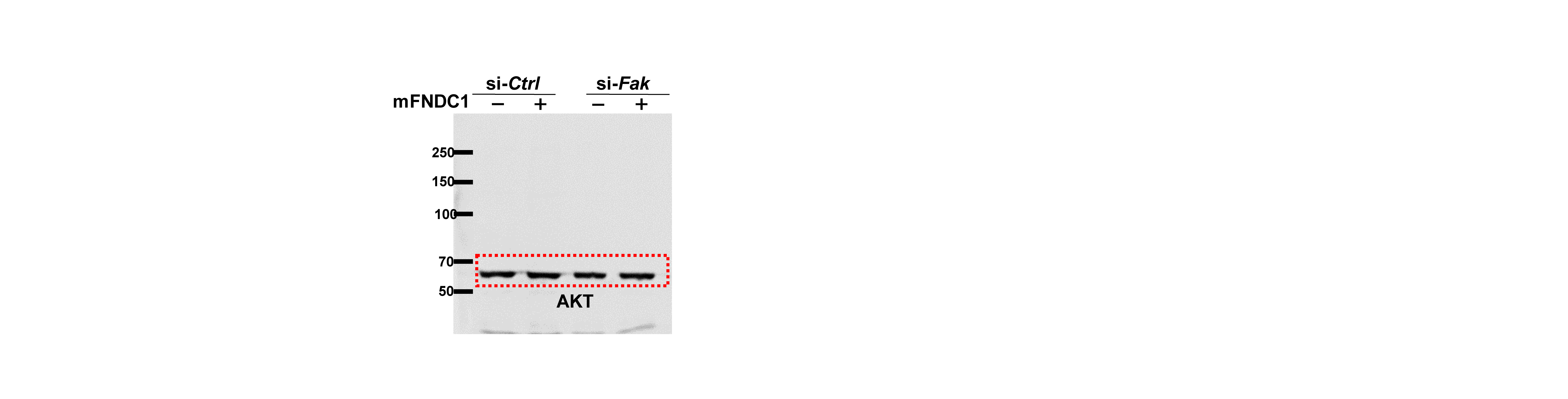

Supplement: Supplementary file 8 — Source data Fig. 5 [file 44318_2024_285_MOESM8_ESM.zip › Fig 5/Fig 5E/5-E-AKT.tif]

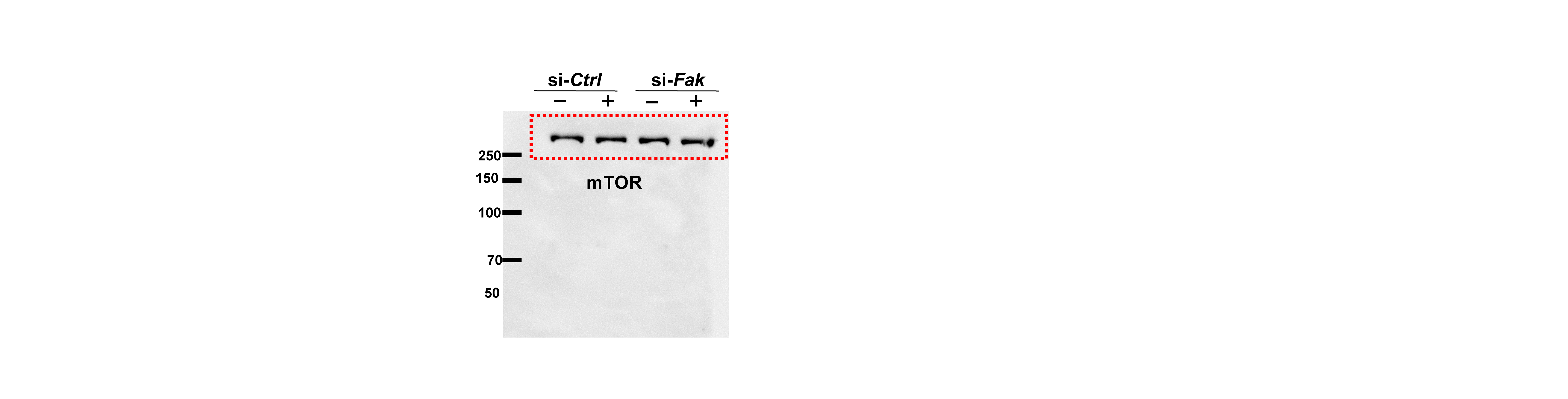

Supplement: Supplementary file 8 — Source data Fig. 5 [file 44318_2024_285_MOESM8_ESM.zip › Fig 5/Fig 5E/5-E-mTOR.tif]

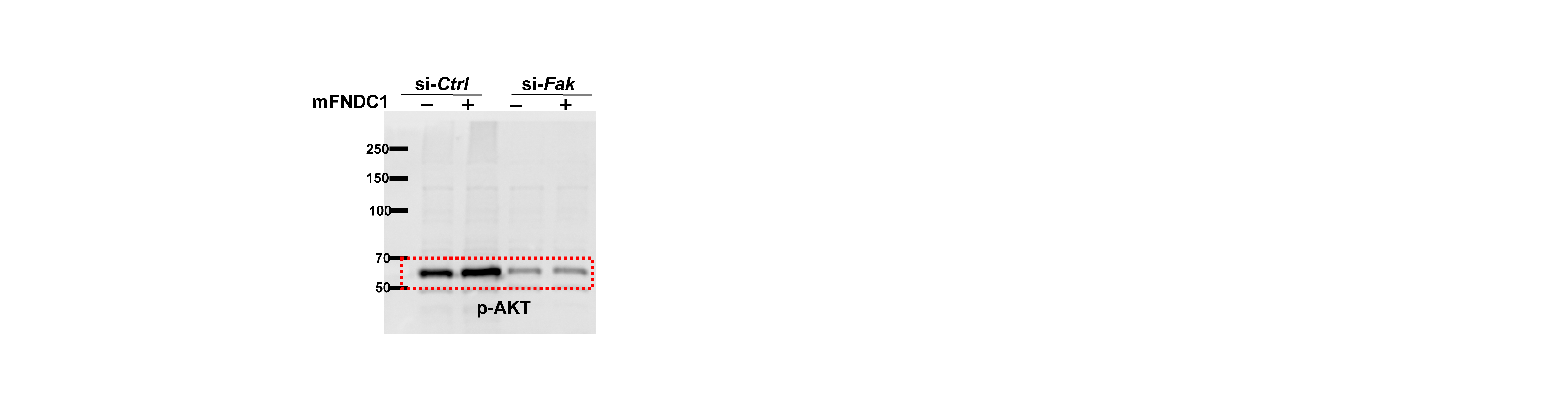

Supplement: Supplementary file 8 — Source data Fig. 5 [file 44318_2024_285_MOESM8_ESM.zip › Fig 5/Fig 5E/5-E-p-AKT.tif]

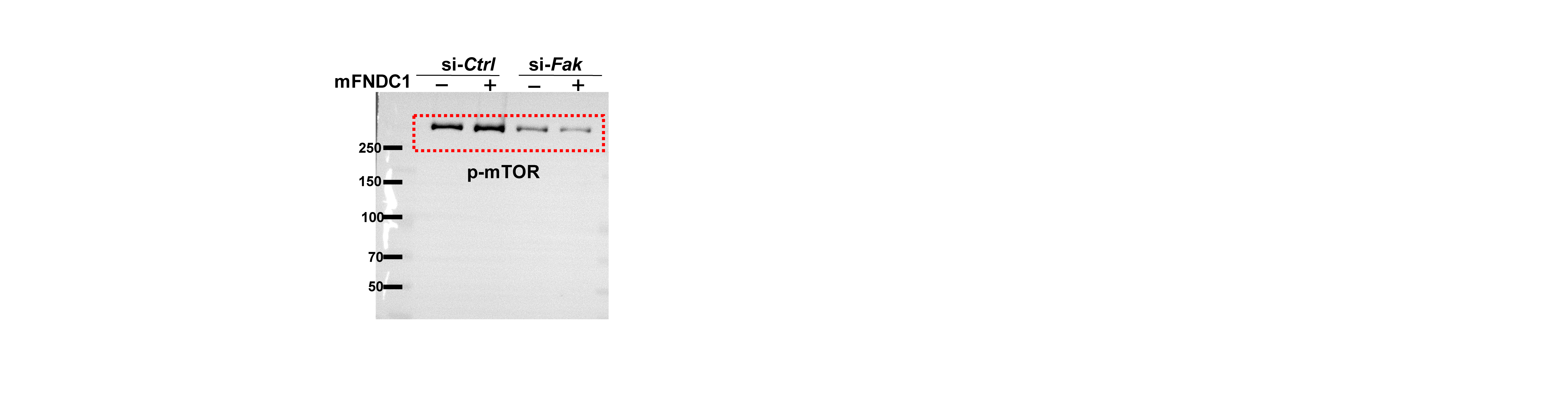

Supplement: Supplementary file 8 — Source data Fig. 5 [file 44318_2024_285_MOESM8_ESM.zip › Fig 5/Fig 5E/5-E-p-mTOR.tif]

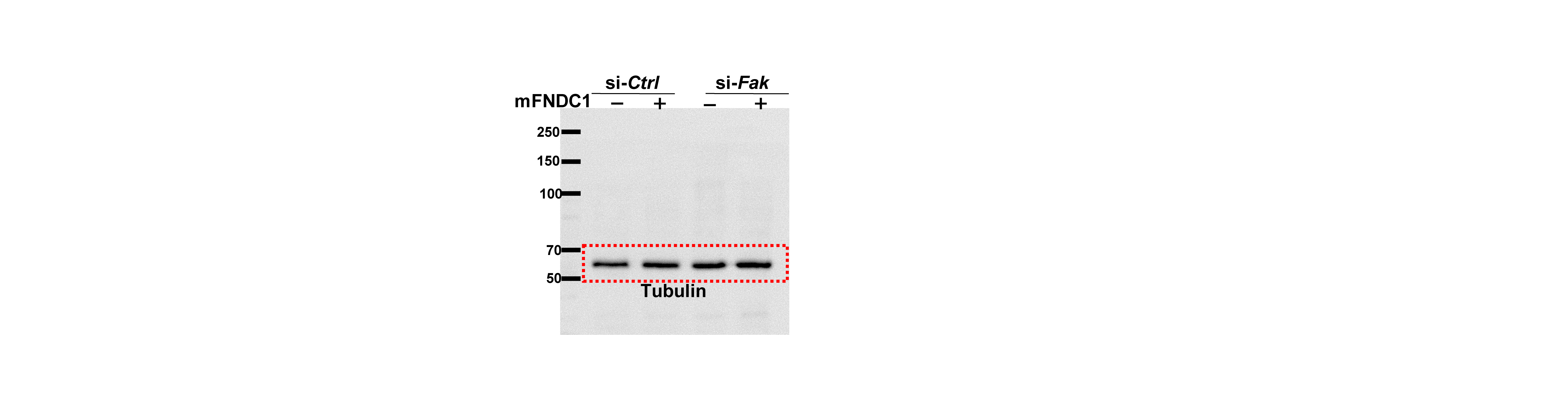

Supplement: Supplementary file 8 — Source data Fig. 5 [file 44318_2024_285_MOESM8_ESM.zip › Fig 5/Fig 5E/5-E-TUBULIN.tif]

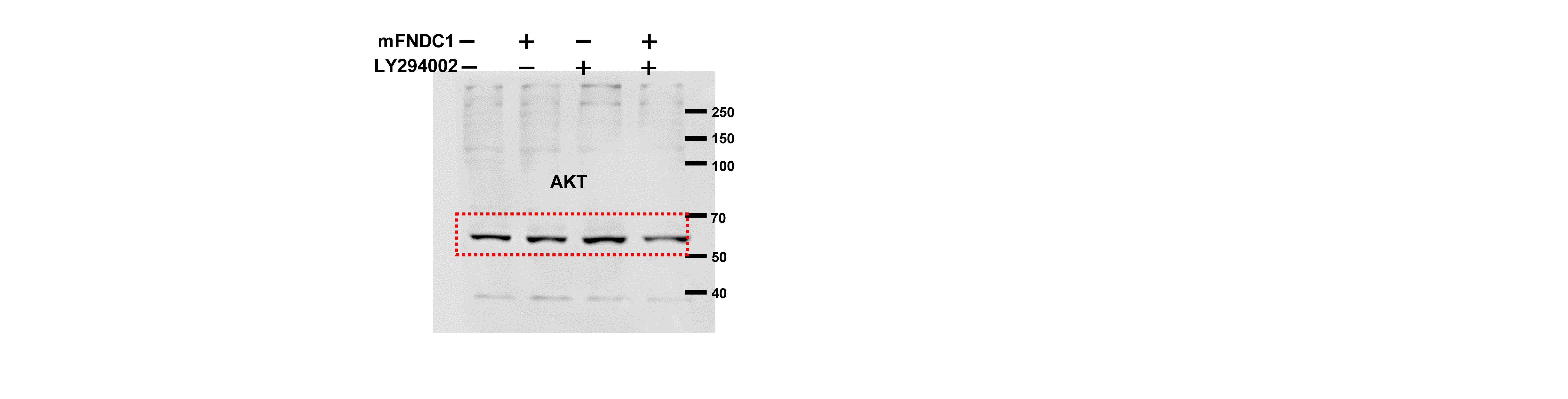

Supplement: Supplementary file 8 — Source data Fig. 5 [file 44318_2024_285_MOESM8_ESM.zip › Fig 5/Fig 5F/5-F-AKT.tif]

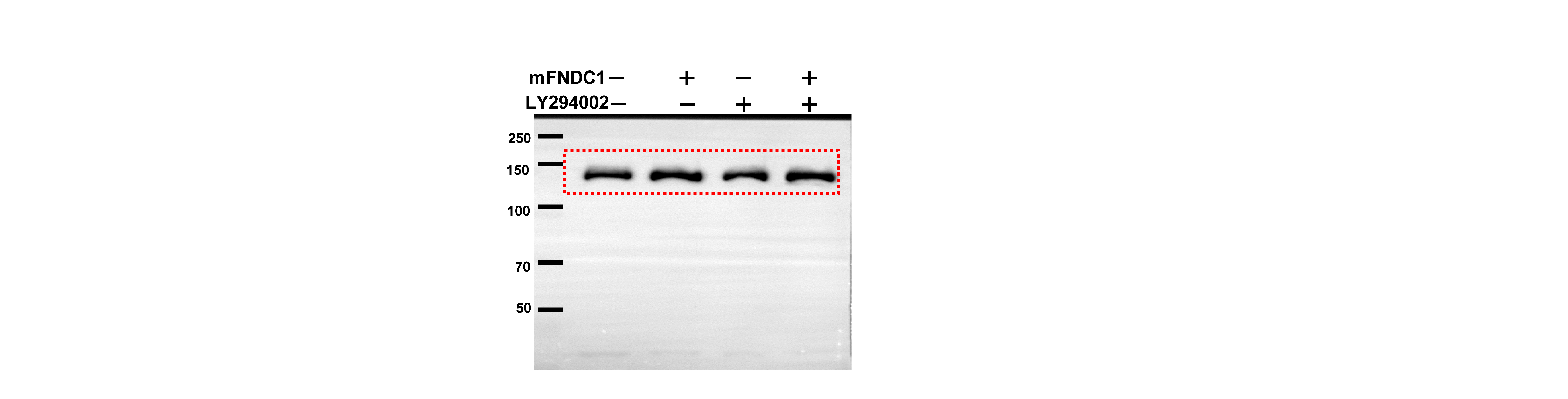

Supplement: Supplementary file 8 — Source data Fig. 5 [file 44318_2024_285_MOESM8_ESM.zip › Fig 5/Fig 5F/5-F-FAK.tif]

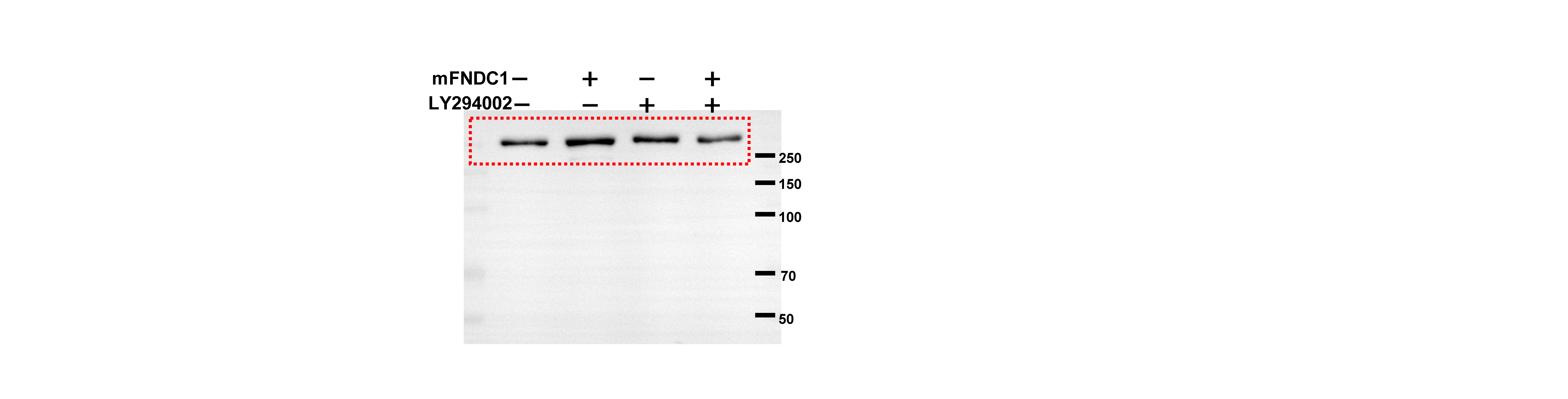

Supplement: Supplementary file 8 — Source data Fig. 5 [file 44318_2024_285_MOESM8_ESM.zip › Fig 5/Fig 5F/5-F-mTOR.tif]

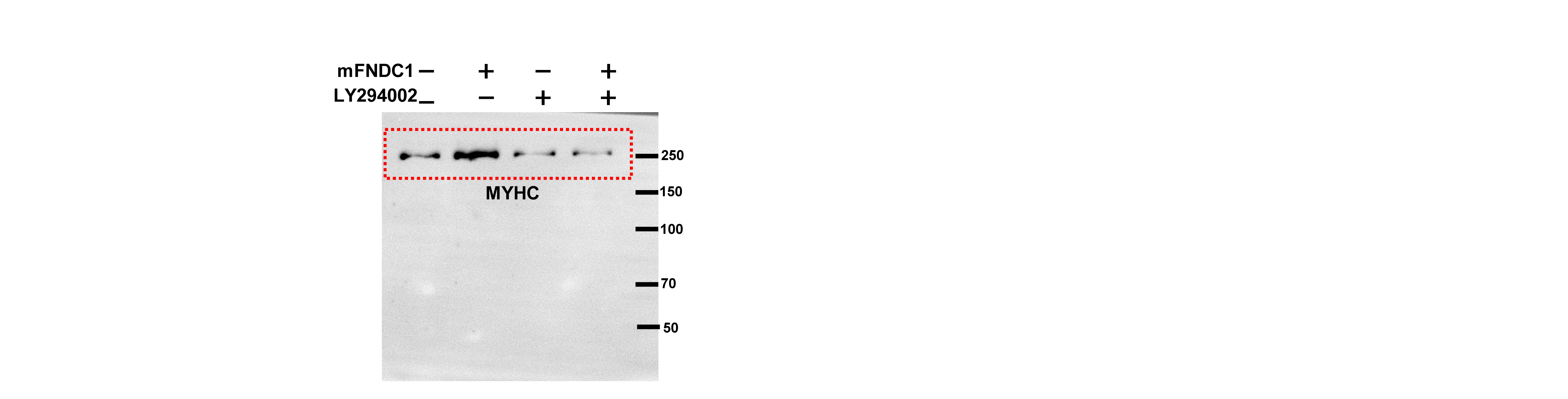

Supplement: Supplementary file 8 — Source data Fig. 5 [file 44318_2024_285_MOESM8_ESM.zip › Fig 5/Fig 5F/5-F-MYHC.tif]

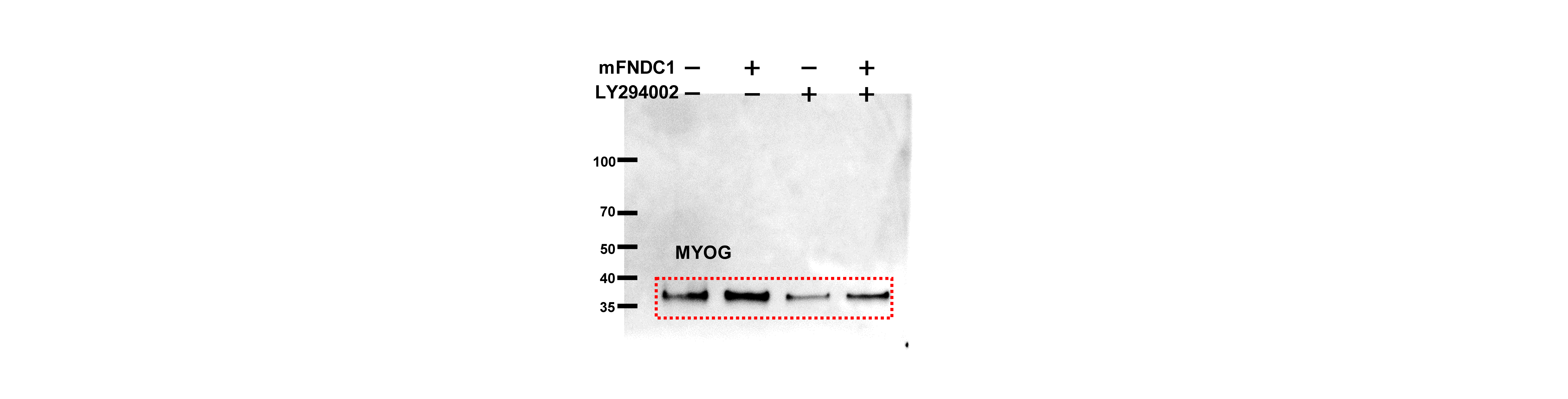

Supplement: Supplementary file 8 — Source data Fig. 5 [file 44318_2024_285_MOESM8_ESM.zip › Fig 5/Fig 5F/5-F-MYOG.tif]

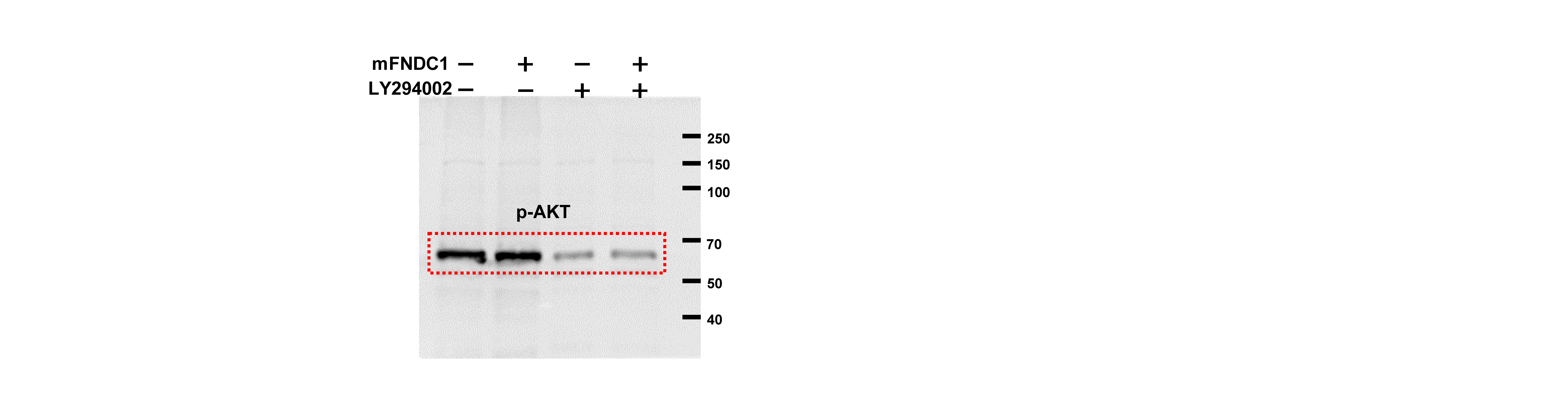

Supplement: Supplementary file 8 — Source data Fig. 5 [file 44318_2024_285_MOESM8_ESM.zip › Fig 5/Fig 5F/5-F-p-AKT.tif]

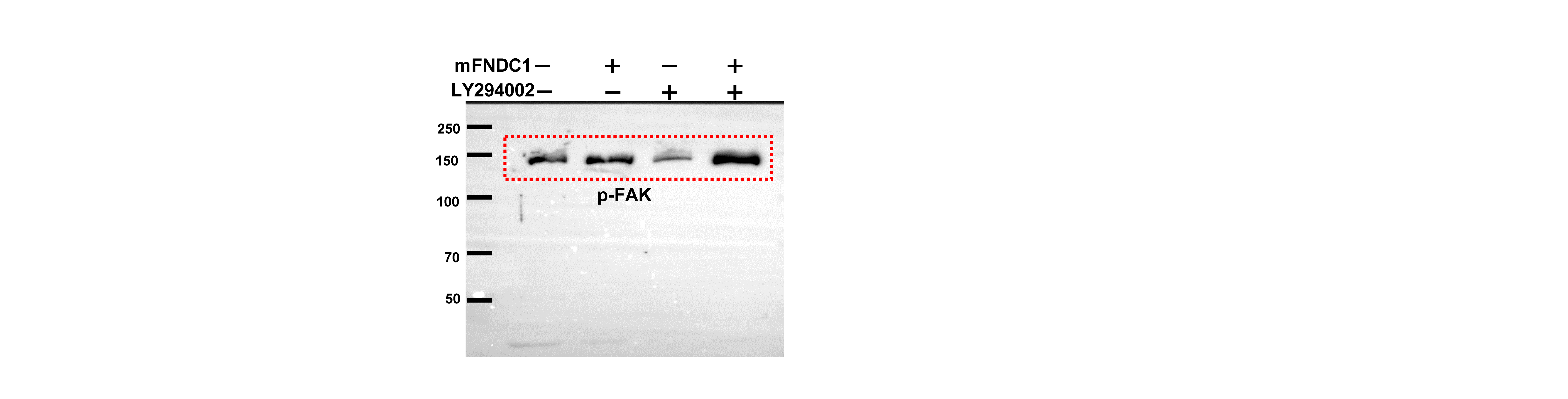

Supplement: Supplementary file 8 — Source data Fig. 5 [file 44318_2024_285_MOESM8_ESM.zip › Fig 5/Fig 5F/5-F-p-FAK.tif]

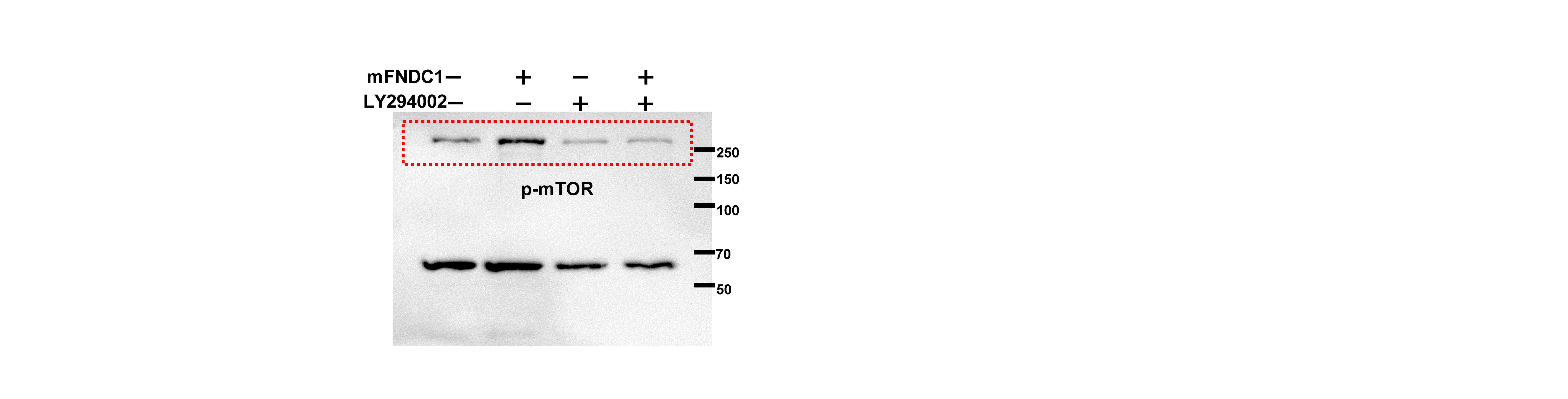

Supplement: Supplementary file 8 — Source data Fig. 5 [file 44318_2024_285_MOESM8_ESM.zip › Fig 5/Fig 5F/5-F-p-mTOR.tif]

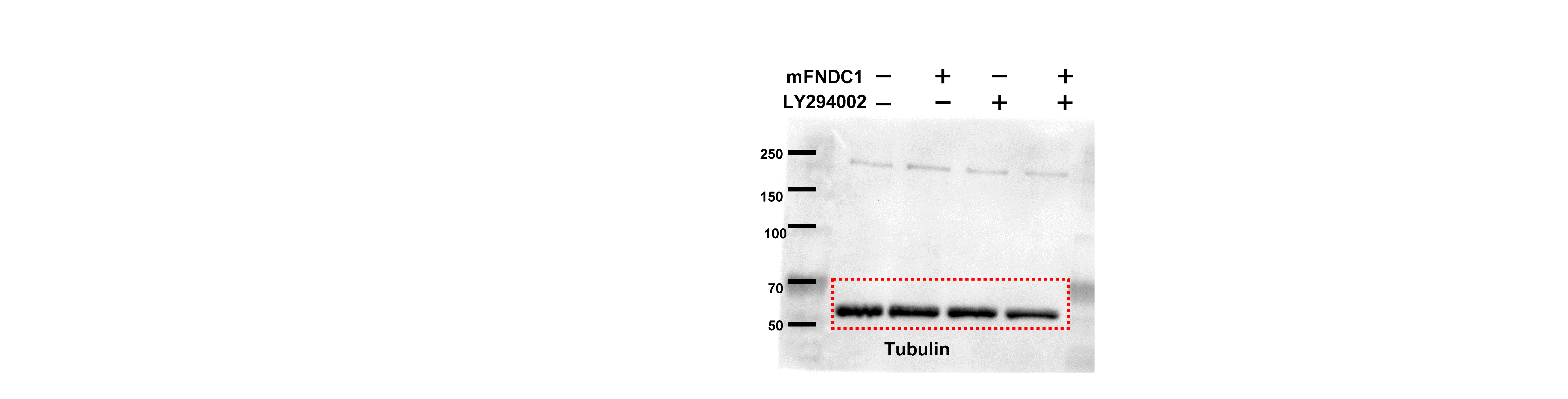

Supplement: Supplementary file 8 — Source data Fig. 5 [file 44318_2024_285_MOESM8_ESM.zip › Fig 5/Fig 5F/5-F-TUBULIN.tif]

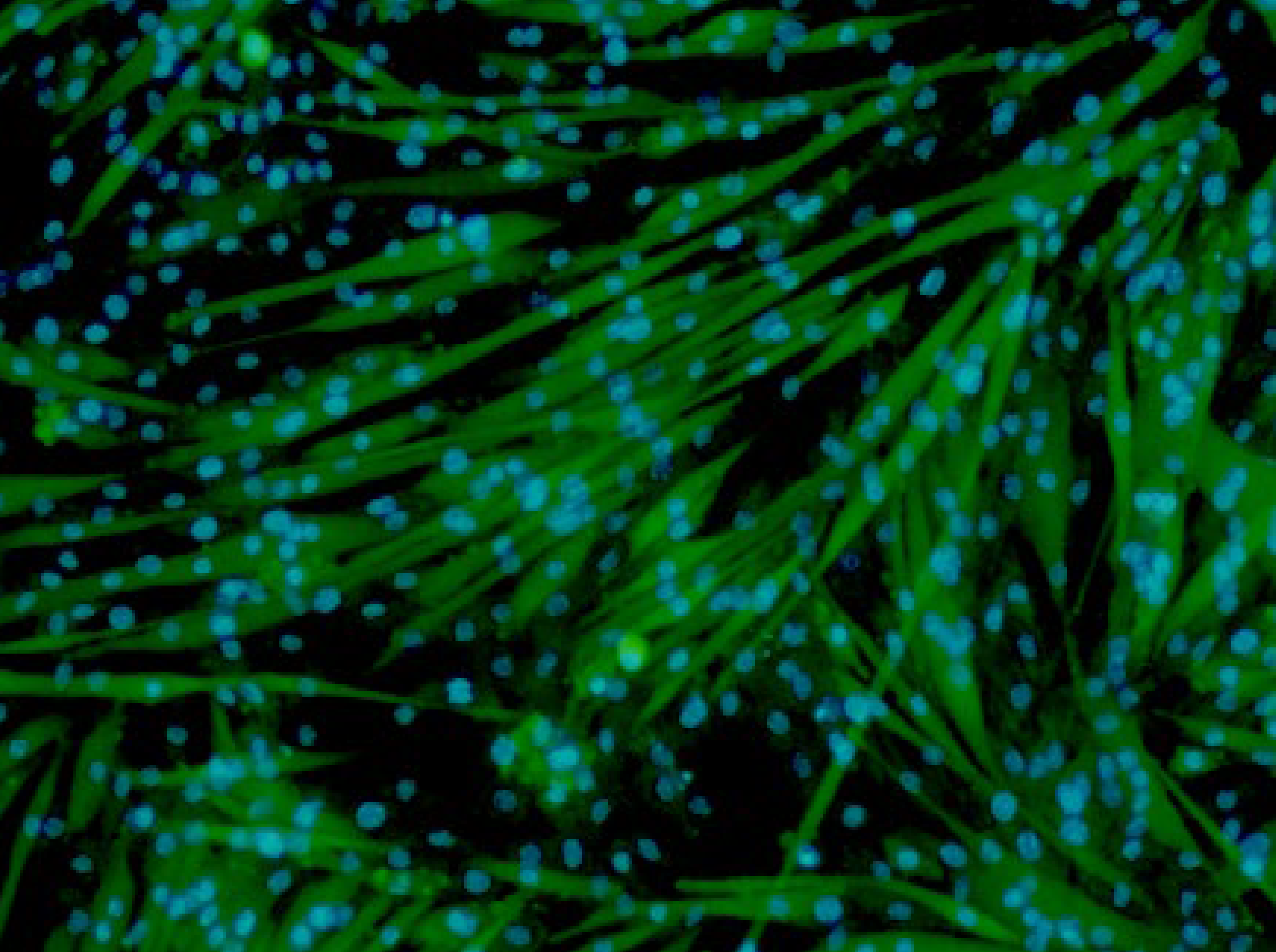

Supplement: Supplementary file 8 — Source data Fig. 5 [file 44318_2024_285_MOESM8_ESM.zip › Fig 5/Fig 5H/5H-Control+LY294002.tif]

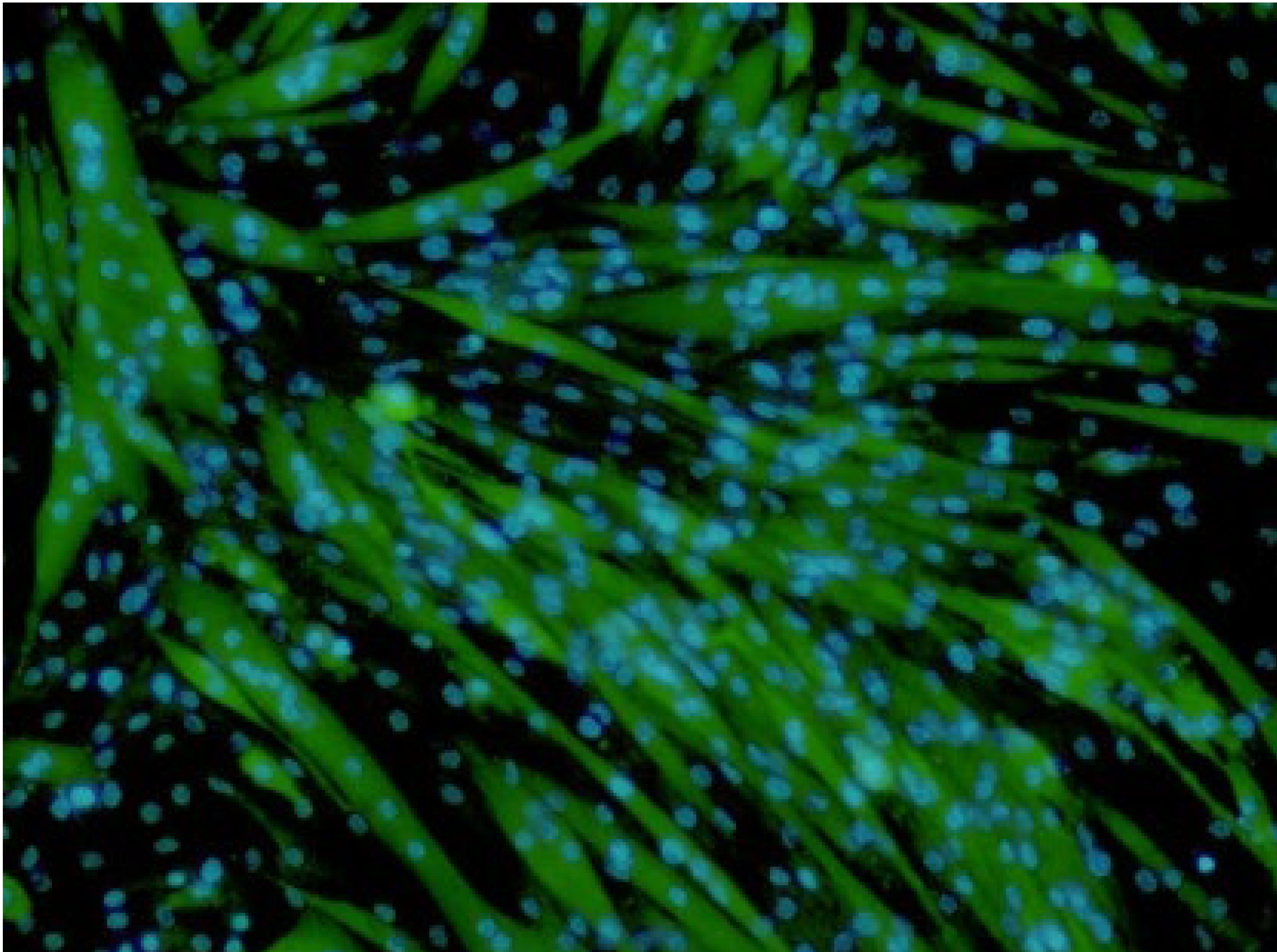

Supplement: Supplementary file 8 — Source data Fig. 5 [file 44318_2024_285_MOESM8_ESM.zip › Fig 5/Fig 5H/5H-Control-DMSO.tif]

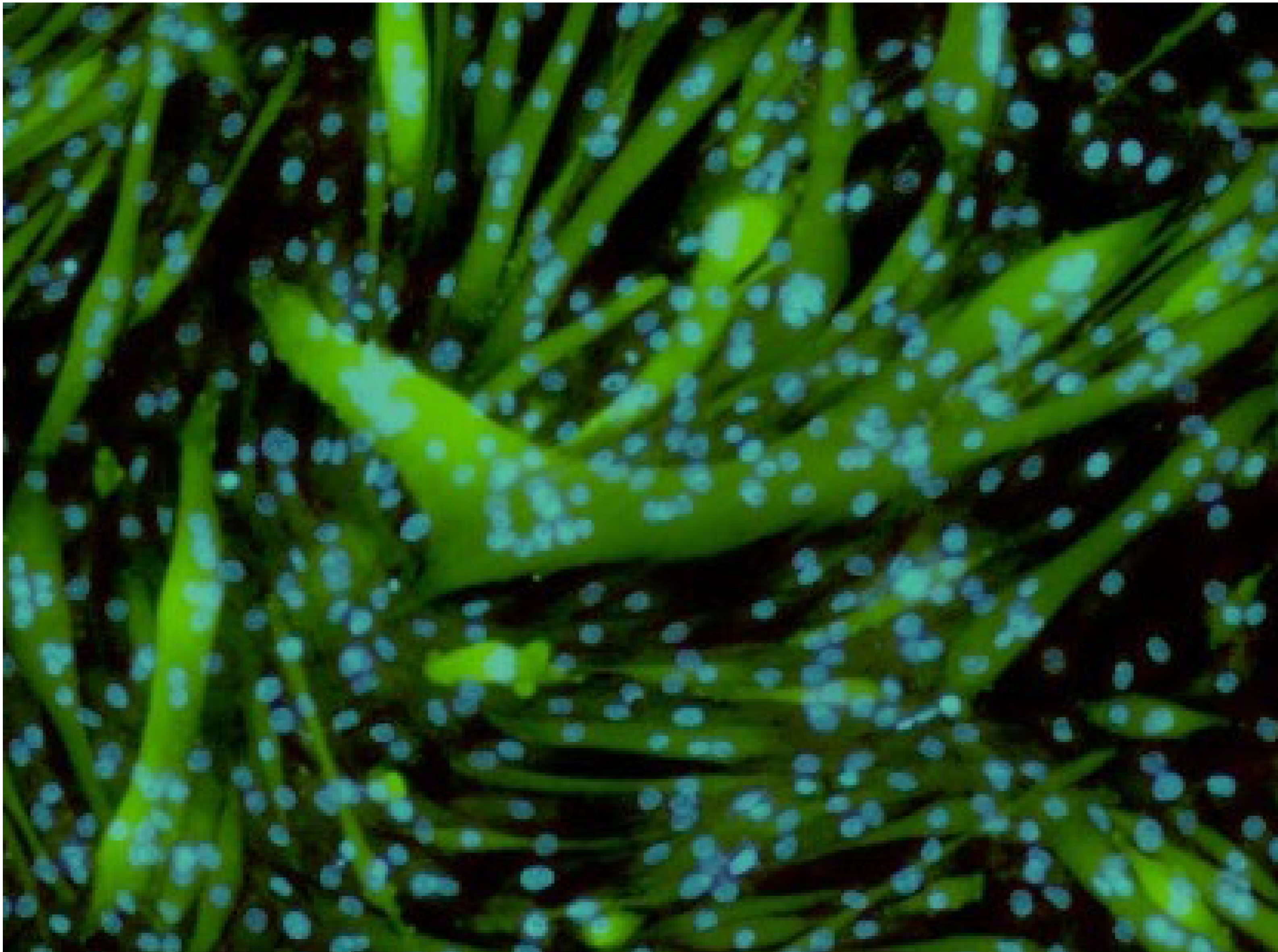

Supplement: Supplementary file 8 — Source data Fig. 5 [file 44318_2024_285_MOESM8_ESM.zip › Fig 5/Fig 5H/5H-mFNDC1+DMSO.tif]

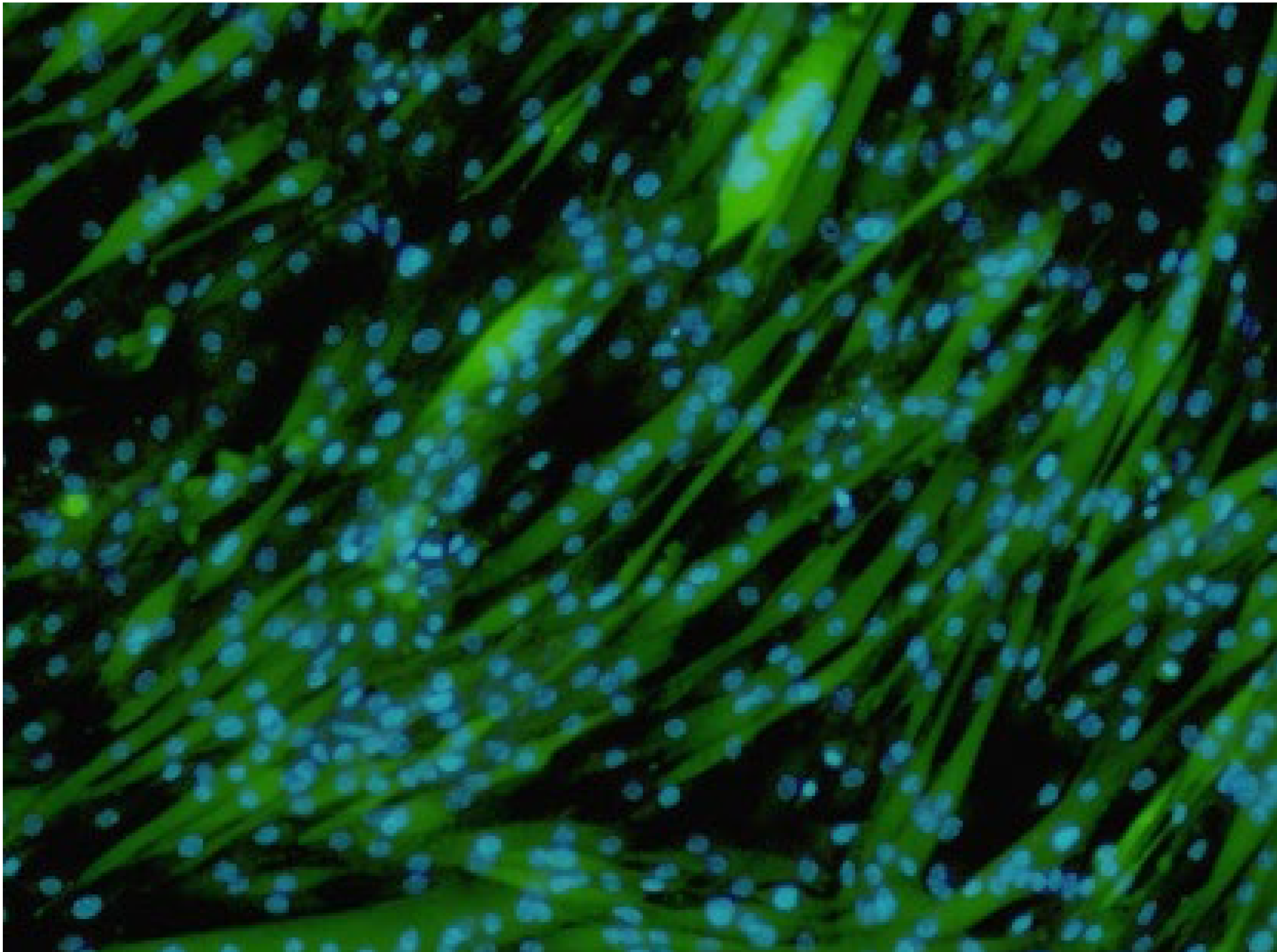

Supplement: Supplementary file 8 — Source data Fig. 5 [file 44318_2024_285_MOESM8_ESM.zip › Fig 5/Fig 5H/5H-mFNDC1+LY294002.tif]

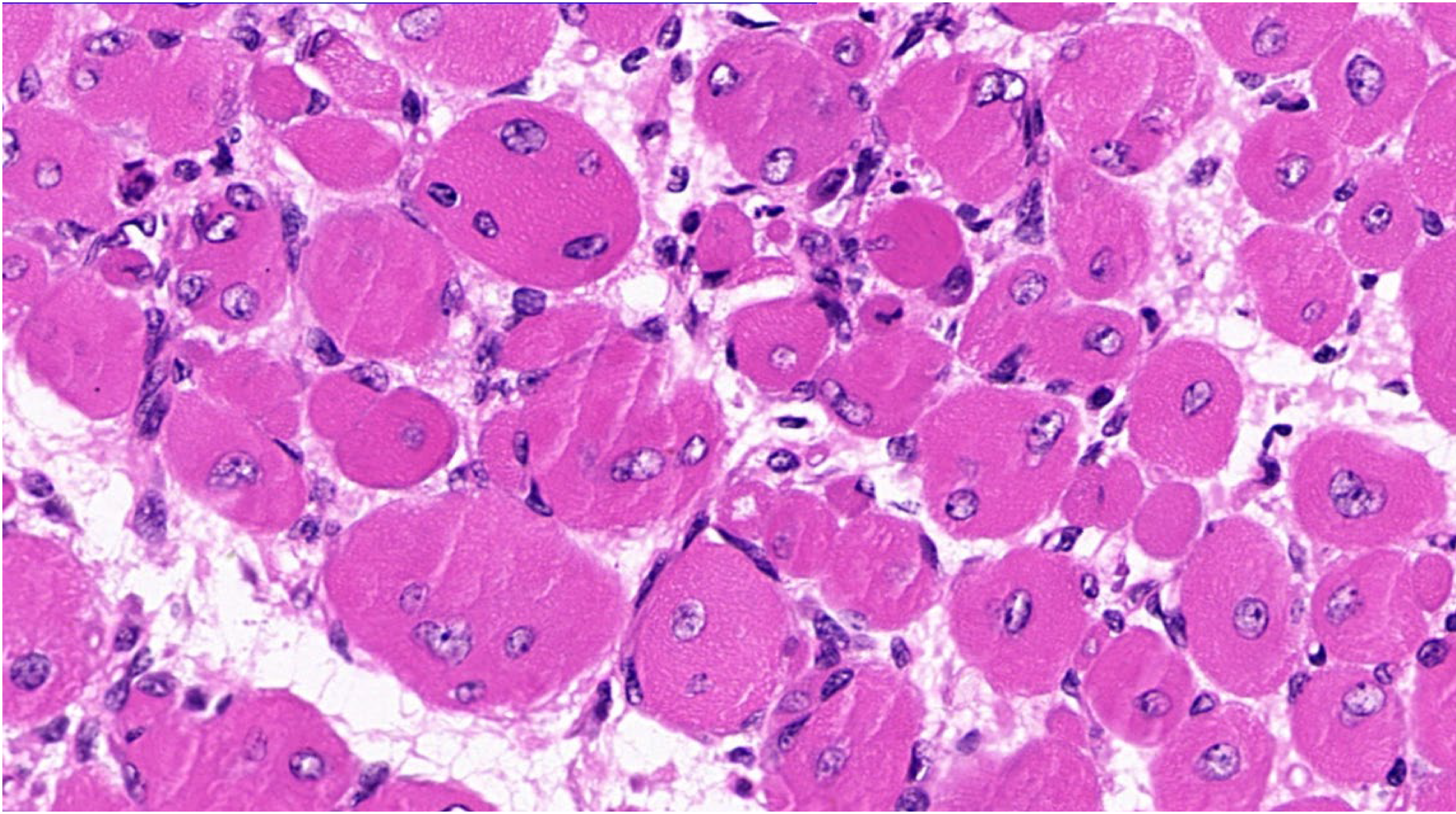

Supplement: Supplementary file 9 — Source data Fig. 6 [file 44318_2024_285_MOESM9_ESM.zip › Fig 6/Fig 6B/6B-Control-Day 14.tif]

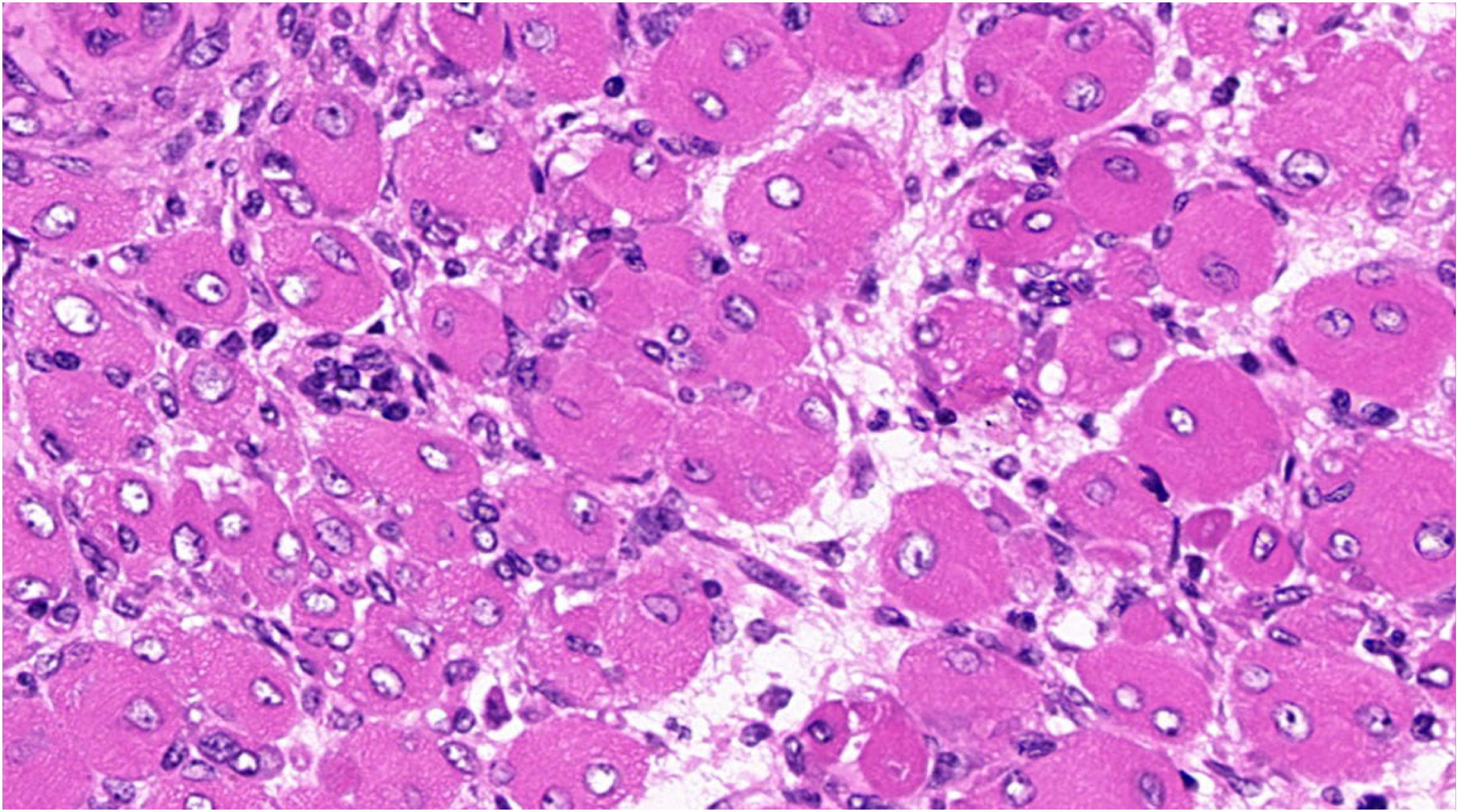

Supplement: Supplementary file 9 — Source data Fig. 6 [file 44318_2024_285_MOESM9_ESM.zip › Fig 6/Fig 6B/6B-Control-Day 5.tif]

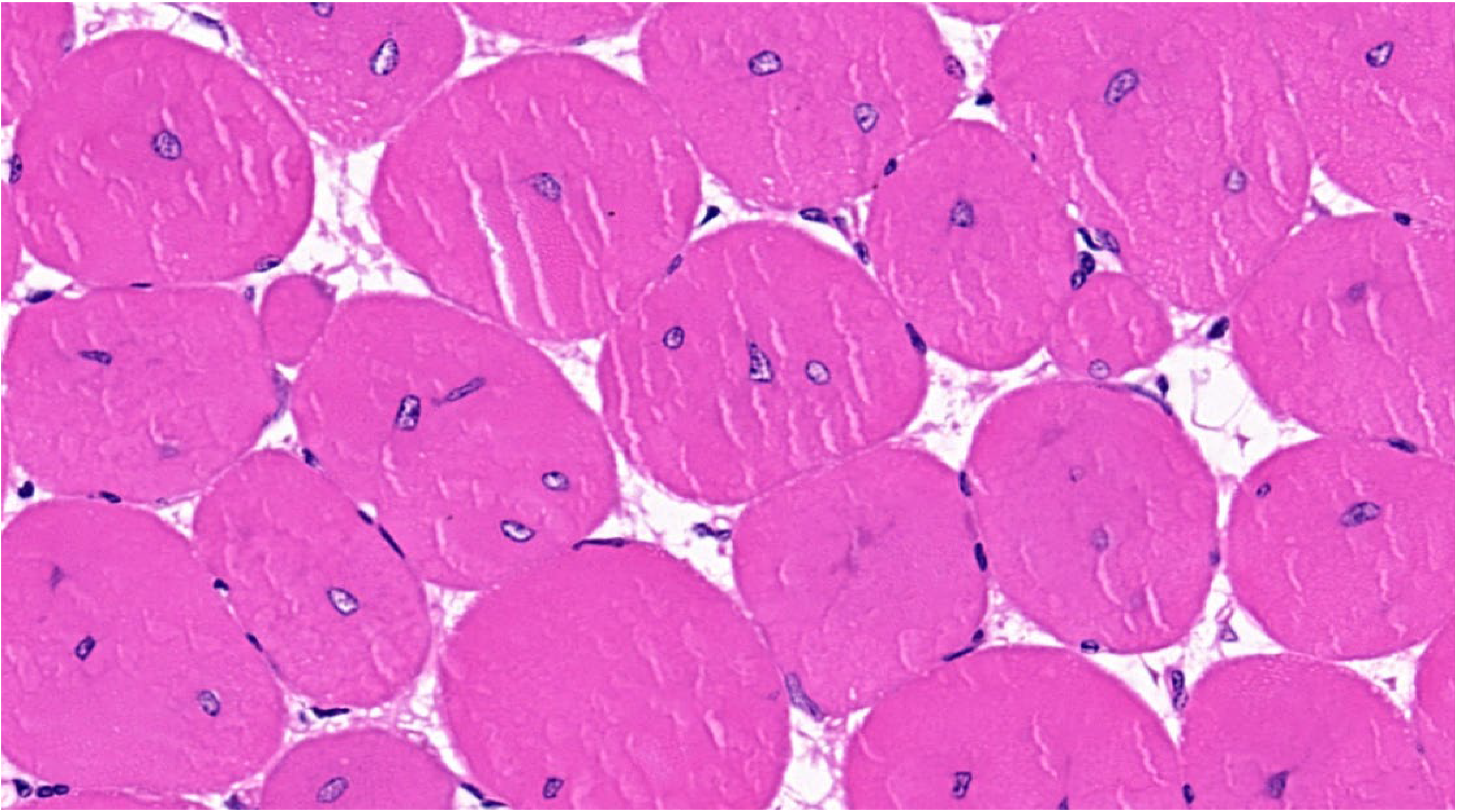

Supplement: Supplementary file 9 — Source data Fig. 6 [file 44318_2024_285_MOESM9_ESM.zip › Fig 6/Fig 6B/6B-mFNDC1-Day 14.tif]

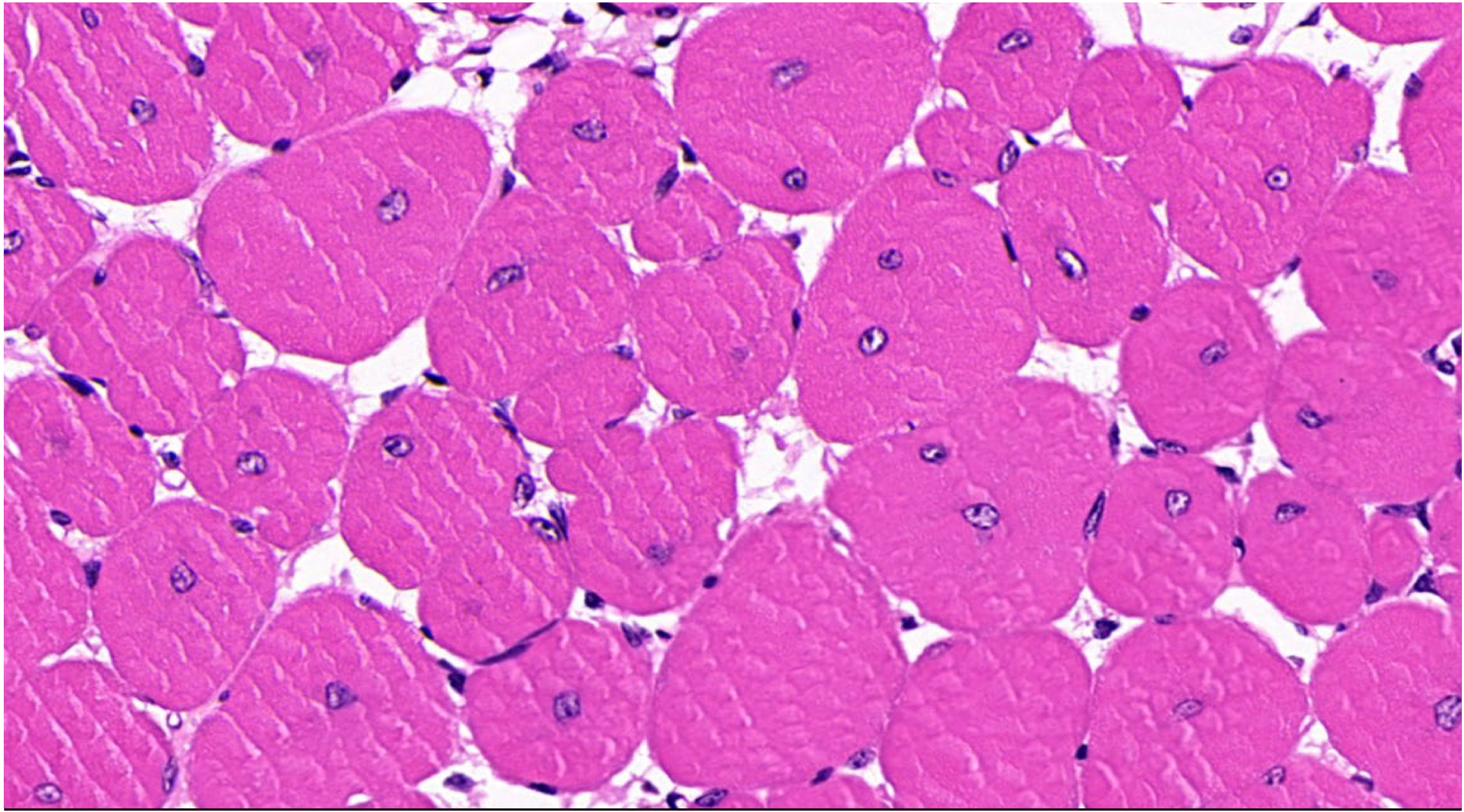

Supplement: Supplementary file 9 — Source data Fig. 6 [file 44318_2024_285_MOESM9_ESM.zip › Fig 6/Fig 6B/6B-mFNDC1-Day 5.tif]

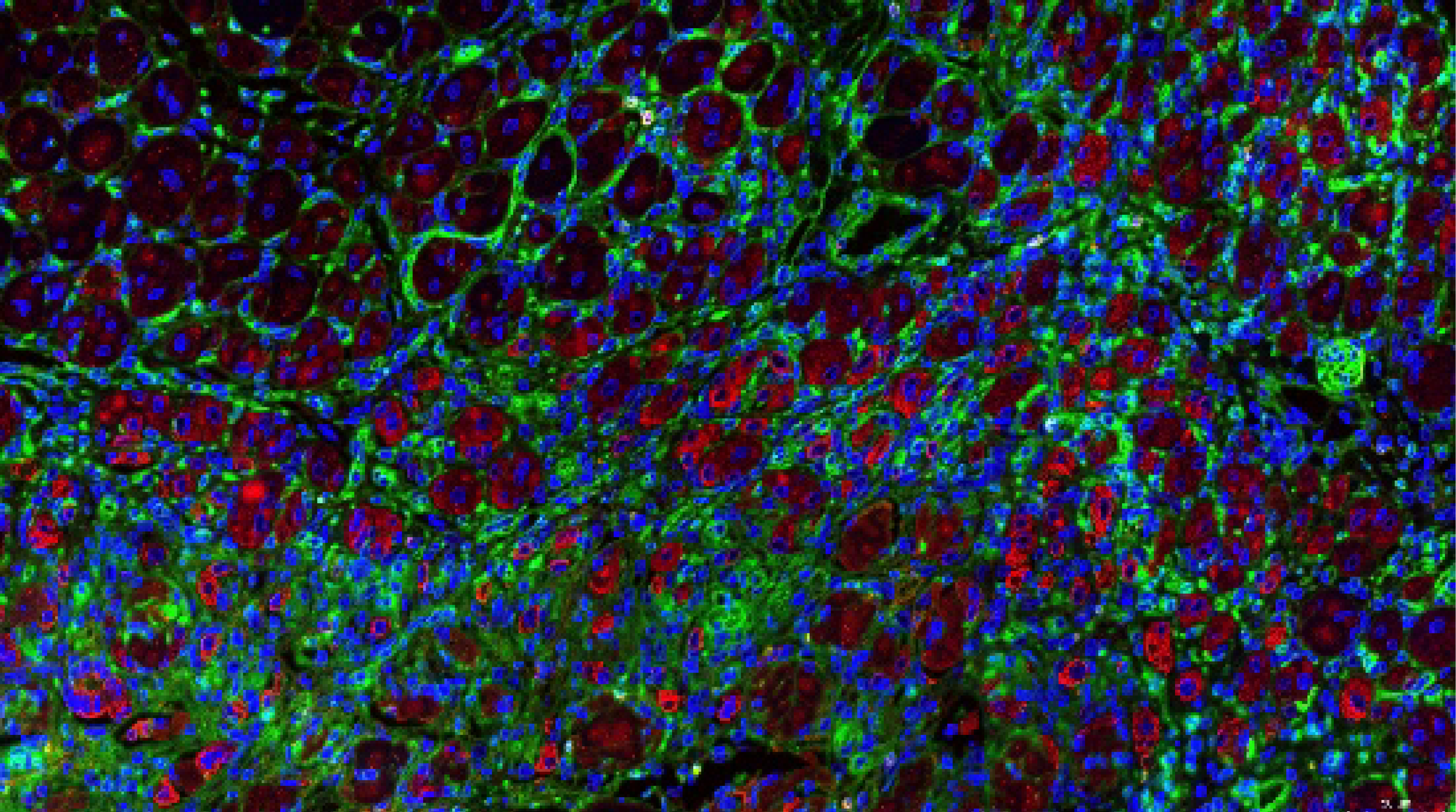

Supplement: Supplementary file 9 — Source data Fig. 6 [file 44318_2024_285_MOESM9_ESM.zip › Fig 6/Fig 6C/6C-Control-Day 5.tif]

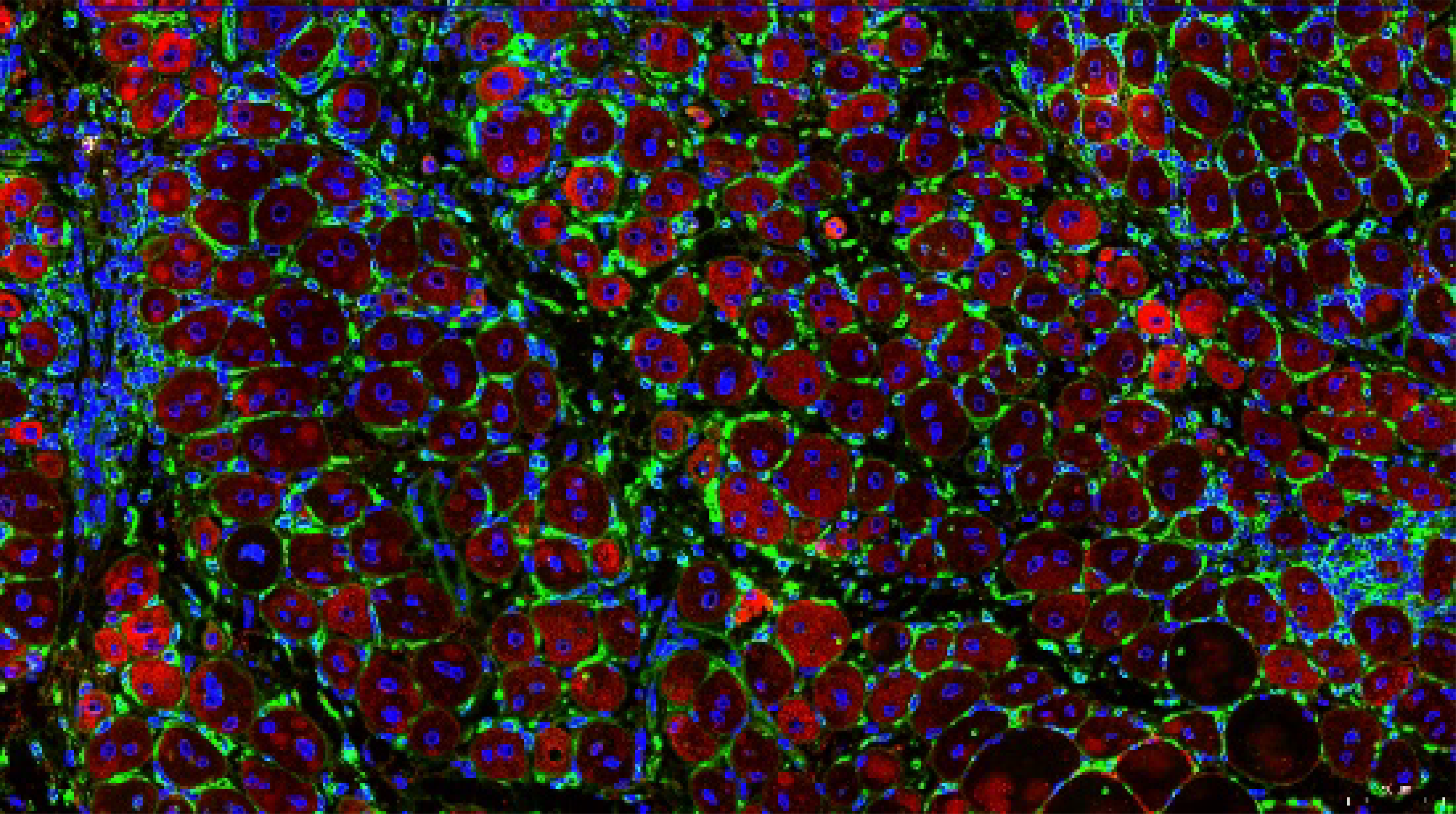

Supplement: Supplementary file 9 — Source data Fig. 6 [file 44318_2024_285_MOESM9_ESM.zip › Fig 6/Fig 6C/6C-mFNDC1-Day 5.tif]

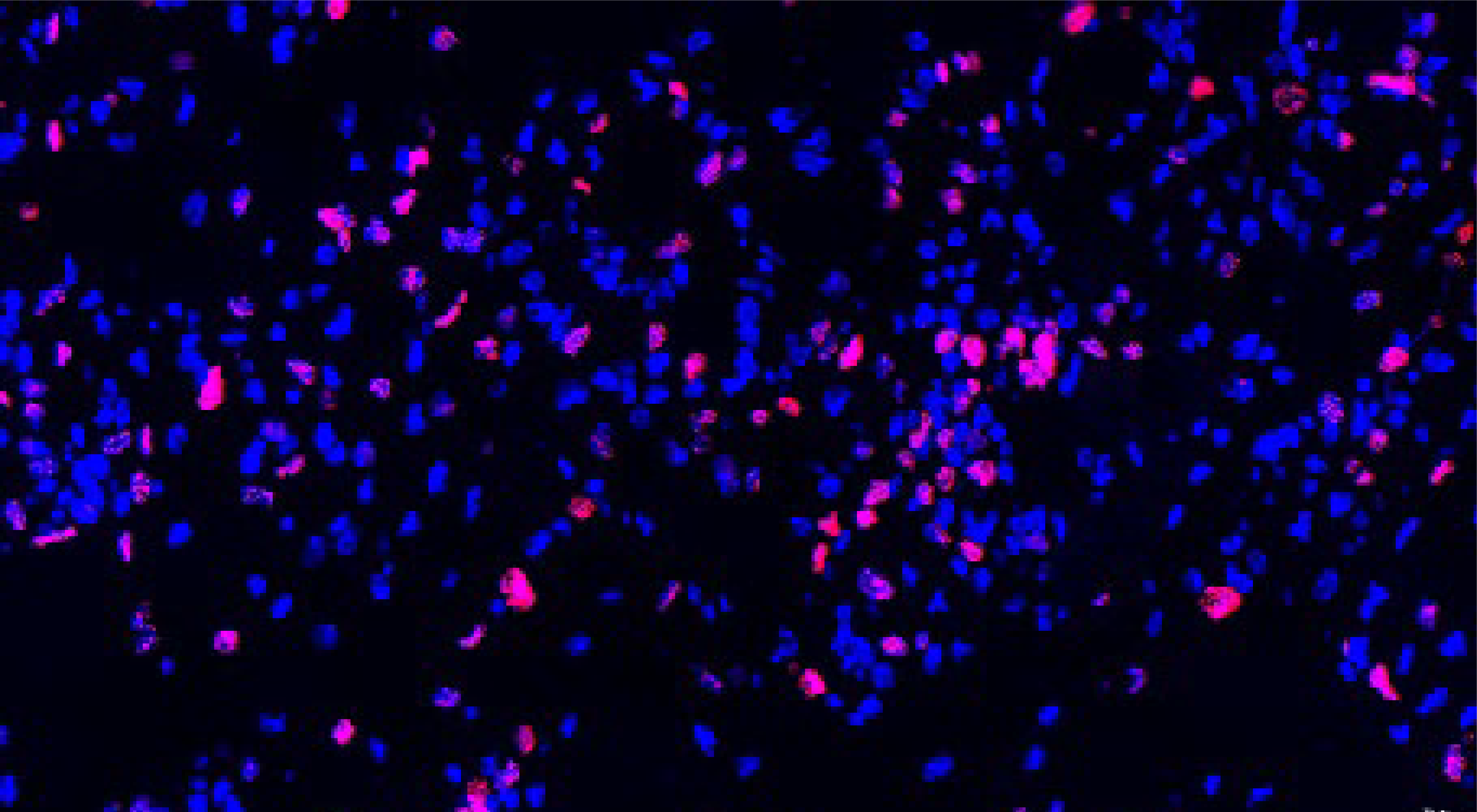

Supplement: Supplementary file 9 — Source data Fig. 6 [file 44318_2024_285_MOESM9_ESM.zip › Fig 6/Fig 6J/6J-Control-MYOG.tif]

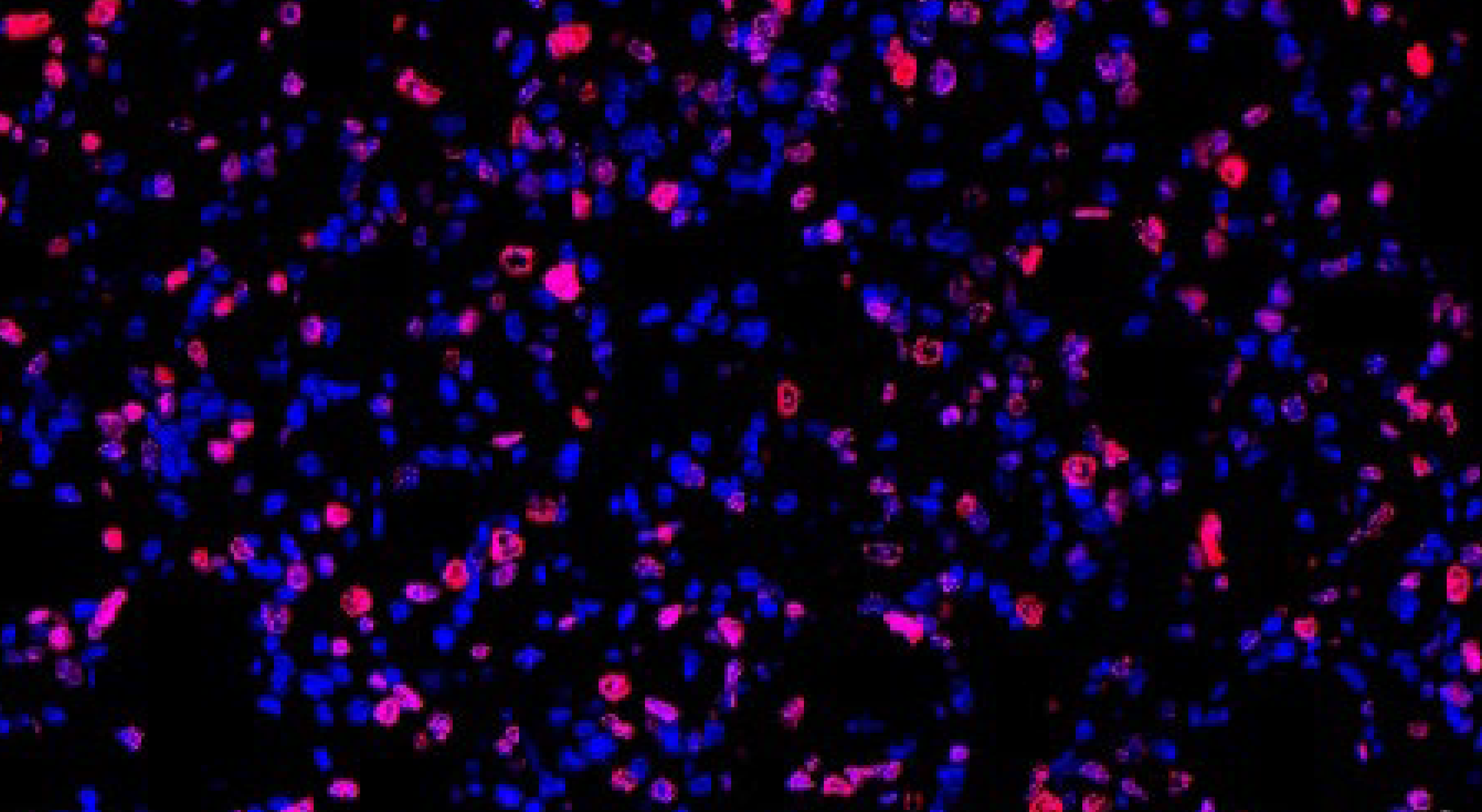

Supplement: Supplementary file 9 — Source data Fig. 6 [file 44318_2024_285_MOESM9_ESM.zip › Fig 6/Fig 6J/6J-mFNDC1-MYOG.tif]

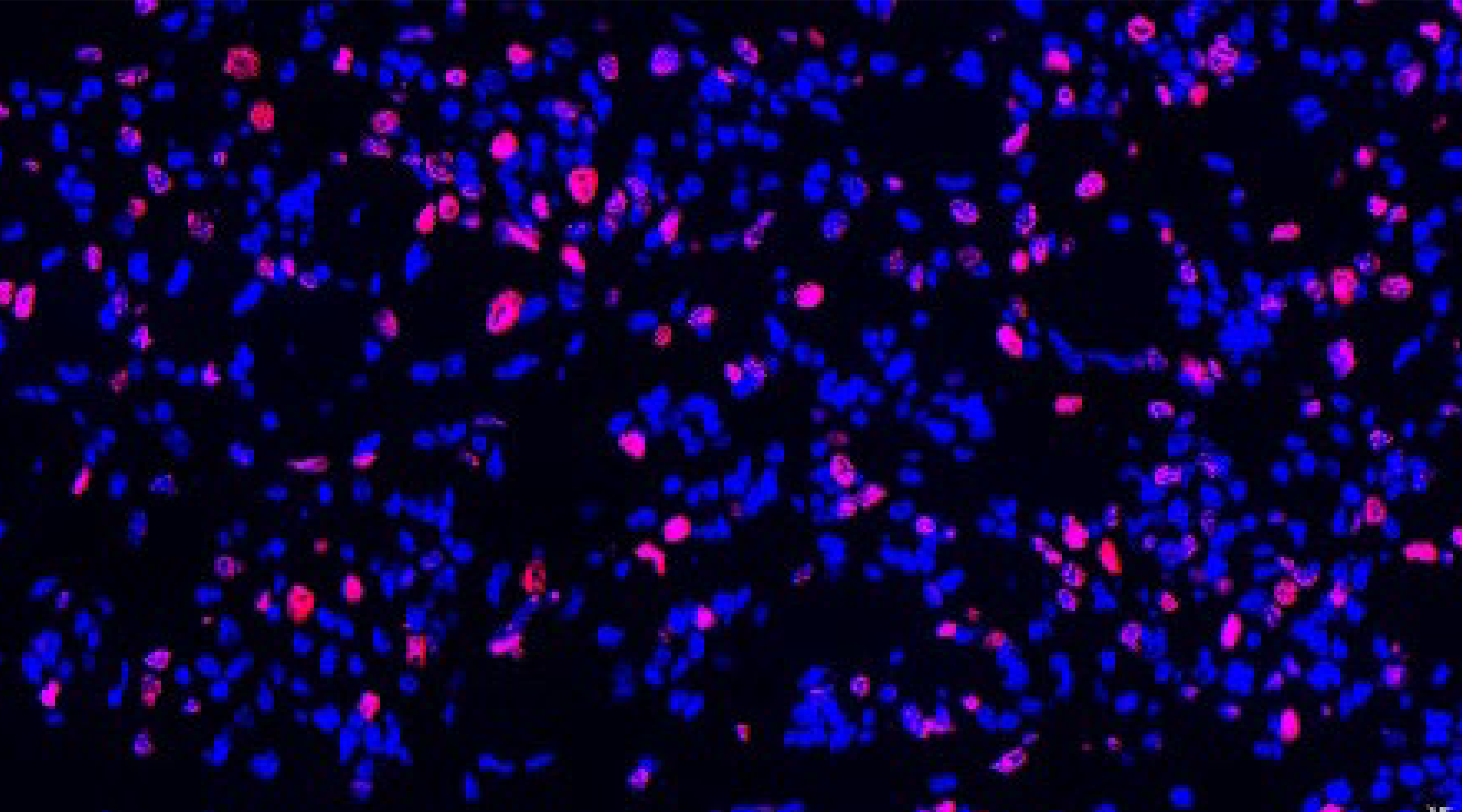

Supplement: Supplementary file 9 — Source data Fig. 6 [file 44318_2024_285_MOESM9_ESM.zip › Fig 6/Fig 6J/6J-shControl-MYOG.tif]

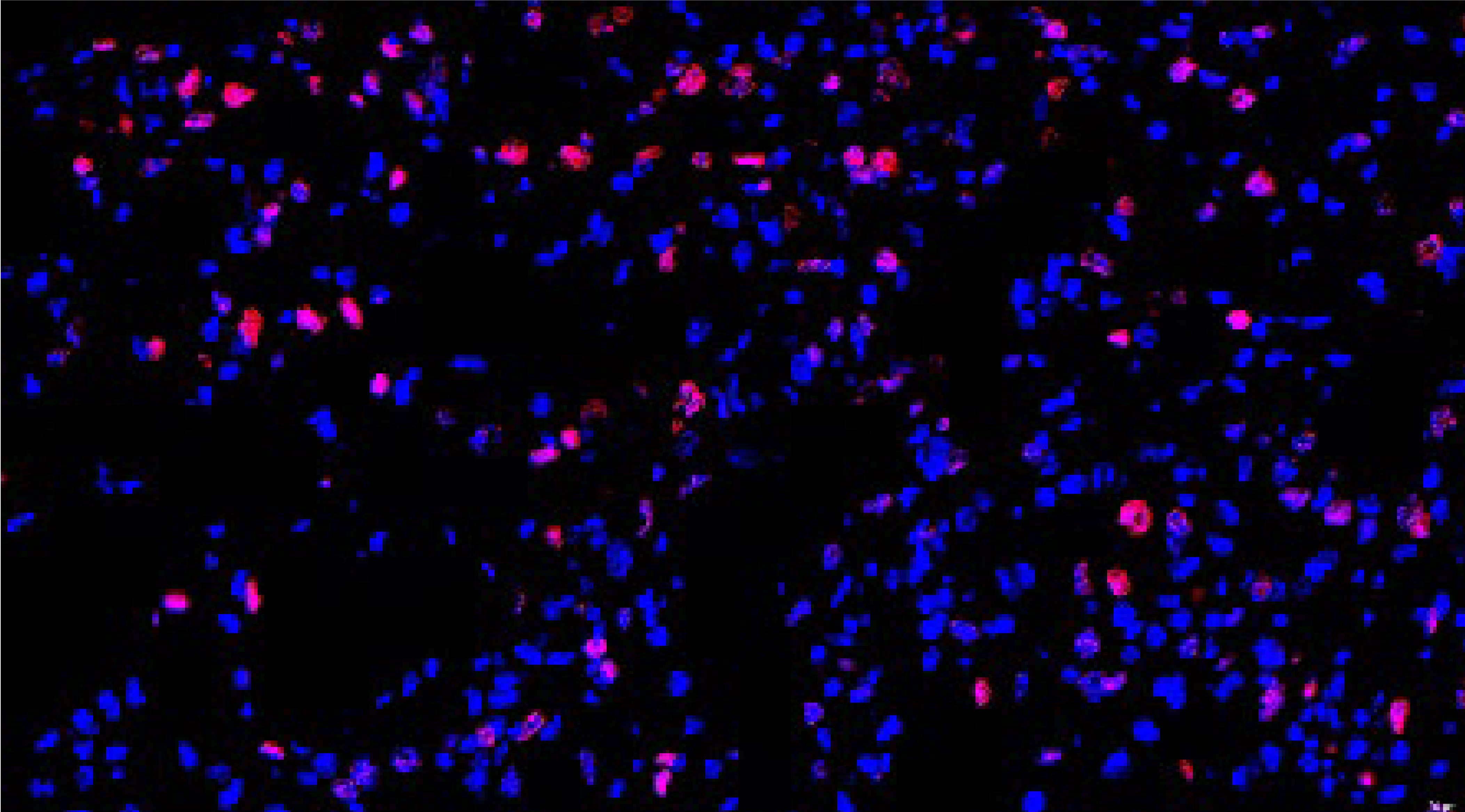

Supplement: Supplementary file 9 — Source data Fig. 6 [file 44318_2024_285_MOESM9_ESM.zip › Fig 6/Fig 6J/6J-shFNDC1-MYOG.tif]

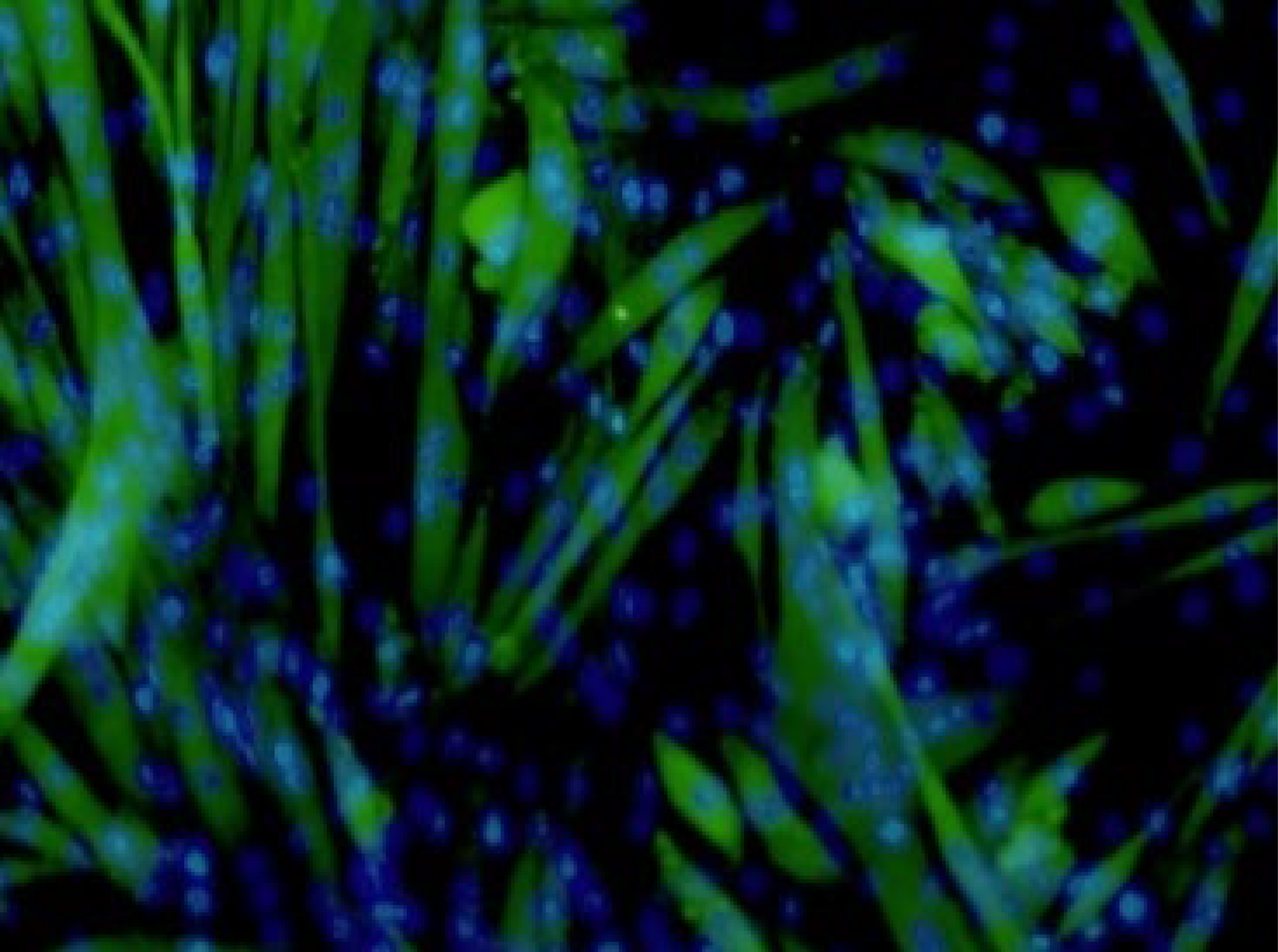

Supplement: Supplementary file 9 — Source data Fig. 6 [file 44318_2024_285_MOESM9_ESM.zip › Fig 6/Fig 6K/6K-Control-MYHC.tif]

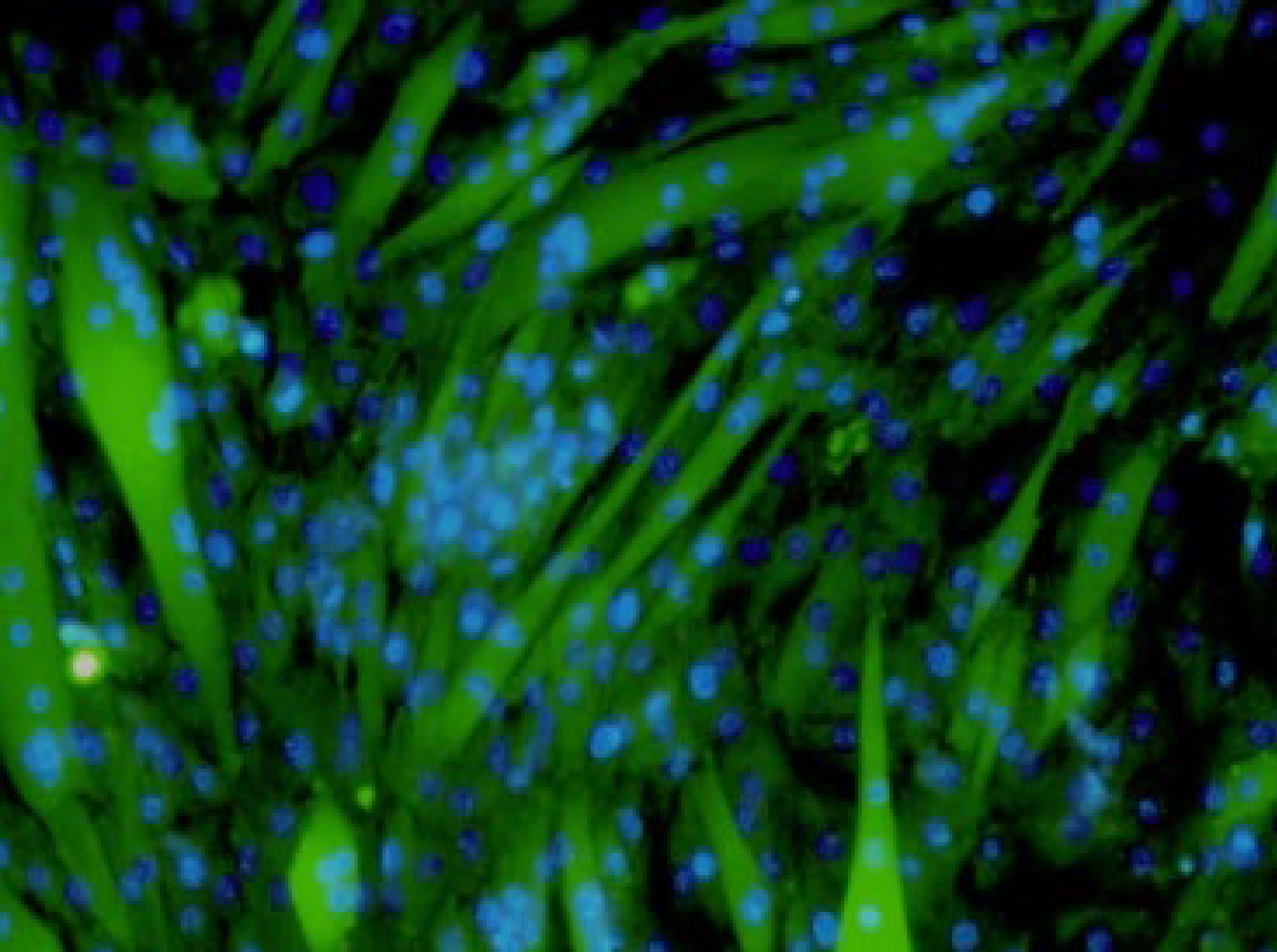

Supplement: Supplementary file 9 — Source data Fig. 6 [file 44318_2024_285_MOESM9_ESM.zip › Fig 6/Fig 6K/6K-mFNDC1-MYHC.tif]

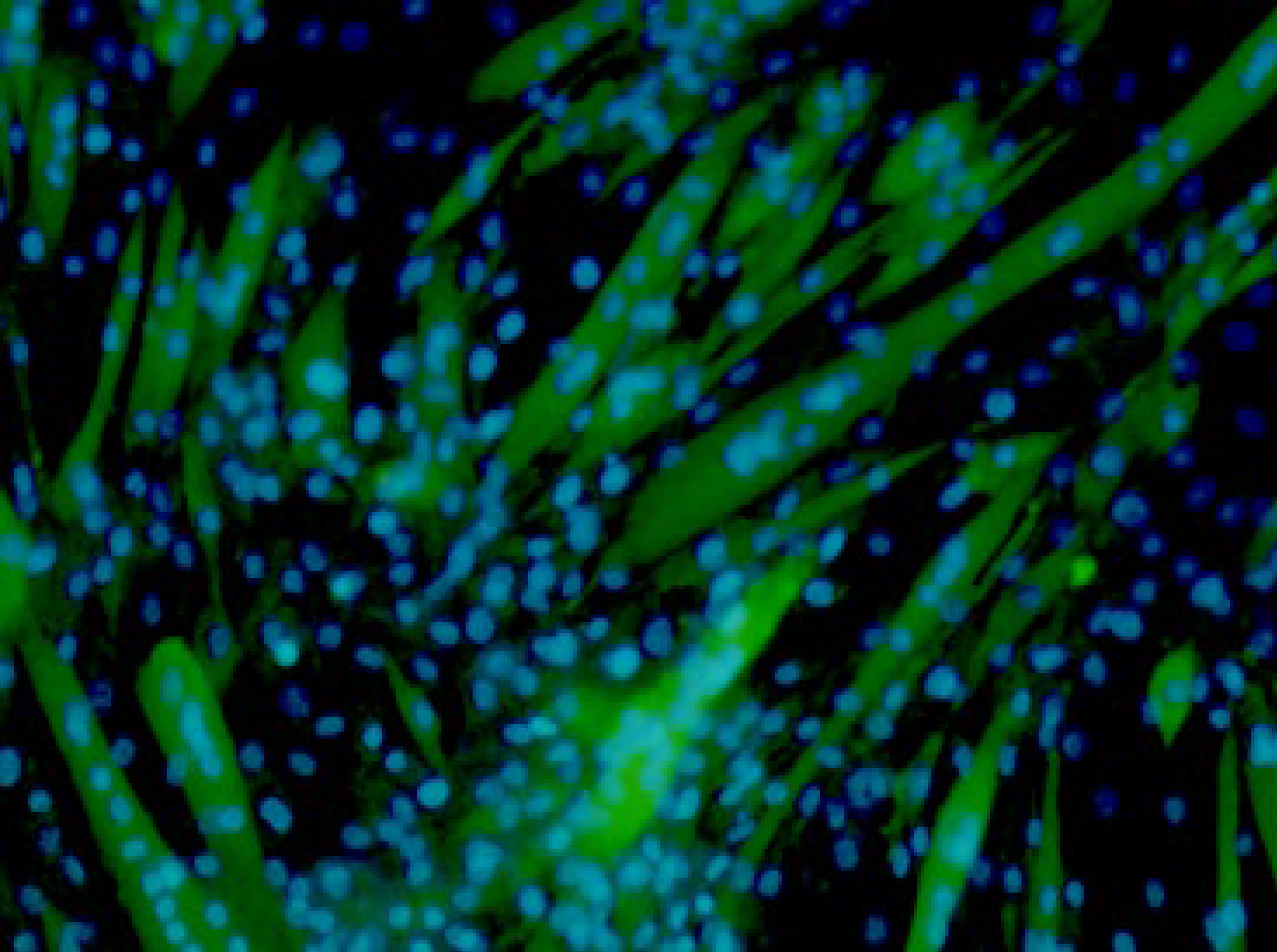

Supplement: Supplementary file 9 — Source data Fig. 6 [file 44318_2024_285_MOESM9_ESM.zip › Fig 6/Fig 6K/6K-shControl-MYHC.tif]

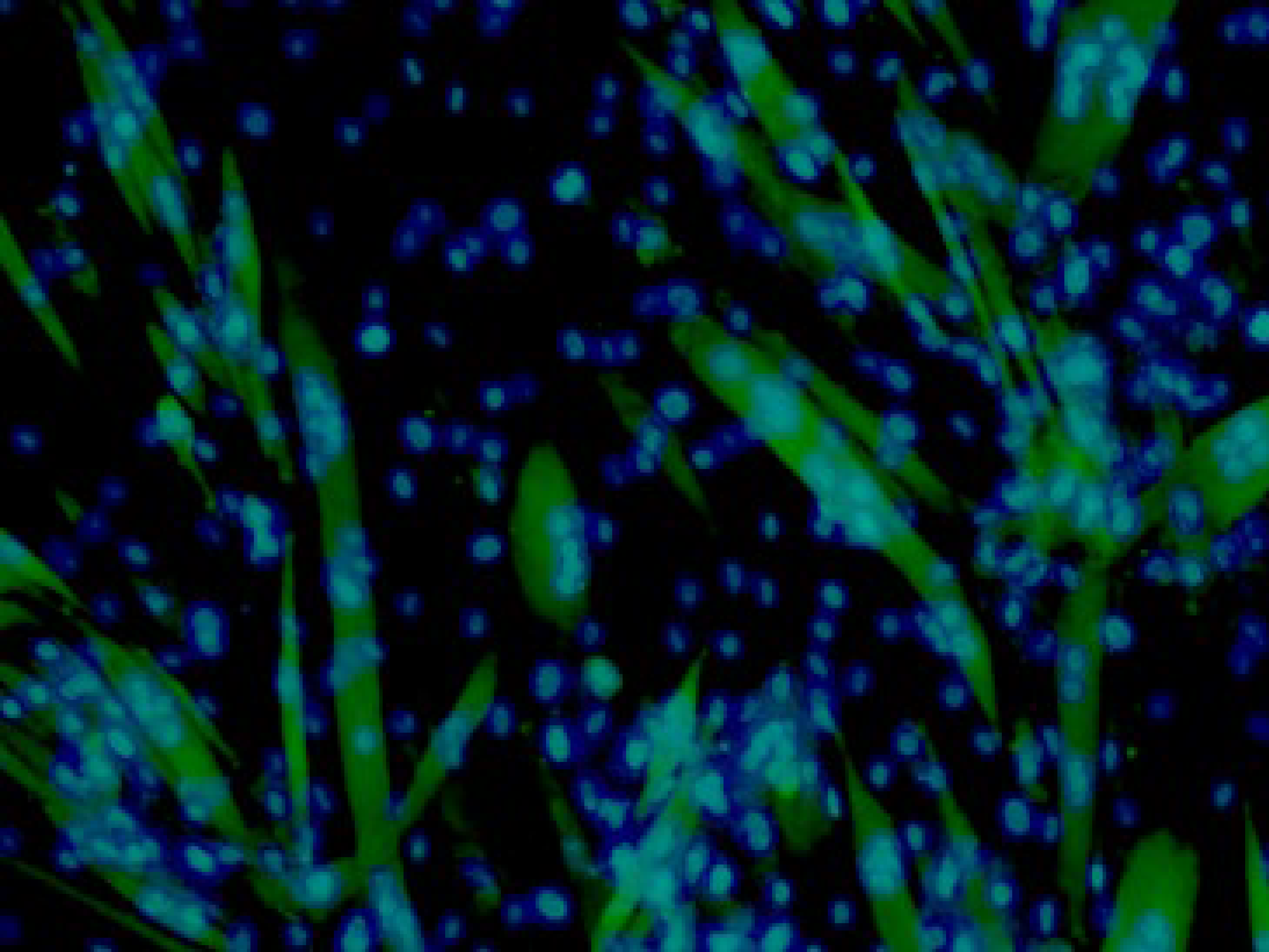

Supplement: Supplementary file 9 — Source data Fig. 6 [file 44318_2024_285_MOESM9_ESM.zip › Fig 6/Fig 6K/6K-shFNDC1-MYHC.tif]

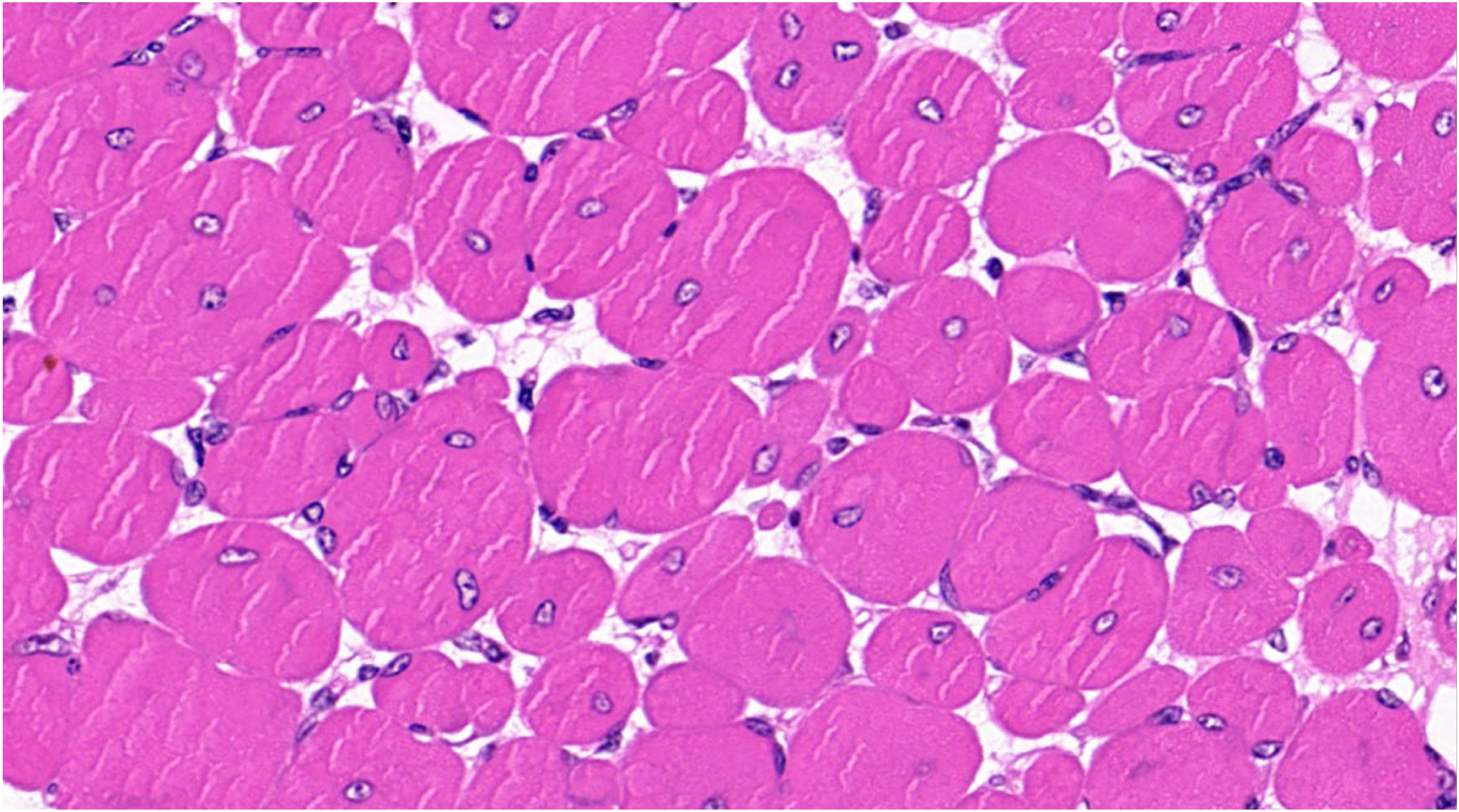

Supplement: Supplementary file 10 — Source data Fig. 7 [file 44318_2024_285_MOESM10_ESM.zip › Fig 7/Fig 7A/7A-Control+AAV-itgb1-Day14.tif]

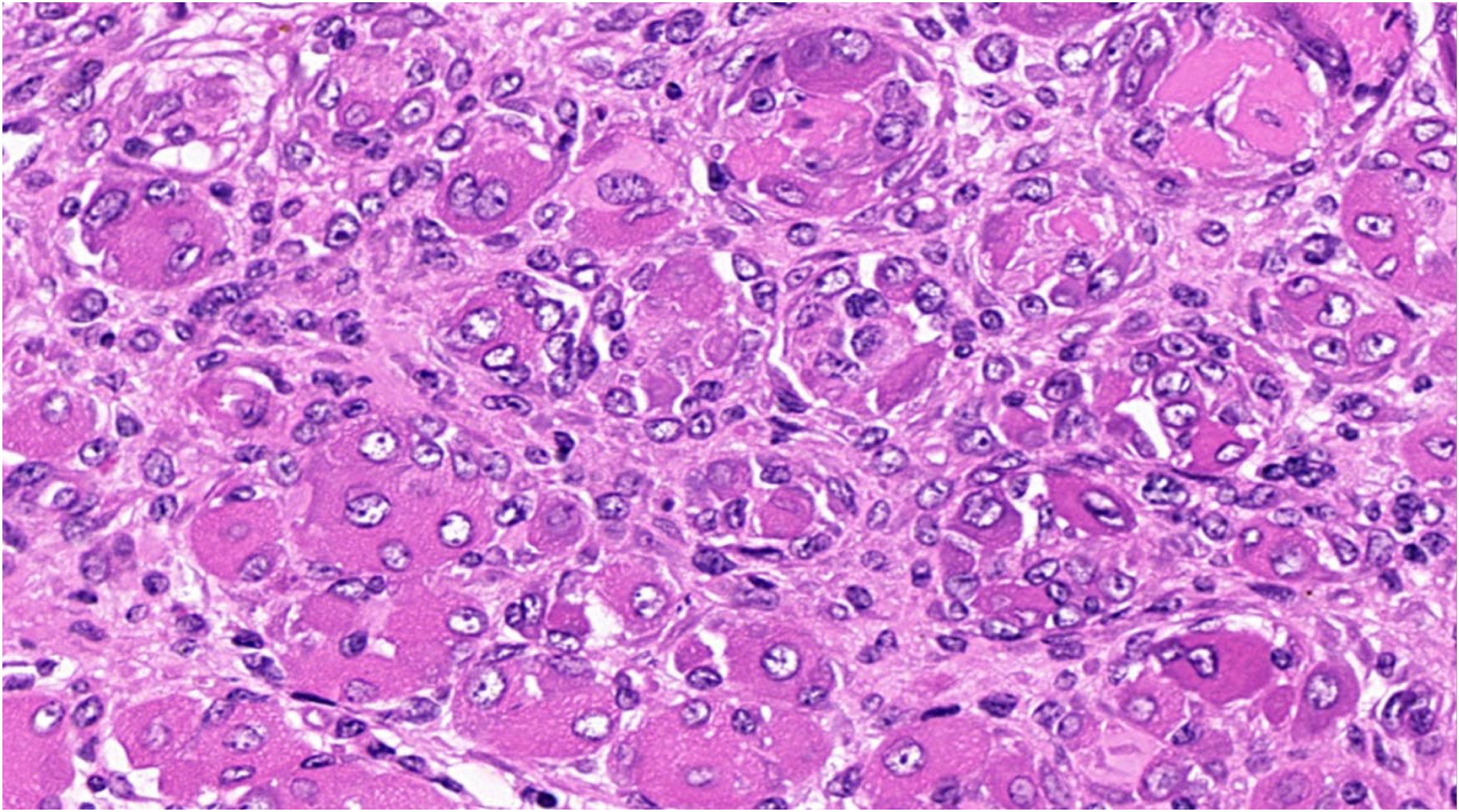

Supplement: Supplementary file 10 — Source data Fig. 7 [file 44318_2024_285_MOESM10_ESM.zip › Fig 7/Fig 7A/7A-Control+AAV-itgb1.tif]

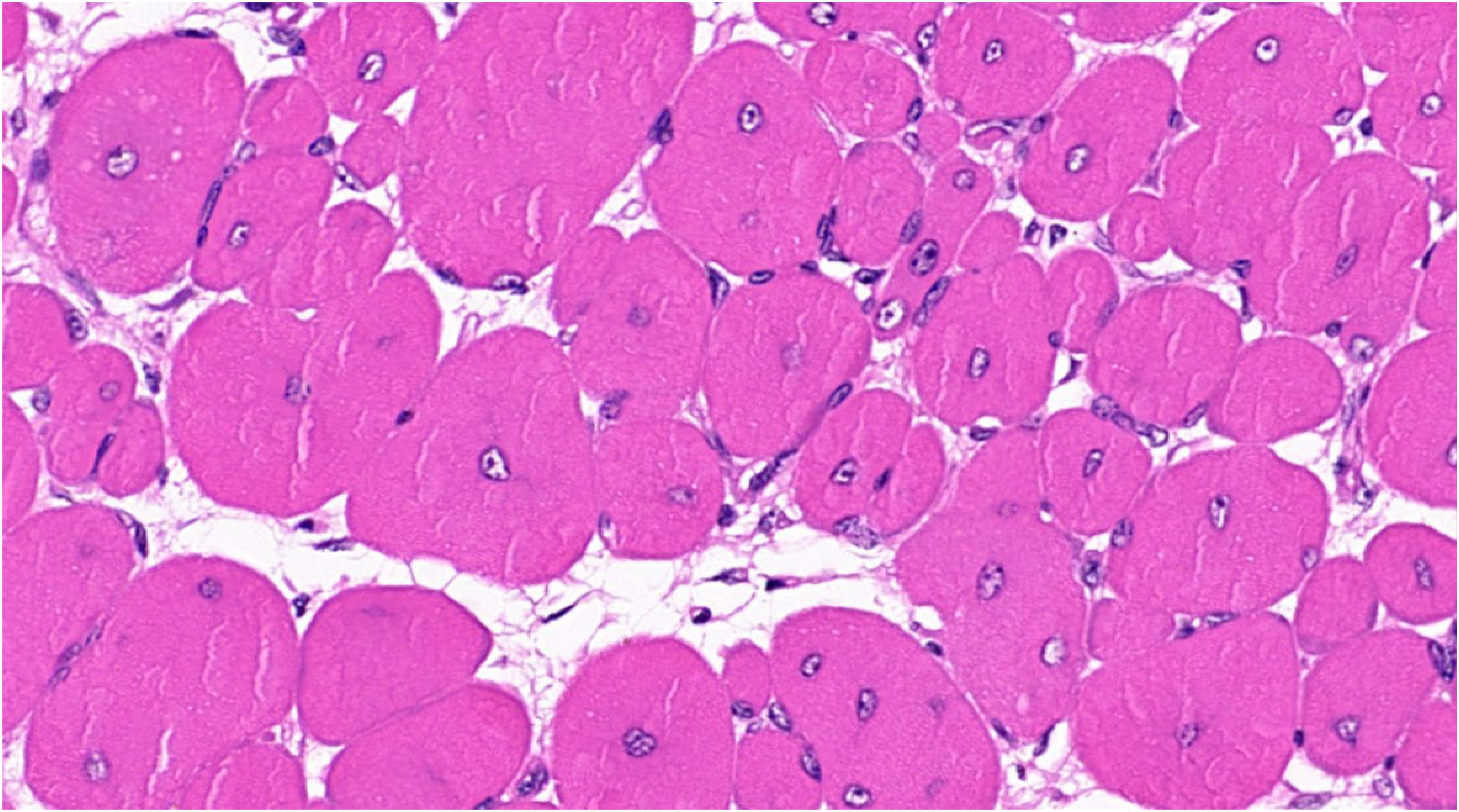

Supplement: Supplementary file 10 — Source data Fig. 7 [file 44318_2024_285_MOESM10_ESM.zip › Fig 7/Fig 7A/7A-Control+AAV-scra-Day14.tif]

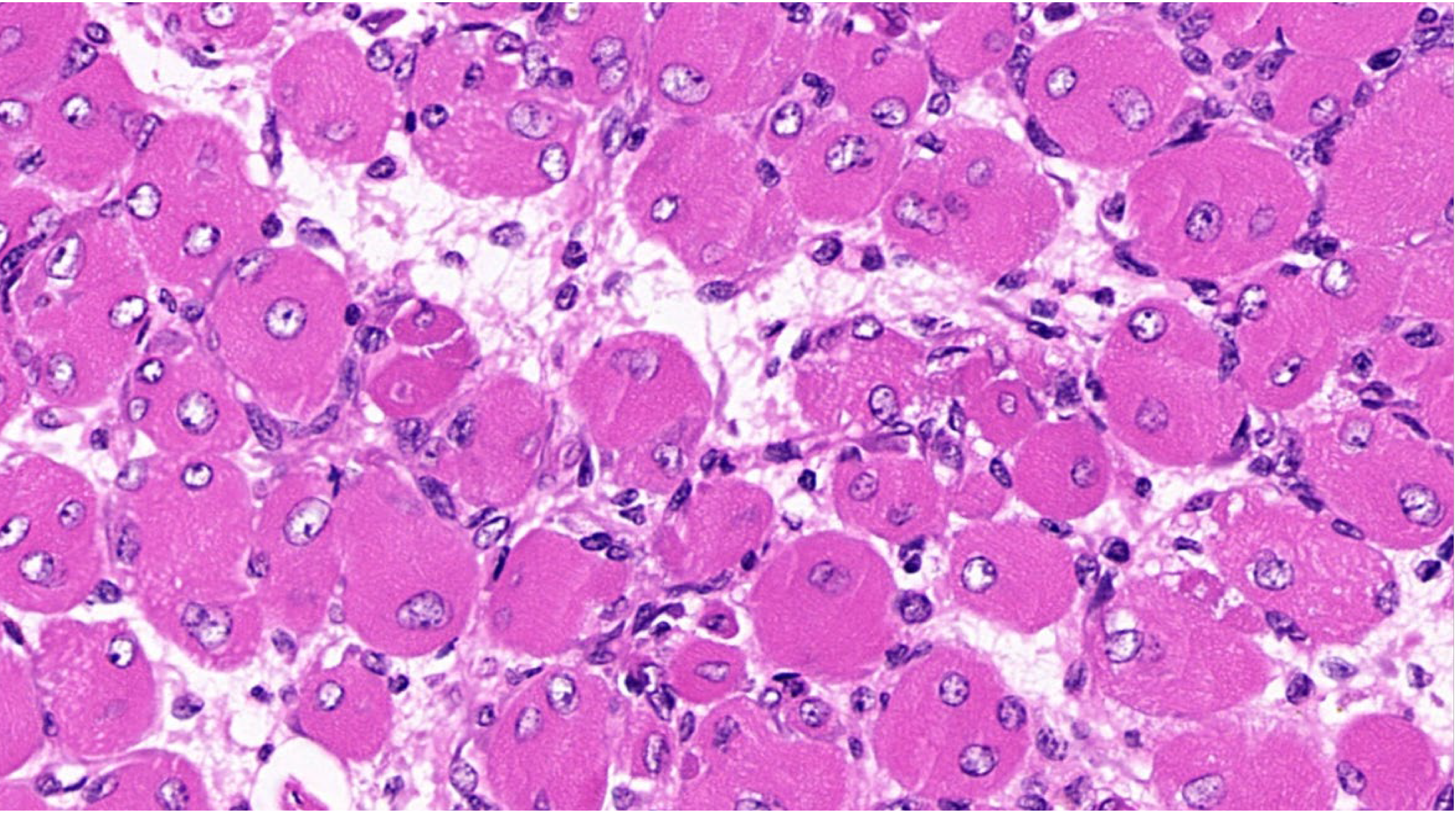

Supplement: Supplementary file 10 — Source data Fig. 7 [file 44318_2024_285_MOESM10_ESM.zip › Fig 7/Fig 7A/7A-Control+AAV-scra.tif]

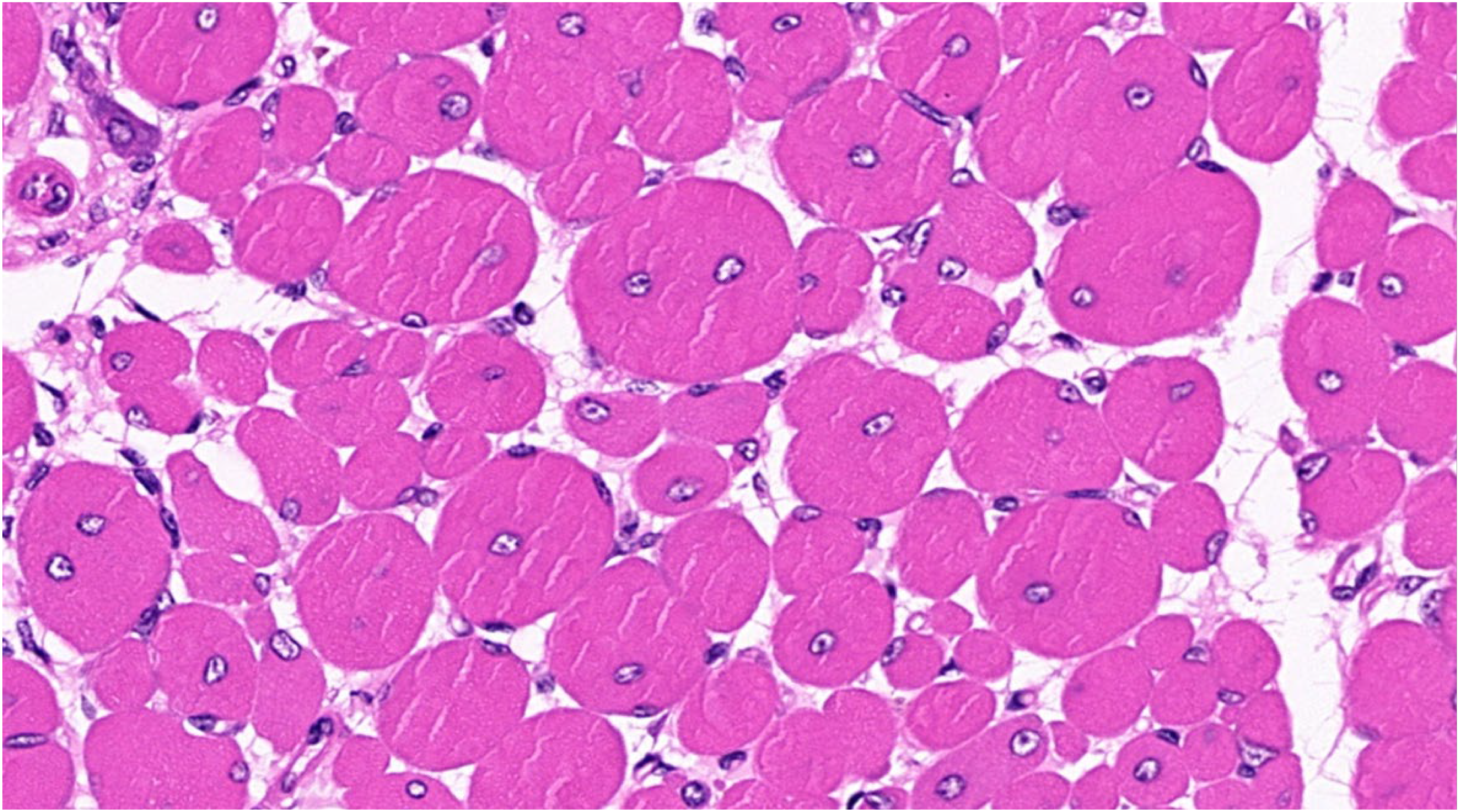

Supplement: Supplementary file 10 — Source data Fig. 7 [file 44318_2024_285_MOESM10_ESM.zip › Fig 7/Fig 7A/7A-mFNDC1+AAV-itgb1-Day14.tif]

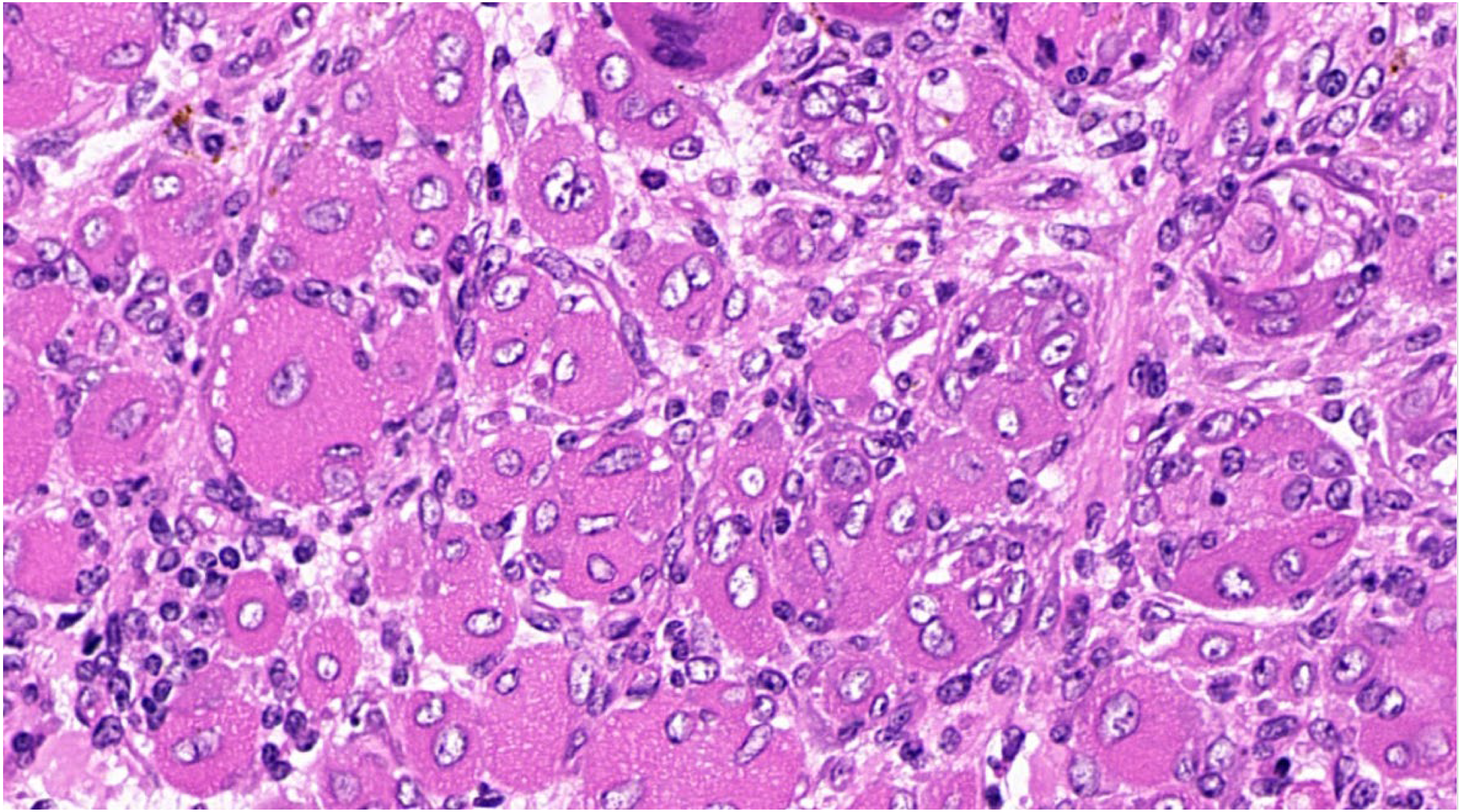

Supplement: Supplementary file 10 — Source data Fig. 7 [file 44318_2024_285_MOESM10_ESM.zip › Fig 7/Fig 7A/7A-mFNDC1+AAV-itgb1.tif]

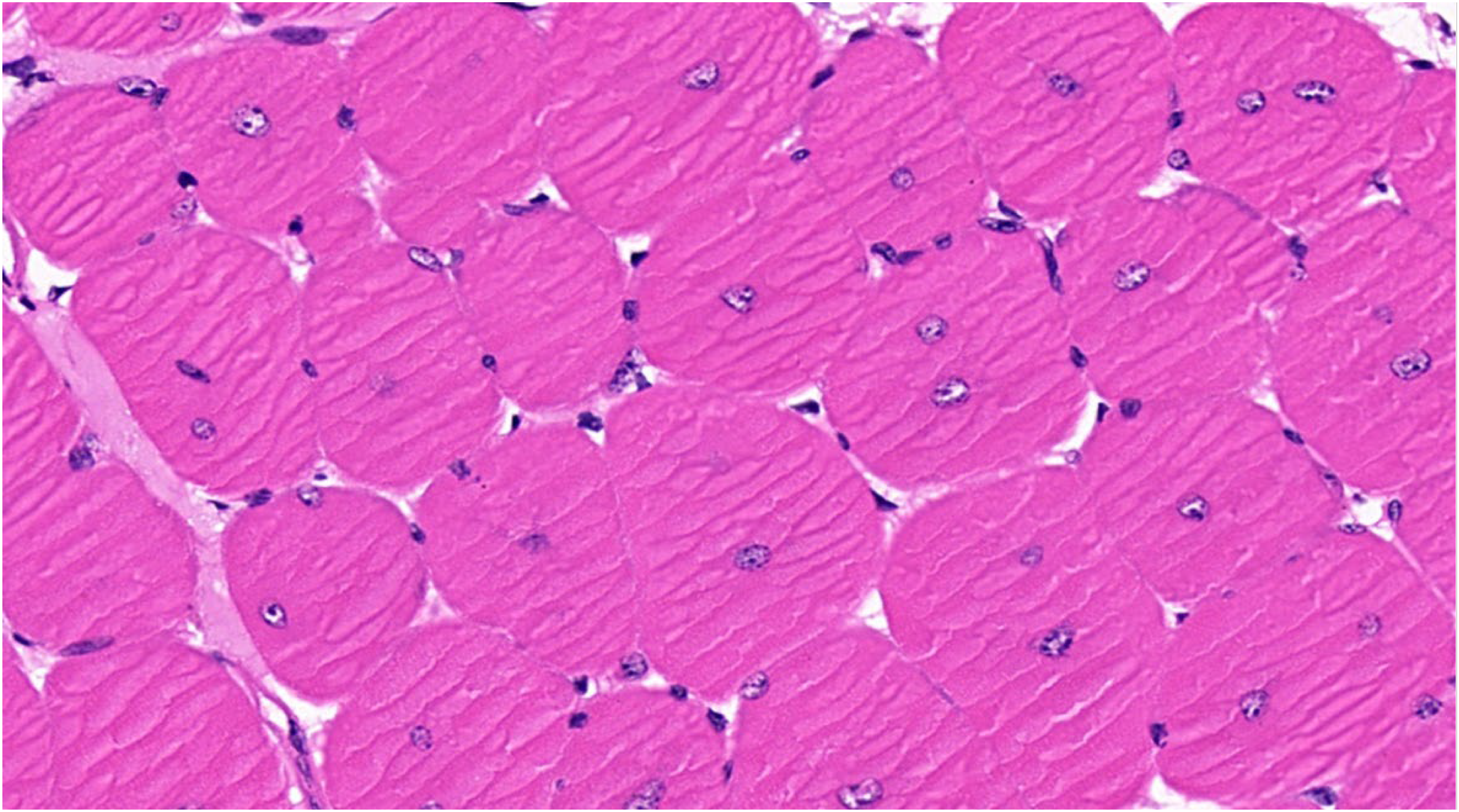

Supplement: Supplementary file 10 — Source data Fig. 7 [file 44318_2024_285_MOESM10_ESM.zip › Fig 7/Fig 7A/7A-mFNDC1+AAV-scra-day14.tif]

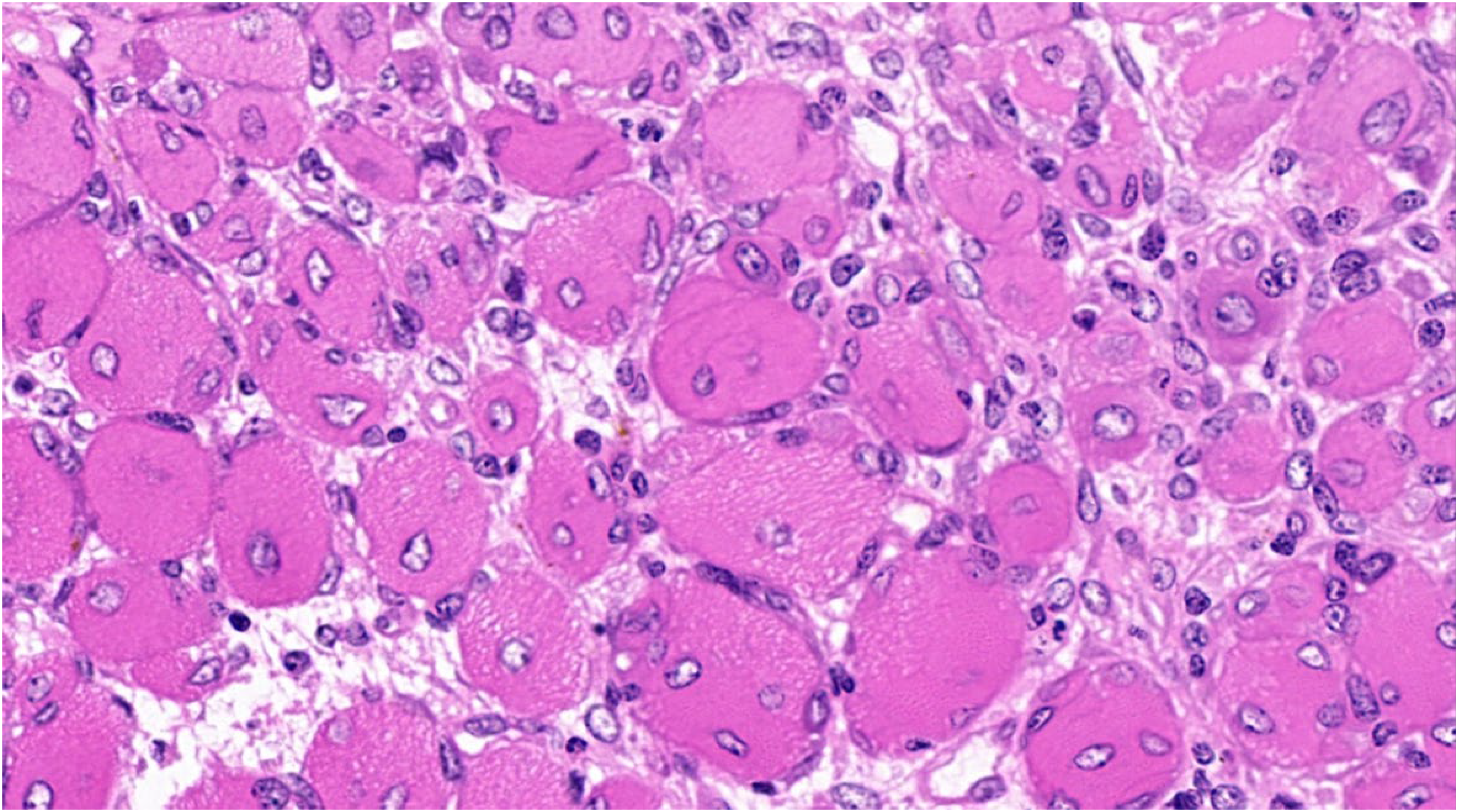

Supplement: Supplementary file 10 — Source data Fig. 7 [file 44318_2024_285_MOESM10_ESM.zip › Fig 7/Fig 7A/7A-mFNDC1+AAV-scra.tif]

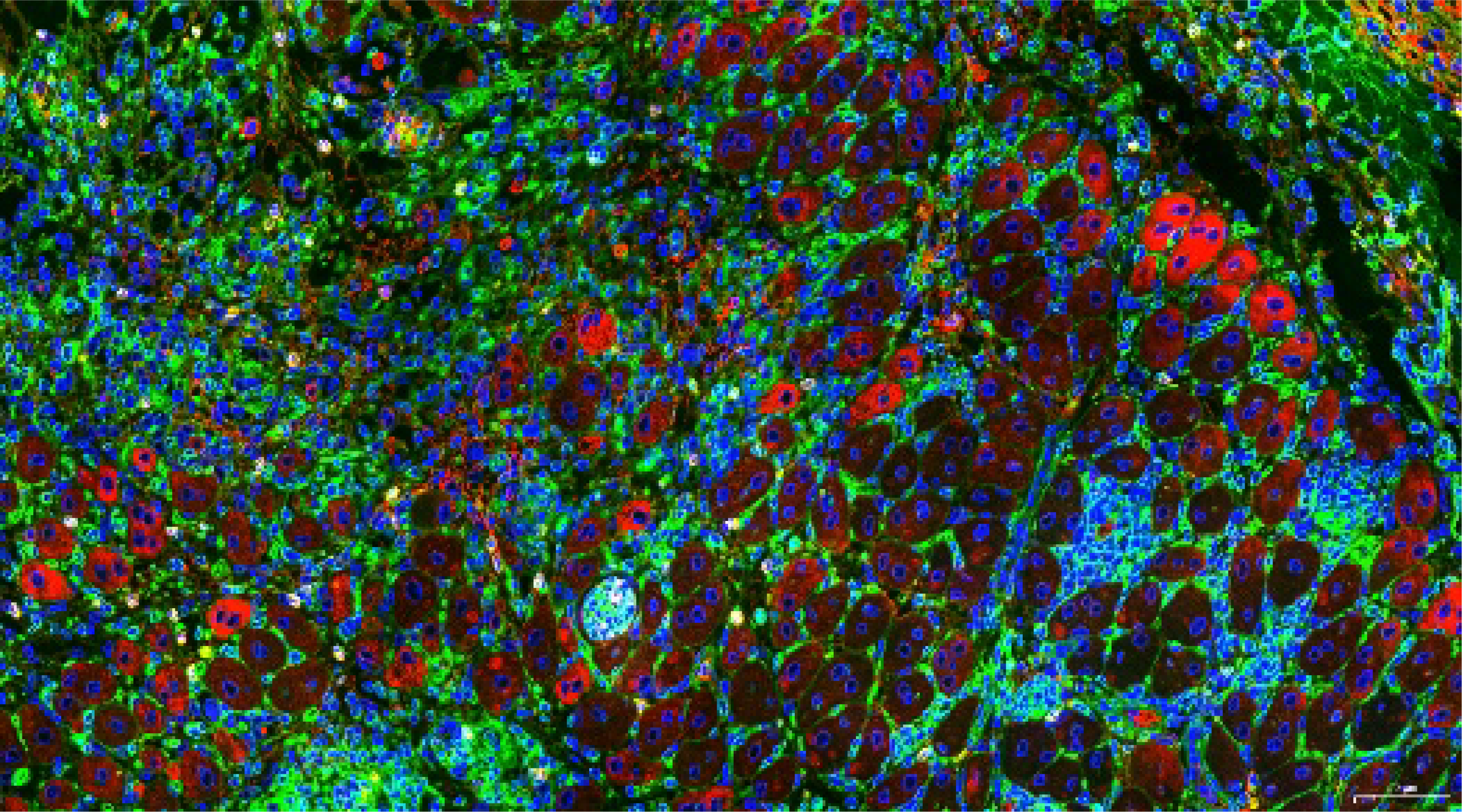

Supplement: Supplementary file 10 — Source data Fig. 7 [file 44318_2024_285_MOESM10_ESM.zip › Fig 7/Fig 7B/7B-Control+AAV-itgb1.tif]

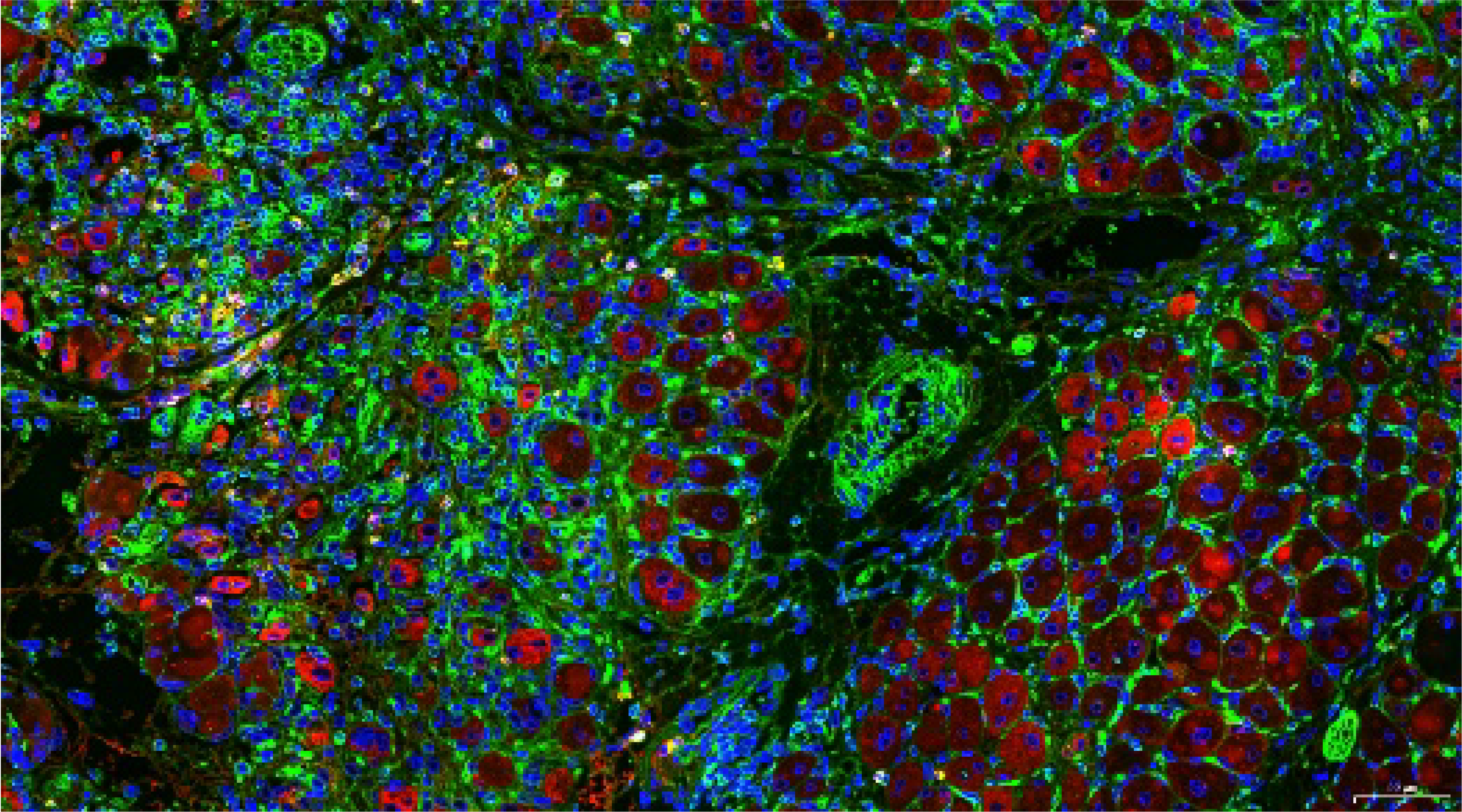

Supplement: Supplementary file 10 — Source data Fig. 7 [file 44318_2024_285_MOESM10_ESM.zip › Fig 7/Fig 7B/7B-Control+AAV-scra.tif]

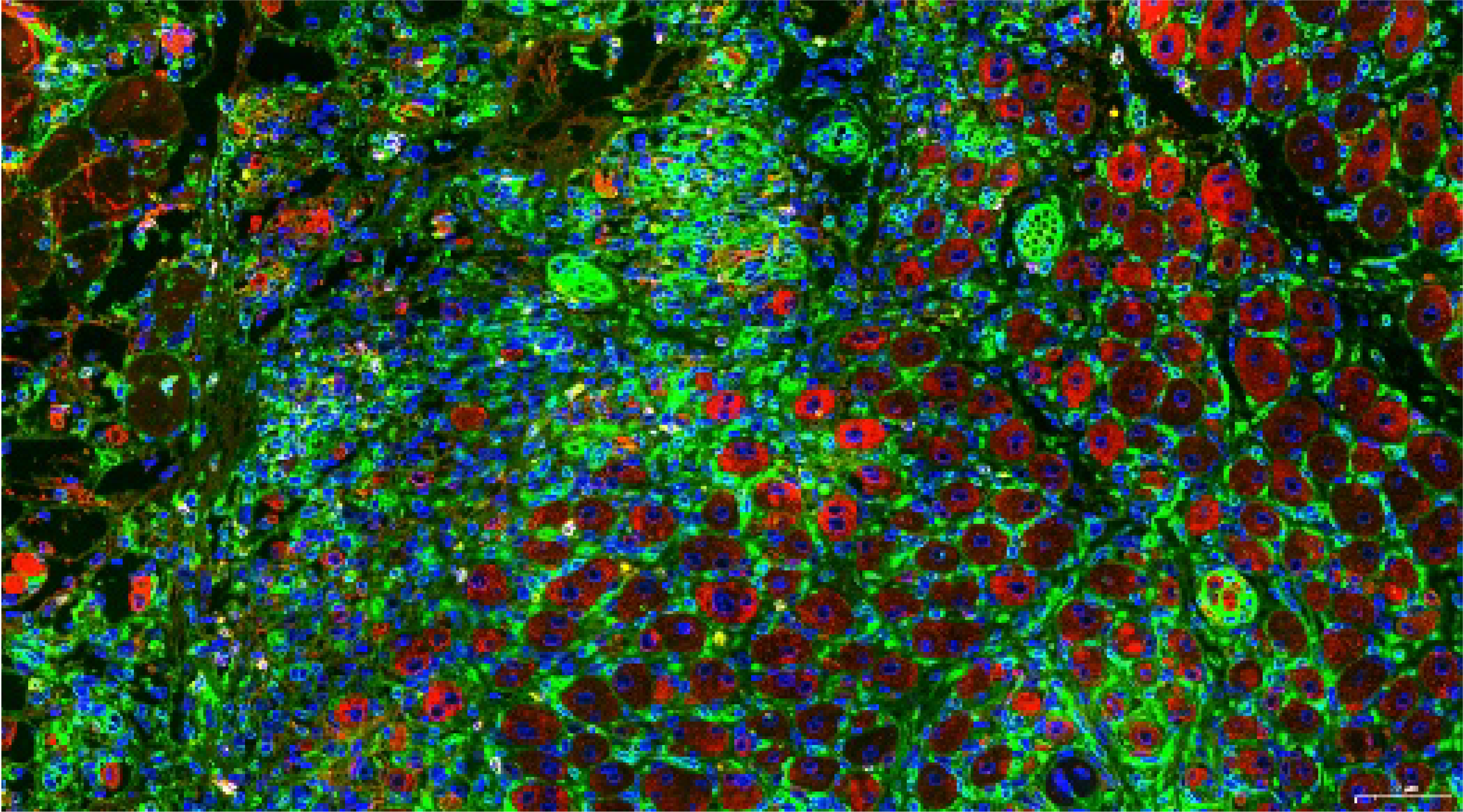

Supplement: Supplementary file 10 — Source data Fig. 7 [file 44318_2024_285_MOESM10_ESM.zip › Fig 7/Fig 7B/7B-mFNDC1+AAV-itgb1.tif]

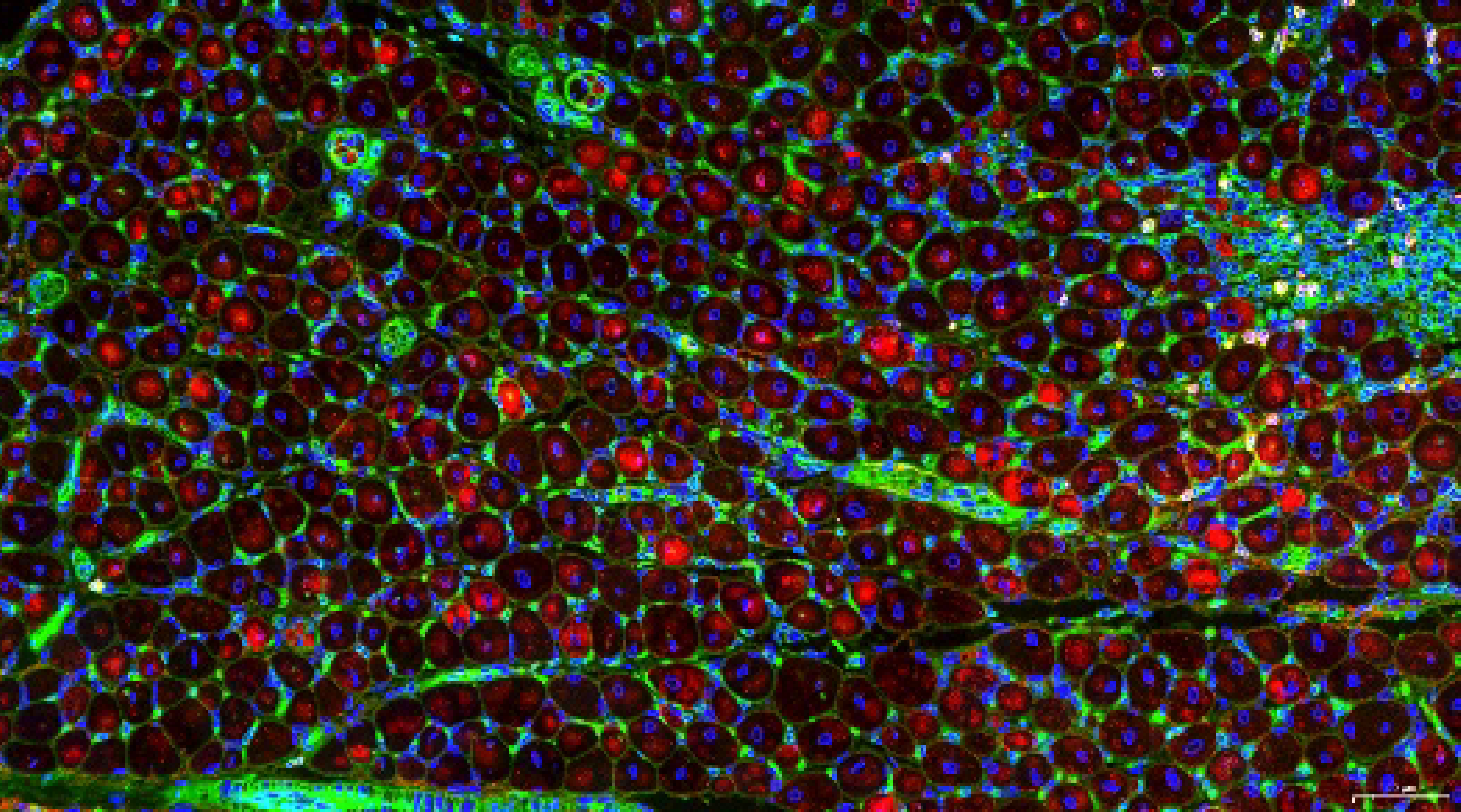

Supplement: Supplementary file 10 — Source data Fig. 7 [file 44318_2024_285_MOESM10_ESM.zip › Fig 7/Fig 7B/7B-mFNDC1+AAV-scra.tif]

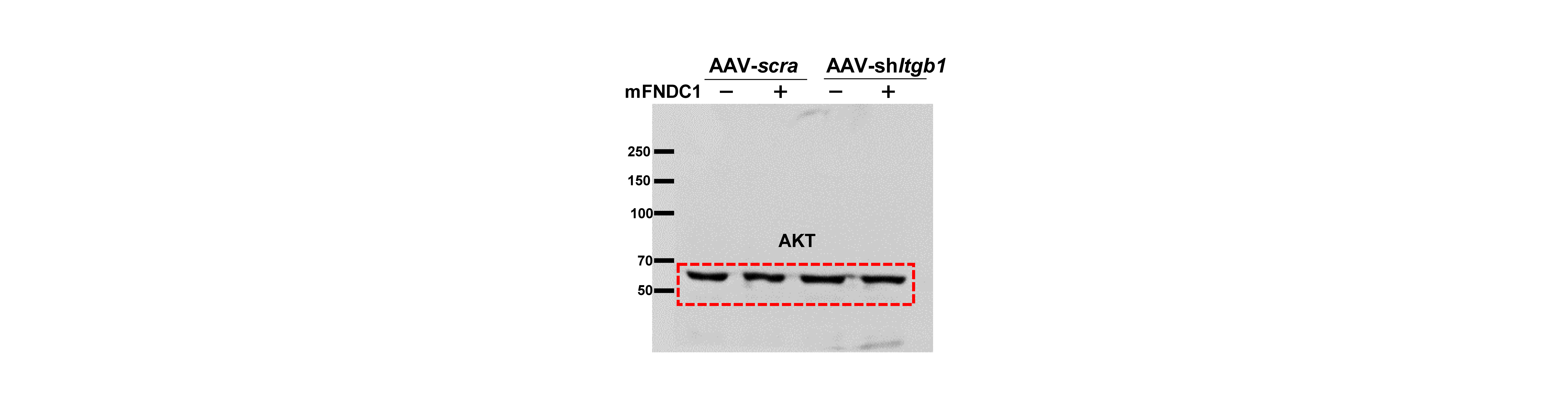

Supplement: Supplementary file 10 — Source data Fig. 7 [file 44318_2024_285_MOESM10_ESM.zip › Fig 7/Fig 7E/7-E-AKT.tif]

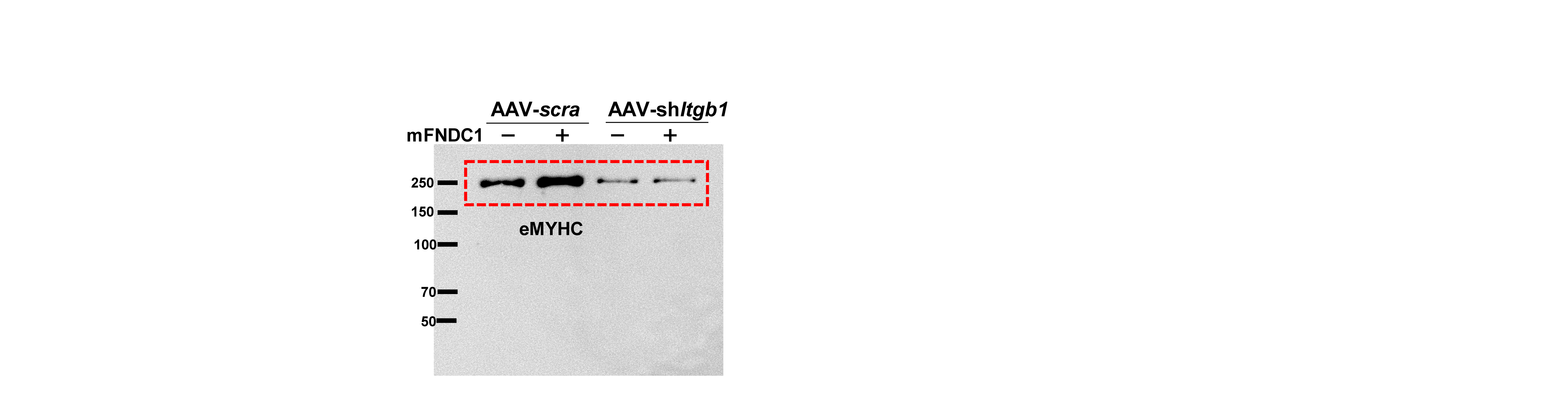

Supplement: Supplementary file 10 — Source data Fig. 7 [file 44318_2024_285_MOESM10_ESM.zip › Fig 7/Fig 7E/7-E-eMYHC.tif]

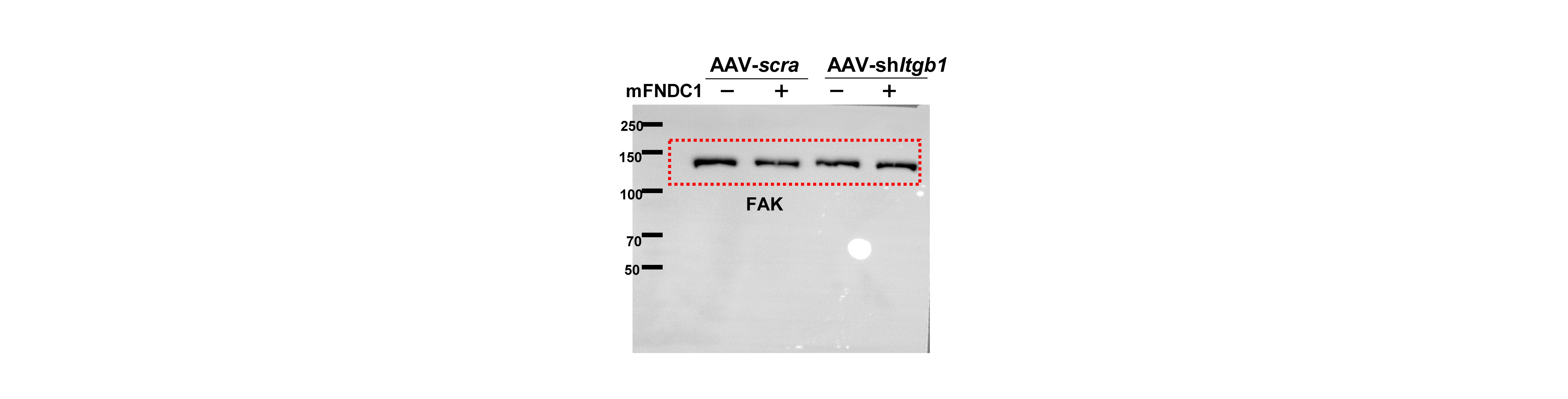

Supplement: Supplementary file 10 — Source data Fig. 7 [file 44318_2024_285_MOESM10_ESM.zip › Fig 7/Fig 7E/7-E-FAK.tif]

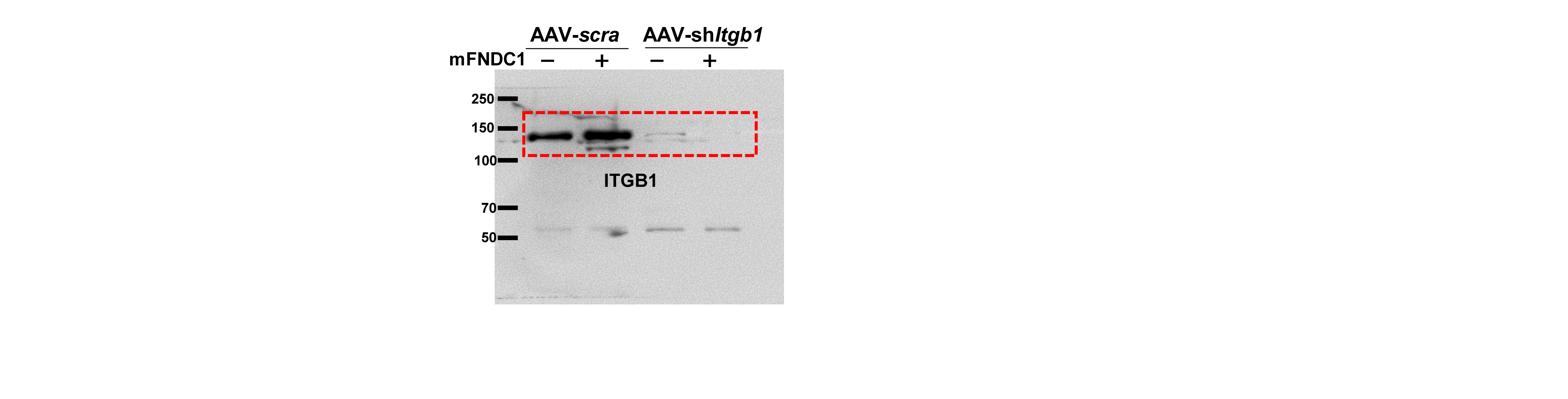

Supplement: Supplementary file 10 — Source data Fig. 7 [file 44318_2024_285_MOESM10_ESM.zip › Fig 7/Fig 7E/7-E-ITGB1.tif]

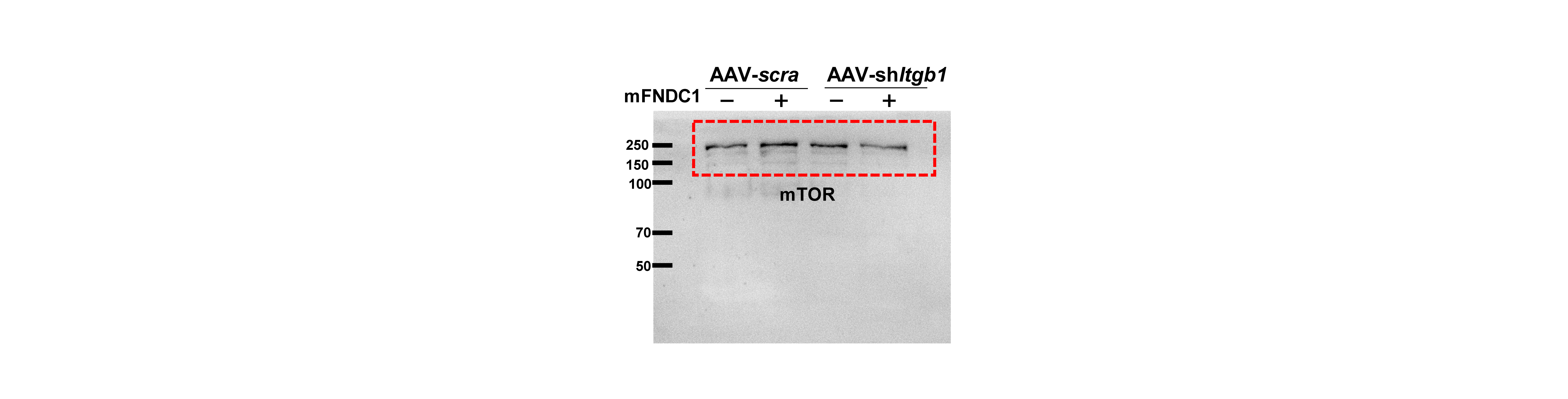

Supplement: Supplementary file 10 — Source data Fig. 7 [file 44318_2024_285_MOESM10_ESM.zip › Fig 7/Fig 7E/7-E-mTOR.tif]

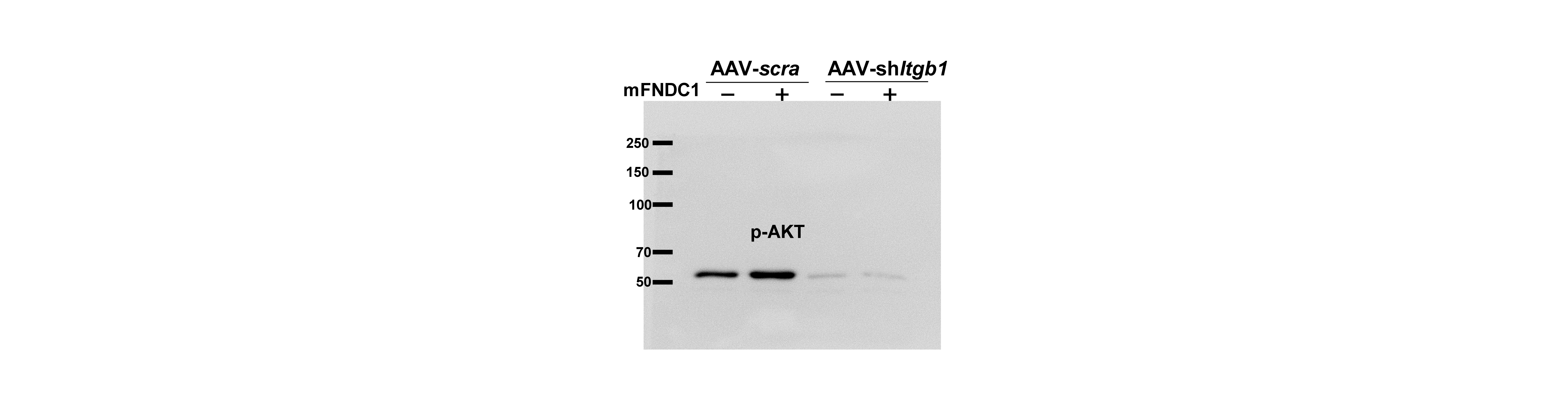

Supplement: Supplementary file 10 — Source data Fig. 7 [file 44318_2024_285_MOESM10_ESM.zip › Fig 7/Fig 7E/7-E-p-AKT.tif]

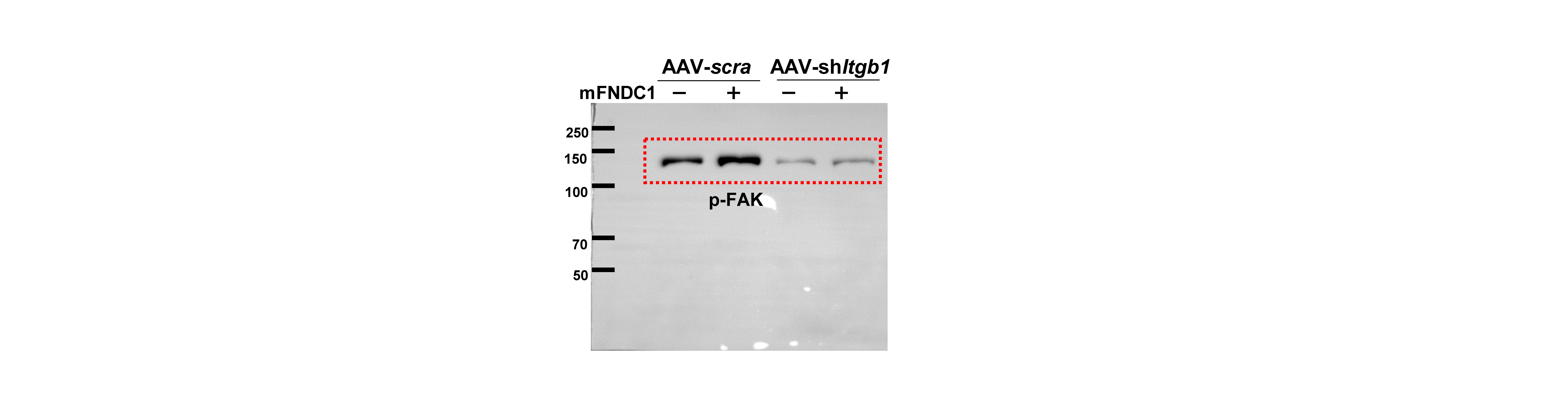

Supplement: Supplementary file 10 — Source data Fig. 7 [file 44318_2024_285_MOESM10_ESM.zip › Fig 7/Fig 7E/7-E-p-FAK.tif]

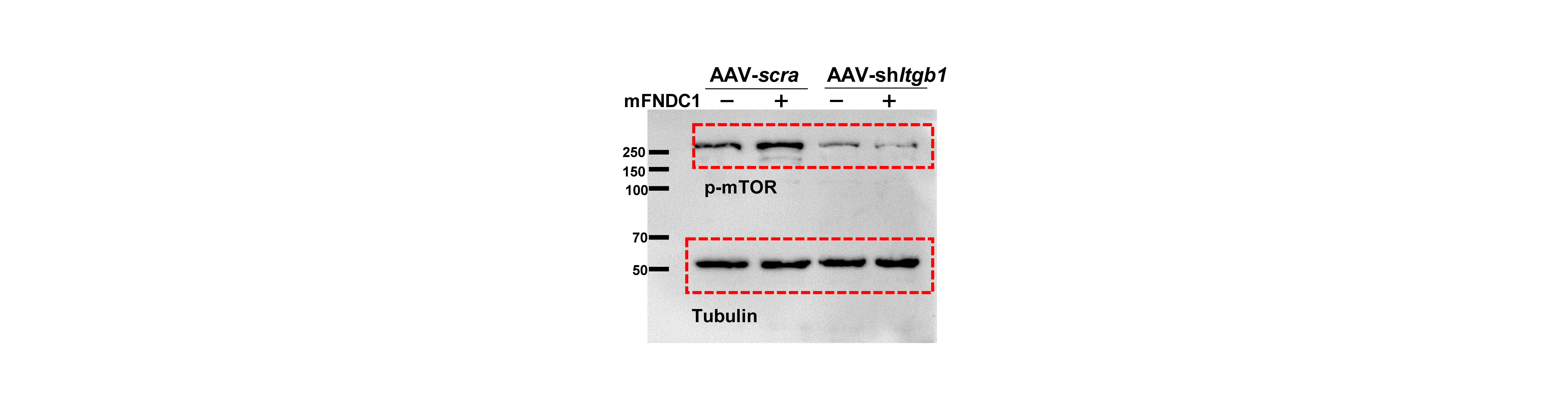

Supplement: Supplementary file 10 — Source data Fig. 7 [file 44318_2024_285_MOESM10_ESM.zip › Fig 7/Fig 7E/7-E-pmTOR-TUBULIN.tif]

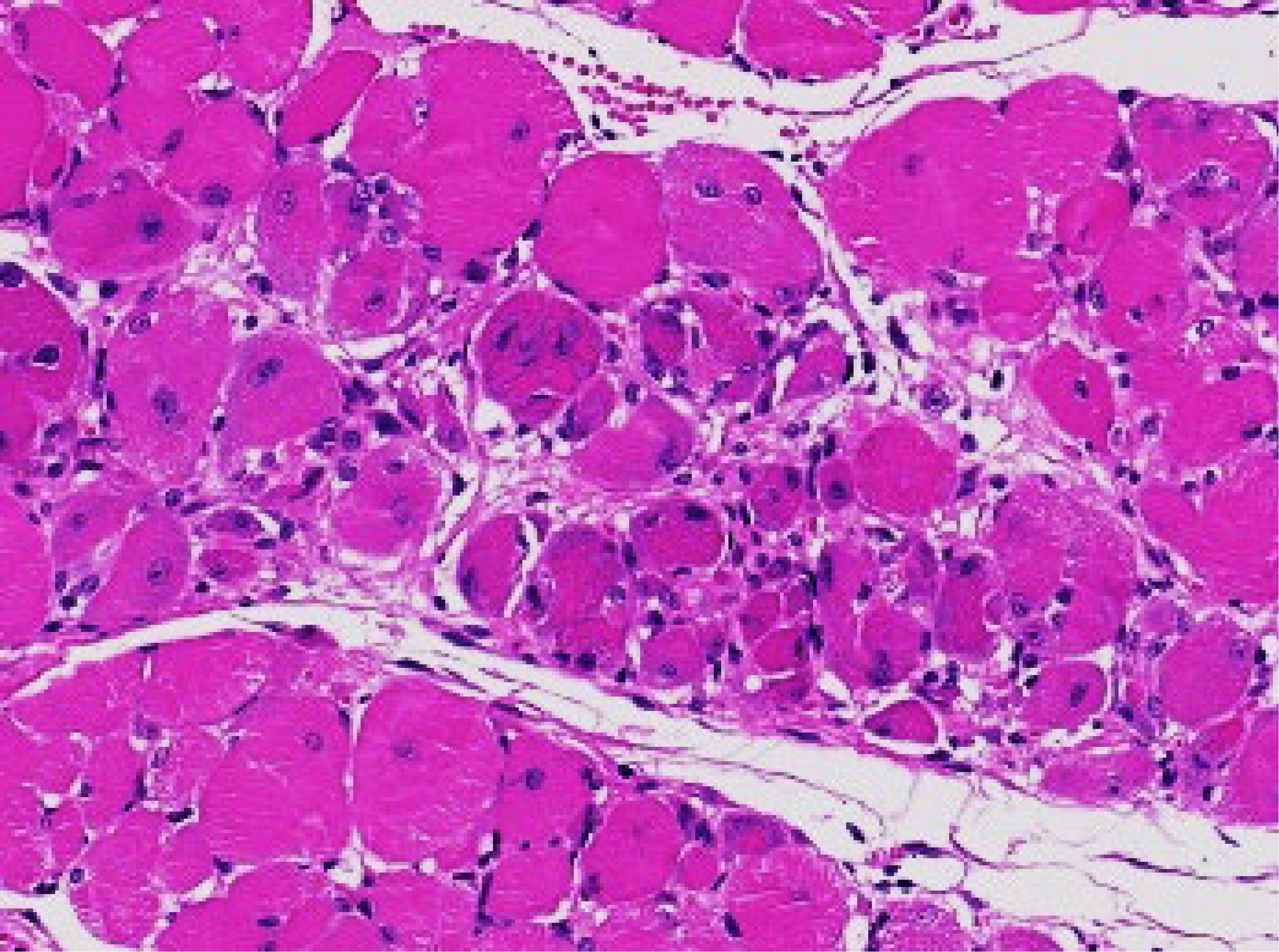

Supplement: Supplementary file 11 — Source data Fig. 8 [file 44318_2024_285_MOESM11_ESM.zip › Fig 8/Fig 8B/8B-Control-DIA.tif]

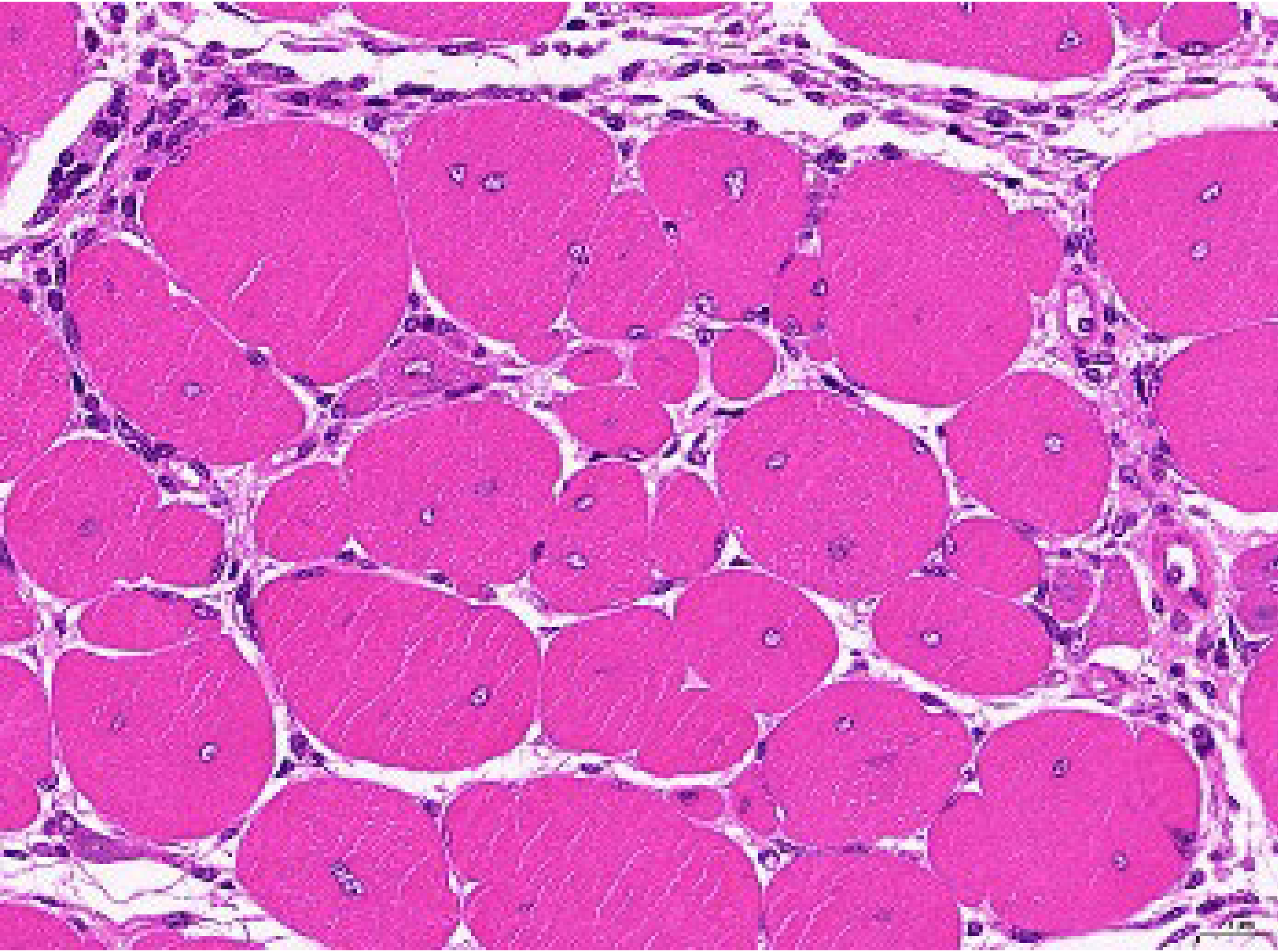

Supplement: Supplementary file 11 — Source data Fig. 8 [file 44318_2024_285_MOESM11_ESM.zip › Fig 8/Fig 8B/8B-Control-QUA.tif]

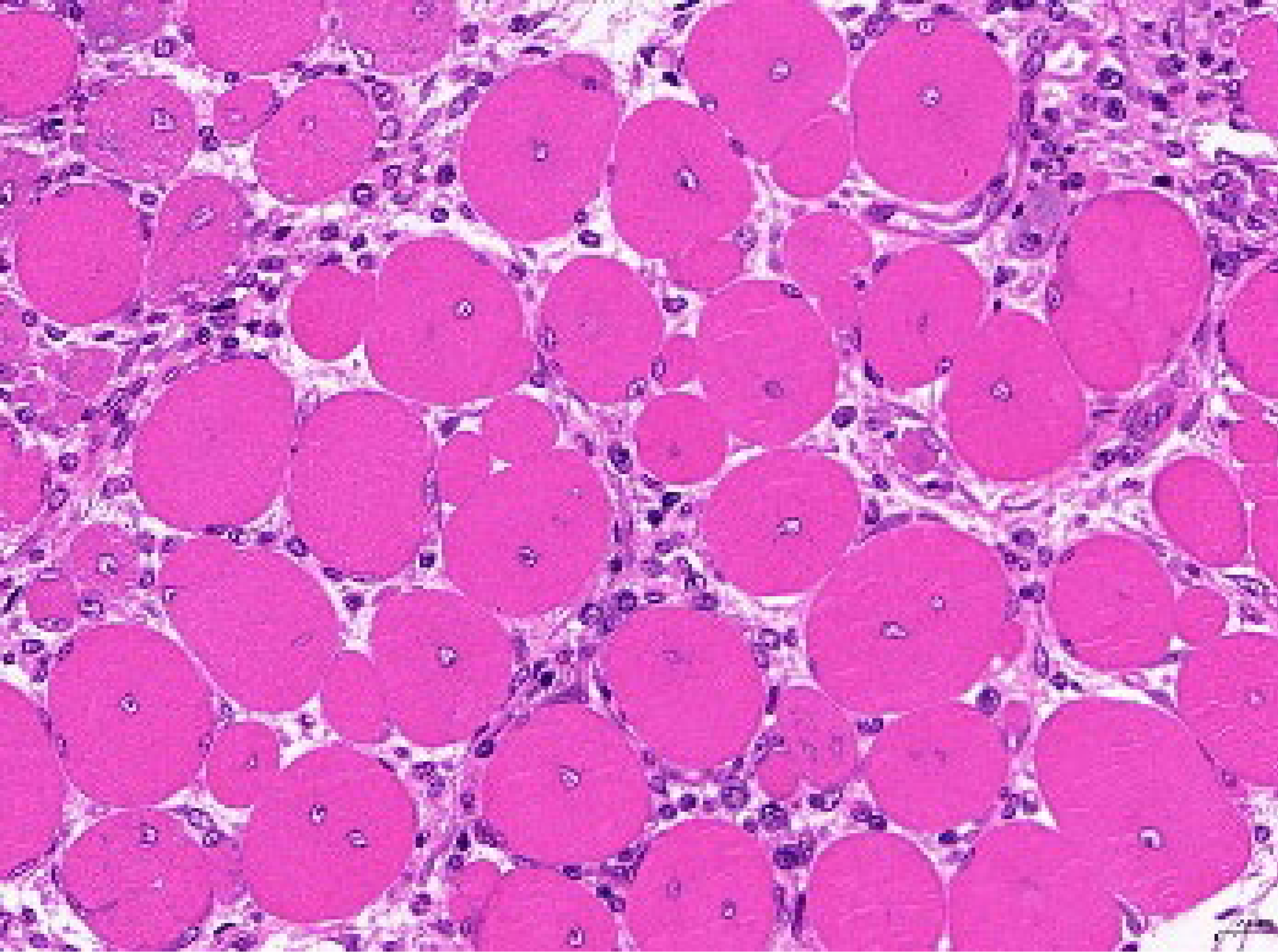

Supplement: Supplementary file 11 — Source data Fig. 8 [file 44318_2024_285_MOESM11_ESM.zip › Fig 8/Fig 8B/8B-Control-TA.tif]

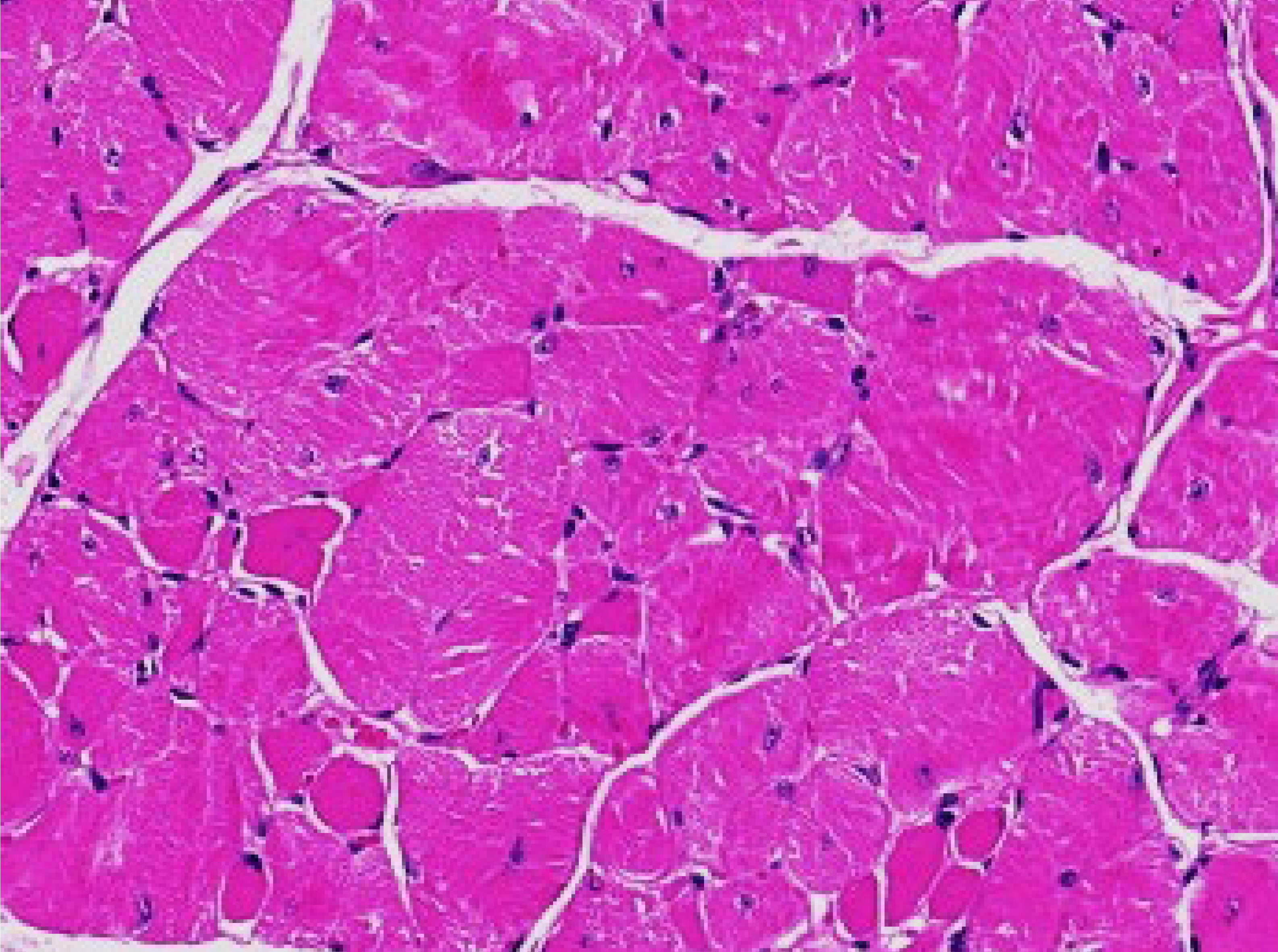

Supplement: Supplementary file 11 — Source data Fig. 8 [file 44318_2024_285_MOESM11_ESM.zip › Fig 8/Fig 8B/8B-mFNDC1-DIA.tif]

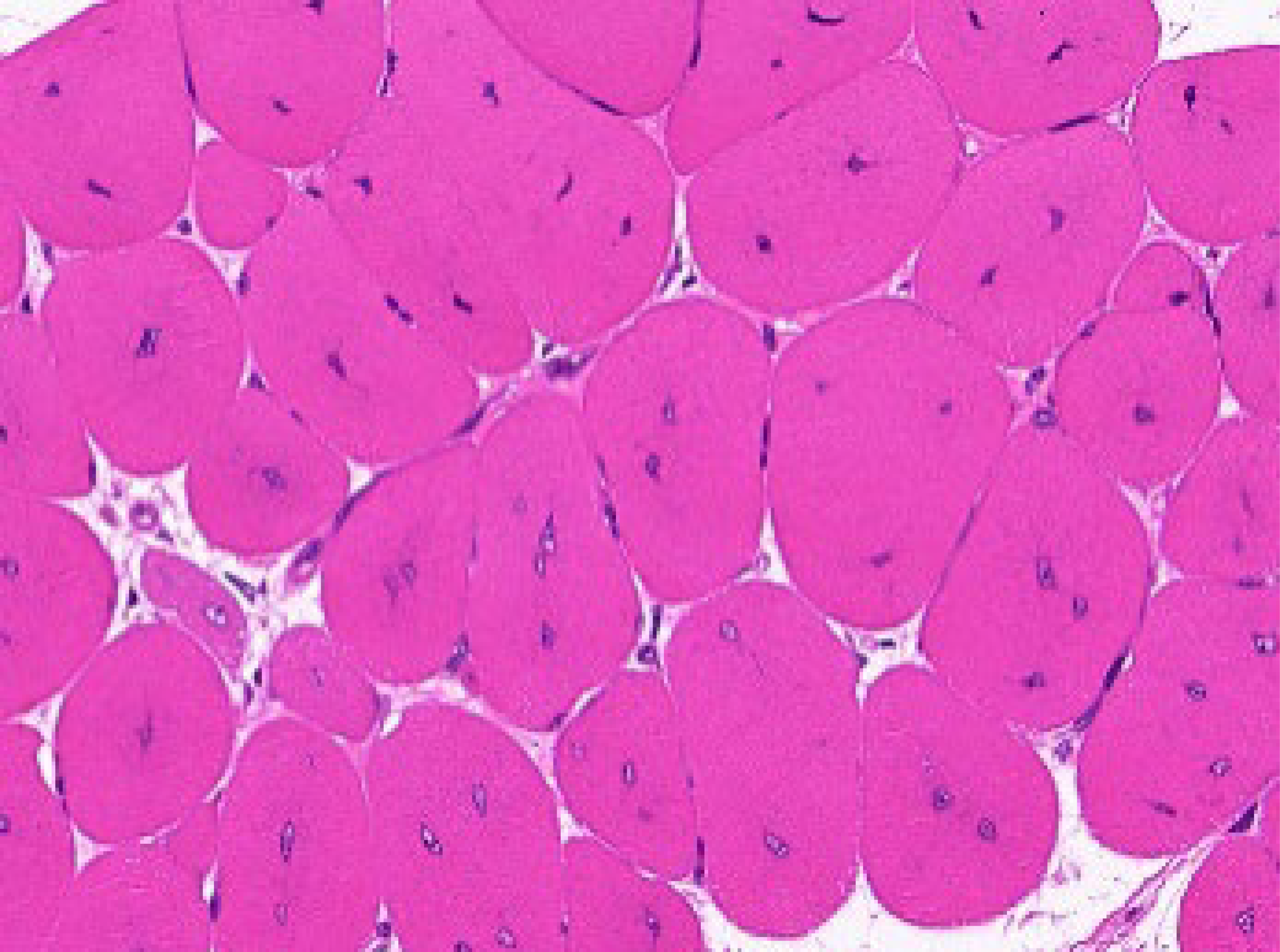

Supplement: Supplementary file 11 — Source data Fig. 8 [file 44318_2024_285_MOESM11_ESM.zip › Fig 8/Fig 8B/8B-mFNDC1-QUA.tif]

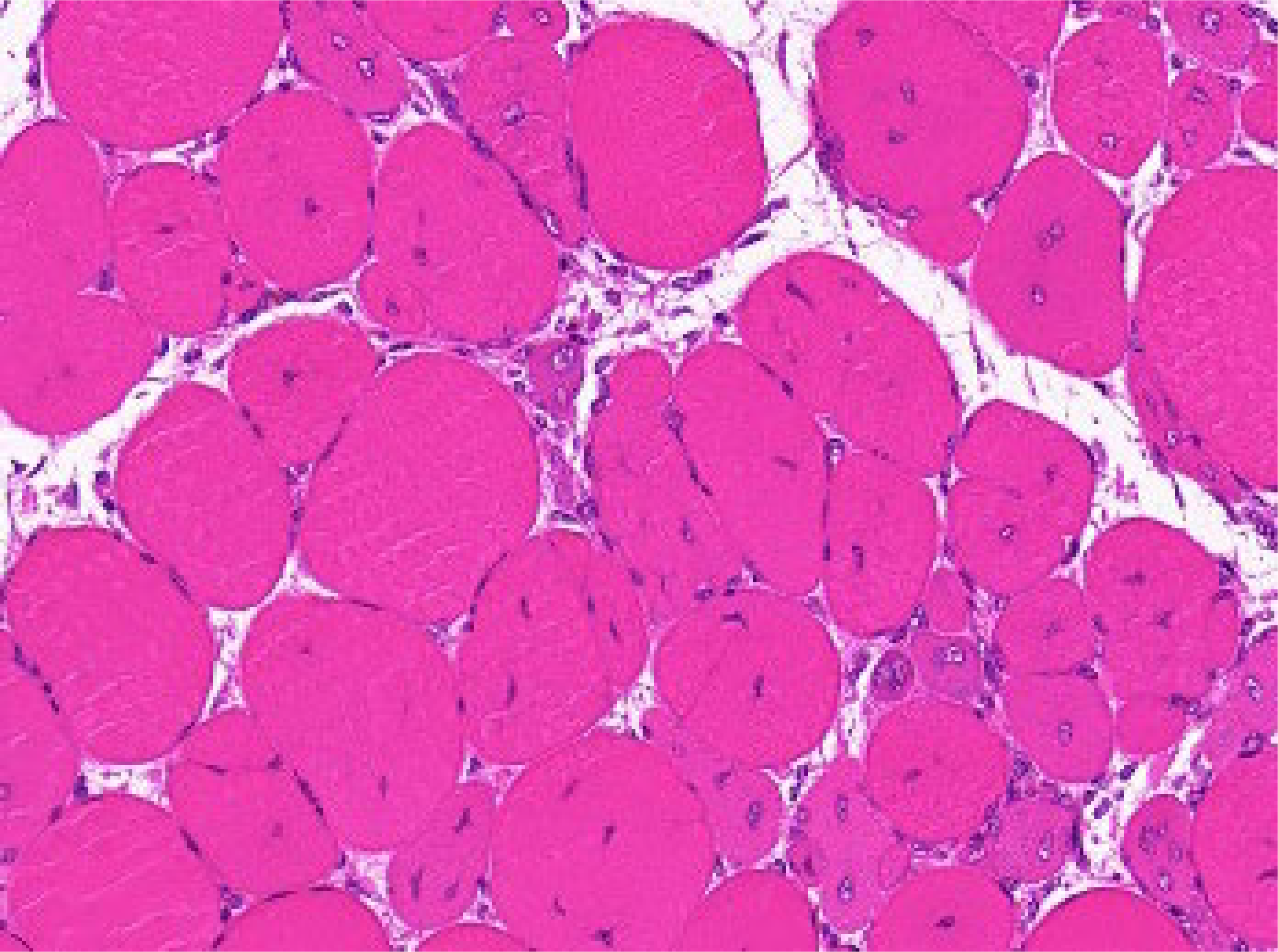

Supplement: Supplementary file 11 — Source data Fig. 8 [file 44318_2024_285_MOESM11_ESM.zip › Fig 8/Fig 8B/8B-mFNDC1-TA.tif]

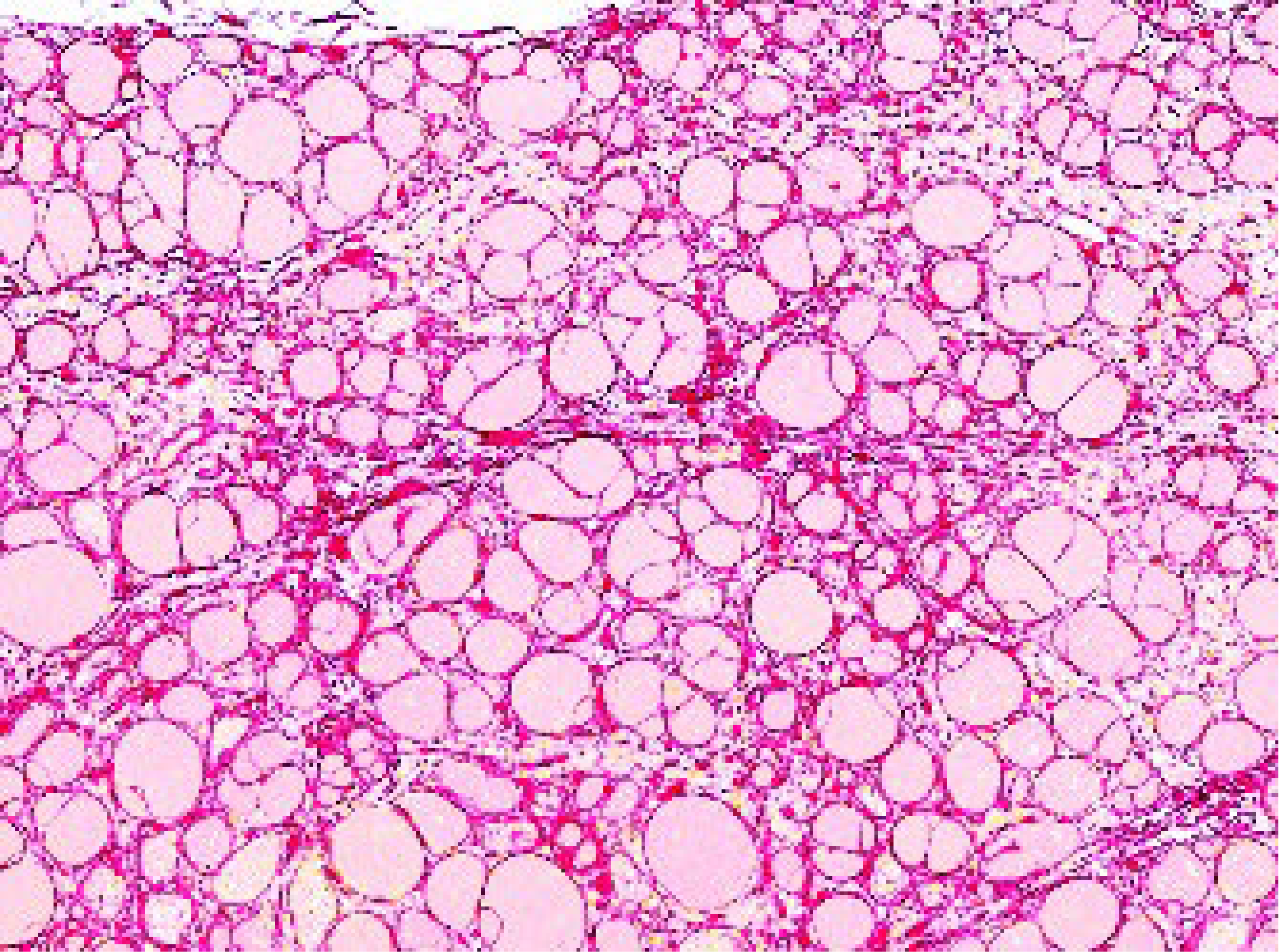

Supplement: Supplementary file 11 — Source data Fig. 8 [file 44318_2024_285_MOESM11_ESM.zip › Fig 8/Fig 8H/8H-Control-DIA.tif]

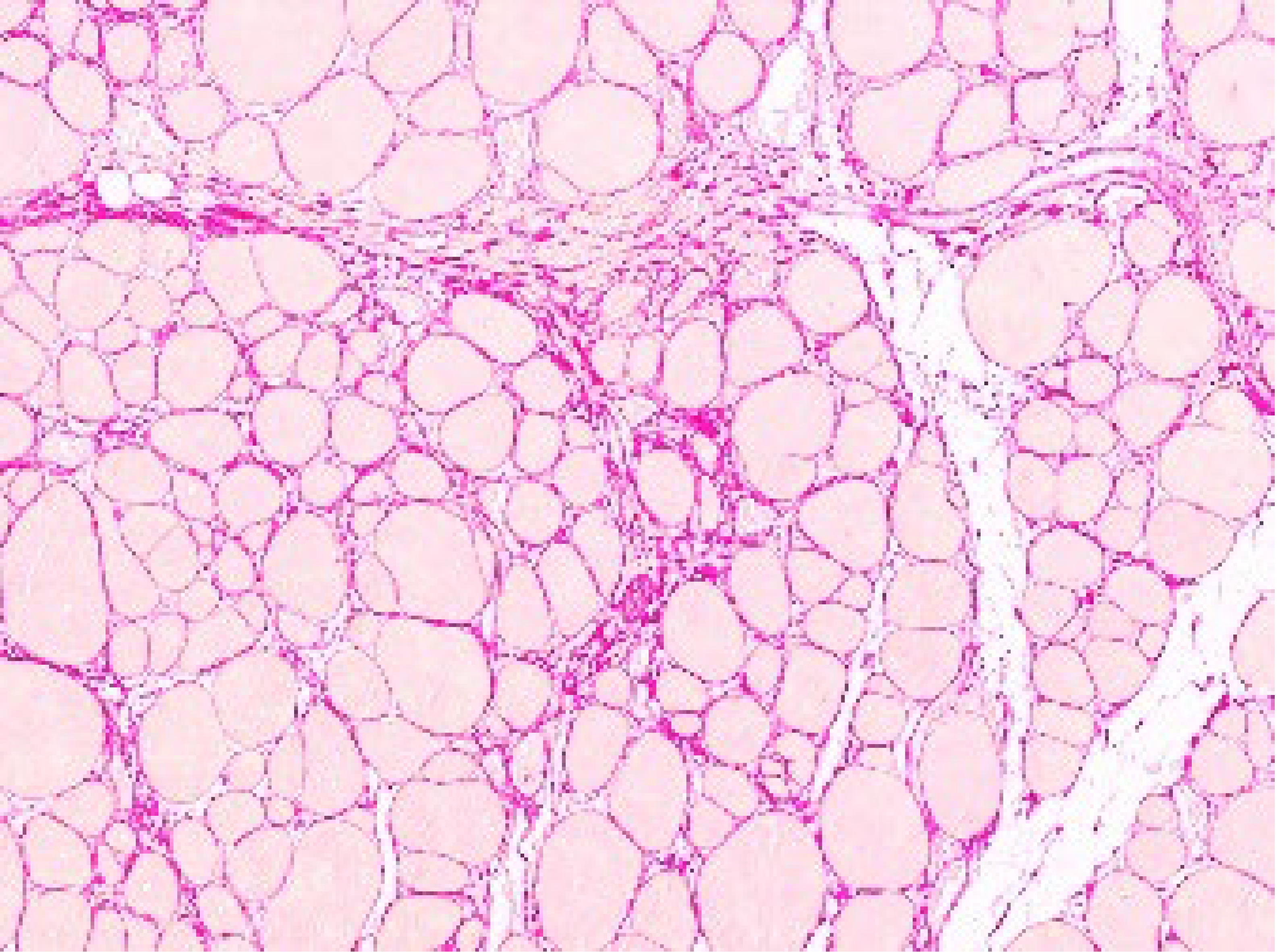

Supplement: Supplementary file 11 — Source data Fig. 8 [file 44318_2024_285_MOESM11_ESM.zip › Fig 8/Fig 8H/8H-Control-QUA.tif]

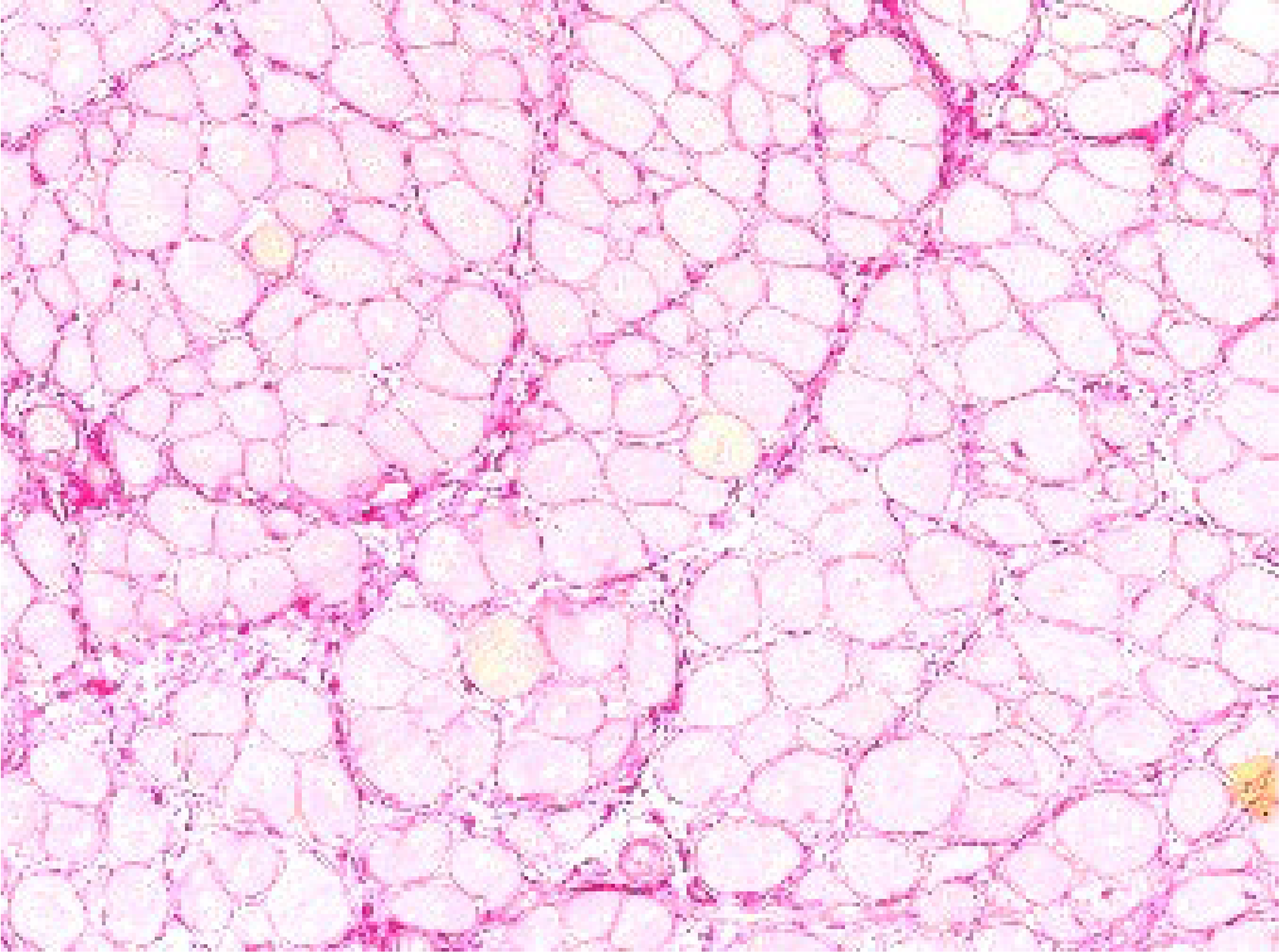

Supplement: Supplementary file 11 — Source data Fig. 8 [file 44318_2024_285_MOESM11_ESM.zip › Fig 8/Fig 8H/8H-Control-TA.tif]

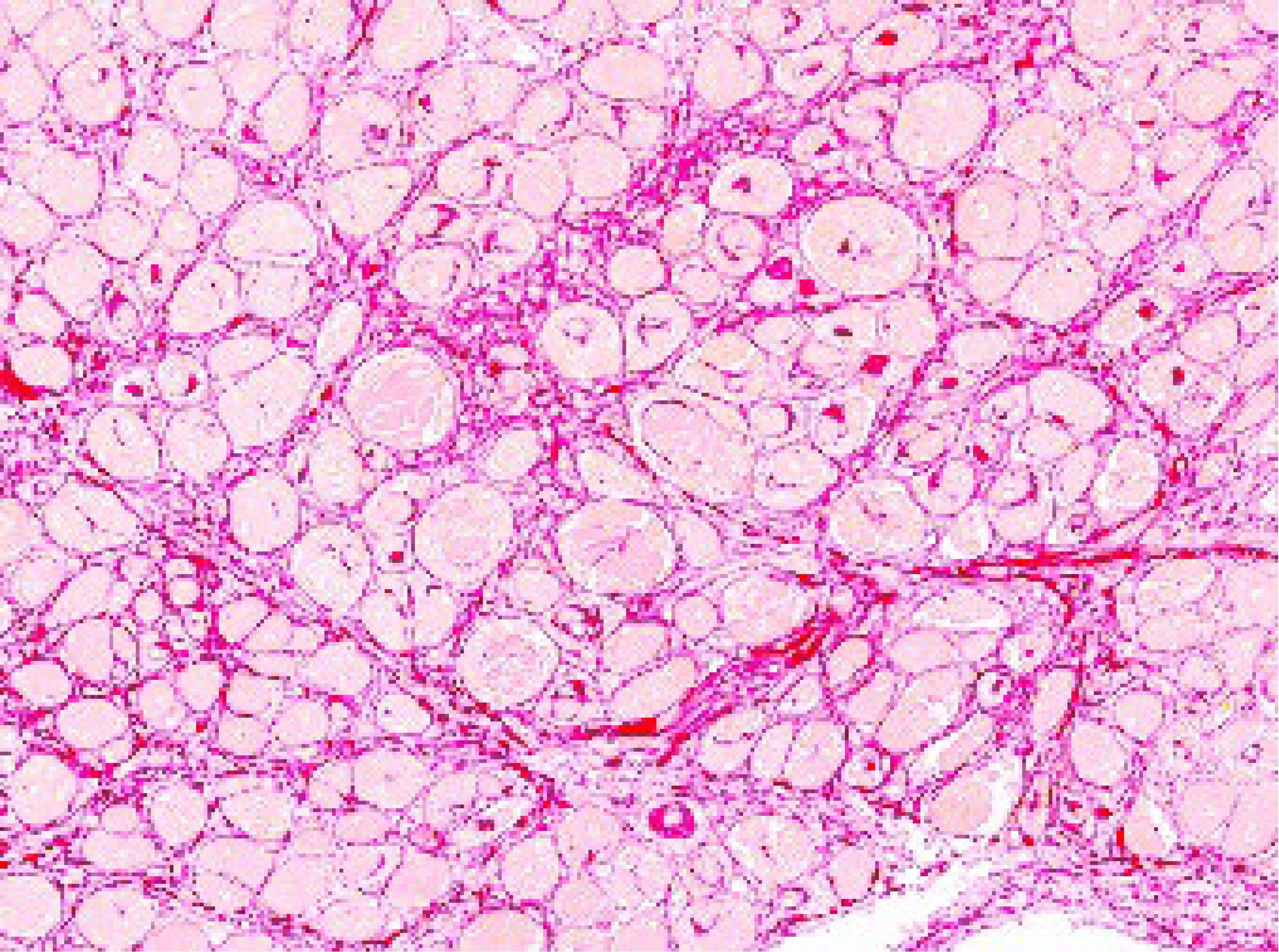

Supplement: Supplementary file 11 — Source data Fig. 8 [file 44318_2024_285_MOESM11_ESM.zip › Fig 8/Fig 8H/8H-mFNDC1-DIA.tif]

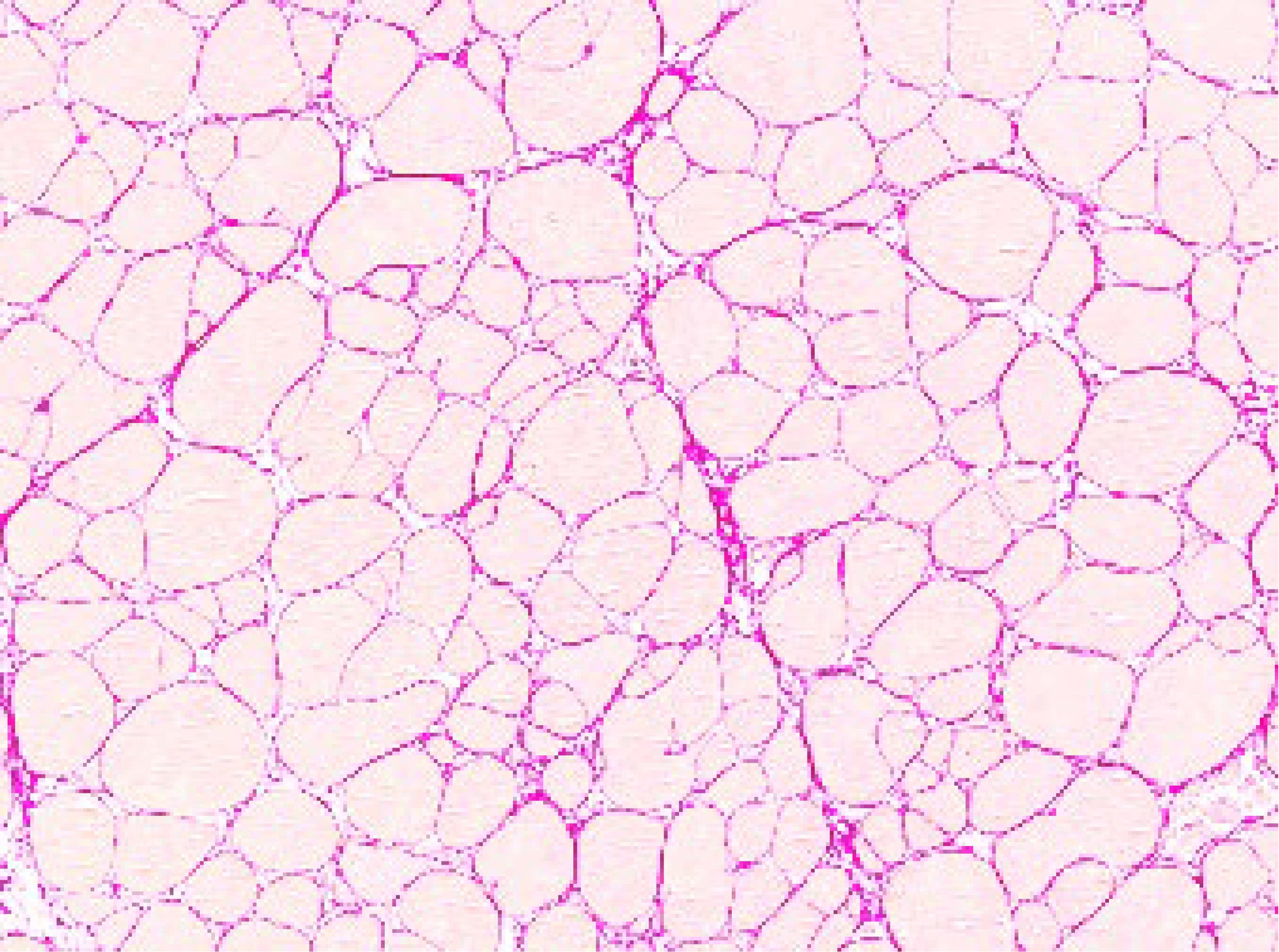

Supplement: Supplementary file 11 — Source data Fig. 8 [file 44318_2024_285_MOESM11_ESM.zip › Fig 8/Fig 8H/8H-mFNDC1-QUA.tif]

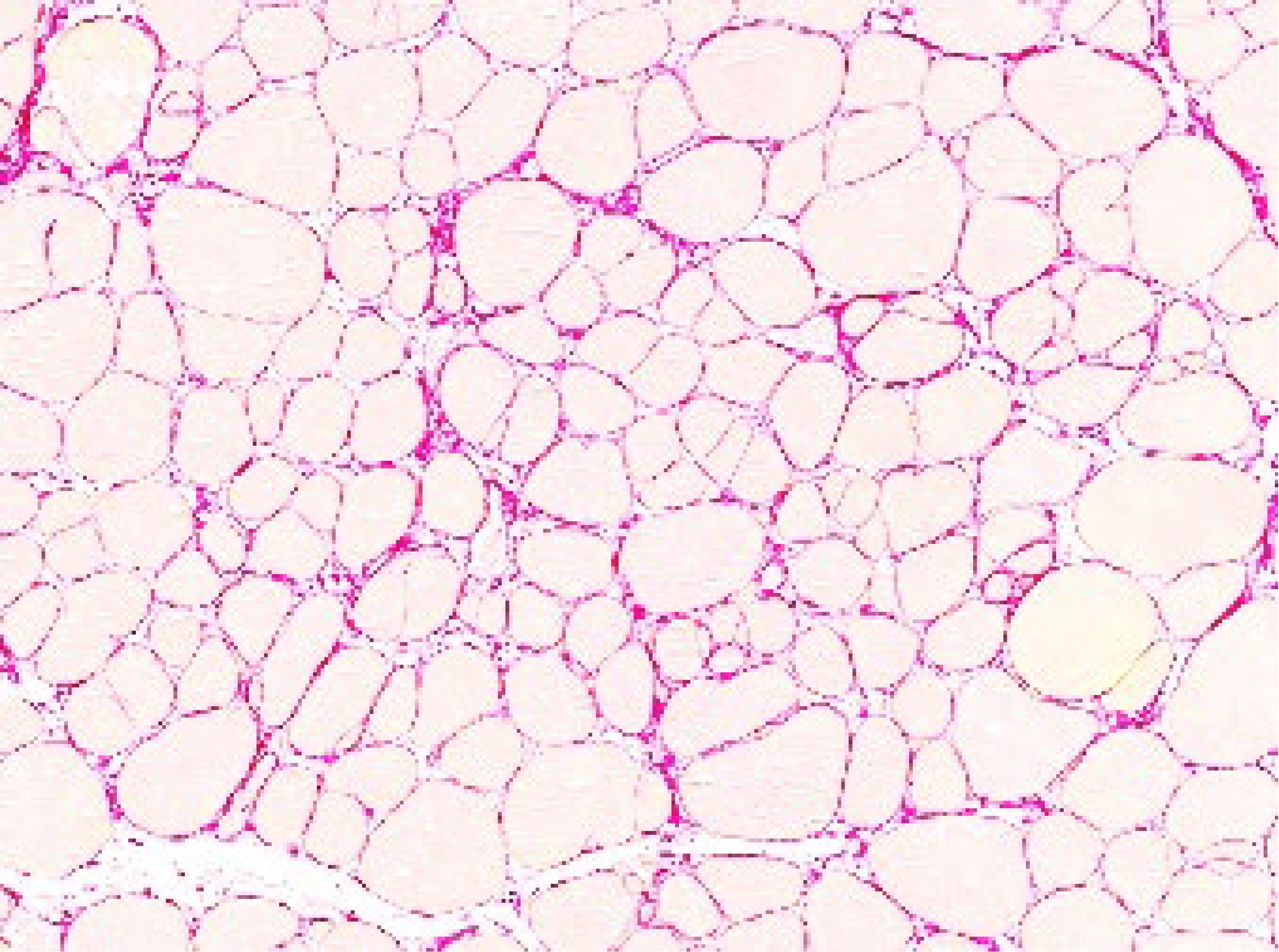

Supplement: Supplementary file 11 — Source data Fig. 8 [file 44318_2024_285_MOESM11_ESM.zip › Fig 8/Fig 8H/8H-mFNDC1-TA.tif]
